# Real‐world reproducibility study characterizing patients newly diagnosed with multiple myeloma using Clinical Practice Research Datalink, a UK‐based electronic health records database

**DOI:** 10.1002/pds.5171

**Published:** 2020-11-21

**Authors:** Anouchka Seesaghur, Natalia Petruski‐Ivleva, Victoria Banks, Jocelyn Ruoyi Wang, Pattra Mattox, Edwin Hoeben, Joe Maskell, David Neasham, Shannon L. Reynolds, George Kafatos

**Affiliations:** ^1^ Department Center for Observational Research Amgen Ltd Uxbridge UK; ^2^ Department Science Aetion, Inc Boston Massachusetts USA; ^3^ VLB Contractors Ltd Kent UK

**Keywords:** epidemiologic methods, healthcare database, observational study, replication, reproducibility, transparency

## Abstract

**Purpose:**

We evaluated the reproducibility of a study characterizing newly‐diagnosed multiple myeloma (MM) patients within an electronic health records (EHR) database using different analytic tools.

**Methods:**

We reproduced the findings of a descriptive cohort study using an iterative two‐phase approach. In Phase I, a common protocol and statistical analysis plan (SAP) were implemented by independent investigators using the Aetion Evidence Platform® (AEP), a rapid‐cycle analytics tool, and SAS statistical software as a gold standard for statistical analyses. Using the UK Clinical Practice Research Datalink (CPRD) dataset, the study included patients newly diagnosed with MM within primary care setting and assessed baseline demographics, conditions, drug exposure, and laboratory procedures. Phase II incorporated analysis revisions based on our initial comparison of the Phase I findings. Reproducibility of findings was evaluate by calculating the match rate and absolute difference in prevalence between the SAS and AEP study results.

**Results:**

Phase I yielded slightly discrepant results, prompting amendments to SAP to add more clarity to operational decisions. After detailed specification of data and operational choices, exact concordance was achieved for the number of eligible patients (N = 2646), demographics, comorbidities (i.e., osteopenia, osteoporosis, cardiovascular disease [CVD], and hypertension), bone pain, skeletal‐related events, drug exposure, and laboratory investigations in the Phase II analyses.

**Conclusions:**

In this reproducibility study, a rapid‐cycle analytics tool and traditional statistical software achieved near‐exact findings after detailed specification of data and operational choices. Transparency and communication of the study design, operational and analytical choices between independent investigators were critical to achieve this reproducibility.

**KEY POINTS**

- Adoption of RWE derived from large databases has resulted in efforts to improve the reproducibility and transparency of research.
- Findings from our reproducibility study using common study documents but two different analytic tools highlighted that at a minimum adherence to standard protocol and reporting guidelines is necessary; however, complete transparency of the study design and operational decisions shared by independent investigators was also critical to a successful reproduction.
- Data source parameters and implementation decisions, especially the nuances of using common data models and enrollment and assessment windows, should be explicitly stated during study planning and protocol development.

## 1 INTRODUCTION

Regulatory, payer, and clinical decision‐makers are increasingly adopting real‐world evidence (RWE) derived from existing real‐world data (RWD), such as longitudinal electronic health records (EHR) and administrative claims data, to support healthcare decisions.^1^, ^2^ The European Medicine Agency (EMA) defines RWD as “*routinely collected data relating to a patient's health status or the delivery of health care from a variety of sources other than traditional clinical trials*.”^1^ An important quality and acceptability criterion for RWE is the ability to replicate studies accurately. The latter requires full transparency of data processing, design and analytic choices, beyond what is typically included in publications. Increasingly, efforts are being made to improve the reproducibility of research by promoting transparency.^3^, ^4^, ^5^, ^6^, ^7^ A reproducible study, defined as “*independent investigators implementing the same methods in the same data and are able to obtain the same results (direct replication)*,”^4^ requires complete access to the study data (i.e., analytic data sets) and methods for sharing codes and software environment as well as ensuring sufficiently detailed study documentation.^7^, ^8^, ^9^ It has also been argued that confirming the findings from observational studies bolsters the overall confidence of scientific evidence.^7^, ^10^

One of the most commonly used UK‐based RWD sources is the Clinical Practice Research Datalink (CPRD) General Practice (GP) Online Data (GOLD).^11^ In a validation study, the CPRD database demonstrated a high positive predictive value of various diagnoses and similar comparisons of incidence with other UK data sources.^12^ The adoption of RWD sources including EHR provides an opportunity to generate clinical evidence in oncology^13^, ^14^ and the CPRD database^14^, ^15^ has been used to evaluate patients with multiple myeloma (MM), a hematological cancer of the bone marrow.^16^ Multiple myeloma, estimated to cause approximately 5700 incident cases of myeloma a year in the UK, is the second most common hematological malignancy in Europe and recognizing MM with nonspecific, multi‐site symptoms is challenging. While several publications using CPRD exist for this patient population,^17^, ^18^, ^19^ the reproducibility of such work using CPRD has not been evaluated. Thus, we sought to evaluate the reproducibility of a study characterizing patients newly diagnosed with MM in CPRD using two different analytic tools.

## 2 METHODS

An iterative two‐phase approach was used to evaluate the reproducibility of a descriptive cohort study using primary care data. In Phase I, two teams of independent investigators implemented a common study protocol and statistical analysis plan (SAP) independently and in‐parallel using different analytic tools, a cloud‐based rapid‐cycle analytic tool, (Aetion Evidence Platform® [AEP], version 3.12), and traditional, line‐programming statistical software (SAS Enterprise Guide version 7.1). Phase II was an iteration of the analyses following the review and comparison of the Phase I findings, implementation decisions, and revisions to study documents (e.g., SAP).

### 2.1 Data source

The population‐based cohort study used the EHRs from the CPRD GOLD database, which contains anonymized longitudinal patient records from more than 600 UK‐based and has primary care medical records of over 18 million patients.^20^ CPRD contains demographic data, medical diagnoses, procedures (including laboratory investigations and results), and death information collected using a standardized form^21^; conditions, interventions and diagnostics use Read codes and medication prescribing recorded using the British National Formulary (BNF). We used CPRD data collected between January 1, 2004 and December 31, 2017 for this study.

Raw CPRD data and raw data converted to Observational Medical Outcomes Partnership (OMOP) CDM (version 4.0) were used for the AEP and SAS analyses, respectively. The latter includes only patients with CPRD data of acceptable quality for research and contains a total of approximately 15 million patients. The raw CPRD data contain approximately 18 million patients, regardless of the data quality.

The research protocol was reviewed and approved by the Independent Scientific Advisory Committee (ISAC, protocol 18_292).

### 2.2 Study population

**FIGURE 1:**
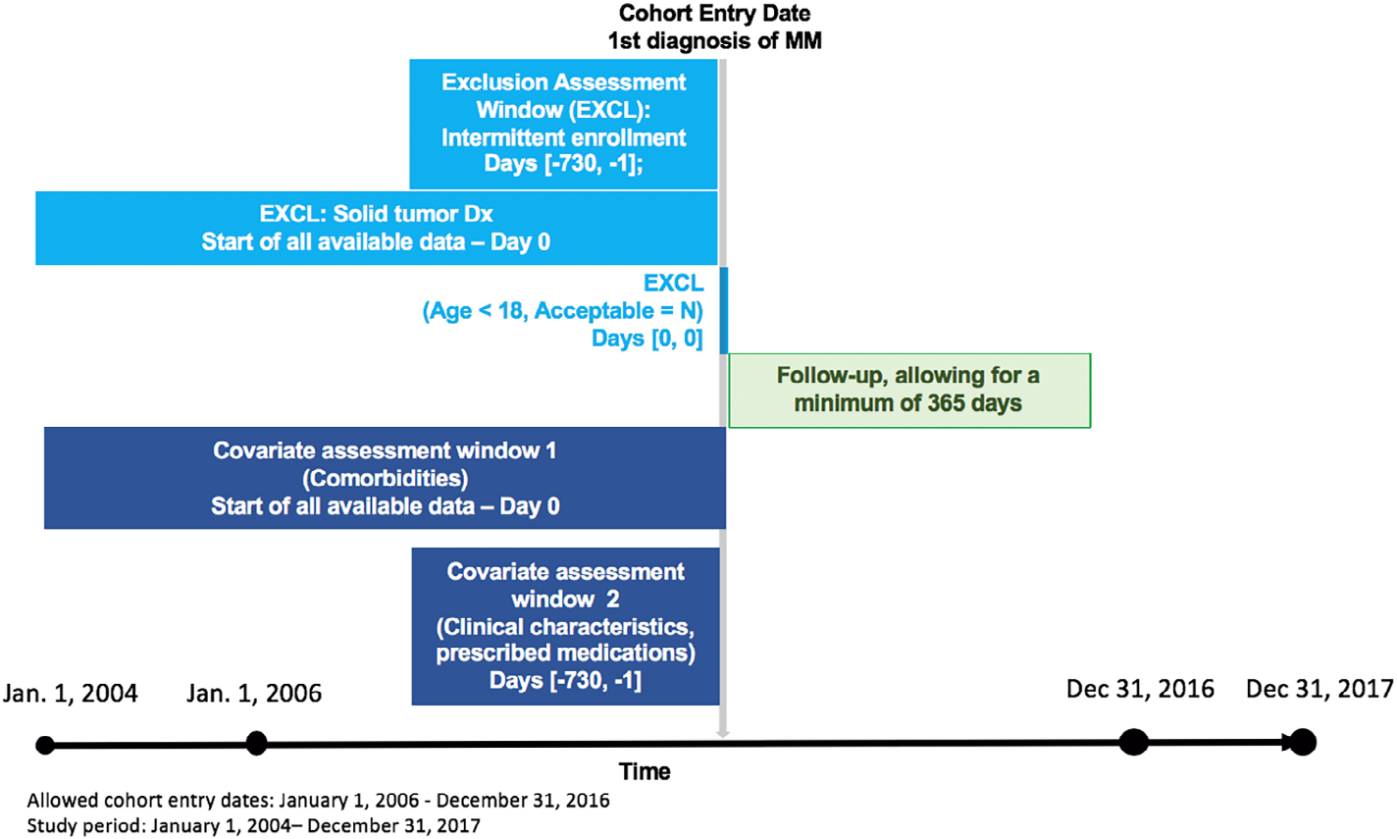
Study design

During the study period (January 1, 2004 to December 31, 2017), we identified patients newly diagnosed with MM between January 1, 2006 through December 31, 2016 to allow for a minimum of 2‐year baseline and 1‐year follow‐up period. Follow‐up began the day after cohort entry and ended at disenrollment from the GP practice, the last data collection date of the practice, death, or end of study period (Figure 1).The study cohort included patients ≥18 years who were registered at the GP for at least 2 years prior to diagnosis and had no history of solid tumors.

### 2.3 Covariates

Several categories of covariates were assessed, including demographics (i.e., age and gender), comorbidities, baseline clinical conditions (i.e., bone pain, and skeletal‐related events [SREs]), prescribed medications, laboratory investigations and laboratory results. Baseline demographics and comorbidities, including cardiovascular disease (CVD), chronic kidney disease (CKD), gout, hypertension, osteopenia, osteoporosis, osteoarthritis, and rheumatoid arthritis (RA), were assessed in all available data prior to MM diagnosis. Baseline clinical conditions, medication prescribed, and laboratory investigation were assessed in the 2 years prior to MM diagnosis. Baseline medications included were prescribed medications related to bone health and pain management, that is, denosumab, prolia, xgeva, bisphosphonates, and analgesics. Laboratory investigation included select diagnostic laboratory tests (calcium, serum creatinine, hemoglobin).

Refer to Data S1 for a list of variables, diagnosis definitions, and covariate definitions.

### 2.4 Statistical analysis

We used descriptive statistics to report the number and proportion of patients meeting the pre‐specified criteria and for binary and categorical covariates, and reported the mean, SD, and range for continuous covariates. The presence of bone complications, such as bone pain and SREs, were assessed during both baseline and in follow‐up.

The two analytic approaches used were AEP, a rapid‐cycle analytic tool that has been previously validated,^3^, ^22^ and SAS software as the gold standard for statistical analyses. The reproducibility of study results was evaluated by calculating the match rate within broad categories (e.g., demographics, conditions, drug exposure, and procedures) and the absolute difference in prevalence between line‐programming and rapid analytics for each individual characteristic included within the broad category. Match rate was defined as a percentage (rounded to the nearest tenth), calculated as the number of individual characteristics with an exact match in the estimated prevalence between the AEP and SAS results, divided by the total number of characteristics within each broad category. An absolute difference in prevalence of 0% and a match rate of 100% represented exact concordance. After the Phase I analysis, the two teams of independent investigators performed a careful review of the results to identify potential reasons for discrepancies and resolve them for the Phase II analysis. Thus, recommendations to promote transparency in study protocols and SAPs were developed.

## 3 RESULTS

### 3.1 Phase I: Initial analysis

In Phase I, the two analytic approaches yielded near‐identical number of eligible patients with newly‐diagnosed MM during the study period (N = 2646 in AEP vs. N = 2648 in SAS). Exact concordance for the distribution of gender and median age was observed; however, there were small discrepancies in patient distribution across age categories and demographic characteristics (absolute difference range of 0.0% to 1.1%) (Table 1). High agreement was observed for the prevalence of comorbidities (CVD, CKD, gout, hypertension, osteoarthritis, osteopenia, osteoporosis, and RA), with a match rate of 75% and an absolute difference in prevalence of 0.0%–0.3%. Agreement was observed for several clinical characteristics of symptomatic bone pain and SREs (match rate of 50% and 85.7%, respectively). The absolute difference for SREs prevalence ranged from 0.0%–0.2%, with exact agreement for pathological fracture, spinal cord compression, radiation therapy to bone and surgery to bone.

**TABLE 1:** Results from the reproducibility assessment between SAS and AEP in the Phase I (initial analysis) and Phase II (revised analysis)

Broad category	Definition used	Description	Phase 1: Initial analysis		Phase 2: Revised analysis	
			Absolute difference^a^	Match rate^b^ AEP versus SAS (%)	Absolute difference^a^	Match rate^b^ AEP versus SAS (%)
**Observations**	**READ codes**	**NDMM patients, n**	2	N/A	0	N/A
**Demographics**	**N/A**	**Age, gender**	0.0%–1.1%	50% (1/2)	0.0%	100% (2/2)
**Conditions**	**READ codes**	**Comorbidities**	0.0%–0.3%	75% (6/8)^c^	0.0–0.1%	87.5% (7/8)^c^
	**READ codes**	**Symptoms (bone pain)**	0.0%–0.1%	50% (2/4)	0.0%	100% (4/4)
	**READ codes**	**Clinical events (SREs)**	0.0%–0.2%	85.7% (6/7)	0.0%	100% (7/7)
**Drug exposure**	**Product codes**	**Record of specific drug**	0.0%–19.6%	28.6% (4/14)	0.0%	100% (14/14)
	**Product codes**	**Absence of drug**	10.8%	0% (0/1)	0.0%	100% (1/1)
**Procedure**	**ENTTYPE** **READ codes**	**Laboratory investigation and results**	0.0%	100% (3/3)	0.0%	100% (3/3)

Abbreviations: AEP, Aetion Evidence Platform; N/A, not applicable; NDMM, newly diagnosed multiple myeloma.

^**a**^ Absolute difference is defined as the range of difference (in forms of either counts or percentages) between the AEP and SAS results within each category.

^b^ Match rate, AEP versus SAS, is defined as a percentage calculated as the number of variables with an exact match in the estimated prevalence between the AEP and SAS results, divided by the total number of variables within each category.

^c^ One patient had chronic kidney disease, osteoarthrosis, and gout. However, since the absolute difference in prevalence for osteoarthritis and gout were 0.0%, those conditions were considered concordant and contributed to the match rate numerator.

We observed a lower agreement on specific drug exposure (match rate of 28.6% and an absolute difference in prevalence of 0.0%–19.6%). Exact concordance was also observed for laboratory investigations (hypercalcemia, renal impairment, and anemia) and valid test results. The Phase I analysis yielded several discrepancies which were subsequently addressed and resolved in the Phase II analysis by clarifying operational decisions not fully specified in the protocol or SAP.

### 3.2 Phase II: Revised analyses (analysis iteration based on Phase I findings)

**FIGURE 2:**
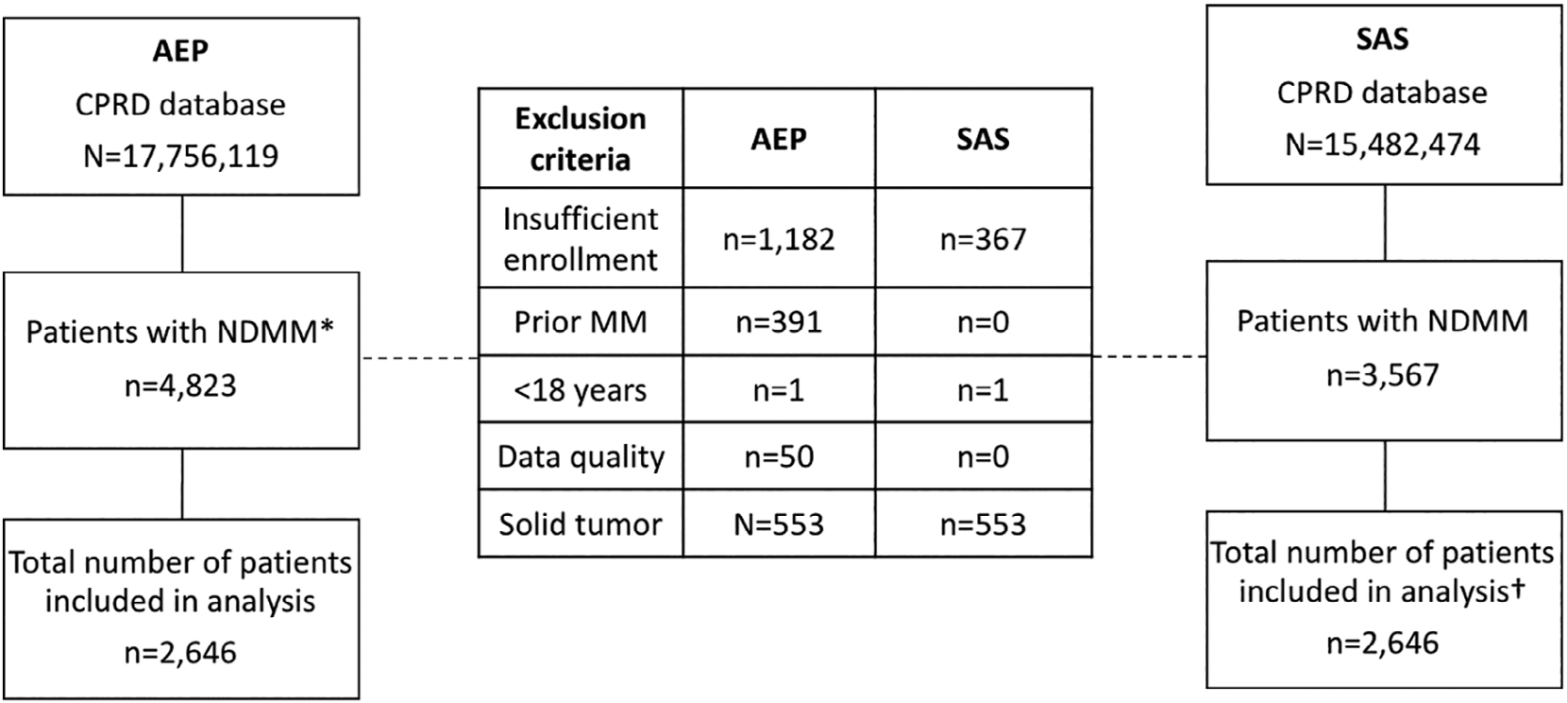
Flow diagram of patient inclusion after reconciliation in Phase 2. AEP, Aetion Evidence Platform; CDM, common data model; MM, multiple myeloma; NDMM, newly‐diagnosed multiple myeloma; OMOP, Observational Medical Outcomes Partnership; SAS, Statistical Analysis System. *Note:* Analyses performed using SAS data used CPRD data converted to Observational Medical Outcomes Partnership (OMOP) common data model (CDM); analyses conducted using AEP used the raw data. The AEP analysis applied the CPRD acceptable flag after identifying patients with MM. The former database includes only patients with CPRD data of acceptable quality for research and contains a total of approximately 15 million patients. The raw CPRD data contains approximately 18 million patients, regardless of the data quality. *A total of n = 391 patients with NDMM met the cohort entry criteria of first MM diagnosis; however, these n = 391 patients were subsequently excluded due to prior MM. †Near‐identical number of eligible patients with newly diagnosed MM was achieved in Phase I (N = 2646 in AEP vs. N = 2648 in SAS). Two patients identified using SAS was due to different assumptions made about which data tables (i.e., clinical, referral, and test tables) to query patients with specific disease diagnoses

Following further detailed specification of study choices that led to different interpretations during Phase I, the analysis was repeated and a 100% match in the number of patients (N = 2646), demographics, bone pain, SREs, drug exposure, laboratory investigations, and valid laboratory results was achieved. A negligible discrepancy remained in the estimated prevalence of CKD (absolute difference 0.1%), as well as osteoarthritis and gout (absolute different 0.0%), driven by the data of an individual patient with multi‐morbidity (Figure 2, Table 1).

### 3.3 Comparison of two approaches in Phase I and Phase II

A mismatch in aggregated results triggered a cascade of steps to investigate and remediate discrepancies identified during Phase I. As a first step, the two set of independent investigators reviewed the protocol and SAP definitions against implementation and discussed individual interpretations, such as assumptions and algorithms used, followed by a more detailed investigation into time interval specifications, which resolved a majority of the discrepancies. For any remaining discrepancies, the investigators reviewed patient‐level data. Amending study documents (e.g., SAP) was an iterative process.

**FIGURE 3:**
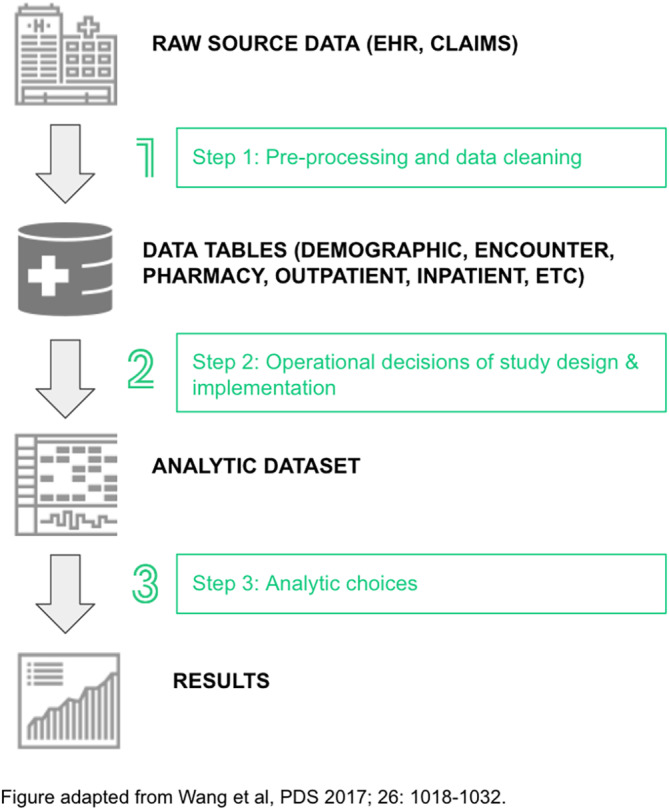
Decisions surrounding the workflow from raw data source to study results

Table 2 displays the sources of the discrepancies in Phase I and the operational decisions utilized in Phase II. The workflow – and transparency during each step – for conducting database studies using routine‐collected longitudinal healthcare data begins with the (1) processing of raw data to (2) developing the study design and operational decisions, and finally (3) deciding on analytical choices. Each step provides opportunities to develop sufficiently clear study documentation (Figure 3).^4^ Lack of specificity in these steps during protocol development and SAP contributed to initial discrepancies.

**TABLE 2:** Improvement of operational decisions for Phase II following identification of discrepancies observed in Phase I

Variables	Source of discrepancies and match rate <100% in Phase I	Final operational decisions for Phase II
**Observations** NDMM patients (n)	Realization that patient diagnoses were captured across different data tables (i.e., clinical, referral, and test tables) in CPRD, leading to differing use of data tables by the independent investigators to identify patients with MM	Consensus regarding which data tables to use for identifying patients with MM diagnosis Inclusion of relevant diagnoses from only the Clinical and Referral tables Non‐use of diagnoses in the Test table
**Demographics** Age Gender^a^	Difference in methods used for handling date data to impute missing age when only year and month of birth are available	Consensus on rules for age calculation: The year of birth (YOB) was defined as YOB + 1800 If month of birth (MOB) was available, the month of birth was defined as MOB, and the day of birth defined as day 15 of the month If MOB was not available, then the month and date of birth was defined as June 30th
**Conditions** Co‐morbidities Symptoms (bone pain) Clinical events (SREs)	Lack of specificity in defining the baseline assessment window (e.g., whether or not to include cohort entry date for assessing chronic conditions such as osteoporosis)	Clarification of time assessment windows: Covariate assessment window for comorbidities to include the cohort entry date (Day 0), based on the assumption that the chronic conditions cannot be cured Covariate assessment window for clinical characteristics (i.e., symptoms, clinical events) to exclude the cohort entry date (Day 0) to assess bone pain and SREs exclusively prior to the MM diagnosis
	Realization that patient diagnoses were captured across different data tables (i.e., clinical, referral, and test tables) in CPRD, leading to differing use of data tables by the independent investigators to identify patients with specific comorbidities	Consensus regarding which data tables to use for identifying patients with specific comorbidities: Inclusion of relevant diagnoses from only the Clinical and Referral tables Non‐use of diagnoses in the Test table
	Differences in data structure of CPRD (i.e., use of transformed data [OMOP CDM structure] in SAS programming), likely contributed to the remaining small discrepancies that were present in comorbidities only	Consensus that the remaining data‐driven discrepancies were unresolvable The rapid‐cycle analytics tool used raw CPRD and the in‐line programming analyses used CPRD data pre‐mapped to OMOP CDM
**Drug exposure** Record of specific drug Absence of drug	Lack of specificity in exposure assessment window (i.e., when is the start date, and whether or not to include cohort entry date in the baseline period)	Agreement on the time assessment window: Covariate assessment window for drug exposures to start from the 730 days prior till 1 day prior to the index date (Day 0) to assess treatment usage exclusively prior to the MM diagnosis
	Lack of a definition for the absence of treatment led to the use of different algorithms by the independent investigators. For instance, patients without any occurrence of analgesic versus patients who did not start any analgesics during the baseline period	Agreement on the definition for absence of treatment: Absence of treatments was measured as not starting any analgesics during the baseline period
**Procedures** Laboratory investigation	Not applicable; exact concordance in phase I	New decision to facilitate interpretation of the data: For investigation, we included any tests (not requiring valid test only)

^a^ Exact concordance achieved for gender in Phase I.

#### 3.3.1 Transparency in raw data processing

Our direct replication study highlights the importance of understanding, communicating and documenting data source parameters (e.g., data extraction date, data source range, data cleaning and transformation) in the initial stage of conducting a database study. In the current study, the independent investigators used the same data source (CPRD), the same data cut (June 22, 2018) and the same data range (November 21, 1987 – June 30, 2018); however, different data model conversions and hence different data structures were used. The rapid‐cycle analytics tool used raw CPRD data processed with a so‐called “adaptive rule system” whereas the SAS analyses were based on CPRD data that had been pre‐mapped to the OMOP CDM. There are pros and cons to both data model approaches.^23^ The OMOP CDM allows the construct of a standard vocabulary for different medical concepts; however, a preconfigured CDM could potentially result in information loss due to incomplete mapping of construct terms.^24^, ^25^ The remaining negligible discrepancies might have arisen from the small loss of information after applying the CDM.

Despite differences in the timing of data processing to exclude patients with poor quality data from the analysis, both analytic approaches eventually yielded the same number of eligible patients in the analysis set upon reconciliation of the implementation approach (Figure 2). In the OMOP CDM data, patients and practices that were considered unacceptable for research were omitted during the extraction, transformation, and loading (ETL) process of data mapping and conversion; these analyses were based on acceptable CPRD data for research from the beginning. In contrast, the AEP analyses applied the data quality checks (e.g., “Up To Standard (UTS)” dates and GP practices considered “acceptable for research”) as an exclusion criterion when identifying patients with MM.

#### 3.3.2 Transparency in study design and operational decisions

In the current study, a common study protocol, implemented by two teams of independent researchers, included an initial study design schema, operational definitions of all variables, and appended code lists and algorithms. A SAP was also co‐developed, which included details of the analyses to be undertaken (e.g., handling of missing data). Despite these common study materials capturing the operational and analytical choices, several discrepancies were obtained in Phase I of the study. Subsequent thorough investigations revealed various reasons (Table 2), such as differences in the interpretation of time assessment periods including the eligibility enrollment window.

##### Eligibility enrollment window

The enrollment window for the inclusion and exclusion criteria is the time window prior to the patient's study entry date, often called the cohort entry date or index date. Different interpretations of the time assessment periods specified in the protocol and SAP were employed. For example, terms such as “baseline” did not specify, for each covariate, whether the cohort entry date should or should not be included in the assessment period.

#### 3.3.3 Transparency in analytical choice decisions

Several discrepancies were due to investigator‐driven interpretation of variable definitions, algorithms, and the exposure assessment window, which were resolved following alignment between both investigators. The remaining discrepancies were data‐driven due to differences in data structure and could not be fully addressed in Phase II.

##### Variable definitions and algorithms

While the variable definitions in the protocol and SAP included a general description of the algorithm and the code list for diagnoses, comorbidities, and clinical conditions, different assumptions were made by the two sets of independent investigators. First, a lack of detail regarding which specific data tables within CPRD to query accounted for the initial discrepancies for all diagnosis events in our study, including MM diagnosis, comorbidities, and baseline clinical conditions. To avoid discrepancies, study documentation should specify both the relevant data fields as well as the data tables to query these fields, depending on the data source used. Second, the inherent logic for certain variable with complicated algorithms was not specific, such as Boolean logic between various components of the algorithms. For example, for events spanning multiple days, such as medication use or hospitalizations, it is important for study documentation to specify whether a patient should be counted as having the medication or event if the event overlaps the time window of interest or only if it begins during the time window of interest.

##### Exposure assessment window

The exposure assessment window describes the time window for identifying the exposure status. A similar lack of specificity in this time window accounted for the initial discrepancies between baseline medication use and comorbidities from the two sets of results. While it was specified that baseline medication use would be assessed during the baseline period, one approach included a look back of 2 years and the other of all available data.

## 4 DISCUSSION

In this direct replication study characterizing newly‐diagnosed MM within a UK‐based EHR, after a thorough investigation on the initial discrepancies, we achieved an exact match of the analytic population and nearly 100% match rate using two different analytic approaches, namely a rapid‐analytics tool and traditional statistical software. This replication exercise demonstrated that differences in study results are often due to insufficient level of detail in the study protocol or SAP.

### 4.1 Standards in transparency and reproducibility

Transparency of the study design and operational decisions shared by the independent investigators were critical to facilitating this direct replication study, highlighting the importance of not only following standard protocol and reporting guidelines (Box 1), but applying greater specificity and transparency to protocol development in the study planning phase. These guidelines on conducting^26^, ^27^, ^28^, ^29^ and reporting^30^, ^31^, ^32^ non‐randomized studies have been developed by professional societies, governmental agencies, and pharmacoepidemiological experts. More recently, a ISPOR‐ISPE joint task force convened to develop recommendations to ensure RWE can approximate the gold‐standard randomized controlled trial and provide “causal conclusions.”^33^ The first report from this joint task force provided recommendations for transparency in the “study hygiene” (i.e., planning and procedures) when evaluating treatment and/or comparative effectiveness studies using RWD.^34^ One recommendation focuses on replication to obtain the same results using the same data and same analytic methods, explaining “*Full transparency in design and operational parameters, data sharing, and open access in clinical research will not only increase confidence in the results but will also foster the reuse of clinical data*.” The second ISPOR‐ISPE joint task force paper^4^ further identifies specific parameters requiring full transparency and explicit reporting of key decisions regarding the data source and study design: data source, overall study design, inclusion/exclusion criteria, exposure definitions, follow‐up time, outcome definition, covariate definitions, control sampling, and statistical software. Our findings highlight that second order temporal anchors (such as enrollment window, covariate assessment window, follow‐up window, exposure assessment window) should be well specified prior to the analysis being undertaken. In a large‐scale reproducibility study of 6 protocols and 31 publications, Wang et al. also identified this lack of clarity on temporal anchors as one of the challenges for replicating source studies.^3^ Including a standardized visual representation of the study design (such as Figure 1) and key time anchors in the protocol of studies using longitudinal health care databases would preclude such temporal ambiguities.^35^ While best practices in reporting studies using RWD exist (Box 1), our study highlighted that key parameters regarding data sources and important operational decisions (e.g., the boundaries of the enrollment and time assessment windows) should be explicitly stated during study planning and the protocol development stage.

Box 1 1 **Standard considerations for ensuring transparency and reproducibility of real‐world research protocols and findings**

- Follow standard protocol^28^, ^29^ and reporting guidelines^30^, ^31^, ^32^ developed and endorsed by governmental agencies^26^, ^27^ professional societies,^4^, ^34^ and other experts to ensure necessary study parameters are fully described and reported.
- Ensure the protocol and reporting include operational definitions of all variables, including exposures, outcomes, potential confounders; include codes lists and algorithms.^28^, ^29^, ^31^
- Consider reporting supplemental information that specifies access to the protocol, raw data, or programming codes.^30^
- Describe statistical methods including how missing data were handled^28^, ^31^ and statistical software program utilized.^4^
- Define critical time periods and temporal anchors in the study design such as study entry date, enrollment windows, washout or lookback periods, exposure assessment windows, and follow‐up window.^4^ Standardized design diagrams allow for a quick visual representation of key temporal parameters.^4^, ^36^ Such time periods should be clearly specified and included in study planning documents.
- Understand and fully report pertinent data source parameters such as data extraction date, data source range, data cleaning and transformations when reporting the study methods and findings^4^ (e.g., manuscripts) as well as in study documents (e.g., protocols).

### 4.2 Strengths and limitations

One of the key strengths of our study was the large number of patients newly diagnosed with MM identified in CPRD and the availability of symptoms and specific diagnostic investigations for MM in the CPRD database, allowing us to investigate the reproducibility of these findings. Another strength is the achievement of nearly 100% concordance in the final analysis using the two different analytic approaches. While other studies published in the literature previously conducted reproducibility studies,^3^ very few studies evaluated the reproducibility among patients newly diagnosed with MM. Ensuring the availability of a study's data, methods, and documentation, as outlined by Peng et al,^7^ will also facilitate reproducibility in epidemiologic research and improve the confidence of observational study findings. The current study, however, is limited to structured data only and does not cover information captured in free text fields and/or non‐automated test results.

## 5 CONCLUSION

In conclusion, this direct replication study characterizing patients newly diagnosed with MM within a UK‐based EHR database demonstrated that study reproducibility requires maximal transparency at each phase of RWE generation. Following standard protocol and reporting guidelines allowed a rapid‐cycle analytics tool to achieve near‐exact replication of findings obtained using traditional statistical software. Specificity of the study design and key operational decisions shared by independent teams of investigators were critical to achieve this successful reproducibility.

## CONFLICT OF INTEREST

The following personal or financial relationships relevant to this manuscript existed during the conduct of the study: JRW, PM, SLR are employees of and hold stock options or equity in Aetion; NPI was an employee of Aetion and held stock options. AS, JM, DN, GK are employees of and hold stock options in Amgen. During the study conduct and reporting, VB and EH were contract workers for Amgen.

## ETHICAL STATEMENT

The research protocol was reviewed and approved by the Independent Scientific Advisory Committee (ISAC, protocol 18_292).

## Supporting information


**Data S1.** Supplementary Information.
