# Supplementary material for: Real‐world reproducibility study characterizing patients newly diagnosed with multiple myeloma using Clinical Practice Research Datalink, a UK‐based electronic health records database
Source: Pharmacoepidemiol Drug Saf. 2020 Nov 21;30(2):248–56. doi: 10.1002/pds.5171 (PMC7984077; doi:10.1002/pds.5171)
Supplement: Supplementary file 1 — Data S1. Supplementary Information. [file PDS-30-248-s001.docx]

**Supplementary Appendix**

ANNEX 1: List of stand-alone documents

| Document No. | Name |
| --- | --- |
| ANNEX 2 | Covariates included in the replication analysis |
| ANNEX 3 | Definition of multiple myeloma (MM) as the cohort entry criteria |
| ANNEX 4 | Definition of bone pain symptoms |
| ANNEX 5 | Definition of skeletal-related events (SRE) |
| ANNEX 6 | Definition of baseline comorbidities |
| ANNEX 7 | Definition of concomitant medications |
| ANNEX 8 | Algorithms of CRAB investigation and confirmation (laboratory records) |
| ANNEX 9 | Definition of CRAB investigation and confirmation (clinical records) |

ANNEX 2. Covariates included in the replication analysis

| **Characteristics** |
| --- |
| **All patients** |
|  |
| **Gender and age category** |
| Gender (male) |
| Age group at MM diagnosis (18-39 years) |
|  |
| **Comorbidities** |
| Osteopenia |
| Osteoporosis |
| Osteoarthritis |
| Gout |
| Rheumatoid arthritis |
| Chronic Kidney Disease |
| Cardiovascular Disease |
| Hypertension |
|  |
| **SRE** |
| Pathological fracture: non-vertebral (excluding skull, face, fingers, toes) |
| Pathological fracture: non-vertebral (skull, face, fingers, toes) |
| Pathological fracture: not specified |
| Pathological fracture: vertebral |
| Radiation therapy to bone |
| Spinal cord compression |
| Surgery to bone |
|  |
| **Site specific bone pain** |
| Rib pain |
| Chest pain |
| Back pain |
| Joint pain |
|  |
| **Medications** |
| Prolia |
| Xgeva |
|  |
| **Bisphosphonates** |
| Alendronic acid |
| Etidronate |
| Ibandronic acid |
| Pamidronic acid |
| Risedronic acid |
| Sodiumclodronate |
| Tiludronic acid |
| Strontium ranelate |
| Zoledronic acid |
| Non-opioid analgesics |
| Weak opioids |
| Strong opiods |
|  |
| **Absence of drug** |
| No analgesics |
|  |
| **Lab investigation** |
| Hypercalcaemia |
| Renal impairment |
| Anaemia |

ANNEX 3. Definition of multiple myeloma (MM) as the cohort entry criteria

| **READ code** | **READ term** |
| --- | --- |
| **Multiple myeloma** | |
| B63..00 | Multiple myeloma and immunoproliferative neoplasms |
| B630.00 | Multiple myeloma |
| B630.11 | Kahler's disease |
| B630.12 | Myelomatosis |
| B630300 | Lambda light chain myeloma |
| B631.00 | Plasma cell leukaemia |
| B63z.00 | Immunoproliferative neoplasm or myeloma NOS |
| N330900 | Osteoporosis in multiple myelomatosis |
| 4C53.00 | Bone marrow: myeloma cells |
| BBn0.00 | [M]Plasma cell myeloma |
| BBn..00 | [M]Plasma cell tumours |
| BBn0.11 | [M]Multiple myeloma |
| BBn0.12 | [M]Myeloma NOS |
| BBn0.13 | [M]Myelomatosis |
| BBn0.14 | [M]Plasmacytic myeloma |
| BBn3.00 | [M]Plasma cell tumour, malignant |

ANNEX 4. Definition of bone pain symptoms

| **READ code** | **READ term** |
| --- | --- |
| **Bone pain - combined** | |
| 1826.00 | Parasternal pain |
| 1829.00 | Retrosternal pain |
| 1973.00 | Left subcostal pain |
| 1974.00 | Right subcostal pain |
| 16C5.00 | C/O - low back pain |
| 16C6.00 | Back pain without radiation NOS |
| 16C9.00 | Chronic low back pain |
| 16CA.00 | Mechanical low back pain |
| 182B.00 | Rib pain |
| 182B000 | Costal margin chest pain |
| 197..14 | Subcostal pain |
| 1A53.12 | C/O - lumbar pain |
| 1M00.00 | Pain in elbow |
| 1M00.11 | Elbow pain |
| 1M01.00 | Pain in wrist |
| 1M10.00 | Knee pain |
| 1M12.00 | Anterior knee pain |
| 1M13.00 | Ankle pain |
| 25C..13 | O/E - lumbar pain on palpation |
| N094111 | Shoulder joint pain |
| N094211 | Elbow joint pain |
| N094311 | Wrist joint pain |
| N094411 | Hand joint pain |
| N094512 | Hip joint pain |
| N094611 | Knee joint pain |
| N094711 | Ankle joint pain |
| N094D11 | Elbow joint pain |
| N094F11 | Wrist pain |
| N094K12 | Hip pain |
| N094W00 | Anterior knee pain |
| N096.12 | Musculoskeletal pain - joints |
| N12..13 | Acute back pain - disc |
| N131.11 | Pain in cervical spine |
| N141.00 | Pain in thoracic spine |
| N141.11 | Acute back pain - thoracic |
| N142.00 | Pain in lumbar spine |
| N142.11 | Low back pain |
| N142.13 | Acute back pain - lumbar |
| N143.11 | Acute back pain with sciatica |
| N145.11 | Acute back pain - unspecified |
| N145.12 | Back pain, unspecified |
| N147211 | Pain in coccyx |
| N245700 | Shoulder pain |
| N33A.00 | Bone pain |
| N33A000 | Bony pelvic pain |
| N33A100 | Clavicle pain |
| R065011 | [D] Retrosternal chest pain |
| R065900 | [D]Parasternal chest pain |
| R065A00 | [D]Musculoskeletal chest pain |
| R065C00 | [D]Retrosternal chest pain |
| SP07A00 | Pain due to hip joint prosthesis |
| SP07B00 | Pain due to knee joint prosthesis |
| SP07C00 | Pain due to shoulder joint prosthesis |
| SyuKE11 | [X] Pain due to internal orthopaedic prosthesis |
| **Bone pain - back** | |
| 16C5.00 | C/O - low back pain |
| 16C6.00 | Back pain without radiation NOS |
| 16C9.00 | Chronic low back pain |
| 16CA.00 | Mechanical low back pain |
| 1A53.12 | C/O - lumbar pain |
| 25C..13 | O/E - lumbar pain on palpation |
| N12..13 | Acute back pain - disc |
| N131.11 | Pain in cervical spine |
| N141.00 | Pain in thoracic spine |
| N141.11 | Acute back pain - thoracic |
| N142.00 | Pain in lumbar spine |
| N142.11 | Low back pain |
| N142.13 | Acute back pain - lumbar |
| N143.11 | Acute back pain with sciatica |
| N145.11 | Acute back pain - unspecified |
| N145.12 | Back pain, unspecified |
| N147211 | Pain in coccyx |
| **Bone pain - chest** | |
| 182B000 | Costal margin chest pain |
| R065011 | [D] Retrosternal chest pain |
| R065900 | [D]Parasternal chest pain |
| R065A00 | [D]Musculoskeletal chest pain |
| R065C00 | [D]Retrosternal chest pain |
| **Bone pain - joint** | |
| 1M00.00 | Pain in elbow |
| 1M00.11 | Elbow pain |
| 1M01.00 | Pain in wrist |
| 1M10.00 | Knee pain |
| 1M12.00 | Anterior knee pain |
| 1M13.00 | Ankle pain |
| N094111 | Shoulder joint pain |
| N094211 | Elbow joint pain |
| N094311 | Wrist joint pain |
| N094411 | Hand joint pain |
| N094512 | Hip joint pain |
| N094611 | Knee joint pain |
| N094711 | Ankle joint pain |
| N094D11 | Elbow joint pain |
| N094F11 | Wrist pain |
| N094K12 | Hip pain |
| N094W00 | Anterior knee pain |
| N096.12 | Musculoskeletal pain - joints |
| N245700 | Shoulder pain |
| N33A000 | Bony pelvic pain |
| N33A100 | Clavicle pain |
| SP07A00 | Pain due to hip joint prosthesis |
| SP07B00 | Pain due to knee joint prosthesis |
| SP07C00 | Pain due to shoulder joint prosthesis |
| SyuKE11 | [X] Pain due to internal orthopaedic prosthesis |
| **Bone pain - rib** | |
| 1973.00 | Left subcostal pain |
| 1974.00 | Right subcostal pain |
| 182B.00 | Rib pain |
| 197..14 | Subcostal pain |

ANNEX 5. Definition of skeletal-related events (SRE)

| **READ code** | **READ term** |
| --- | --- |
| **SRE: Pathological fracture – non-vertebral (excluding skull, face, fingers, toes)** | |
| S234100 | Closed Colles' fracture |
| S200300 | Closed fracture clavicle, lateral end |
| S200100 | Closed fracture clavicle, medial end |
| S200200 | Closed fracture clavicle, shaft |
| S312.00 | Closed fracture distal femur |
| S312600 | Closed fracture distal femur, bicondylar (T-Y fracture) |
| S312x00 | Closed fracture distal femur, comminuted/intra-articular |
| S312500 | Closed fracture distal femur, lateral condyle |
| S312400 | Closed fracture distal femur, medial condyle |
| S312300 | Closed fracture distal femur, supracondylar |
| S224900 | Closed fracture distal humerus, bicondylar (T-Y fracture) |
| S224800 | Closed fracture distal humerus, capitellum |
| S224200 | Closed fracture distal humerus, lateral condyle |
| S224600 | Closed fracture distal humerus, lateral epicondyle |
| S224300 | Closed fracture distal humerus, medial condyle |
| S224700 | Closed fracture distal humerus, medial epicondyle |
| S224100 | Closed fracture distal humerus, supracondylar |
| S200.00 | Closed fracture of clavicle |
| S200z00 | Closed fracture of clavicle NOS |
| S200000 | Closed fracture of clavicle, unspecified part |
| S312z00 | Closed fracture of distal femur not otherwise specified |
| S312000 | Closed fracture of distal femur, unspecified |
| S224400 | Closed fracture of distal humerus, condyle(s) unspecified |
| S224x00 | Closed fracture of distal humerus, multiple |
| S224z00 | Closed fracture of distal humerus, not otherwise specified |
| S224500 | Closed fracture of distal humerus, trochlea |
| S33C.00 | Closed fracture of distal tibia and fibula |
| S312.11 | Closed fracture of femur, distal end |
| S302011 | Closed fracture of femur, greater trochanter |
| S302400 | Closed fracture of femur, intertrochanteric |
| S302012 | Closed fracture of femur, lesser trochanter |
| S312200 | Closed fracture of femur, lower epiphysis |
| S310.00 | Closed fracture of femur, shaft or unspecified part |
| S300y11 | Closed fracture of femur, subcapital |
| S310000 | Closed fracture of femur, unspecified part |
| S300A00 | Closed fracture of femur, upper epiphysis |
| S130.00 | Closed fracture acetabulum |
| S130z00 | Closed fracture acetabulum NOS |
| S130200 | Closed fracture acetabulum, anterior column |
| S130000 | Closed fracture acetabulum, anterior lip alone |
| S130600 | Closed fracture acetabulum, double column unspecified |
| S130400 | Closed fracture acetabulum, floor |
| S130300 | Closed fracture acetabulum, posterior column |
| S130100 | Closed fracture acetabulum, posterior lip alone |
| S134800 | Closed fracture dislocation of sacro-iliac joint |
| S334.00 | Closed fracture distal tibia |
| S334000 | Closed fracture distal tibia, extra-articular |
| S334100 | Closed fracture distal tibia, intra-articular |
| S344.00 | Closed fracture ankle, bimalleolar |
| S344100 | Closed fracture ankle, bimalleolar, high fibular fracture |
| S344000 | Closed fracture ankle, bimalleolar, low fibular fracture |
| S342.00 | Closed fracture ankle, lateral malleolus |
| S342100 | Closed fracture ankle, lateral malleolus, high |
| S342000 | Closed fracture ankle, lateral malleolus, low |
| S340.00 | Closed fracture ankle, medial malleolus |
| S346.00 | Closed fracture ankle, trimalleolar |
| S346100 | Closed fracture ankle, trimalleolar, high fibular fracture |
| S346000 | Closed fracture ankle, trimalleolar, low fibular fracture |
| S34x.00 | Closed fracture ankle, unspecified |
| S330800 | Closed fracture fibula, head |
| S330900 | Closed fracture fibula, neck |
| S300400 | Closed fracture head of femur |
| S339000 | Closed fracture of distal fibula |
| S33x100 | Closed fracture of fibula, unspecified part, NOS |
| S222000 | Closed fracture of humerus NOS |
| S222100 | Closed fracture of humerus, shaft |
| S222.00 | Closed fracture of humerus, shaft or unspecified part |
| S222z00 | Closed fracture of humerus, shaft or unspecified part NOS |
| S220500 | Closed fracture of humerus, upper epiphysis |
| S30y.00 | Closed fracture of neck of femur NOS |
| S23x100 | Closed fracture of radius (alone), unspecified |
| S234.00 | Closed fracture of radius and ulna, lower end |
| S23xz00 | Closed fracture of radius and ulna, NOS |
| S232.00 | Closed fracture of radius and ulna, shaft |
| S232z00 | Closed fracture of radius and ulna, shaft, NOS |
| S23x.00 | Closed fracture of radius and ulna, unspecified part |
| S232000 | Closed fracture of radius, shaft, unspecified |
| S234600 | Closed fracture radius and ulna, distal |
| S310100 | Closed fracture shaft of femur |
| S332100 | Closed fracture shaft of fibula |
| S332000 | Closed fracture shaft of tibia |
| S4E0.00 | Closed fracture-dislocation, hip joint |
| S4C2.00 | Closed fracture-subluxation of the wrist |
| S4B2100 | Closed fracture-subluxation superior radio-ulnar joint |
| S4G2.00 | Closed fracture-subluxation, ankle joint |
| S4C2000 | Closed fracture-subluxation, distal radio-ulnar jt |
| S4E2.00 | Closed fracture-subluxation, hip joint |
| S300311 | Closed fracture, base of neck of femur |
| S234800 | Closed Galeazzi fracture |
| S280.00 | Closed ill-defined fractures of upper limb |
| S230300 | Closed Monteggia's fracture |
| S292000 | Closed multiple fractures of clavicle, scapula and humerus |
| S234700 | Closed Smith's fracture |
| S3x0.00 | Other, multiple and ill-defined closed fractures lower limb |
| S224.00 | Closed fracture of the distal humerus |
| S234200 | Closed fracture of the distal radius, unspecified |
| S330000 | Closed fracture of the proximal tibia |
| S230500 | Closed fracture of the proximal ulna |
| S232100 | Closed fracture of the radial shaft |
| S23x300 | Closed fracture of the radius and ulna |
| S232200 | Closed fracture of the ulnar shaft |
| S330.00 | Closed fracture of tibia and fibula, proximal |
| S330200 | Closed fracture of tibia and fibula, proximal |
| S330z00 | Closed fracture of tibia and fibula, proximal NOS |
| S332200 | Closed fracture of tibia and fibula, shaft |
| S332z00 | Closed fracture of tibia and fibula, shaft, NOS |
| S33x200 | Closed fracture of tibia and fibula, unspecified part |
| S33x.00 | Closed fracture of tibia and fibula, unspecified part, NOS |
| S33xz00 | Closed fracture of tibia and fibula, unspecified part, NOS |
| S33x000 | Closed fracture of tibia, unspecified part, NOS |
| S332.00 | Closed fracture of tibia/fibula, shaft |
| S330011 | Closed fracture of tibial condyles |
| S330012 | Closed fracture of tibial tuberosity |
| S23x200 | Closed fracture of ulna (alone), unspecified |
| S230200 | Closed fracture of ulna, coronoid |
| S234400 | Closed fracture of ulna, lower epiphysis |
| S234300 | Closed fracture of ulna, styloid process |
| S30w.00 | Closed fracture of unspecified proximal femur |
| S330100 | Closed fracture proximal fibula |
| S220700 | Closed fracture proximal humerus, four part |
| S220300 | Closed fracture proximal humerus, greater tuberosity |
| S220400 | Closed fracture proximal humerus, head |
| S220100 | Closed fracture proximal humerus, neck |
| S220600 | Closed fracture proximal humerus, three part |
| S330500 | Closed fracture proximal tibia, bicondylar |
| S330400 | Closed fracture proximal tibia, lateral condyle (plateau) |
| S330300 | Closed fracture proximal tibia, medial condyle (plateau) |
| S132.00 | Closed fracture pubis |
| S132z00 | Closed fracture pubis NOS |
| S230600 | Closed fracture radius, head |
| S230700 | Closed fracture radius, neck |
| S330600 | Closed fracture spine, tibia |
| S330700 | Closed fracture tubercle, tibia |
| S4B0.00 | Closed fracture-dislocation elbow |
| S4B0000 | Closed fracture-dislocation elbow joint |
| S4J0100 | Closed fracture-dislocation of pelvis |
| S4G0.00 | Closed fracture-dislocation, ankle joint |
| S4J2100 | Closed fracture-subluxation of pelvis |
| S234912 | Closed volar Barton fracture-subluxation |
| S234900 | Closed volar Barton's fracture |
| S234911 | Closed volar Barton's fracture-dislocation |
| S234211 | Dupuytren's fracture, radius - closed |
| S134.00 | Other or multiple closed fracture of pelvis |
| S134z00 | Other or multiple closed fracture of pelvis NOS |
| S130y00 | Other specified closed fracture acetabulum |
| S234111 | Smith's fracture - closed |
| S234.11 | Wrist fracture - closed |
| S234F00 | Closed Barton's fracture |
| S234A12 | Closed dorsal Barton fracture-subluxation |
| S234A11 | Closed dorsal Barton's fracture-dislocation |
| S234D00 | Closed fracture distal radius, extra-articular, other type |
| S234C00 | Closed fracture distal radius, intra-articular, die-punch |
| S234E00 | Closed fracture distal radius, intra-articular, other type |
| S234500 | Closed fracture distal ulna, unspecified |
| S13y.00 | Closed fracture of pelvis NOS |
| S302.00 | Closed fracture of proximal femur, pertrochanteric |
| S230z00 | Closed fracture of proximal forearm not otherwise specified |
| S230000 | Closed fracture of proximal forearm, unspecified part |
| S220z00 | Closed fracture of proximal humerus not otherwise specified |
| S220200 | Closed fracture of proximal humerus, anatomical neck |
| S220000 | Closed fracture of proximal humerus, unspecified part |
| S230.00 | Closed fracture of proximal radius and ulna |
| S230400 | Closed fracture of proximal ulna, comminuted |
| S220.00 | Closed fracture of the proximal humerus |
| S134500 | Closed fracture pelvis, anterior inferior iliac spine |
| S134400 | Closed fracture pelvis, anterior superior iliac spine |
| S134600 | Closed fracture pelvis, iliac wing |
| S134300 | Closed fracture pelvis, ischial tuberosity |
| S134100 | Closed fracture pelvis, ischium |
| S132100 | Closed fracture pelvis, multiple pubic rami - stable |
| S132200 | Closed fracture pelvis, multiple pubic rami - unstable |
| S132000 | Closed fracture pelvis, single pubic ramus |
| S300300 | Closed fracture proximal femur, basicervical |
| S302100 | Closed fracture proximal femur, intertrochanteric, two part |
| S300200 | Closed fracture proximal femur, midcervical section |
| S300y00 | Closed fracture proximal femur, other transcervical |
| S300600 | Closed fracture proximal femur, subcapital, Garden grade I |
| S300700 | Closed fracture proximal femur, subcapital, Garden grade II |
| S300800 | Closed fracture proximal femur, subcapital, Garden grade III |
| S300900 | Closed fracture proximal femur, subcapital, Garden grade IV |
| S302200 | Closed fracture proximal femur, subtrochanteric |
| S300.00 | Closed fracture proximal femur, transcervical |
| S300z00 | Closed fracture proximal femur, transcervical, NOS |
| S300100 | Closed fracture proximal femur, transepiphyseal |
| S230A00 | Closed fracture radius and ulna, proximal |
| S4F0.00 | Closed fracture-dislocation, knee joint |
| SR12000 | Closed fractures involving multiple regions of one upp limb |
| S4F2.00 | Closed fracture-subluxation, knee joint |
| S350000 | Closed fracture calcaneus, extra-articular |
| S350100 | Closed fracture calcaneus, intra-articular |
| S240F00 | Closed fracture carpal bones, multiple |
| S4C0.00 | Closed fracture dislocation of wrist |
| **SRE: Pathological fractures - vertebral** | |
| N331A00 | Osteoporosis + pathological fracture cervical vertebrae |
| N331800 | Osteoporosis + pathological fracture lumbar vertebrae |
| N331900 | Osteoporosis + pathological fracture thoracic vertebrae |
| N331C00 | Pathological fracture of cervical vertebra |
| N331100 | Pathological fracture of lumbar vertebra |
| N331000 | Pathological fracture of thoracic vertebra |
| S100111 | C1 vertebra closed fracture - no spinal cord lesion |
| S100211 | C2 vertebra closed fracture without spinal cord lesion |
| S100311 | C3 vertebra closed fracture without spinal cord lesion |
| S100411 | C4 vertebra closed fracture without spinal cord lesion |
| S100511 | C5 vertebra closed fracture without spinal cord lesion |
| S100611 | C6 vertebra closed fracture without spinal cord lesion |
| S100711 | C7 vertebra closed fracture without spinal cord lesion |
| S100G00 | Closed fracture cervical vertebra, burst |
| S100M00 | Closed fracture cervical vertebra, posterior arch |
| S100K00 | Closed fracture cervical vertebra, spinous process |
| S100J00 | Closed fracture cervical vertebra, spondylolysis |
| S100L00 | Closed fracture cervical vertebra, transverse process |
| S100H00 | Closed fracture cervical vertebra, wedge |
| S104.00 | Closed fracture lumbar vertebra |
| S104000 | Closed fracture lumbar vertebra, burst |
| S104500 | Closed fracture lumbar vertebra, posterior arch |
| S104300 | Closed fracture lumbar vertebra, spinous process |
| S104200 | Closed fracture lumbar vertebra, spondylolysis |
| S104400 | Closed fracture lumbar vertebra, transverse process |
| S104600 | Closed fracture lumbar vertebra, tricolumnar |
| S104100 | Closed fracture lumbar vertebra, wedge |
| S100.00 | Closed fracture of cervical spine |
| S100z00 | Closed fracture of cervical spine not otherwise specified |
| S110.00 | Closed fracture of cervical spine with cord lesion |
| S110z00 | Closed fracture of cervical spine with cord lesion NOS |
| S118.00 | Closed fracture of coccyx with spinal cord lesion |
| S118z00 | Closed fracture of coccyx with spinal cord lesion NOS |
| S100100 | Closed fracture atlas |
| S100900 | Closed fracture atlas, comminuted |
| S100800 | Closed fracture atlas, isolated arch or articular process |
| S100200 | Closed fracture axis |
| S100A00 | Closed fracture axis, odontoid process |
| S100E00 | Closed fracture axis, posterior arch |
| S100C00 | Closed fracture axis, spinous process |
| S100B00 | Closed fracture axis, spondylolysis |
| S100D00 | Closed fracture axis, transverse process |
| S106000 | Closed compression fracture sacrum |
| S116.00 | Closed fracture of sacrum with spinal cord lesion |
| S116z00 | Closed fracture of sacrum with spinal cord lesion NOS |
| S106.00 | Closed fracture sacrum |
| S102.00 | Closed fracture thoracic vertebra |
| S102z00 | Closed fracture thoracic vertebra not otherwise specified |
| S102000 | Closed fracture thoracic vertebra, burst |
| S102500 | Closed fracture thoracic vertebra, posterior arch |
| S102300 | Closed fracture thoracic vertebra, spinous process |
| S102200 | Closed fracture thoracic vertebra, spondylolysis |
| S102400 | Closed fracture thoracic vertebra, transverse process |
| S102100 | Closed fracture thoracic vertebra, wedge |
| S150000 | Closed multiple fractures of thoracic spine |
| S114500 | Closed spinal fracture with cauda equina lesion |
| S114100 | Closed spinal fracture with complete lumbar cord lesion |
| S114000 | Closed spinal fracture with unspecified lumbar cord lesion |
| S106100 | Closed vertical fracture of sacrum |
| S100x00 | Multiple closed fractures of cervical vertebrae |
| S102y00 | Other specified closed fracture thoracic vertebra |
| S114.00 | Closed fracture of lumbar spine with spinal cord lesion |
| S11x.00 | Closed fracture of spine with spinal cord lesion unspecified |
| S10x.00 | Closed fracture of spine, unspecified, |
| S100000 | Closed fracture of unspecified cervical vertebra |
| S100300 | Closed fracture of third cervical vertebra |
| S112z00 | Closed fracture of thoracic spine with cord lesion NOS |
| S112.00 | Closed fracture of thoracic spine with spinal cord lesion |
| S108.00 | Closed fracture pelvis, coccyx |
| **SRE: Pathological fractures – non-vertebral (skull, face, fingers, toes)** | |
| S260R00 | Closed fracture finger distal phalanx |
| S260S00 | Closed fracture finger distal phalanx, base |
| S260V00 | Closed fracture finger distal phalanx, mallet |
| S260W00 | Closed fracture finger distal phalanx, multiple |
| S260T00 | Closed fracture finger distal phalanx, shaft |
| S260U00 | Closed fracture finger distal phalanx, tuft |
| S250600 | Closed fracture finger metacarpal |
| S250200 | Closed fracture finger metacarpal base |
| S250500 | Closed fracture finger metacarpal head |
| S250400 | Closed fracture finger metacarpal neck |
| S250300 | Closed fracture finger metacarpal shaft |
| S250700 | Closed fracture finger metacarpal, multiple |
| S260K00 | Closed fracture finger middle phalanx |
| S260L00 | Closed fracture finger middle phalanx, base |
| S260P00 | Closed fracture finger middle phalanx, head |
| S260Q00 | Closed fracture finger middle phalanx, multiple |
| S260N00 | Closed fracture finger middle phalanx, neck |
| S260M00 | Closed fracture finger middle phalanx, shaft |
| S260D00 | Closed fracture finger proximal phalanx |
| S260E00 | Closed fracture finger proximal phalanx, base |
| S260H00 | Closed fracture finger proximal phalanx, head |
| S260J00 | Closed fracture finger proximal phalanx, multiple |
| S260G00 | Closed fracture finger proximal phalanx, neck |
| S260F00 | Closed fracture finger proximal phalanx, shaft |
| S240800 | Closed fracture hamate |
| S240900 | Closed fracture hamate, hook |
| S240200 | Closed fracture lunate |
| S240.00 | Closed fracture of carpal bone |
| S240z00 | Closed fracture of carpal bone NOS |
| S240000 | Closed fracture of carpal bone, unspecified |
| S250800 | Closed fracture of thumb metacarpal |
| S352A00 | Closed fracture talus, body |
| S352800 | Closed fracture talus, head |
| S352900 | Closed fracture talus, neck |
| S260800 | Closed fracture thumb distal phalanx |
| S260900 | Closed fracture thumb distal phalanx, base |
| S260C00 | Closed fracture thumb distal phalanx, mallet |
| S260A00 | Closed fracture thumb distal phalanx, shaft |
| S260B00 | Closed fracture thumb distal phalanx, tuft |
| S250C00 | Closed fracture thumb metacarpal head |
| S250B00 | Closed fracture thumb metacarpal neck |
| S250A00 | Closed fracture thumb metacarpal shaft |
| S260300 | Closed fracture thumb proximal phalanx |
| S260400 | Closed fracture thumb proximal phalanx, base |
| S260700 | Closed fracture thumb proximal phalanx, head |
| S260600 | Closed fracture thumb proximal phalanx, neck |
| S260500 | Closed fracture thumb proximal phalanx, shaft |
| S4D0.00 | Closed fracture-dislocation digit |
| S4D0000 | Closed fracture-dislocation digit, unspecified |
| S4C0000 | Closed fracture-dislocation distal radio-ulnar joint |
| S4C0200 | Closed fracture-dislocation mid carpal |
| S4D0600 | Closed fracture-dislocation multiple digits |
| S4C0100 | Closed fracture-dislocation radiocarpal joint |
| S4D0300 | Closed fracture-dislocation, distal interphalangeal joint |
| S4D0500 | Closed fracture-dislocation, interphalangeal joint thumb |
| S4D2.00 | Closed fracture-subluxation digit |
| S4D2000 | Closed fracture-subluxation digit, unspecified |
| S4C2200 | Closed fracture-subluxation mid carpal |
| S4C2100 | Closed fracture-subluxation radiocarpal joint |
| S4C2300 | Closed fracture-subluxation, carpometacarpal joint |
| S4D2300 | Closed fracture-subluxation, distal interphalangeal joint |
| S4D2500 | Closed fracture-subluxation, interphalangeal joint thumb |
| S4D2400 | Closed fracture-subluxation, proximal interphalangeal joint |
| S250x00 | Closed fractures of multiple sites of unspecified metacarpus |
| S260x00 | Closed fractures of phalanx or phalanges, multiple sites |
| S270.00 | Closed multiple fractures of hand bones |
| S352G00 | Closed tarsal fractures, multiple |
| S260.00 | Closed fracture of one or more phalanges of hand |
| S260z00 | Closed fracture of one or more phalanges of hand NOS |
| S260000 | Closed fracture of phalanx or phalanges, unspecified |
| S030.00 | Closed fracture of skull NOS without intracranial injury |
| S010.00 | Closed fracture base of skull without intracranial injury |
| S000.00 | Closed fracture vault of skull without intracranial injury |
| S030.00 | Closed fracture of skull NOS without intracranial injury |
| **SRE: Pathological fractures – not specified** | |
| NyuB000 | [X]Other osteoporosis with pathological fracture |
| NyuB800 | [X]Unspecified osteoporosis with pathological fracture |
| N331500 | Drug-induced osteoporosis with pathological fracture |
| N331600 | Idiopathic osteoporosis with pathological fracture |
| N331300 | Osteoporosis of disuse with pathological fracture |
| N331y00 | Other specified pathological fracture |
| N331.00 | Pathological fracture |
| B585000 | Pathological fracture due to metastatic bone disease |
| N331z00 | Pathological fracture NOS |
| N331B00 | Postmenopausal osteoporosis with pathological fracture |
| N331200 | Postoophorectomy osteoporosis with pathological fracture |
| N331M00 | Fragility fracture due to unspecified osteoporosis |
| 12I6.00 | FH: Fragility fracture |
| 14G6.00 | H/O: fragility fracture |
| N331N00 | Fragility fracture |
| SR1z000 | [X]Closed multiple fractures unspecified |
| S3z0.00 | Closed fracture of bones, unspecified |
| **SRE: Radiation therapy to bone** | |
| 5136.00 | X-ray metastasis control |
| 7L1Z300 | Delivery of a fraction of external beam radiotherapy NEC |
| 7L1hz00 | Preparation for external beam radiotherapy NOS |
| ZV14H00 | [V]Personal history of strontium ranelate allergy |
| 14LW.00 | H/O: strontium ranelate allergy |
| 5AC..00 | Strontium 89 therapy |
| 8I2Y.00 | Strontium ranelate contraindicated |
| 8I3h.00 | Strontium ranelate declined |
| 8I6V.00 | Strontium ranelate not indicated |
| 8I7H.00 | Strontium ranelate not tolerated |
| 59...00 | External radiotherapy |
| 59Z..00 | External radiotherapy NOS |
| 5A8..00 | Other radiotherapy misc. |
| 5A8Z.00 | Other radiotherapy NOS |
| 7L1Zy00 | Other specified radiotherapy delivery |
| 7M0cy00 | Other specified radiotherapy procedures |
| 5149.00 | Radiotherapy-tumour palliation |
| 5137.00 | X-ray radiotherapy control |
| 863..12 | Radiation therapy |
| 7K1V700 | Percutaneous radiofrequency ablation of lesion of bone |
| 5941.00 | Proton therapy |
| 7K13400 | Cryoablation of bone lesion |
| 7048300 | Radiofrequency controlled thermal destruction spinal n root |
| 7049000 | Radiofrequency control thermal destruct spinothalamic tract |
| 7051400 | Radiofrequency controlled thermal destruct peripheral nerve |
| 7060300 | Radiofrequency controlled thermal destruct cerv sympath n |
| 7061400 | Radiofrequency controlled thermal destruct lumbar sympath n |
| 7062700 | Radiofrequency control thermal destruct thoracic sympath nrv |
| 7063w00 | Radiofrequency controlled thermal destruction sympathet n OS |
| 7063x00 | Radiofrequency controlled thermal destruction sympath n NOS |
| 5914.11 | Deep X-ray therapy |
| 5914.00 | Deep X-ray therapy 150-400 Kv |
| 591..00 | X-ray beam therapy |
| 591Z.00 | X-ray beam therapy NOS |
| 59...11 | X-ray therapy -external |
| 5912.00 | Superfic.X-ray therapy 10-60Kv |
| **SRE: Spinal cord compression** | |
| F29y411 | Spinal cord compression |
| F16z.12 | Spinal cord compression NOS |
| S106000 | Closed compression fracture sacrum |
| N12C300 | Lumbar disc prolapse with cauda equina compression |
| F29y411 | Spinal cord compression |
| F16z.12 | Spinal cord compression NOS |
| 7N05000 | [SO]Cervical spinal cord |
| 7N05100 | [SO]Dorsal spinal cord |
| 7N05200 | [SO]Lumbar spinal cord |
| 7N05y00 | [SO]Specified spinal cord NEC |
| 7N05.00 | [SO]Spinal cord |
| 7N05z00 | [SO]Spinal cord NEC |
| 7N05111 | [SO]Thoracic spinal cord |
| S118.00 | Closed fracture of coccyx with spinal cord lesion |
| S118z00 | Closed fracture of coccyx with spinal cord lesion NOS |
| S114.00 | Closed fracture of lumbar spine with spinal cord lesion |
| S116.00 | Closed fracture of sacrum with spinal cord lesion |
| S116z00 | Closed fracture of sacrum with spinal cord lesion NOS |
| S11x.00 | Closed fracture of spine with spinal cord lesion unspecified |
| S112.00 | Closed fracture of thoracic spine with spinal cord lesion |
| S114100 | Closed spinal fracture with complete lumbar cord lesion |
| S114000 | Closed spinal fracture with unspecified lumbar cord lesion |
| S11..00 | Fracture of spine with spinal cord lesion |
| S11z.00 | Fracture of spine with spinal cord lesion NOS |
| S11..11 | Fracture of transverse process of spine + spinal cord lesion |
| S11..12 | Fracture of vertebra with spinal cord lesion |
| B583100 | Secondary malignant neoplasm of spinal cord |
| F29y411 | Spinal cord compression |
| F16z.12 | Spinal cord compression NOS |
| N331H00 | Collapse of cervical vertebra due to osteoporosis |
| N331J00 | Collapse of lumbar vertebra due to osteoporosis |
| N331K00 | Collapse of thoracic vertebra due to osteoporosis |
| N331L00 | Collapse of vertebra due to osteoporosis NOS |
| **SRE: Surgery to bone** | |
| 14N3000 | H/O Spinal surgery |
| 14N3.00 | H/O: major orthopaedic surgery |
| N12A.11 | Post spinal surgery syndrome |
| 7K1K.00 | Closed (or no) reduction of fracture and external fixation |
| 7K1J.00 | Closed (or no) reduction of fracture and internal fixation |
| 7K1K000 | Closed reduction fracture bone and fixation to skeleton HFQ |
| 7K6GN00 | Closed reduction fracture disloc joint & internal fixation |
| 7K1J300 | Closed reduction fracture small bone & fixation using screw |
| 7K1Kz00 | Closed reduction of bone fracture and external fixation NOS |
| 7K1Ky00 | Closed reduction of bone fracture and external fixation OS |
| 7K1Jz00 | Closed reduction of bone fracture and internal fixation NOS |
| 7K1Jy00 | Closed reduction of bone fracture and internal fixation OS |
| 82...11 | Closed reduction of fracture |
| 7K1L800 | Closed reduction of fracture of ankle |
| 7K1LE00 | Closed reduction of fracture of elbow |
| 7K1L500 | Closed reduction of fracture of femur |
| 7K1LH00 | Closed reduction of fracture of finger |
| 7K1LB00 | Closed reduction of fracture of hallux |
| 7K1L400 | Closed reduction of fracture of hip |
| 7K1LF00 | Closed reduction of fracture of humerus |
| 7K1L600 | Closed reduction of fracture of knee |
| 7K1LC00 | Closed reduction of fracture of lower limb |
| 7K1LK00 | Closed reduction of fracture of metacarpus |
| 7K1L900 | Closed reduction of fracture of metatarsus |
| 7K1LD00 | Closed reduction of fracture of nasal bone |
| 7K1LL00 | Closed reduction of fracture of radius and or ulna |
| 7K1LG00 | Closed reduction of fracture of shoulder |
| 7K1LJ00 | Closed reduction of fracture of thumb |
| 7K1L700 | Closed reduction of fracture of tibia and or fibula |
| 7K1LA00 | Closed reduction of fracture of toe |
| 7K1LN00 | Closed reduction of fracture of upper limb |
| 7K1LM00 | Closed reduction of fracture of wrist |
| 7J42200 | Manipulative reduction of fracture of spine |
| 7J42000 | Open reduction of fracture of spine & excis facet of spine |
| 7J42100 | Open reduction of fracture of spine NEC |
| 7K1L.00 | Other closed reduction of fracture of bone |
| 7K1Lz00 | Other closed reduction of fracture of bone NOS |
| 7K1G.00 | Other primary open reduction of fracture of bone |
| 7K1Gz00 | Other primary open reduction of fracture of bone NOS |
| 7J42.00 | Other reduction of fracture of spine |
| 7J42.11 | Other reduction of fracture of spine and stabilisation |
| 7J42z00 | Other reduction of fracture of spine NOS |
| 7K1Ly00 | Other specified other closed reduction of fracture of bone |
| 7J42y00 | Other specified other reduction of fracture of spine |
| 7K1Hy00 | Other specified secondary open reduction of fracture of bone |
| 7K1F400 | Prim extraarticular reduction intraartic fracture bone NEC |
| 7K1Ez00 | Prim open reduction fracture bone & extramedull fixation NOS |
| 7K1Dz00 | Prim open reduction fracture bone & intramedull fixation NOS |
| 7K1Dy00 | Prim open reduction fracture bone & intramedullary fixatn OS |
| 7K1Le00 | Primary arthroscopic reduction and fixation of fracture |
| 7K1Ld00 | Primary arthroscopic reduction of fracture |
| 7K1LV00 | Primary closed reduction of fracture alone |
| 7K1LT00 | Primary closed reduction of fracture and cast immobilisation |
| 7K1KE00 | Primary closed reduction of fracture and external fixation |
| 7K1LW00 | Primary closed reduction of fracture and skin traction |
| 7K1JK00 | Primary closed reduction of fracture and wire fixation |
| 7K6GX00 | Primary closed reduction of fracture dislocation alone |
| 7J42L00 | Primary cls reduction spinal fracture+bedrest stabilisation |
| 7K1D.00 | Primary open reduction fracture bone & intramedull fixation |
| 7K1F500 | Primary open reduction fracture patella fixat tension band |
| 7K1Gy11 | Primary open reduction of bone fracture & external fixation |
| 7K1G300 | Primary open reduction of fracture alone |
| 7K1G400 | Primary open reduction of fracture and cast immobilisation |
| 7K1G500 | Primary open reduction of fracture and functional bracing |
| 7K1G600 | Primary open reduction of fracture and skin traction |
| 7K6FE00 | Primary open reduction of fracture dislocation alone |
| 7K6F200 | Primary open reduction of fracture dislocation of joint NEC |
| 7K1Fz00 | Primary open reduction of intraarticular fracture bone NOS |
| 7K1Fy00 | Primary open reduction of intraarticular fracture bone OS |
| 7K1F.00 | Primary open reduction of intraarticular fracture of bone |
| 7K1G200 | Primary open reduction+external fixation of femoral fracture |
| 7K1G100 | Prmy open reduction of fracture and external fixation |
| 7K1G000 | Prmy open reduction of fracture and skeletal traction |
| 7K1Lg00 | Revision to arthroscopic reduction and fixation of fracture |
| 7K1Lf00 | Revision to arthroscopic reduction of fracture |
| 7K1LX00 | Revision to closed reduction of fracture alone |
| 7K1JL00 | Revision to closed reduction of fracture and wire fixation |
| 7K6H400 | Revision to closed reduction of fracture dislocation alone |
| 7K6HX00 | Revision to open reduction fracture dislocation alone |
| 7K1H900 | Revision to open reduction of fracture alone |
| 7K1HE00 | Revision to open reduction of fracture and external fixation |
| 7K1HD00 | Revision to open reduction of fracture and skeletal traction |
| 7K1H.11 | Revision to open reduction of fracture of bone |
| 7K1Y.00 | Second closed reduction fracture bone and internal fixation |
| 7K6H700 | Secondary open reduction fracture disloc joint & fixation |
| 7K6H200 | Secondary open reduction fracture dislocation of joint NEC |
| 7K1H.00 | Secondary open reduction of fracture of bone |
| 7K1Hz00 | Secondary open reduction of fracture of bone NOS |
| 7K1H200 | Secondary open reduction of intraarticular fracture of bone |
| 7204900 | Reconstruction of cavity of orbit with bone graft |
| 7400900 | Total reconstruction of nose with bone graft and skin flap |
| 7400A00 | Total reconstruction of nose with bone graft and free flap |
| 7411D00 | Reconstruction of maxilla with bone graft |
| 7414B00 | Reconstruction of defect of nasal sinus with bone graft |
| 7513100 | Augmentation of alveolar ridge using auto bone graft |
| 7J48E00 | Graft of bone to spine NEC |
| 7K00200 | Thumb reconstruction using bone graft and skin flap |
| 7K12200 | Excision of bone and bone graft however further qualified |
| 7K13100 | Curettage of lesion of bone and graft HFQ |
| 7K13500 | Curettage of tumour of bone and graft HFQ |
| 7K1Q.00 | Graft of bone marrow |
| 7K1Qy00 | Other specified graft of bone marrow |
| 7K1Qz00 | Graft of bone marrow NOS |
| 7K1S.00 | Other graft of bone |
| 7K1S000 | Prepared graft of bone |
| 7K1S011 | Keil prepared graft of bone |
| 7K1S300 | Synthetic graft of bone |
| 7K1Sy00 | Other specified graft of bone |
| 7K1Sz00 | Other graft of bone NOS |
| 7K6A.00 | Fusion of other joint and extraarticular bone graft |
| 7K6A.11 | Arthrodesis and extraarticular bone graft |
| 7K6Ay00 | Fusion of joint and extraarticular bone graft OS |
| 7K6Az00 | Fusion of joint and extraarticular bone graft NOS |
| 7K6B.00 | Fusion of other joint and other articular bone graft |
| 7K6B.11 | Arthrodesis and other articular bone graft |
| 7K6B000 | Primary arthrodesis and articular bone graft NEC |
| 7K6B100 | Revision of arthrodesis and articular bone graft NEC |
| 7K6B200 | Conversion to arthrodesis and articular bone graft NEC |
| 7K6By00 | Other specified fusion of joint and articular bone graft |
| 7K6Bz00 | Fusion of joint and articular bone graft NOS |
| 7K6i.00 | Graft of cord blood to bone marrow |
| 7L0FC00 | Correction of clinodactyly with osteotomy and bone graft |
| 7L0H112 | McFarland bone graft pseudoarthrosis of tibia |
| SP04000 | Mechanical complication of bone graft |
| SP06500 | Infection of bone graft |
| SP0C200 | Bone graft failure |
| 7J43.11 | Internal fixation of fracture of spine |
| 7K14000 | Open osteoclasis, angular correction & internal fixation HFQ |
| 7K14300 | Open osteoclasis and internal fixation NEC |
| 7K16100 | Angulation periarticular osteotomy and internal fixation NEC |
| 7K18000 | Angulation diaphyseal osteotomy and internal fixation HFQ |
| 7K18300 | Rotation diaphyseal osteotomy and internal fixation HFQ |
| 7K1A000 | Multiple osteotomy and internal fixation HFQ |
| 7K1A300 | Internal fixation of osteotomy NEC |
| 7K1DC00 | Prmy open reduction of #+internal fixation with K-wire |
| 7K1E000 | Prmy open reduction of #+internal fixation with plate NEC |
| 7K1E100 | Prmy open reduction #+internal fixation with cerclage wiring |
| 7K1E800 | Prmy open reduction of #+internal fixation with screw(s) |
| 7K1HV00 | Secondary open reduction # bone and internal fixation HFQ |
| 7K1Ja00 | Revision to closed reduction # + other internal fixation |
| 7K1JR00 | Primary closed reduction #+internal fixation with wire |
| 7K1JS00 | Primary closed reduction #+internal fixation with screw(s) |
| 7K1JT00 | Primary closed reduction #+other internal fixation |
| 7K1JY00 | Revision to closed reduction # + internal fixation with wire |
| 7K1JZ00 | Revision to closed reduction #+internal fixation with screws |
| 7K1P200 | Adjustment to internal fixation device NEC |
| 7K1P400 | Removal of internal fixation device NEC |
| 7K1P600 | Internal fixation of bone NEC |
| 7K1Pw00 | Other specified internal fixation of bone |
| 7K1Px00 | Internal fixation of bone NOS |
| 7K1Y100 | Remanip fracture long bone and rigid internal fixation NEC |
| 7K1Yy00 | OS second closed reduct fracture bone and internal fixation |
| 7K1Yz00 | Second closed reduc fract of bone and internal fixation NOS |
| 7K6C000 | Primary arthrodesis and internal fixation of joint NEC |
| 7K6D000 | Revision of arthrodesis and internal fixation NEC |
| 7K6GK00 | Prim closed reduc fract dislocat joint and internal fixation |
| 7K6GM00 | Closed reduction of dislocation of joint & internal fixation |
| 7K6J000 | Open reduction injury growth plate and internal fixation HFQ |
| 7K6J300 | Closed reduction injury growth plate & internal fixation HFQ |
| SP04500 | Mechanical complication of internal rod fixation device |
| SyuKR00 | [X]Mechanical comp of internal fixation device of other bone |
| ZV54000 | [V]Removal of internal orthopaedic fixation device |
| ZLEQC00 | Discharge from hand surgery service |

ANNEX 6. Definition of baseline comorbidities

| **READ code** | **READ term** |
| --- | --- |
| **Chronic Kidney Disease** | |
| 1Z1..00 | Chronic renal impairment |
| 1Z10.00 | Chronic kidney disease stage 1 |
| 1Z11.00 | Chronic kidney disease stage 2 |
| 1Z12.00 | Chronic kidney disease stage 3 |
| 1Z13.00 | Chronic kidney disease stage 4 |
| 1Z14.00 | Chronic kidney disease stage 5 |
| 1Z15.00 | Chronic kidney disease stage 3A |
| 1Z16.00 | Chronic kidney disease stage 3B |
| 1Z17.00 | Chronic kidney disease stage 1 with proteinuria |
| 1Z18.00 | Chronic kidney disease stage 1 without proteinuria |
| 1Z19.00 | Chronic kidney disease stage 2 with proteinuria |
| 1Z1A.00 | Chronic kidney disease stage 2 without proteinuria |
| 1Z1B.00 | Chronic kidney disease stage 3 with proteinuria |
| 1Z1B.11 | CKD stage 3 with proteinuria |
| 1Z1C.00 | Chronic kidney disease stage 3 without proteinuria |
| 1Z1C.11 | CKD stage 3 without proteinuria |
| 1Z1D.00 | Chronic kidney disease stage 3A with proteinuria |
| 1Z1D.11 | CKD stage 3A with proteinuria |
| 1Z1E.00 | Chronic kidney disease stage 3A without proteinuria |
| 1Z1E.11 | CKD stage 3A without proteinuria |
| 1Z1F.00 | Chronic kidney disease stage 3B with proteinuria |
| 1Z1F.11 | CKD stage 3B with proteinuria |
| 1Z1G.00 | Chronic kidney disease stage 3B without proteinuria |
| 1Z1G.11 | CKD stage 3B without proteinuria |
| 1Z1H.00 | Chronic kidney disease stage 4 with proteinuria |
| 1Z1H.11 | CKD stage 4 with proteinuria |
| 1Z1J.00 | Chronic kidney disease stage 4 without proteinuria |
| 1Z1J.11 | CKD stage 4 without proteinuria |
| 1Z1K.00 | Chronic kidney disease stage 5 with proteinuria |
| 1Z1K.11 | CKD stage 5 with proteinuria |
| 1Z1L.00 | Chronic kidney disease stage 5 without proteinuria |
| 1Z1L.11 | CKD stage 5 without proteinuria |
| 661M200 | Chronic kidney disease self-management plan agreed |
| 7B00.00 | Transplantation of kidney |
| 7B00200 | Transplantation of kidney from cadaver |
| 7B00212 | Cadaveric renal transplant |
| 7B00600 | Xenograft renal transplant |
| 7B00y00 | Other specified transplantation of kidney |
| 7B00z00 | Transplantation of kidney NOS |
| 7B01511 | Excision of rejected transplanted kidney |
| 7B06300 | Exploration of renal transplant |
| 7B0F.00 | Interventions associated with transplantation of kidney |
| 7B0F100 | Pre-transplantation of kidney work-up, recipient |
| 7B0F300 | Post-transplantation of kidney examination, recipient |
| 7B0Fy00 | OS interventions associated with transplantation of kidney |
| 7B0Fz00 | Interventions associated with transplantation of kidney NOS |
| 7B00100 | Transplantation of kidney from live donor |
| 8L50.00 | Renal transplant planned |
| D215000 | Anaemia secondary to chronic renal failure |
| K02..00 | Chronic glomerulonephritis |
| K020.00 | Chronic proliferative glomerulonephritis |
| K021.00 | Chronic membranous glomerulonephritis |
| K022.00 | Chronic membranoproliferative glomerulonephritis |
| K023.00 | Chronic rapidly progressive glomerulonephritis |
| K02y.00 | Other chronic glomerulonephritis |
| K02y000 | Chronic glomerulonephritis + diseases EC |
| K02y200 | Chronic focal glomerulonephritis |
| K02y300 | Chronic diffuse glomerulonephritis |
| K02yz00 | Other chronic glomerulonephritis NOS |
| K02z.00 | Chronic glomerulonephritis NOS |
| K05..00 | Chronic renal failure |
| K05..11 | Chronic uraemia |
| K05..12 | End stage renal failure |
| K05..13 | Chronic kidney disease |
| K050.00 | End stage renal failure |
| K051.00 | Chronic kidney disease stage 1 |
| K052.00 | Chronic kidney disease stage 2 |
| K053.00 | Chronic kidney disease stage 3 |
| K054.00 | Chronic kidney disease stage 4 |
| K055.00 | Chronic kidney disease stage 5 |
| K0A3200 | Chron nephritic syndrom difuse membranous glomerulonephritis |
| K0A3300 | Chron neph syn difus mesangial prolifrtiv glomerulonephritis |
| K0A3500 | Chronic neph syn difus mesangiocapillary glomerulonephritis |
| K0A3700 | Chronic nephritic syn diffuse crescentic glomerulonephritis |
| K0B5.00 | Renal tubulo-interstitial disordrs in transplant rejectn |
| K0D..00 | End-stage renal disease |
| K0E..00 | Acute-on-chronic renal failure |
| Kyu1C00 | [X]Renal tubulo-interstitial disorders/transplant rejection |
| Kyu2100 | [X]Other chronic renal failure |
| SP08300 | Kidney transplant failure and rejection |
| SP08D00 | Acute-on-chronic rejection of renal transplant |
| SP08E00 | Acute rejection of renal transplant - grade I |
| SP08F00 | Acute rejection of renal transplant - grade II |
| SP08G00 | Acute rejection of renal transplant - grade III |
| SP08H00 | Acute rejection of renal transplant |
| SP08J00 | Chronic rejection of renal transplant |
| SP08N00 | Unexplained episode of renal transplant dysfunction |
| SP08P00 | Stenosis of vein of transplanted kidney |
| SP08R00 | Renal transplant rejection |
| SP08T00 | Urological complication of renal transplant |
| SP08V00 | Very mild acute rejection of renal transplant |
| SP08W00 | Vascular complication of renal transplant |
| TB00100 | Kidney transplant with complication, without blame |
| TB00111 | Renal transplant with complication, without blame |
| ZV42000 | [V]Kidney transplanted |
| **Cardiovascular diseases (CVD)** | |
| G30..00 | Acute myocardial infarction |
| G30..11 | Attack - heart |
| G30..12 | Coronary thrombosis |
| G30..13 | Cardiac rupture following myocardial infarction (MI) |
| G30..14 | Heart attack |
| G30..15 | MI - acute myocardial infarction |
| G30..16 | Thrombosis - coronary |
| G30..17 | Silent myocardial infarction |
| G300.00 | Acute anterolateral infarction |
| G301.00 | Other specified anterior myocardial infarction |
| G301000 | Acute anteroapical infarction |
| G301100 | Acute anteroseptal infarction |
| G301z00 | Anterior myocardial infarction NOS |
| G302.00 | Acute inferolateral infarction |
| G303.00 | Acute inferoposterior infarction |
| G304.00 | Posterior myocardial infarction NOS |
| G305.00 | Lateral myocardial infarction NOS |
| G306.00 | True posterior myocardial infarction |
| G307.00 | Acute subendocardial infarction |
| G307000 | Acute non-Q wave infarction |
| G307100 | Acute non-ST segment elevation myocardial infarction |
| G308.00 | Inferior myocardial infarction NOS |
| G309.00 | Acute Q-wave infarct |
| G30B.00 | Acute posterolateral myocardial infarction |
| G30X.00 | Acute transmural myocardial infarction of unspecif site |
| G30X000 | Acute ST segment elevation myocardial infarction |
| G30y.00 | Other acute myocardial infarction |
| G30y000 | Acute atrial infarction |
| G30y100 | Acute papillary muscle infarction |
| G30y200 | Acute septal infarction |
| G30yz00 | Other acute myocardial infarction NOS |
| G30z.00 | Acute myocardial infarction NOS |
| G35..00 | Subsequent myocardial infarction |
| G350.00 | Subsequent myocardial infarction of anterior wall |
| G351.00 | Subsequent myocardial infarction of inferior wall |
| G353.00 | Subsequent myocardial infarction of other sites |
| G35X.00 | Subsequent myocardial infarction of unspecified site |
| G38..00 | Postoperative myocardial infarction |
| G380.00 | Postoperative transmural myocardial infarction anterior wall |
| G381.00 | Postoperative transmural myocardial infarction inferior wall |
| G383.00 | Postoperative transmural myocardial infarction unspec site |
| G384.00 | Postoperative subendocardial myocardial infarction |
| G38z.00 | Postoperative myocardial infarction, unspecified |
| Gyu3400 | [X]Acute transmural myocardial infarction of unspecif site |
| Gyu3600 | [X]Subsequent myocardial infarction of unspecified site |
| G3...00 | Ischaemic heart disease |
| G3...11 | Arteriosclerotic heart disease |
| G3...12 | Atherosclerotic heart disease |
| G3...13 | IHD - Ischaemic heart disease |
| G31..00 | Other acute and subacute ischaemic heart disease |
| G312.00 | Coronary thrombosis not resulting in myocardial infarction |
| G31y.00 | Other acute and subacute ischaemic heart disease |
| G31y000 | Acute coronary insufficiency |
| G31y100 | Microinfarction of heart |
| G31y200 | Subendocardial ischaemia |
| G31y300 | Transient myocardial ischaemia |
| G31yz00 | Other acute and subacute ischaemic heart disease NOS |
| G32..00 | Old myocardial infarction |
| G32..11 | Healed myocardial infarction |
| G32..12 | Personal history of myocardial infarction |
| G34..00 | Other chronic ischaemic heart disease |
| G340.00 | Coronary atherosclerosis |
| G340.11 | Triple vessel disease of the heart |
| G340.12 | Coronary artery disease |
| G340000 | Single coronary vessel disease |
| G340100 | Double coronary vessel disease |
| G342.00 | Atherosclerotic cardiovascular disease |
| G343.00 | Ischaemic cardiomyopathy |
| G344.00 | Silent myocardial ischaemia |
| G34y.00 | Other specified chronic ischaemic heart disease |
| G34y000 | Chronic coronary insufficiency |
| G34y100 | Chronic myocardial ischaemia |
| G34yz00 | Other specified chronic ischaemic heart disease NOS |
| G34z.00 | Other chronic ischaemic heart disease NOS |
| G34z000 | Asymptomatic coronary heart disease |
| G35..00 | Subsequent myocardial infarction |
| G39..00 | Coronary microvascular disease |
| G3y..00 | Other specified ischaemic heart disease |
| G3z..00 | Ischaemic heart disease NOS |
| Gyu3.00 | [X]Ischaemic heart diseases |
| Gyu3200 | [X]Other forms of acute ischaemic heart disease |
| Gyu3300 | [X]Other forms of chronic ischaemic heart disease |
| 792..11 | Coronary artery bypass graft operations |
| 7920.00 | Saphenous vein graft replacement of coronary artery |
| 7920.11 | Saphenous vein graft bypass of coronary artery |
| 7920000 | Saphenous vein graft replacement of one coronary artery |
| 7920100 | Saphenous vein graft replacement of two coronary arteries |
| 7920200 | Saphenous vein graft replacement of three coronary arteries |
| 7920300 | Saphenous vein graft replacement of four+ coronary arteries |
| 7920y00 | Saphenous vein graft replacement of coronary artery OS |
| 7920z00 | Saphenous vein graft replacement coronary artery NOS |
| 7921.00 | Other autograft replacement of coronary artery |
| 7921.11 | Other autograft bypass of coronary artery |
| 7921000 | Autograft replacement of one coronary artery NEC |
| 7921100 | Autograft replacement of two coronary arteries NEC |
| 7921200 | Autograft replacement of three coronary arteries NEC |
| 7921300 | Autograft replacement of four of more coronary arteries NEC |
| 7921y00 | Other autograft replacement of coronary artery OS |
| 7921z00 | Other autograft replacement of coronary artery NOS |
| 7922.00 | Allograft replacement of coronary artery |
| 7922.11 | Allograft bypass of coronary artery |
| 7922000 | Allograft replacement of one coronary artery |
| 7922100 | Allograft replacement of two coronary arteries |
| 7922200 | Allograft replacement of three coronary arteries |
| 7922300 | Allograft replacement of four or more coronary arteries |
| 7922y00 | Other specified allograft replacement of coronary artery |
| 7922z00 | Allograft replacement of coronary artery NOS |
| 7923.00 | Prosthetic replacement of coronary artery |
| 7923.11 | Prosthetic bypass of coronary artery |
| 7923000 | Prosthetic replacement of one coronary artery |
| 7923100 | Prosthetic replacement of two coronary arteries |
| 7923200 | Prosthetic replacement of three coronary arteries |
| 7923300 | Prosthetic replacement of four or more coronary arteries |
| 7923z00 | Prosthetic replacement of coronary artery NOS |
| 7924.00 | Revision of bypass for coronary artery |
| 7924000 | Revision of bypass for one coronary artery |
| 7924100 | Revision of bypass for two coronary arteries |
| 7924200 | Revision of bypass for three coronary arteries |
| 7924300 | Revision of bypass for four or more coronary arteries |
| 7924500 | Revision of implantation of thoracic artery into heart |
| 7924y00 | Other specified revision of bypass for coronary artery |
| 7924z00 | Revision of bypass for coronary artery NOS |
| 7925.00 | Connection of mammary artery to coronary artery |
| 7925.11 | Creation of bypass from mammary artery to coronary artery |
| 7925000 | Double anastomosis of mammary arteries to coronary arteries |
| 7925011 | LIMA sequential anastomosis |
| 7925012 | RIMA sequential anastomosis |
| 7925100 | Double implant of mammary arteries into coronary arteries |
| 7925200 | Single anast mammary art to left ant descend coronary art |
| 7925300 | Single anastomosis of mammary artery to coronary artery NEC |
| 7925311 | LIMA single anastomosis |
| 7925312 | RIMA single anastomosis |
| 7925400 | Single implantation of mammary artery into coronary artery |
| 7925y00 | Connection of mammary artery to coronary artery OS |
| 7925z00 | Connection of mammary artery to coronary artery NOS |
| 7926.00 | Connection of other thoracic artery to coronary artery |
| 7926000 | Double anastom thoracic arteries to coronary arteries NEC |
| 7926200 | Single anastomosis of thoracic artery to coronary artery NEC |
| 7926300 | Single implantation thoracic artery into coronary artery NEC |
| 7926z00 | Connection of other thoracic artery to coronary artery NOS |
| 792C.00 | Other replacement of coronary artery |
| 792C000 | Replacement of coronary arteries using multiple methods |
| 792Cy00 | Other specified replacement of coronary artery |
| 792Cz00 | Replacement of coronary artery NOS |
| 792D.00 | Other bypass of coronary artery |
| 792Dy00 | Other specified other bypass of coronary artery |
| 792Dz00 | Other bypass of coronary artery NOS |
| 792y.00 | Other specified operations on coronary artery |
| 7927500 | Open angioplasty of coronary artery |
| 7928.00 | Transluminal balloon angioplasty of coronary artery |
| 7928.11 | Percutaneous balloon coronary angioplasty |
| 7928000 | Percut transluminal balloon angioplasty one coronary artery |
| 7928100 | Percut translum balloon angioplasty mult coronary arteries |
| 7928200 | Percut translum balloon angioplasty bypass graft coronary a |
| 7928300 | Percut translum cutting balloon angioplasty coronary artery |
| 7928y00 | Transluminal balloon angioplasty of coronary artery OS |
| 7928z00 | Transluminal balloon angioplasty of coronary artery NOS |
| 7929.00 | Other therapeutic transluminal operations on coronary artery |
| 7929000 | Percutaneous transluminal laser coronary angioplasty |
| 7929100 | Percut transluminal coronary thrombolysis with streptokinase |
| 7929111 | Percut translum coronary thrombolytic therapy- streptokinase |
| 7929200 | Percut translum inject therap subst to coronary artery NEC |
| 7929300 | Rotary blade coronary angioplasty |
| 7929400 | Insertion of coronary artery stent |
| 7929500 | Insertion of drug-eluting coronary artery stent |
| 7929600 | Percutaneous transluminal atherectomy of coronary artery |
| 7929y00 | Other therapeutic transluminal op on coronary artery OS |
| 7929z00 | Other therapeutic transluminal op on coronary artery NOS |
| 793G.00 | Perc translumin balloon angioplasty stenting coronary artery |
| 793G000 | Perc translum ball angio insert 1-2 drug elut stents cor art |
| 793G100 | Perc tran ball angio ins 3 or more drug elut stents cor art |
| 793G200 | Perc translum balloon angioplasty insert 1-2 stents cor art |
| 793G300 | Percutaneous cor balloon angiop 3 more stents cor art NEC |
| 793Gy00 | OS perc translumina balloon angioplast stenting coronary art |
| 793Gz00 | Perc translum balloon angioplasty stenting coronary art NOS |
| ZV45L00 | [V]Status following coronary angioplasty NOS |
| 14A5.00 | H/O: angina pectoris |
| 14AJ.00 | H/O: Angina in last year |
| 662K.00 | Angina control |
| 662K000 | Angina control - good |
| 662K100 | Angina control - poor |
| 662K200 | Angina control - improving |
| 662K300 | Angina control - worsening |
| 662Kz00 | Angina control NOS |
| 8B27.00 | Antianginal therapy |
| G33..00 | Angina pectoris |
| G330.00 | Angina decubitus |
| G330000 | Nocturnal angina |
| G330z00 | Angina decubitus NOS |
| G331.11 | Variant angina pectoris |
| G332.00 | Coronary artery spasm |
| G33z.00 | Angina pectoris NOS |
| G33z000 | Status anginosus |
| G33z100 | Stenocardia |
| G33z200 | Syncope anginosa |
| G33z300 | Angina on effort |
| G33z400 | Ischaemic chest pain |
| G33z500 | Post infarct angina |
| G33z600 | New onset angina |
| G33z700 | Stable angina |
| G33zz00 | Angina pectoris NOS |
| Gyu3000 | [X]Other forms of angina pectoris |
| G311.00 | Preinfarction syndrome |
| G311.11 | Crescendo angina |
| G311.12 | Impending infarction |
| G311.13 | Unstable angina |
| G311.14 | Angina at rest |
| G311000 | Myocardial infarction aborted |
| G311011 | MI - myocardial infarction aborted |
| G311100 | Unstable angina |
| G311200 | Angina at rest |
| G311300 | Refractory angina |
| G311400 | Worsening angina |
| G311500 | Acute coronary syndrome |
| G311z00 | Preinfarction syndrome NOS |
| G63y000 | Cerebral infarct due to thrombosis of precerebral arteries |
| G63y100 | Cerebral infarction due to embolism of precerebral arteries |
| G64..00 | Cerebral arterial occlusion |
| G64..11 | CVA - cerebral artery occlusion |
| G64..12 | Infarction - cerebral |
| G64..13 | Stroke due to cerebral arterial occlusion |
| G640.00 | Cerebral thrombosis |
| G640000 | Cerebral infarction due to thrombosis of cerebral arteries |
| G641.00 | Cerebral embolism |
| G641.11 | Cerebral embolus |
| G641000 | Cerebral infarction due to embolism of cerebral arteries |
| G64z.00 | Cerebral infarction NOS |
| G64z.11 | Brainstem infarction NOS |
| G64z.12 | Cerebellar infarction |
| G64z000 | Brainstem infarction |
| G64z100 | Wallenberg syndrome |
| G64z111 | Lateral medullary syndrome |
| G64z200 | Left sided cerebral infarction |
| G64z300 | Right sided cerebral infarction |
| G64z400 | Infarction of basal ganglia |
| G665.00 | Pure motor lacunar syndrome |
| G666.00 | Pure sensory lacunar syndrome |
| G676000 | Cereb infarct due cerebral venous thrombosis, nonpyogenic |
| G6W..00 | Cereb infarct due unsp occlus/stenos precerebr arteries |
| Gyu6300 | [X]Cerebrl infarctn due/unspcf occlusn or sten/cerebrl artrs |
| Gyu6400 | [X]Other cerebral infarction |
| Gyu6500 | [X]Occlusion and stenosis of other precerebral arteries |
| Gyu6600 | [X]Occlusion and stenosis of other cerebral arteries |
| Gyu6G00 | [X]Cereb infarct due unsp occlus/stenos precerebr arteries |
| G631.00 | Carotid artery occlusion |
| G631.11 | Stenosis, carotid artery |
| G631.12 | Thrombosis, carotid artery |
| G634.00 | Carotid artery stenosis |
| G70y000 | Carotid artery atherosclerosis |
| G70y011 | Carotid artery disease |
| Fyu5500 | [X]Other transnt cerebral ischaemic attacks+related syndroms |
| G65..00 | Transient cerebral ischaemia |
| G65..11 | Drop attack |
| G65..12 | Transient ischaemic attack |
| G65..13 | Vertebro-basilar insufficiency |
| G650.00 | Basilar artery syndrome |
| G650.11 | Insufficiency - basilar artery |
| G651.00 | Vertebral artery syndrome |
| G651000 | Vertebro-basilar artery syndrome |
| G652.00 | Subclavian steal syndrome |
| G653.00 | Carotid artery syndrome hemispheric |
| G654.00 | Multiple and bilateral precerebral artery syndromes |
| G656.00 | Vertebrobasilar insufficiency |
| G657.00 | Carotid territory transient ischaemic attack |
| G65y.00 | Other transient cerebral ischaemia |
| G65z.00 | Transient cerebral ischaemia NOS |
| G65z000 | Impending cerebral ischaemia |
| G65z100 | Intermittent cerebral ischaemia |
| G65zz00 | Transient cerebral ischaemia NOS |
| ZV12D00 | [V]Personal history of transient ischaemic attack |
| G73..00 | Other peripheral vascular disease |
| G73..11 | Peripheral ischaemic vascular disease |
| G73..12 | Ischaemia of legs |
| G73..13 | Peripheral ischaemia |
| G734.00 | Peripheral arterial disease |
| G73y.00 | Other specified peripheral vascular disease |
| G73z.00 | Peripheral vascular disease NOS |
| G73z000 | Intermittent claudication |
| G73z011 | Claudication |
| G73z012 | Vascular claudication |
| G73zz00 | Peripheral vascular disease NOS |
| Gyu7400 | [X]Other specified peripheral vascular diseases |
| 7A14400 | Replace aneurysm abdominal aorta by anast aorta to aorta NEC |
| 7A14411 | Tube graft of Abdominal aortic aneurysm |
| 7A34K00 | Operation on aneurysm visceral branch of abdominal aorta NEC |
| G713.00 | Abdominal aortic aneurysm which has ruptured |
| G713.11 | Ruptured abdominal aortic aneurysm |
| G714.00 | Abdominal aortic aneurysm without mention of rupture |
| G714.11 | AAA - Abdominal aortic aneurysm without mention of rupture |
| G714000 | Juxtarenal aortic aneurysm |
| G714100 | Inflammatory abdominal aortic aneurysm |
| G714200 | Infrarenal abdominal aortic aneurysm |
| G714300 | Aneurysm of suprarenal aorta |
| G715000 | Thoracoabdominal aortic aneurysm, ruptured |
| G716000 | Thoracoabdominal aortic aneurysm, without mention of rupture |
| G718.00 | Leaking abdominal aortic aneurysm |
| **Gout** | |
| 1443.00 | H/O: gout |
| 6693.00 | Joints gout affected |
| 6695.00 | Date gout treatment started |
| 6696.00 | Date of last gout attack |
| 6697.00 | Gout associated problems |
| 6698.00 | Gout drug side effects |
| 6699.00 | Gout treatment changed |
| 669A.00 | Date gout treatment stopped |
| C34..00 | Gout |
| C340.00 | Gouty arthropathy |
| C341.00 | Gouty nephropathy |
| C341z00 | Gouty nephropathy NOS |
| C342.00 | Idiopathic gout |
| C344.00 | Drug-induced gout |
| C345.00 | Gout due to impairment of renal function |
| C34y.00 | Other specified gouty manifestation |
| C34y000 | Gouty tophi of ear |
| C34y100 | Gouty tophi of heart |
| C34y200 | Gouty tophi of other sites |
| C34y300 | Gouty iritis |
| C34y400 | Gouty neuritis |
| C34y500 | Gouty tophi of hand |
| C34yz00 | Other specified gouty manifestation NOS |
| C34z.00 | Gout NOS |
| G557300 | Gouty tophi of heart |
| N023.00 | Gouty arthritis |
| N023100 | Gouty arthritis of the shoulder region |
| N023200 | Gouty arthritis of the upper arm |
| N023300 | Gouty arthritis of the forearm |
| N023400 | Gouty arthritis of the hand |
| N023600 | Gouty arthritis of the lower leg |
| N023700 | Gouty arthritis of the ankle and foot |
| N023800 | Gouty arthritis of toe |
| N023x00 | Gouty arthritis of multiple sites |
| N023y00 | Gouty arthritis of other specified site |
| N023z00 | Gouty arthritis NOS |
| Nyu1700 | [X]Other secondary gout |
| **Hypertension** | |
| 6627.00 | Good hypertension control |
| 6628.00 | Poor hypertension control |
| 662b.00 | Moderate hypertension control |
| 662F.00 | Hypertension treatm. started |
| 662G.00 | Hypertensive treatm.changed |
| 662O.00 | On treatment for hypertension |
| 8I3N.00 | Hypertension treatment refused |
| G2...00 | Hypertensive disease |
| G2...11 | BP - hypertensive disease |
| G20..00 | Essential hypertension |
| G20..11 | High blood pressure |
| G20..12 | Primary hypertension |
| G200.00 | Malignant essential hypertension |
| G201.00 | Benign essential hypertension |
| G202.00 | Systolic hypertension |
| G203.00 | Diastolic hypertension |
| G20z.00 | Essential hypertension NOS |
| G20z.11 | Hypertension NOS |
| G21..00 | Hypertensive heart disease |
| G210.00 | Malignant hypertensive heart disease |
| G210000 | Malignant hypertensive heart disease without CCF |
| G210100 | Malignant hypertensive heart disease with CCF |
| G210z00 | Malignant hypertensive heart disease NOS |
| G211.00 | Benign hypertensive heart disease |
| G211000 | Benign hypertensive heart disease without CCF |
| G211100 | Benign hypertensive heart disease with CCF |
| G211z00 | Benign hypertensive heart disease NOS |
| G21z.00 | Hypertensive heart disease NOS |
| G21z000 | Hypertensive heart disease NOS without CCF |
| G21z011 | Cardiomegaly - hypertensive |
| G21z100 | Hypertensive heart disease NOS with CCF |
| G21zz00 | Hypertensive heart disease NOS |
| G22..00 | Hypertensive renal disease |
| G220.00 | Malignant hypertensive renal disease |
| G221.00 | Benign hypertensive renal disease |
| G222.00 | Hypertensive renal disease with renal failure |
| G22z.00 | Hypertensive renal disease NOS |
| G22z.11 | Renal hypertension |
| G23..00 | Hypertensive heart and renal disease |
| G230.00 | Malignant hypertensive heart and renal disease |
| G231.00 | Benign hypertensive heart and renal disease |
| G232.00 | Hypertensive heart&renal dis wth (congestive) heart failure |
| G233.00 | Hypertensive heart and renal disease with renal failure |
| G234.00 | Hyperten heart&renal dis+both(congestv)heart and renal fail |
| G23z.00 | Hypertensive heart and renal disease NOS |
| G24..00 | Secondary hypertension |
| G240.00 | Secondary malignant hypertension |
| G240000 | Secondary malignant renovascular hypertension |
| G240z00 | Secondary malignant hypertension NOS |
| G241.00 | Secondary benign hypertension |
| G241000 | Secondary benign renovascular hypertension |
| G241z00 | Secondary benign hypertension NOS |
| G244.00 | Hypertension secondary to endocrine disorders |
| G24z.00 | Secondary hypertension NOS |
| G24z000 | Secondary renovascular hypertension NOS |
| G24z100 | Hypertension secondary to drug |
| G24zz00 | Secondary hypertension NOS |
| G25..00 | Stage 1 hypertension (NICE - Nat Ins for Hth Clin Excl 2011) |
| G25..11 | Stage 1 hypertension |
| G26..00 | Severe hypertension (Nat Inst for Health Clinical Ex 2011) |
| G26..11 | Severe hypertension |
| G27..00 | Hypertension resistant to drug therapy |
| G28..00 | Stage 2 hypertension (NICE - Nat Ins for Hth Clin Excl 2011) |
| G2y..00 | Other specified hypertensive disease |
| G2z..00 | Hypertensive disease NOS |
| G672.11 | Hypertensive crisis |
| Gyu2.00 | [X]Hypertensive diseases |
| Gyu2000 | [X]Other secondary hypertension |
| Gyu2100 | [X]Hypertension secondary to other renal disorders |
| **Osteoarthritis** | |
| 14G2.00 | H/O: osteoarthritis |
| 7P20400 | Delivery of rehabilitation for osteoarthritis |
| N05..00 | Osteoarthritis and allied disorders |
| N05..11 | Osteoarthritis |
| N050.00 | Generalised osteoarthritis - OA |
| N050000 | Generalised osteoarthritis of unspecified site |
| N050100 | Generalised osteoarthritis of the hand |
| N050200 | Generalised osteoarthritis of multiple sites |
| N050400 | Primary generalized osteoarthrosis |
| N050600 | Erosive osteoarthrosis |
| N050z00 | Generalised osteoarthritis NOS |
| N051.00 | Localised, primary osteoarthritis |
| N051000 | Localised, primary osteoarthritis of unspecified site |
| N051100 | Localised, primary osteoarthritis of the shoulder region |
| N051200 | Localised, primary osteoarthritis of the upper arm |
| N051300 | Localised, primary osteoarthritis of the forearm |
| N051400 | Localised, primary osteoarthritis of the hand |
| N051500 | Localised, primary osteoarthritis of the pelvic region/thigh |
| N051600 | Localised, primary osteoarthritis of the lower leg |
| N051700 | Localised, primary osteoarthritis of the ankle and foot |
| N051800 | Localised, primary osteoarthritis of other specified site |
| N051D00 | Localised, primary osteoarthritis of the wrist |
| N051E00 | Localised, primary osteoarthritis of toe |
| N051F00 | Localised, primary osteoarthritis of elbow |
| N051G00 | Osteoarthritis of spinal facet joint |
| N051z00 | Localised, primary osteoarthritis NOS |
| N052.00 | Localised, secondary osteoarthritis |
| N052000 | Localised, secondary osteoarthritis of unspecified site |
| N052100 | Localised, secondary osteoarthritis of the shoulder region |
| N052200 | Localised, secondary osteoarthritis of the upper arm |
| N052300 | Localised, secondary osteoarthritis of the forearm |
| N052400 | Localised, secondary osteoarthritis of the hand |
| N052500 | Localised, secondary osteoarthritis of pelvic region/thigh |
| N052600 | Localised, secondary osteoarthritis of the lower leg |
| N052700 | Localised, secondary osteoarthritis of the ankle and foot |
| N052800 | Localised, secondary osteoarthritis of other specified site |
| N052z00 | Localised, secondary osteoarthritis NOS |
| N053.00 | Localised osteoarthritis, unspecified |
| N053000 | Localised osteoarthritis, unspecified, of unspecified site |
| N053100 | Localised osteoarthritis, unspecified, of shoulder region |
| N053200 | Localised osteoarthritis, unspecified, of the upper arm |
| N053300 | Localised osteoarthritis, unspecified, of the forearm |
| N053400 | Localised osteoarthritis, unspecified, of the hand |
| N053500 | Localised osteoarthritis, unspecified, pelvic region/thigh |
| N053512 | Hip osteoarthitis NOS |
| N053600 | Localised osteoarthritis, unspecified, of the lower leg |
| N053611 | Patellofemoral osteoarthritis |
| N053700 | Localised osteoarthritis, unspecified, of the ankle and foot |
| N053800 | Localised osteoarthritis, unspecified, of other spec site |
| N053z00 | Localised osteoarthritis, unspecified, NOS |
| N054.00 | Oligoarticular osteoarthritis, unspecified |
| N054000 | Oligoarticular osteoarthritis, unspec, of unspecified sites |
| N054100 | Oligoarticular osteoarthritis, unspecified, of shoulder |
| N054200 | Oligoarticular osteoarthritis, unspecified, of upper arm |
| N054400 | Oligoarticular osteoarthritis, unspecified, of hand |
| N054500 | Oligoarticular osteoarthritis, unspecified, of pelvis/thigh |
| N054600 | Oligoarticular osteoarthritis, unspecified, of lower leg |
| N054700 | Oligoarticular osteoarthritis, unspecified, of ankle/foot |
| N054800 | Oligoarticular osteoarthritis, unspecified, other spec sites |
| N054900 | Oligoarticular osteoarthritis, unspecified, multiple sites |
| N054z00 | Osteoarthritis of more than one site, unspecified, NOS |
| N05z.00 | Osteoarthritis NOS |
| N05z000 | Osteoarthritis NOS, of unspecified site |
| N05z100 | Osteoarthritis NOS, of shoulder region |
| N05z200 | Osteoarthritis NOS, of the upper arm |
| N05z211 | Elbow osteoarthritis NOS |
| N05z300 | Osteoarthritis NOS, of the forearm |
| N05z311 | Wrist osteoarthritis NOS |
| N05z400 | Osteoarthritis NOS, of the hand |
| N05z411 | Finger osteoarthritis NOS |
| N05z412 | Thumb osteoarthritis NOS |
| N05z500 | Osteoarthritis NOS, pelvic region/thigh |
| N05z511 | Hip osteoarthritis NOS |
| N05z600 | Osteoarthritis NOS, of the lower leg |
| N05z611 | Knee osteoarthritis NOS |
| N05z700 | Osteoarthritis NOS, of ankle and foot |
| N05z711 | Ankle osteoarthritis NOS |
| N05z712 | Foot osteoarthritis NOS |
| N05z713 | Toe osteoarthritis NOS |
| N05z800 | Osteoarthritis NOS, other specified site |
| N05z900 | Osteoarthritis NOS, of shoulder |
| N05zA00 | Osteoarthritis NOS, of sternoclavicular joint |
| N05zB00 | Osteoarthritis NOS, of acromioclavicular joint |
| N05zC00 | Osteoarthritis NOS, of elbow |
| N05zD00 | Osteoarthritis NOS, of distal radio-ulnar joint |
| N05zE00 | Osteoarthritis NOS, of wrist |
| N05zF00 | Osteoarthritis NOS, of MCP joint |
| N05zG00 | Osteoarthritis NOS, of PIP joint of finger |
| N05zH00 | Osteoarthritis NOS, of DIP joint of finger |
| N05zJ00 | Osteoarthritis NOS, of hip |
| N05zK00 | Osteoarthritis NOS, of sacro-iliac joint |
| N05zL00 | Osteoarthritis NOS, of knee |
| N05zM00 | Osteoarthritis NOS, of tibio-fibular joint |
| N05zN00 | Osteoarthritis NOS, of ankle |
| N05zP00 | Osteoarthritis NOS, of subtalar joint |
| N05zQ00 | Osteoarthritis NOS, of talonavicular joint |
| N05zR00 | Osteoarthritis NOS, of other tarsal joint |
| N05zS00 | Osteoarthritis NOS, of 1st MTP joint |
| N05zT00 | Osteoarthritis NOS, of lesser MTP joint |
| N05zU00 | Osteoarthritis NOS, of IP joint of toe |
| N05zz00 | Osteoarthritis NOS |
| N11..12 | Osteoarthritis of spine |
| N110.12 | Osteoarthritis cervical spine |
| N11D.00 | Osteoarthritis of spine |
| N11D000 | Osteoarthritis of cervical spine |
| N11D100 | Osteoarthritis of thoracic spine |
| N11D200 | Osteoarthritis of lumbar spine |
| N11D300 | Osteoarthritis of spine NOS |
| N11z.11 | Osteoarthritis spine |
| N312.00 | Hypertrophic pulmonary osteoarthropathy |
| NyuC700 | [X]Other hypertrophic osteoarthropathy |
| **Osteopenia** | |
| 5681200 | Bone densimetry abnormal |
| 14O9.00 | At risk of osteoporosis |
| 585P.00 | Quantitative ultrasound scan of heel - result osteopenic |
| 58E5.00 | Forearm DXA scan result osteopenic |
| 58EB.00 | Heel DXA scan result osteopenic |
| 58EH.00 | Hip DXA scan result osteopenic |
| 58EN.00 | Lumbar DXA scan result osteopenic |
| 58EW.00 | Femoral neck DEXA scan result osteopenic |
| NyuB.00 | [X]Disorders of bone density and structure |
| NyuB700 | [X]Other specified disorders of bone density and structure |
| NyuBA00 | [X]Disorder of bone density and structure, unspecified |
| NyuBC00 | [X]Osteopenia |
| **Osteoporosis** | |
| 14GB.00 | History of osteoporosis |
| 14OD.00 | At risk of osteoporotic fracture |
| 585O.00 | Quantitative ultrasound scan of heel - result osteoporotic |
| 58E4.00 | Forearm DXA scan result osteoporotic |
| 58EA.00 | Heel DXA scan result osteoporotic |
| 58EG.00 | Hip DXA scan result osteoporotic |
| 58EM.00 | Lumbar DXA scan result osteoporotic |
| 58EV.00 | Femoral neck DEXA scan result osteoporotic |
| 66a2.00 | Osteoporosis treatment started |
| 66a3.00 | Osteoporosis treatment stopped |
| 66a4.00 | Osteoporosis treatment changed |
| 66a5.00 | Osteoporosis - no treatment |
| 66a6.00 | Osteoporosis - dietary advice |
| 66a7.00 | Osteoporosis - dietary assessment |
| 66a8.00 | Osteoporosis - exercise advice |
| 66a9.00 | Osteoporosis - falls prevention |
| 66aA.00 | Osteoporosis - treatment response |
| 66aB.00 | Osteoporosis - no treatment response |
| 8B6b.00 | Osteoporosis medication prophylaxis |
| 8I6c.00 | Osteoporosis treatment not indicated |
| 9kj0.00 | Bone sparing drug treatment offered for osteoporosis - ESA |
| N330.00 | Osteoporosis |
| N330000 | Osteoporosis, unspecified |
| N330100 | Senile osteoporosis |
| N330200 | Postmenopausal osteoporosis |
| N330300 | Idiopathic osteoporosis |
| N330400 | Dissuse osteoporosis |
| N330500 | Drug-induced osteoporosis |
| N330600 | Postoophorectomy osteoporosis |
| N330700 | Postsurgical malabsorption osteoporosis |
| N330800 | Localized osteoporosis - Lequesne |
| N330900 | Osteoporosis in multiple myelomatosis |
| N330A00 | Osteoporosis in endocrine disorders |
| N330B00 | Vertebral osteoporosis |
| N330C00 | Osteoporosis localized to spine |
| N330D00 | Osteoporosis due to corticosteroids |
| N330z00 | Osteoporosis NOS |
| N331.00 | Pathological fracture |
| N331.14 | Osteoporotic vertebral collapse |
| N331000 | Pathological fracture of thoracic vertebra |
| N331100 | Pathological fracture of lumbar vertebra |
| N331200 | Postoophorectomy osteoporosis with pathological fracture |
| N331300 | Osteoporosis of disuse with pathological fracture |
| N331400 | Postsurgical malabsorption osteoporosis with path fracture |
| N331500 | Drug-induced osteoporosis with pathological fracture |
| N331600 | Idiopathic osteoporosis with pathological fracture |
| N331800 | Osteoporosis + pathological fracture lumbar vertebrae |
| N331900 | Osteoporosis + pathological fracture thoracic vertebrae |
| N331A00 | Osteoporosis + pathological fracture cervical vertebrae |
| N331B00 | Postmenopausal osteoporosis with pathological fracture |
| N331C00 | Pathological fracture of cervical vertebra |
| N331H00 | Collapse of cervical vertebra due to osteoporosis |
| N331J00 | Collapse of lumbar vertebra due to osteoporosis |
| N331K00 | Collapse of thoracic vertebra due to osteoporosis |
| N331L00 | Collapse of vertebra due to osteoporosis NOS |
| N331M00 | Fragility fracture due to unspecified osteoporosis |
| N331M11 | Minimal trauma fracture due to unspecified osteoporosis |
| N331y00 | Other specified pathological fracture |
| N331z00 | Pathological fracture NOS |
| N374600 | Osteoporotic kyphosis |
| NyuB000 | [X]Other osteoporosis with pathological fracture |
| NyuB100 | [X]Other osteoporosis |
| NyuB200 | [X]Osteoporosis in other disorders classified elsewhere |
| NyuB800 | [X]Unspecified osteoporosis with pathological fracture |
| **Rheumatoid Arthritis** | |
| 14G1.00 | H/O: rheumatoid arthritis |
| 38DZ.00 | Disease activity score in rheumatoid arthritis |
| 38DZ000 | Disease activity score 28 joint in rheumatoid arthritis |
| 7P20300 | Delivery of rehabilitation for rheumatoid arthritis |
| F371200 | Polyneuropathy in rheumatoid arthritis |
| F396400 | Myopathy due to rheumatoid arthritis |
| N04..00 | Rheumatoid arthritis and other inflammatory polyarthropathy |
| N040.00 | Rheumatoid arthritis |
| N040000 | Rheumatoid arthritis of cervical spine |
| N040100 | Other rheumatoid arthritis of spine |
| N040200 | Rheumatoid arthritis of shoulder |
| N040300 | Rheumatoid arthritis of sternoclavicular joint |
| N040400 | Rheumatoid arthritis of acromioclavicular joint |
| N040500 | Rheumatoid arthritis of elbow |
| N040600 | Rheumatoid arthritis of distal radio-ulnar joint |
| N040700 | Rheumatoid arthritis of wrist |
| N040800 | Rheumatoid arthritis of MCP joint |
| N040900 | Rheumatoid arthritis of PIP joint of finger |
| N040A00 | Rheumatoid arthritis of DIP joint of finger |
| N040B00 | Rheumatoid arthritis of hip |
| N040C00 | Rheumatoid arthritis of sacro-iliac joint |
| N040D00 | Rheumatoid arthritis of knee |
| N040E00 | Rheumatoid arthritis of tibio-fibular joint |
| N040F00 | Rheumatoid arthritis of ankle |
| N040G00 | Rheumatoid arthritis of subtalar joint |
| N040H00 | Rheumatoid arthritis of talonavicular joint |
| N040J00 | Rheumatoid arthritis of other tarsal joint |
| N040K00 | Rheumatoid arthritis of 1st MTP joint |
| N040L00 | Rheumatoid arthritis of lesser MTP joint |
| N040M00 | Rheumatoid arthritis of IP joint of toe |
| N040P00 | Seronegative rheumatoid arthritis |
| N040S00 | Rheumatoid arthritis - multiple joint |
| N040T00 | Flare of rheumatoid arthritis |
| N047.00 | Seropositive errosive rheumatoid arthritis |
| N04X.00 | Seropositive rheumatoid arthritis, unspecified |
| N04y012 | Fibrosing alveolitis associated with rheumatoid arthritis |
| Nyu1000 | [X]Rheumatoid arthritis+involvement/other organs or systems |
| Nyu1100 | [X]Other seropositive rheumatoid arthritis |
| Nyu1200 | [X]Other specified rheumatoid arthritis |
| Nyu1G00 | [X]Seropositive rheumatoid arthritis, unspecified |
| ZV7y100 | [V]Screening for rheumatoid arthritis |
| M160.00 | Psoriatic arthropathy |
| M160100 | Distal interphalangeal psoriatic arthropathy |
| M160200 | Arthritis mutilans |
| M160z00 | Psoriatic arthropathy NOS |
| Nyu1300 | [X]Other psoriatic arthropathies |
| G5y8.00 | Rheumatoid myocarditis |
| G5yA.00 | Rheumatoid carditis |
| H570.00 | Rheumatoid lung |
| N04..11 | Inflammatory polyarthropathy |
| N040N00 | Rheumatoid vasculitis |
| N040Q00 | Rheumatoid bursitis |
| N040R00 | Rheumatoid nodule |
| N041.00 | Felty's syndrome |
| N042.00 | Other rheumatoid arthropathy + visceral/systemic involvement |
| N042000 | Rheumatic carditis |
| N042100 | Rheumatoid lung disease |
| N042200 | Rheumatoid nodule |
| N042z00 | Rheumatoid arthropathy + visceral/systemic involvement NOS |
| N04y000 | Rheumatoid lung |
| N04y011 | Caplan's syndrome |
| N04y100 | Sero negative arthritis |
| N04y111 | Sero negative polyarthritis |
| N093.00 | Palindromic rheumatism |
| N093.12 | Intermittent hydrarthrosis |
| N093.13 | Intermittent joint effusion |
| N093900 | Palindromic rheumatism of multiple sites |
| N093z00 | Palindromic rheumatism NOS |
| N04y.00 | Other specified inflammatory polyarthropathy |
| N04yz00 | Other specified inflammatory polyarthropathy NOS |
| N04z.00 | Inflammatory polyarthropathy NOS |
| N005.00 | Adult Still's Disease |
| N04y200 | Adult-onset Still's disease |
| 388p.00 | BASDAI - Bath ankylosing spondylitis disease activity index |
| N100.00 | Ankylosing spondylitis |
| N093000 | Palindromic rheumatism of unspecified site |
| N093100 | Palindromic rheumatism of the shoulder region |
| N093200 | Palindromic rheumatism of the upper arm |
| N093400 | Palindromic rheumatism of the hand |
| N093500 | Palindromic rheumatism of the pelvic region and thigh |
| N093600 | Palindromic rheumatism of the lower leg |
| N093700 | Palindromic rheumatism of the ankle and foot |
| N093800 | Palindromic rheumatism of other specified site |

ANNEX 7. Definition of concomitant medications

| **Product code** | **Code term** |
| --- | --- |
| **Denosumab - Prolia** | |
| 44084 | Prolia 60mg/1ml solution for injection pre-filled syringes (Amgen Ltd) |
| 44004 | Denosumab 60mg/1ml solution for injection pre-filled syringes |
| **Denosumab - XGEVA** | |
| 54058 | Denosumab 120mg/1.7ml solution for injection vials |
| 58018 | Xgeva 120mg/1.7ml solution for injection vials (Amgen Ltd) |
| **Alendronic acid** | |
| 2298 | Alendronic acid 10mg tablets |
| 544 | Fosamax Once Weekly 70mg tablets (Merck Sharp & Dohme Ltd) |
| 688 | Alendronic acid 70mg tablets |
| 7530 | Alendronic acid 5mg tablets |
| 782 | Fosamax 5mg tablets (Merck Sharp & Dohme Ltd) |
| 663 | Fosamax 10mg tablets (Merck Sharp & Dohme Ltd) |
| 43958 | Alendronic acid 70mg tablets (Actavis UK Ltd) |
| 37218 | Alendronic acid 70mg tablets (Teva UK Ltd) |
| 37217 | Alendronic acid 10mg tablets (Teva UK Ltd) |
| 47380 | Alendronic acid 70mg tablets (Arrow Generics Ltd) |
| 52624 | Alendronic acid 70mg tablets (Phoenix Healthcare Distribution Ltd) |
| 56260 | Alendronic acid 70mg tablets (Kent Pharmaceuticals Ltd) |
| 58744 | Fosamax Once Weekly 70mg tablets (DE Pharmaceuticals) |
| 56730 | Alendronic acid 70mg tablets (Almus Pharmaceuticals Ltd) |
| 65008 | Alendronic acid 70mg effervescent tablets sugar free |
| 61686 | Alendronic acid 70mg tablets (DE Pharmaceuticals) |
| 35937 | Alendronic acid 70mg tablets (A A H Pharmaceuticals Ltd) |
| 40449 | Alendronic acid 70mg tablets (PLIVA Pharma Ltd) |
| 46245 | Alendronic acid 70mg tablets (Mylan) |
| 54566 | Alendronic acid 10mg tablets (A A H Pharmaceuticals Ltd) |
| 51877 | Alendronic acid 70mg tablets (Alliance Healthcare (Distribution) Ltd) |
| 59485 | Alendronic acid 10mg tablets (Accord Healthcare Ltd) |
| 55965 | Alendronic acid 70mg tablets (Zentiva) |
| 56061 | Alendronic acid 10mg tablets (Actavis UK Ltd) |
| 65905 | Alendronic acid 10mg tablets (Mylan) |
| 59079 | Alendronic acid 10mg tablets (Almus Pharmaceuticals Ltd) |
| 59247 | Fosamax Once Weekly 70mg tablets (Necessity Supplies Ltd) |
| 50278 | Alendronic acid 70mg tablets (Wockhardt UK Ltd) |
| 52834 | Alendronic acid 70mg tablets (Accord Healthcare Ltd) |
| 57875 | Fosamax Once Weekly 70mg tablets (Lexon (UK) Ltd) |
| 59555 | Alendronic acid 10mg tablets (Alliance Healthcare (Distribution) Ltd) |
| 63008 | Alendronic acid 70mg tablets (Somex Pharma) |
| 64331 | Alendronic acid 10mg tablets (DE Pharmaceuticals) |
| 66203 | Binosto 70mg effervescent tablets (Internis Pharmaceuticals Ltd) |
| 69995 | Alendronic acid 35mg/5ml oral solution |
| 50880 | Fosamax 10mg tablets (Necessity Supplies Ltd) |
| 52284 | Fosamax 10mg tablets (Sigma Pharmaceuticals Plc) |
| 63175 | Alendronic acid 10mg tablets (Phoenix Healthcare Distribution Ltd) |
| 52564 | Alendronic acid 70mg/100ml oral solution unit dose sugar free (Rosemont Pharmaceuticals Ltd) |
| 55998 | Alendronic acid 70mg/75ml oral solution unit dose |
| 45787 | Alendronic acid 70mg/100ml oral solution unit dose sugar free |
| 55295 | Alendronic acid 70mg/100ml oral solution unit dose sugar free (Alliance Healthcare (Distribution) Ltd) |
| 60144 | Alendronic acid 70mg/100ml oral solution unit dose sugar free (Waymade Healthcare Plc) |
| 10227 | Fosavance tablets (Merck Sharp & Dohme Ltd) |
| 7224 | Alendronic acid 70mg / Colecalciferol 70microgram tablets |
| 66485 | Fosavance tablets (Lexon (UK) Ltd) |
| 70927 | Alendronic acid 70mg / Colecalciferol 70microgram tablets (Creo Pharma Ltd) |
| **Etidronate** | |
| 54436 | Etidronate disodium Oral solution |
| 53169 | Etidronate disodium 400mg Tablet |
| 766 | Didronel 200mg tablets (Warner Chilcott UK Ltd) |
| 4680 | Etidronate disodium 200mg tablets |
| 63371 | Etidronate disodium 200mg tablets (Mylan) |
| 11368 | Calcium carbonate 1.25g effervescent tablets and Disodium etidronate 400mg tablets |
| 468 | Didronel PMO tablets (Warner Chilcott UK Ltd) |
| **Ibandronic acid** | |
| 7146 | Ibandronic sodium monohydrate |
| 10193 | Ibandronic sodium monohydrate |
| 24998 | Ibandronic sodium monohydrate |
| 32426 | Ibandronic sodium monohydrate |
| 51342 | Ibandronic sodium monohydrate |
| 23223 | Ibandronic Sodium Monohydrate |
| 26913 | Ibandronic sodium monohydrate |
| 56369 | Ibandronic sodium monohydrate |
| 7112 | Ibandronic sodium monohydrate |
| 66618 | Ibandronic sodium monohydrate |
| 44265 | Ibandronic Sodium Monohydrate |
| 56030 | Ibandronic sodium monohydrate |
| 25387 | Ibandronic sodium monohydrate |
| 47911 | Ibandronic sodium monohydrate |
| 54453 | Ibandronic sodium monohydrate |
| 62017 | Ibandronic sodium monohydrate |
| 57980 | Ibandronic sodium monohydrate |
| 71000 | Ibandronic sodium monohydrate |
| 59587 | Ibandronic sodium monohydrate |
| 67159 | Ibandronic sodium monohydrate |
| **Pamidronic acid** | |
| 16204 | Pamidronate disodium |
| 16430 | Pamidronate disodium |
| 30448 | Pamidronate disodium |
| 42102 | Pamidronate disodium |
| 47838 | Pamidronate disodium |
| 29415 | Pamidronate disodium |
| 26324 | Pamidronate disodium |
| 48507 | Pamidronate disodium |
| 33824 | Pamidronate disodium |
| 41494 | Pamidronate disodium |
| 35027 | Pamidronate disodium |
| 23563 | Pamidronate disodium |
| 36083 | Pamidronate disodium |
| 63164 | Pamidronate disodium |
| 52255 | Pamidronate disodium |
| 10025 | Pamidronate disodium 3mg/ml Injection |
| 21485 | Pamidronate disodium 15mg powder for concentrate for infusion vials |
| 16904 | Pamidronate disodium 90mg powder for concentrate for infusion vials |
| 23530 | Pamidronate disodium 30mg powder for concentrate for infusion vials |
| **Risedronic acid** | |
| 7527 | Risedronate sodium |
| 7089 | Risedronate sodium |
| 6058 | Risedronate sodium |
| 6634 | Risedronate sodium |
| 7546 | Risedronate sodium |
| 52373 | Risedronate sodium |
| 6084 | Risedronate sodium |
| 66028 | Risedronate sodium |
| 48013 | Risedronate sodium |
| 61313 | Risedronate sodium |
| 44511 | Risedronate sodium |
| 69630 | Risedronate sodium |
| 69929 | Risedronate sodium |
| 59449 | Risedronate sodium |
| 71209 | Risedronate sodium |
| 64431 | Risedronate sodium |
| 58618 | Risedronate sodium |
| 63802 | Risedronate sodium |
| 67078 | Risedronate sodium |
| 69958 | Risedronate sodium |
| 56663 | Risedronate sodium |
| 59916 | Risedronate sodium |
| 60288 | Risedronate sodium |
| 56431 | Risedronate sodium |
| 65971 | Risedronate sodium |
| 45280 | Risedronate Sodium/Calcium Carbonate |
| 37833 | Actonel Combi 35mg tablets and 1000mg/880unit effervescent granules sachets (Teva UK Ltd) |
| 37575 | Risedronate sodium 35mg with calcium carbonate 2500mg & colecalciferol 22micrograms tablets and granules |
| **Sodium clodronate** | |
| 6568 | Sodium clodronate |
| 9189 | Sodium clodronate |
| 5629 | Sodium clodronate |
| 54989 | Sodium clodronate |
| 3680 | Sodium clodronate |
| 4868 | Sodium clodronate |
| 11244 | Sodium clodronate |
| 4927 | Sodium clodronate |
| 39043 | Sodium clodronate |
| **Strontium ranelate** | |
| 7227 | Strontium ranelate 2g granules sachets sugar free |
| 6792 | Protelos 2g granules sachets (Servier Laboratories Ltd) |
| 57138 | Protelos 2g granules sachets (DE Pharmaceuticals) |
| 71060 | Protelos 2g granules sachets (Lexon (UK) Ltd) |
| Tiludronic acid |  |
| 9208 | Tiludronic acid 200mg tablets |
| 9525 | Skelid 200mg tablets (Sanofi) |
| **Zoledronic acid** | |
| 36400 | Aclasta 5mg/100ml infusion bottles (Novartis Pharmaceuticals UK Ltd) |
| 46982 | Zoledronic acid 4mg/100ml infusion bottles |
| 63400 | Zoledronic acid 4mg/100ml infusion bottles (medac UK) |
| 30987 | Zometa 4mg/5ml solution for infusion vials (Novartis Pharmaceuticals UK Ltd) |
| 15368 | Zoledronic acid 4mg/5ml solution for infusion vials |
| 61338 | Zoledronic acid 4mg/5ml concentrate for solution for infusion vials (Dr Reddy's Laboratories (UK) Ltd) |
| 36399 | Zoledronic acid 5mg/100ml infusion bottles |
| 64122 | Zoledronic acid 5mg/100ml infusion bags |
| 47866 | Zometa 4mg/100ml infusion bottles (Novartis Pharmaceuticals UK Ltd) |
| 68625 | Zoledronic acid 5mg/100ml solution for infusion vials (Dr Reddy's Laboratories (UK) Ltd) |
| 59494 | Zoledronic acid 4mg/5ml solution for infusion vials (Actavis UK Ltd) |
| 60022 | Zoledronic acid 5mg/100ml solution for infusion vials |
| 60644 | Zoledronic acid 4mg/100ml solution for infusion vials |
| 62240 | Zoledronic acid 4mg/5ml concentrate for solution for infusion vials (Intrapharm Laboratories Ltd) |
| 65192 | Zoledronic acid 4mg/100ml) infusion bags |
| 71279 | Zoledronic acid 4mg/5ml concentrate for solution for infusion vials (Peckforton Pharmaceuticals Ltd) |
| 61044 | Zoledronic acid 4mg/5ml concentrate for solution for infusion vials (medac UK) |
| 65160 | Zoledronic acid 4mg/5ml concentrate for solution for infusion vials (Pfizer Ltd) |
| 70932 | Zoledronic acid 4mg/5ml concentrate for solution for infusion vials (Accord Healthcare Ltd) |
| 63043 | Zoledronic acid 4mg/5ml concentrate for solution for infusion vials (Sandoz Ltd |
| **Strong Opioids** | |
| 23906 | Alfentanil 5mg/1ml solution for injection ampoules |
| 24790 | Alfentanil 500micrograms/ml injection |
| 28567 | Rapifen 100microgram/ml Paediatric injection (Janssen-Cilag Ltd) |
| 32381 | Rapifen 5mg/ml Concentrate for solution for infusion (Janssen-Cilag Ltd) |
| 35500 | Alfentanil 1mg/2ml solution for injection ampoules |
| 36185 | Alfentanil 5mg/10ml solution for injection ampoules |
| 37251 | Alfentanil 0.1% nasal spray |
| 38524 | Rapifen 1mg/2ml solution for injection ampoules (Piramal Critical Care Ltd) |
| 40427 | Rapifen 500microgram/ml Injection (Janssen-Cilag Ltd) |
| 43384 | Rapifen Intensive Care 5mg/1ml solution for injection ampoules (Piramal Critical Care Ltd) |
| 43720 | Rapifen 5mg/10ml solution for injection ampoules (Piramal Critical Care Ltd) |
| 56581 | Alfentanil 5mg/5ml buccal spray |
| 60080 | Alfentanil 500micrograms/1ml solution for injection ampoules |
| 484 | Equagesic Tablet (Wyeth Pharmaceuticals) |
| 685 | Aspav dispersible tablets (Actavis UK Ltd) |
| 4557 | ASPIRIN & PAPAVERUTUM 10 MG TAB |
| 6226 | Aspirin 500mg / Papaveretum 7.71mg dispersible tablets sugar free |
| 11326 | Meprobamate with ethoheptazine citrate and aspirin Tablet |
| 18261 | Aspirin 500mg with Papaveretum 7.71mg dispersible tablets |
| 21932 | ETHOHEPTALINE CITRATE/ASPIRIN MG TAB |
| 23250 | ASPIRIN /ETHOHEPTAZINE CITRATE /MEPROBAM 250 MG TAB |
| 320 | Buprenorphine HCl 300micrograms injection |
| 396 | Buprenorphine 200microgram sublingual tablets sugar free |
| 3064 | Buprenorphine 400microgram sublingual tablets sugar free |
| 3522 | Temgesic 200microgram sublingual tablets (Indivior UK Ltd) |
| 5936 | Transtec 35micrograms/hour transdermal patches (Napp Pharmaceuticals Ltd) |
| 6040 | Transtec 52.5micrograms/hour transdermal patches (Napp Pharmaceuticals Ltd) |
| 6056 | Buprenorphine 8mg sublingual tablets sugar free |
| 6181 | Transtec 70micrograms/hour transdermal patches (Napp Pharmaceuticals Ltd) |
| 6210 | Subutex 8mg sublingual tablets (Indivior UK Ltd) |
| 6547 | Buprenorphine 2mg sublingual tablets sugar free |
| 6879 | Buprenorphine 35micrograms/hour transdermal patches |
| 6917 | Buprenorphine 52.5micrograms/hour transdermal patches |
| 7236 | Buprenorphine 10micrograms/hour transdermal patches |
| 7238 | Buprenorphine 20micrograms/hour transdermal patches |
| 7334 | Buprenorphine 5micrograms/hour transdermal patches |
| 7457 | Temgesic 0.3mg/ml Injection (Reckitt Benckiser Healthcare (UK) Ltd) |
| 7555 | BuTrans 5micrograms/hour transdermal patches (Napp Pharmaceuticals Ltd) |
| 8017 | Temgesic 400microgram sublingual tablets (Indivior UK Ltd) |
| 10077 | Subutex 2mg sublingual tablets (Indivior UK Ltd) |
| 10205 | BuTrans 10micrograms/hour transdermal patches (Napp Pharmaceuticals Ltd) |
| 11584 | Buprenorphine 70micrograms/hour transdermal patches |
| 13031 | Subutex 0.4mg sublingual tablets (Indivior UK Ltd) |
| 13300 | BuTrans 20micrograms/hour transdermal patches (Napp Pharmaceuticals Ltd) |
| 35169 | Suboxone 8mg/2mg sublingual tablets (Indivior UK Ltd) |
| 35170 | Suboxone 2mg/500microgram sublingual tablets (Indivior UK Ltd) |
| 35269 | Temgesic 300micrograms/1ml solution for injection ampoules (Indivior UK Ltd) |
| 35681 | Buprenorphine 2mg / Naloxone 500microgram sublingual tablets sugar free |
| 35682 | Buprenorphine 8mg / Naloxone 2mg sublingual tablets sugar free |
| 38311 | Naloxone hc 2mg + 8mg Tablet |
| 40211 | Buprenorphine 2mg sublingual tablets sugar free (Teva UK Ltd) |
| 40212 | Buprenorphine 8mg sublingual tablets sugar free (Teva UK Ltd) |
| 40473 | Buprenorphine 300micrograms/1ml solution for injection ampoules |
| 42074 | Naloxone hc 500micrograms + 2mg Tablet |
| 50380 | Subutex 2mg sublingual tablets (Lexon (UK) Ltd) |
| 54806 | Transtec 52.5micrograms/hour transdermal patches (DE Pharmaceuticals) |
| 56671 | BuTrans 5micrograms/hour transdermal patches (Waymade Healthcare Plc) |
| 57454 | Prefibin 8mg sublingual tablets (Sandoz Ltd) |
| 58273 | Buprenorphine 2mg sublingual tablets sugar free (A A H Pharmaceuticals Ltd) |
| 58766 | BuTrans 10micrograms/hour transdermal patches (Waymade Healthcare Plc) |
| 59146 | BuTrans 20micrograms/hour transdermal patches (Waymade Healthcare Plc) |
| 59392 | Hapoctasin 70micrograms/hour transdermal patches (Actavis UK Ltd) |
| 59473 | Hapoctasin 52.5micrograms/hour transdermal patches (Actavis UK Ltd) |
| 59618 | Transtec 35micrograms/hour transdermal patches (Mawdsley-Brooks & Company Ltd) |
| 59970 | Buprenorphine 2mg sublingual tablets sugar free (Actavis UK Ltd) |
| 60053 | Tephine 200microgram sublingual tablets (Sandoz Ltd) |
| 60170 | Hapoctasin 35micrograms/hour transdermal patches (Actavis UK Ltd) |
| 60943 | Transtec 35micrograms/hour transdermal patches (Sigma Pharmaceuticals Plc) |
| 61100 | Tephine 400microgram sublingual tablets (Sandoz Ltd) |
| 62675 | Buprenorphine 200microgram sublingual tablets sugar free (A A H Pharmaceuticals Ltd) |
| 62776 | Buprenorphine 4mg sublingual tablets sugar free |
| 62874 | Buprenorphine 6mg sublingual tablets sugar free |
| 62969 | Buprenorphine 8mg sublingual tablets sugar free (Zentiva) |
| 63640 | Subutex 2mg sublingual tablets (DE Pharmaceuticals) |
| 63788 | Buprenorphine 1mg sublingual tablets sugar free |
| 64155 | Buprenorphine 400microgram sublingual tablets sugar free (Teva UK Ltd) |
| 64847 | Gabup 8mg sublingual tablets (Martindale Pharmaceuticals Ltd) |
| 65157 | Buprenorphine 2mg sublingual tablets sugar free (Sigma Pharmaceuticals Plc) |
| 66280 | BuTrans 15micrograms/hour transdermal patches (Napp Pharmaceuticals Ltd) |
| 66463 | Buprenorphine 15micrograms/hour transdermal patches |
| 66470 | Butec 10micrograms/hour transdermal patches (Qdem Pharmaceuticals Ltd) |
| 66689 | Butec 5micrograms/hour transdermal patches (Qdem Pharmaceuticals Ltd) |
| 66695 | Butec 20micrograms/hour transdermal patches (Qdem Pharmaceuticals Ltd) |
| 67018 | Buprenorphine 35micrograms/hour transdermal patches (A A H Pharmaceuticals Ltd) |
| 67356 | Transtec 70micrograms/hour transdermal patches (Lexon (UK) Ltd) |
| 67901 | Transtec 52.5micrograms/hour transdermal patches (Lexon (UK) Ltd) |
| 68167 | Reletrans 5micrograms/hour transdermal patches (Sandoz Ltd) |
| 68172 | Reletrans 20micrograms/hour transdermal patches (Sandoz Ltd) |
| 68196 | Reletrans 15micrograms/hour transdermal patches (Sandoz Ltd) |
| 68241 | Reletrans 10micrograms/hour transdermal patches (Sandoz Ltd) |
| 68402 | Panitaz 5micrograms/hour transdermal patches (Dr Reddy's Laboratories (UK) Ltd) |
| 68472 | Prenotrix 52.5micrograms/hour transdermal patches (Genesis Pharmaceuticals Ltd) |
| 68479 | Bupeaze 70micrograms/hour transdermal patches (Dr Reddy's Laboratories (UK) Ltd) |
| 68559 | Panitaz 10micrograms/hour transdermal patches (Dr Reddy's Laboratories (UK) Ltd) |
| 68743 | Bupeaze 35micrograms/hour transdermal patches (Dr Reddy's Laboratories (UK) Ltd) |
| 68848 | Buplast 52.5micrograms/hour transdermal patches (Mylan) |
| 68888 | Sevodyne 20micrograms/hour transdermal patches (Aspire Pharma Ltd) |
| 68889 | Sevodyne 10micrograms/hour transdermal patches (Aspire Pharma Ltd) |
| 68890 | Sevodyne 5micrograms/hour transdermal patches (Aspire Pharma Ltd) |
| 68910 | Suboxone 16mg/4mg sublingual tablets (Indivior UK Ltd) |
| 68988 | Buprenorphine 8mg oral lyophilisates sugar free |
| 68989 | Buprenorphine 2mg oral lyophilisates sugar free |
| 69243 | Bupeaze 52.5micrograms/hour transdermal patches (Dr Reddy's Laboratories (UK) Ltd) |
| 69254 | Buplast 35micrograms/hour transdermal patches (Mylan) |
| 69315 | Butec 15micrograms/hour transdermal patches (Qdem Pharmaceuticals Ltd) |
| 69795 | Prenotrix 35micrograms/hour transdermal patches (Genesis Pharmaceuticals Ltd) |
| 69942 | Buprenorphine 400microgram sublingual tablets sugar free (Phoenix Healthcare Distribution Ltd) |
| 70065 | Espranor 2mg oral lyophilisates (Martindale Pharmaceuticals Ltd) |
| 70066 | Espranor 8mg oral lyophilisates (Martindale Pharmaceuticals Ltd) |
| 70117 | Panitaz 20micrograms/hour transdermal patches (Dr Reddy's Laboratories (UK) Ltd) |
| 70124 | Relevtec 70micrograms/hour transdermal patches (Sandoz Ltd) |
| 70139 | Relevtec 35micrograms/hour transdermal patches (Sandoz Ltd) |
| 70283 | Buprenorphine 16mg / Naloxone 4mg sublingual tablets sugar free |
| 70460 | Bupramyl 10micrograms/hour transdermal patches (Mylan) |
| 70461 | Bupramyl 5micrograms/hour transdermal patches (Mylan) |
| 70464 | Subutex 2mg sublingual tablets (Waymade Healthcare Plc) |
| 70631 | Relevtec 52.5micrograms/hour transdermal patches (Sandoz Ltd) |
| 71310 | Bupramyl 20micrograms/hour transdermal patches (Mylan) |
| 71410 | Suboxone 8mg/2mg sublingual tablets (Mawdsley-Brooks & Company Ltd) |
| 71630 | Natzon 0.4mg sublingual tablets (Morningside Healthcare Ltd) |
| 71695 | Prefibin 0.4mg sublingual tablets (Sandoz Ltd) |
| 71711 | Busiete 10micrograms/hour transdermal patches (Teva UK Ltd) |
| 72098 | Turgeon 35micrograms/hour transdermal patches (Teva UK Ltd) |
| 72160 | Turgeon 52.5micrograms/hour transdermal patches (Teva UK Ltd) |
| 19317 | Omnopon 10mg Tablet (Roche Products Ltd) |
| 19764 | Papaveretum 10mg tablet |
| 3165 | Diamorphine 10mg Injection (Manufacturer unknown) |
| 5079 | Diamorphine hydrochloride 5mg powder for injection solution |
| 5668 | Diamorphine hydrochloride 30mg powder for injection solution |
| 5670 | Diamorphine hydrochloride 10mg powder for injection solution |
| 6458 | Diamorphine hydrochloride 500mg powder for injection solution |
| 6459 | Diamorphine hydrochloride 100mg powder for injection solution |
| 7114 | Diamorphine 3mg/5ml oral solution |
| 7849 | Diamorphine 100mg Injection (Manufacturer unknown) |
| 7999 | Diamorphine 5mg Injection (Manufacturer unknown) |
| 8040 | Diamorphine 30mg Injection (Manufacturer unknown) |
| 8460 | DIAMORPHINE 1.5 GM INJ |
| 8735 | Diamorphine 5mg/5ml Oral solution (Manufacturer unknown) |
| 8823 | DIAMORPHINE HYDROCHLORIDE 60 MG INJ |
| 8866 | Diamorphine 10mg Tablet (Manufacturer unknown) |
| 9053 | Diamorphine 500mg Injection (Manufacturer unknown) |
| 9126 | DIAMORPHINE 4 GM INJ |
| 9945 | Diamorphine 10mg Tablet (Aurum Pharmaceuticals Ltd) |
| 10473 | DIAMORPHINE 10 MG LIN |
| 13364 | DIAMORPHINE 20 MG ELI |
| 13420 | Diamorphine 15mg/5ml Oral solution (Manufacturer unknown) |
| 15339 | Diamorphine 10mg/5ml oral solution |
| 15514 | DIAMORPHINE 2.5 MG LIN |
| 15793 | Diamorphine hydrochloride powder |
| 17163 | DIAMORPHINE 1 MG INJ |
| 18792 | Diamorphine 10mg tablets |
| 18965 | DIAMORPHINE 2.5 GM INJ |
| 18977 | DIAMORPHINE HYDROCHLORIDE 20 MG INJ |
| 19351 | DIAMORPHINE 3 GM INJ |
| 20713 | DIAMORPHINE 30 MG SUP |
| 20752 | DIAMORPHINE 15 MG INJ |
| 21868 | Diamorphine hydrochloride and cocaine oral solution |
| 23778 | DIAMORPHINE 5 MG SUP |
| 23785 | DIAMORPHINE 10 MG SUP |
| 24108 | Diagesil 10mg Injection (Berk Pharmaceuticals Ltd) |
| 24640 | Diaphine 100mg Injection (Napp Pharmaceuticals Ltd) |
| 24697 | DIAMORPHINE 50 MG SUP |
| 24840 | DIAMORPHINE 75 MG SUP |
| 25649 | DIAMORPHINE 60 MG SUP |
| 25830 | DIAMORPHINE 20 MG SUP |
| 26407 | DIAMORPHINE 1 GM SUP |
| 27352 | DIAMORPHINE 64 MG INJ |
| 28396 | DIAMORPHINE 100 MG SUP |
| 28711 | Diamorphine hydrochloride bpc 1973 3mg/5ml oral solution |
| 29014 | Diaphine 10mg Injection (Napp Pharmaceuticals Ltd) |
| 29500 | Diamorphine 5mg/5ml oral solution |
| 29591 | DIAMORPHINE 25 MG SUP |
| 29592 | DIAMORPHINE 40 MG SUP |
| 30320 | DIAMORPHINE 200 MG SUP |
| 30514 | Diaphine 30mg Injection (Napp Pharmaceuticals Ltd) |
| 30698 | Diagesil 500mg Injection (Berk Pharmaceuticals Ltd) |
| 30761 | Diamorphine 10mg/5ml Oral solution (Manufacturer unknown) |
| 31033 | Diamorphine hydrochloride 3mg/5ml oral solution |
| 31960 | Diamorphine 15mg/5ml oral solution |
| 32687 | DIAMORPHINE & TERPOIN LIN |
| 32688 | Diagesil 5mg Injection (Berk Pharmaceuticals Ltd) |
| 32897 | Diamorphine 5mg powder for solution for injection ampoules (Novartis Vaccines and Diagnostics Ltd) |
| 34489 | Diamorphine hydrochloride 10mg Injection (Hillcross Pharmaceuticals Ltd) |
| 34786 | Diamorphine 10mg powder for solution for injection ampoules (Novartis Vaccines and Diagnostics Ltd) |
| 34787 | Diamorphine 30mg powder for solution for injection ampoules (Novartis Vaccines and Diagnostics Ltd) |
| 41722 | Diamorphine 30mg powder for solution for injection ampoules (Wockhardt UK Ltd) |
| 42913 | Diamorphine 10mg powder for solution for injection ampoules (Wockhardt UK Ltd) |
| 47671 | Diamorphine hydrochloride 10mg Injection (Approved Prescription Services Ltd) |
| 47672 | Diamorphine hydrochloride 30mg Injection (Hillcross Pharmaceuticals Ltd) |
| 48158 | Diamorphine 5mg powder for solution for injection vials (Teva UK Ltd) |
| 48259 | Diamorphine 5mg powder for solution for injection ampoules |
| 48413 | Diamorphine 5mg powder for solution for injection vials |
| 48434 | Diamorphine 10mg powder for solution for injection ampoules |
| 48483 | Diamorphine 30mg powder for solution for injection ampoules |
| 48880 | Diamorphine 10mg powder for solution for injection vials |
| 48912 | Diamorphine 30mg powder for solution for injection vials |
| 48913 | Diamorphine 100mg powder for solution for injection ampoules |
| 48953 | Diamorphine 100mg powder for solution for injection vials |
| 53181 | Diamorphine 10mg powder for solution for injection vials (Teva UK Ltd) |
| 53417 | Diamorphine 10mg powder for solution for injection ampoules (Accord Healthcare Ltd) |
| 55221 | Diamorphine 500mg powder for solution for injection ampoules |
| 55724 | Diamorphine 500mg powder for solution for injection vials |
| 58279 | Diamorphine 10mg tablets (A A H Pharmaceuticals Ltd) |
| 58499 | Diamorphine 3mg/5ml oral solution |
| 60721 | Diamorphine 5mg powder for solution for injection ampoules (Accord Healthcare Ltd) |
| 65372 | Diamorphine 5mg powder for solution for injection ampoules (A A H Pharmaceuticals Ltd) |
| 66654 | Diamorphine 15mg powder for solution for injection ampoules |
| 67796 | Diamorphine 10mg powder for solution for injection ampoules (A A H Pharmaceuticals Ltd) |
| 9001 | Diconal tablets (Amdipharm Plc) |
| 12020 | Dipipanone 10mg / Cyclizine 30mg tablets |
| 38301 | Cyclizine 30mg with dipipanone 10mg tablets |
| 21932 | ETHOHEPTALINE CITRATE/ASPIRIN MG TAB |
| 484 | Equagesic Tablet (Wyeth Pharmaceuticals) |
| 11326 | Meprobamate with ethoheptazine citrate and aspirin Tablet |
| 23250 | ASPIRIN /ETHOHEPTAZINE CITRATE /MEPROBAM 250 MG TAB |
| 34073 | Collins 1.5mg/5ml elixir (Collins Elixir Co) |
| 617 | Fentanyl 50microgram/ml Injection |
| 620 | Fentanyl 25micrograms/hour transdermal patches |
| 748 | Durogesic 25micrograms transdermal patches (Janssen-Cilag Ltd) |
| 757 | Fentanyl 50micrograms/hour transdermal patches |
| 4691 | Fentanyl 100micrograms/hour transdermal patches |
| 5048 | Durogesic 50micrograms transdermal patches (Janssen-Cilag Ltd) |
| 5651 | Fentanyl 400microgram lozenges |
| 5657 | Durogesic 75micrograms transdermal patches (Janssen-Cilag Ltd) |
| 5696 | Fentanyl 600microgram lozenges |
| 5697 | Fentanyl 800microgram lozenges |
| 6298 | Fentanyl 75micrograms/hour transdermal patches |
| 7082 | Durogesic DTrans 25micrograms transdermal patches (Janssen-Cilag Ltd) |
| 7107 | Durogesic DTrans 50micrograms transdermal patches (Janssen-Cilag Ltd) |
| 7126 | Fentanyl 12micrograms/hour transdermal patches |
| 7397 | Durogesic DTrans 75micrograms transdermal patches (Janssen-Cilag Ltd) |
| 10922 | Durogesic 100micrograms transdermal patches (Janssen-Cilag Ltd) |
| 11843 | Fentanyl 200microgram lozenges |
| 11982 | Durogesic DTrans 12micrograms transdermal patches (Janssen-Cilag Ltd) |
| 13076 | Actiq 200microgram lozenges with integral oromucosal applicator (Teva UK Ltd) |
| 14900 | Durogesic DTrans 100micrograms transdermal patches (Janssen-Cilag Ltd) |
| 15337 | Fentanyl 1.2mg lozenges |
| 15350 | FENTANYL (10ML) 50 MCG/ML INJ |
| 16618 | Tilofyl 75micrograms/hour transdermal patches (Tillomed Laboratories Ltd) |
| 18174 | Actiq 400microgram lozenges with integral oromucosal applicator (Teva UK Ltd) |
| 22066 | Tilofyl 50micrograms/hour transdermal patches (Tillomed Laboratories Ltd) |
| 24457 | Fentanyl with droperidol 500microgramwith2.5mg/ml Injection |
| 24986 | Fentanyl 1.6mg lozenges |
| 25185 | Sublimaze 50microgram/ml Injection (Janssen-Cilag Ltd) |
| 25199 | Actiq 600microgram lozenges with integral oromucosal applicator (Teva UK Ltd) |
| 26021 | Actiq 1.6mg lozenges with integral oromucosal applicator (Teva UK Ltd) |
| 26908 | Actiq 800microgram lozenges with integral oromucosal applicator (Teva UK Ltd) |
| 28189 | Tilofyl 100micrograms/hour transdermal patches (Tillomed Laboratories Ltd) |
| 29577 | Actiq 1.2mg lozenges with integral oromucosal applicator (Teva UK Ltd) |
| 31053 | Tilofyl 25micrograms/hour transdermal patches (Tillomed Laboratories Ltd) |
| 35330 | Fentanyl 100micrograms/2ml solution for injection ampoules |
| 35853 | Fentanyl 500micrograms/10ml solution for injection ampoules |
| 35968 | Matrifen 25micrograms/hour transdermal patches (Teva UK Ltd) |
| 36040 | Matrifen 100micrograms/hour transdermal patches (Teva UK Ltd) |
| 36211 | Matrifen 50micrograms/hour transdermal patches (Teva UK Ltd) |
| 37719 | Fentalis Reservoir 100micrograms/hour transdermal patches (Sandoz Ltd) |
| 37779 | Fentalis Reservoir 25micrograms/hour transdermal patches (Sandoz Ltd) |
| 37923 | Fentalis Reservoir 50micrograms/hour transdermal patches (Sandoz Ltd) |
| 37928 | Matrifen 12micrograms/hour transdermal patches (Teva UK Ltd) |
| 37954 | Mezolar Matrix 12micrograms/hour transdermal patches (Sandoz Ltd) |
| 37960 | Osmach 50micrograms/hour transdermal patches (Teva UK Ltd) |
| 37968 | Fentanyl 40micrograms/dose transdermal system |
| 38031 | Mezolar Matrix 25micrograms/hour transdermal patches (Sandoz Ltd) |
| 38326 | Mezolar Matrix 75micrograms/hour transdermal patches (Sandoz Ltd) |
| 38351 | Matrifen 75micrograms/hour transdermal patches (Teva UK Ltd) |
| 38365 | Fentalis Reservoir 75micrograms/hour transdermal patches (Sandoz Ltd) |
| 38553 | Mezolar Matrix 100micrograms/hour transdermal patches (Sandoz Ltd) |
| 39084 | Osmach 100micrograms/hour transdermal patches (Ratiopharm UK Ltd) |
| 39180 | Mezolar Matrix 50micrograms/hour transdermal patches (Sandoz Ltd) |
| 39251 | Osmach 25micrograms/hour transdermal patches (Teva UK Ltd) |
| 39469 | Fentanyl 100microgram sublingual tablets sugar free |
| 39518 | Abstral 100microgram sublingual tablets (Kyowa Kirin Ltd) |
| 39590 | Fentanyl 200microgram sublingual tablets sugar free |
| 39723 | Fentanyl 100microgram buccal tablets sugar free |
| 39746 | Effentora 100microgram buccal tablets (Teva UK Ltd) |
| 39756 | Abstral 200microgram sublingual tablets (Kyowa Kirin Ltd) |
| 39799 | Abstral 800microgram sublingual tablets (Kyowa Kirin Ltd) |
| 39929 | Effentora 200microgram buccal tablets (Teva UK Ltd) |
| 39987 | Abstral 400microgram sublingual tablets (Kyowa Kirin Ltd) |
| 40018 | Fentanyl 200microgram buccal tablets sugar free |
| 40098 | Fentanyl 400microgram sublingual tablets sugar free |
| 40128 | Effentora 400microgram buccal tablets (Teva UK Ltd) |
| 40434 | Effentora 600microgram buccal tablets (Teva UK Ltd) |
| 40508 | Abstral 600microgram sublingual tablets (Kyowa Kirin Ltd) |
| 40576 | Fentanyl 400microgram buccal tablets sugar free |
| 40940 | Fentanyl 300microgram sublingual tablets sugar free |
| 40957 | Effentora 800microgram buccal tablets (Teva UK Ltd) |
| 41135 | Fentanyl 50micrograms/dose nasal spray |
| 41161 | Osmach 75micrograms/hour transdermal patches (Teva UK Ltd) |
| 41286 | Abstral 300microgram sublingual tablets (Kyowa Kirin Ltd) |
| 41348 | Fentanyl 600microgram sublingual tablets sugar free |
| 42021 | Victanyl 100micrograms/hour transdermal patches (Actavis UK Ltd) |
| 42399 | Fentanyl 800microgram sublingual tablets sugar free |
| 42538 | Fentanyl 600microgram buccal tablets sugar free |
| 42576 | Victanyl 75micrograms/hour transdermal patches (Actavis UK Ltd) |
| 42590 | Osmanil 75micrograms/hour transdermal patches (Zentiva) |
| 42591 | Osmanil 25micrograms/hour transdermal patches (Zentiva) |
| 43089 | Instanyl 100micrograms/dose nasal spray (Takeda UK Ltd) |
| 43152 | Osmanil 50micrograms/hour transdermal patches (Zentiva) |
| 43617 | Instanyl 200micrograms/dose nasal spray (Takeda UK Ltd) |
| 43812 | Sublimaze 500micrograms/10ml solution for injection ampoules (Piramal Critical Care Ltd) |
| 44487 | Osmanil 12micrograms/hour transdermal patches (Zentiva) |
| 44837 | Victanyl 50micrograms/hour transdermal patches (Actavis UK Ltd) |
| 45092 | Fentanyl 200micrograms/dose nasal spray |
| 45439 | PecFent 100micrograms/dose nasal spray (Kyowa Kirin Ltd) |
| 45460 | Osmanil 100micrograms/hour transdermal patches (Zentiva) |
| 45549 | Victanyl 25micrograms/hour transdermal patches (Actavis UK Ltd) |
| 45598 | Instanyl 50micrograms/dose nasal spray (Takeda UK Ltd) |
| 45894 | Fentanyl 100micrograms/dose nasal spray |
| 46354 | PecFent 400micrograms/dose nasal spray (Kyowa Kirin Ltd) |
| 46555 | Fentanyl 800microgram buccal tablets sugar free |
| 46559 | Fencino 12micrograms/hour transdermal patches (Ethypharm UK Ltd) |
| 46560 | Fencino 50micrograms/hour transdermal patches (Ethypharm UK Ltd) |
| 46657 | Fencino 75micrograms/hour transdermal patches (Ethypharm UK Ltd) |
| 46658 | Fencino 100micrograms/hour transdermal patches (Ethypharm UK Ltd) |
| 46733 | Fencino 25micrograms/hour transdermal patches (Ethypharm UK Ltd) |
| 47413 | Fentanyl 75micrograms/hr Transdermal patch (Sandoz Ltd) |
| 47759 | Fentanyl 400micrograms/dose nasal spray |
| 48183 | Fentanyl 100micrograms/2ml solution for injection ampoules (Martindale Pharmaceuticals Ltd) |
| 48571 | Durogesic DTrans 50micrograms transdermal patches (Waymade Healthcare Plc) |
| 50671 | Fentanyl 12micrograms/hour transdermal patches (A A H Pharmaceuticals Ltd) |
| 50929 | Durogesic DTrans 12micrograms transdermal patches (Mawdsley-Brooks & Company Ltd) |
| 51235 | Fentanyl 25micrograms/hour transdermal patches (Phoenix Healthcare Distribution Ltd) |
| 52178 | Fentanyl 100micrograms/2ml solution for injection ampoules (AMCo) |
| 54979 | Fentanyl 50micrograms/hour transdermal patches (A A H Pharmaceuticals Ltd) |
| 55752 | Fentanyl 100micrograms/hour transdermal patches (Phoenix Healthcare Distribution Ltd) |
| 56670 | Fentanyl 25micrograms/hour transdermal patches (Sigma Pharmaceuticals Plc) |
| 59057 | Fentanyl 400microgram buccal films sugar free |
| 59443 | Fentanyl 200microgram buccal films sugar free |
| 59482 | Fentanyl 37.5microgram/hour transdermal patches |
| 59490 | Mezolar Matrix 37.5microgram/hour transdermal patches (Sandoz Ltd) |
| 59678 | Fentanyl 100micrograms/2ml solution for injection ampoules (A A H Pharmaceuticals Ltd) |
| 60477 | Fentanyl 25micrograms/hour transdermal patches (A A H Pharmaceuticals Ltd) |
| 60766 | Fentanyl 25micrograms/hour transdermal patches (Waymade Healthcare Plc) |
| 61086 | Opiodur 12micrograms/hour transdermal patches (Pfizer Ltd) |
| 61156 | Fentanyl 12micrograms/hour transdermal patches (Waymade Healthcare Plc) |
| 61305 | Mylafent 75micrograms/hour transdermal patches (Mylan) |
| 61708 | Recivit 267microgram sublingual tablets (Grunenthal Ltd) |
| 63139 | Fentanyl 12micrograms/hour transdermal patches (Phoenix Healthcare Distribution Ltd) |
| 63340 | Fentanyl 133microgram sublingual tablets sugar free |
| 63398 | Fentanyl 2.5mg/50ml solution for infusion vials |
| 65168 | Mylafent 12micrograms/hour transdermal patches (Mylan) |
| 65359 | Mylafent 50micrograms/hour transdermal patches (Mylan) |
| 65437 | Fentanyl 100micrograms/hour transdermal patches (A A H Pharmaceuticals Ltd) |
| 65646 | Fentanyl 267microgram sublingual tablets sugar free |
| 67258 | Durogesic DTrans 25micrograms transdermal patches (Waymade Healthcare Plc) |
| 67425 | Yemex 25micrograms/hour transdermal patches (Sandoz Ltd) |
| 67474 | Opiodur 50micrograms/hour transdermal patches (Pfizer Ltd) |
| 67766 | Fentanyl 75micrograms/hour transdermal patches (Phoenix Healthcare Distribution Ltd) |
| 67830 | Fentanyl 12micrograms/hour transdermal patches (DE Pharmaceuticals) |
| 68209 | Mylafent 100micrograms/hour transdermal patches (Mylan) |
| 69023 | Yemex 100micrograms/hour transdermal patches (Sandoz Ltd) |
| 70376 | Yemex 50micrograms/hour transdermal patches (Sandoz Ltd) |
| 70810 | Yemex 12micrograms/hour transdermal patches (Sandoz Ltd) |
| 70988 | Fentanyl 50micrograms/hour transdermal patches (Phoenix Healthcare Distribution Ltd) |
| 72342 | Yemex 75micrograms/hour transdermal patches (Sandoz Ltd) |
| 2952 | Methadone 1mg/ml oral solution |
| 5211 | Methadone 2mg/5ml linctus |
| 5322 | Physeptone 5mg tablets (Martindale Pharmaceuticals Ltd) |
| 6441 | Methadone 5mg tablets |
| 9728 | Methadone 1mg/ml oral solution sugar free |
| 11722 | Methadone 10mg/ml oral solution sugar free |
| 12132 | METHADONE 5 MG/ML INJ |
| 14086 | Methadone 10mg/ml Injection |
| 15449 | METHADONE 40 MG SUP |
| 17671 | Methadone 50mg/1ml solution for injection ampoules |
| 17896 | Physeptone 10mg/ml Injection (Martindale Pharmaceuticals Ltd) |
| 21337 | METHADONE 15 MG SUP |
| 21562 | Physeptone 1mg/ml oral solution sugar free (Martindale Pharmaceuticals Ltd) |
| 23158 | Methadone 20mg/ml oral solution sugar free |
| 23769 | METHADONE 25 MG SUP |
| 23947 | METHADONE 30 MG SUP |
| 23948 | METHADONE 20 MG SUP |
| 24343 | Methadose 10mg/ml oral solution concentrate (Rosemont Pharmaceuticals Ltd) |
| 24361 | Methadose 20mg/ml oral solution concentrate (Rosemont Pharmaceuticals Ltd) |
| 24440 | Methodex 1mg/ml Mixture (Link Pharmaceuticals Ltd) |
| 24446 | METHADONE 100 MG SUP |
| 24584 | Methadone 50mg/2ml solution for injection ampoules |
| 25046 | Methadone diluent Liquid |
| 26277 | Physeptone 1mg/ml mixture (Martindale Pharmaceuticals Ltd) |
| 26801 | Methadone colourant for Liquid |
| 28328 | Metharose 1mg/ml oral solution sugar free (Rosemont Pharmaceuticals Ltd) |
| 28861 | METHADONE 50 MG SUP |
| 29304 | Physeptone 10mg/1ml solution for injection ampoules (Martindale Pharmaceuticals Ltd) |
| 29769 | Methadone 2mg/5ml Oral solution (Martindale Pharmaceuticals Ltd) |
| 29911 | Methadone 2mg/5ml linctus (Thornton & Ross Ltd) |
| 30531 | Methadone 1mg/ml oral solution sugar free (Rosemont Pharmaceuticals Ltd) |
| 32237 | Methex 1mg/ml Mixture (Generics (UK) Ltd) |
| 32526 | METHADONE GREEN S/F |
| 33068 | Methadone 10mg/1ml solution for injection ampoules |
| 33475 | Methadone 35mg/ml Injection |
| 33832 | Methadone 1mg/ml oral solution (Martindale Pharmaceuticals Ltd) |
| 35506 | Methadone 20mg/2ml solution for injection ampoules |
| 36436 | Methadone 50mg/5ml solution for injection ampoules |
| 36994 | Methadone 5mg/ml oral solution |
| 37507 | Physeptone 50mg/1ml solution for injection ampoules (Martindale Pharmaceuticals Ltd) |
| 37518 | Methadone 35mg/3.5ml solution for injection ampoules |
| 39437 | Eptadone 1mg/ml oral solution (Dee Pharmaceuticals Ltd) |
| 41608 | Methadone 1mg/ml oral solution (Rosemont Pharmaceuticals Ltd) |
| 41720 | Methadone 1mg/ml Mixture (Macarthy Medical Ltd) |
| 43260 | Methadone Oral solution |
| 43766 | Eptadone 5mg/ml oral solution (Dee Pharmaceuticals Ltd) |
| 46578 | Physeptone 20mg/2ml solution for injection ampoules (Martindale Pharmaceuticals Ltd) |
| 47706 | Methadone 1mg/ml oral solution sugar free (Martindale Pharmaceuticals Ltd) |
| 55825 | Methadone 1mg/ml oral solution sugar free (Thornton & Ross Ltd) |
| 59295 | Methadone 100mg capsules |
| 60944 | Methadone 5mg capsules |
| 62708 | Methadone hydrochloride powder |
| 63077 | Methadone 1mg/5ml oral suspension |
| 64463 | Methadone 30mg capsules |
| 66921 | Methadone 1mg/ml oral solution sugar free (Waymade Healthcare Plc) |
| 67342 | Methadone 50mg/1ml solution for injection ampoules (Alliance Healthcare (Distribution) Ltd) |
| 68959 | Methadone 20mg/5ml oral solution |
| 69053 | Pinadone methadone 1mg/ml Oral solution sugar free (Pinewood Healthcare) |
| 70267 | Methadone 15mg/5ml oral solution |
| 148 | MORPHINE 60 MG SUP |
| 354 | Morphine sulfate 100mg/50ml solution for infusion vials |
| 458 | Morphine 15mg Suppository |
| 495 | MST Continus 10mg tablets (Napp Pharmaceuticals Ltd) |
| 607 | MST Continus Suspension 20mg granules sachets (Napp Pharmaceuticals Ltd) |
| 655 | Morphine sulfate 10mg/5ml oral solution unit dose vials sugar free |
| 659 | Morphine 30mg Suppository |
| 715 | Morphine sulphate 1mg/ml Injection |
| 824 | Morphine hydrochloride powder |
| 1503 | Oramorph 10mg/5ml oral solution (Boehringer Ingelheim Ltd) |
| 2425 | Kaolin and Morphine mixture |
| 2957 | MST Continus 30mg tablets (Napp Pharmaceuticals Ltd) |
| 2997 | Oramorph sr 10mg Tablet (Boehringer Ingelheim Ltd) |
| 3919 | Sevredol 10mg tablets (Napp Pharmaceuticals Ltd) |
| 4266 | Morphine 10mg tablets |
| 4280 | MST Continus 5mg tablets (Napp Pharmaceuticals Ltd) |
| 4476 | MST Continus Suspension 60mg granules sachets (Napp Pharmaceuticals Ltd) |
| 4477 | MST Continus 60mg tablets (Napp Pharmaceuticals Ltd) |
| 4693 | Oramorph 10mg/5ml oral solution unit dose vials (Boehringer Ingelheim Ltd) |
| 5555 | Sevredol 10mg/5ml oral solution (Napp Pharmaceuticals Ltd) |
| 5563 | Morphine sulphate 12 20mg Modified-release capsule |
| 5652 | Morphine sulphate 12 50mg Modified-release capsule |
| 5664 | Morphine hydrochloride 15mg suppositories |
| 5681 | Morphine 10mg modified-release tablets |
| 5714 | MST Continus 15mg tablets (Napp Pharmaceuticals Ltd) |
| 5777 | Diocalm Dual Action chewable tablets (SSL International Plc) |
| 5833 | Cyclimorph 10 solution for injection 1ml ampoules (AMCo) |
| 5840 | Morphine sulfate 10mg/5ml oral solution |
| 5991 | MST Continus 100mg tablets (Napp Pharmaceuticals Ltd) |
| 6002 | Morphine 10mg modified-release capsules |
| 6232 | Sevredol 20mg tablets (Napp Pharmaceuticals Ltd) |
| 6269 | Morphine sulfate 20mg/ml oral solution sugar free |
| 6366 | Sevredol 50mg tablets (Napp Pharmaceuticals Ltd) |
| 6736 | Morphine 20mg modified-release granules sachets sugar free |
| 6892 | Morphine sulphate 10mg/ml Injection |
| 7181 | J Collis Browne's tablets (Thornton & Ross Ltd) |
| 7197 | Morphine sulphate 12 30mg Modified-release capsule |
| 7517 | Morphine sulfate 15mg suppositories |
| 7729 | Morphine hcl Oral solution (Thornton and Ross Ltd) |
| 7801 | MORPHINE SULPHATE SR 30 MG TAB |
| 7872 | MORPHINE S/R 64 MG INJ |
| 7875 | Morphine 30mg modified-release tablets |
| 8039 | MST Continus 200mg tablets (Napp Pharmaceuticals Ltd) |
| 8075 | Morphine sulphate 30mg/ml Injection |
| 8220 | MORPHINE ANHYDROUS 8.4 MG ELI |
| 8420 | MORPHINE SULPHATE 5 MG INJ |
| 8740 | Morphine hydrochloride 30mg suppositories |
| 8766 | MORPHINE HCl 30 MG INJ |
| 8822 | Morphine 60mg modified-release tablets |
| 8867 | MORPHINE SULPHATE CR 30 MG TAB |
| 8876 | Oramorph 20mg/ml concentrated oral solution (Boehringer Ingelheim Ltd) |
| 8959 | MORPHINE SULPHATE 15 MG TAB |
| 9012 | Cyclimorph 15 solution for injection 1ml ampoules (AMCo) |
| 9137 | Morphine 20mg tablets |
| 9183 | Morphine 100mg modified-release tablets |
| 9337 | MXL 30mg capsules (Napp Pharmaceuticals Ltd) |
| 9342 | MXL 60mg capsules (Napp Pharmaceuticals Ltd) |
| 9371 | MXL 120mg capsules (Napp Pharmaceuticals Ltd) |
| 9381 | MXL 90mg capsules (Napp Pharmaceuticals Ltd) |
| 9484 | Morphine sulphate 24 60mg Modified-release capsule |
| 9557 | Morphine 15mg modified-release tablets |
| 9602 | Morphine 5mg modified-release tablets |
| 9672 | Morphine 100mg modified-release granules sachets sugar free |
| 9960 | Morphine sulphate 12 60mg Modified-release capsule |
| 10239 | MXL 150mg capsules (Napp Pharmaceuticals Ltd) |
| 10631 | Morphine 10mg/ml Tincture |
| 10730 | Sevredol 10mg Suppository (Napp Pharmaceuticals Ltd) |
| 10749 | Ipecacuanha and morphine Mixture |
| 10907 | MORPHINE SULPHATE CR 15 MG TAB |
| 11342 | Oramorph 30mg/5ml oral solution unit dose vials (Boehringer Ingelheim Ltd) |
| 11698 | Morphine sulphate 24 30mg Modified-release capsule |
| 11755 | Morcap SR 20mg capsules (Hospira UK Ltd) |
| 11838 | Morphine 200mg modified-release tablets |
| 11971 | Morphine and Cocaine elixir |
| 12011 | MORPHINE SULPHATE CR 60 MG TAB |
| 12219 | Morphine sulfate 15mg/1ml solution for injection ampoules |
| 12508 | MORPHINE SULPHATE CR 100 MG TAB |
| 12583 | MORPHINE SULPHATE CR 5 MG TAB |
| 12591 | Morphine 60mg modified-release granules sachets sugar free |
| 12602 | Morphine sulfate 10mg suppositories |
| 12604 | MST Continus Suspension 100mg granules sachets (Napp Pharmaceuticals Ltd) |
| 12608 | MORPHINE SULPHATE CR 200 MG TAB |
| 12889 | Oramorph 100mg/5ml oral solution unit dose vials (Boehringer Ingelheim Ltd) |
| 12900 | MST Continus Suspension 30mg granules sachets (Napp Pharmaceuticals Ltd) |
| 13114 | Zomorph 10mg modified-release capsules (Ethypharm UK Ltd) |
| 13117 | Zomorph 30mg modified-release capsules (Ethypharm UK Ltd) |
| 13172 | Morphine sulfate 20mg/1ml solution for injection ampoules |
| 13225 | Morphine sulfate 30mg/1ml solution for injection ampoules |
| 13280 | Morphine sulfate 60mg/2ml solution for injection ampoules |
| 13423 | MORPHINE SULPHATE BP GRANULES 30 MG |
| 13588 | MORPHINE SULPHATE susp C/R 30 MG |
| 13711 | Morcap sr 20mg Modified-release capsule (Faulding Pharmaceuticals (Dbl)) |
| 13995 | Morphine sulphate 24 200mg Modified-release capsule |
| 13997 | Morphine sulphate 100mg Modified-release capsule |
| 14050 | Morphine sulphate 12 100mg Modified-release capsule |
| 14063 | Zomorph 100mg modified-release capsules (Ethypharm UK Ltd) |
| 14156 | Morphine sulfate 30mg/5ml oral solution unit dose vials sugar free |
| 14226 | Morphine 30mg modified-release granules sachets sugar free |
| 15064 | MORPHINE HCL 10MG/CHLOROFORM WATER > 5ML SOL |
| 15781 | Morphine sulphate 24 90mg Modified-release capsule |
| 15815 | Morphine 50mg tablets |
| 15950 | Zomorph 200mg modified-release capsules (Ethypharm UK Ltd) |
| 15964 | Zomorph 60mg modified-release capsules (Ethypharm UK Ltd) |
| 16189 | Morphine sulphate 10mg/ml Injection |
| 16273 | Oramorph sr 30mg Tablet (Boehringer Ingelheim Ltd) |
| 16335 | Morphine tartrate 10mg/1ml / Cyclizine tartrate 50mg/1ml solution for injection ampoules |
| 17092 | MORPHINE HCl 10 MG INJ |
| 17271 | MORPHINE SULPHATE 5 MG SUP |
| 17398 | Morphine tartrate 15mg/1ml / Cyclizine tartrate 50mg/1ml solution for injection ampoules |
| 17490 | Cyclizine tartrate with morphine tartrate 50mg+15mg/ml injection |
| 17825 | MORPHINE HCL 5MG/CHLOROFORM WATER TO 5ML SOL |
| 17893 | Oramorph sr 60mg Tablet (Boehringer Ingelheim Ltd) |
| 17936 | MXL 200mg capsules (Napp Pharmaceuticals Ltd) |
| 17943 | Sevredol 20mg/ml concentrated oral solution (Napp Pharmaceuticals Ltd) |
| 18166 | Morphine sulphate 12 200mg Modified-release capsule |
| 18468 | J Collis Browne's mixture (Thornton & Ross Ltd) |
| 18626 | MORPHINE SULPHATE 200 MG TAB |
| 18639 | MORPHINE SULPHATE SR 100 MG TAB |
| 18656 | SRM-RHOTARD 10mg Modified-release tablet (Pharmacia Ltd) |
| 18700 | SRM-RHOTARD 30mg Modified-release tablet (Pharmacia Ltd) |
| 18727 | MORPHINE SULPHATE BP GRANULES 20 MG |
| 18734 | Oramorph sr 100mg Tablet (Boehringer Ingelheim Ltd) |
| 18801 | Morcap SR 100mg capsules (Hospira UK Ltd) |
| 18881 | Morphgesic SR 10mg tablets (AMCo) |
| 19092 | Morcap sr 50mg Modified-release capsule (Faulding Pharmaceuticals (Dbl)) |
| 19291 | Morphine sulfate 100mg/5ml oral solution unit dose vials sugar free |
| 19317 | Omnopon 10mg Tablet (Roche Products Ltd) |
| 19449 | Morphgesic SR 30mg tablets (AMCo) |
| 19471 | Morphgesic SR 60mg tablets (AMCo) |
| 19477 | Morphgesic SR 100mg tablets (AMCo) |
| 19738 | MORPHINE,COCAINE & CHLORPROMAZINE MIX |
| 19764 | Papaveretum 10mg tablet |
| 20005 | Morphine sulfate 10mg/10ml solution for injection pre-filled syringes |
| 20008 | MORPHINE SULPHATE 20 MG CAP |
| 20219 | MORPHINE SULPHATE 120 MG CAP |
| 20783 | Morphine sulfate 30mg suppositories |
| 20815 | Morphine sulfate 20mg suppositories |
| 21972 | MORPHINE ANHYDROUS |
| 22024 | Rhotard Morphine SR 10mg tablets (Sovereign Medical Ltd) |
| 22026 | Rhotard Morphine SR 30mg tablets (Sovereign Medical Ltd) |
| 22051 | Morphine sulphate Crystals |
| 22571 | Morphine sulphate and atropine 10mg + 600microgram/ml Injection |
| 22622 | MORPHINE TARTRATE/CYCLIZINE TARTRATE 15 MG INJ |
| 22690 | Morphine sulphate 24 120mg Modified-release capsule |
| 22756 | Filnarine SR 30mg tablets (Teva UK Ltd) |
| 23060 | MST Continus Suspension 200mg granules sachets (Napp Pharmaceuticals Ltd) |
| 23063 | Morphine 1mg/5ml / Peppermint oil 1.5microlitres/5ml oral solution |
| 23128 | AMMONIUM CHLOR.& MORPHINE DOUBLE STRENGT MIX |
| 23581 | Morphine hcl light kaolin, belladonna and aluminium hydroxide chewable tablet |
| 23775 | MORPHINE SULPHATE 60 MG INJ |
| 23777 | MORPHINE SULPHATE 100 MG SUP |
| 24424 | Morphine hcl and activated attapulgite and attapulgite Tablet |
| 24453 | Morcap sr 100mg Modified-release capsule (Faulding Pharmaceuticals (Dbl)) |
| 24808 | MORPHINE SULPHATE SUP |
| 24816 | MORPHINE ANHYDROUS 8.4 MG INJ |
| 24830 | Morphine sulphate 20mg/ml Injection |
| 25234 | Chloroform and Morphine tincture |
| 25316 | Cyclizine tartrate with morphine tartrate 50mg+10mg/ml injection |
| 25481 | Nepenthe 8.4mg/ml Injection (Celltech Pharma Europe Ltd) |
| 25503 | AMMONIUM CHLORIDE & MORPHINE MIX |
| 25650 | MORPHINE SULPHATE 60 MG SUP |
| 26144 | Morphine with ipecacuanha Mixture |
| 26283 | Filnarine SR 10mg tablets (Teva UK Ltd) |
| 26284 | Filnarine SR 100mg tablets (Teva UK Ltd) |
| 26805 | MORPHINE SULPHATE 50 MG SUP |
| 27058 | Filnarine SR 60mg tablets (Teva UK Ltd) |
| 27298 | MORPHINE SULPHATE EPIDURAL 2 MG INJ |
| 27338 | Morphine hcl and light kaolin and calcium carbonate Tablet |
| 27436 | MORPHINE SULPHATE 90 MG CAP |
| 27441 | Opazimes chewable tablets (Strides Shasun (UK) Ltd) |
| 27749 | Morphine sulphate 24 150mg Modified-release capsule |
| 28421 | Moraxen 35mg Rectal tampon (Schwarz Pharma Ltd) |
| 28503 | MORPHINE SULPHATE 100 MG CAP |
| 28837 | MORPHINE SULPHATE 200 MG CAP |
| 29019 | Bismuth with Morphine mixture |
| 29020 | Morphine 200mg modified-release granules sachets sugar free |
| 29379 | CHALK AROMAT & MORPHINE MIX |
| 29898 | Morphine sulfate powder |
| 29970 | Morphine hcl and kaolin and belladonna Tablet |
| 30049 | Morphine sulphate rapiject 1mg/ml Injection (International Medication Systems (UK) Ltd) |
| 30252 | Morphine 8.4mg/ml elixir |
| 30597 | Ipecacuanha and Morphine mixture BP 1980 |
| 31044 | MORPHINE SULPHATE 150 MG SUP |
| 31407 | IPECACUANHA & MORPHINE CONC 1-4 MIX |
| 31599 | MORPHINE SULPHATE 4 MG INJ |
| 31650 | Morphine sulfate 30mg/30ml solution for infusion vials |
| 32357 | MORPHINE SULPHATE 300 MG SUP |
| 32459 | MORPHINE SULPHATE 50 MG CAP |
| 32460 | MORPHINE SULPHATE 150 MG CAP |
| 32520 | Opium tincture |
| 34477 | Morphine sulfate 10mg/5ml oral solution (Martindale Pharmaceuticals Ltd) |
| 34771 | Morphine sulphate 30mg/ml Injection (Celltech Pharma Europe Ltd) |
| 35093 | Morphine sulfate 50mg/50ml solution for infusion vials |
| 40563 | Morphine 30mg modified-release capsules |
| 41668 | Morphine hcl 15mg Suppository (Martindale Pharmaceuticals Ltd) |
| 41673 | Morphine sulphate 10mg Suppository (Aurum Pharmaceuticals Ltd) |
| 41674 | Morphine sulphate 15mg Suppository (Celltech Pharma Europe Ltd) |
| 41974 | Morphine sulfate 10mg/1ml solution for injection ampoules (Martindale Pharmaceuticals Ltd) |
| 42380 | Morphine sulphate 10mg Modified-release capsule |
| 43315 | Morphine sulfate 10mg/1ml suspension for injection vials |
| 43652 | Morphine 100mg modified-release capsules |
| 43657 | Morphine 200mg modified-release capsules |
| 45736 | Morphine 60mg modified-release capsules |
| 47154 | Filnarine SR 200mg tablets (Teva UK Ltd) |
| 47555 | Morphine sulfate 10mg/10ml solution for injection Minijet pre-filled syringes (UCB Pharma Ltd) |
| 47753 | Morphine 90mg modified-release capsules |
| 47867 | Morphine 150mg modified-release capsules |
| 47949 | Morphine 120mg modified-release capsules |
| 47985 | M-eslon 10mg Capsule (Trinity Pharmaceuticals Ltd) |
| 48604 | Morphine sulfate 10mg/1ml solution for injection ampoules |
| 49976 | Morphine sulfate 10mg/1ml solution for injection ampoules (A A H Pharmaceuticals Ltd) |
| 50513 | Morphine sulfate 10mg/1ml solution for injection ampoules (UCB Pharma Ltd) |
| 53106 | Morphine sulfate 5mg/5ml solution for injection ampoules |
| 53273 | Morphine hydrochloride 10mg/5ml oral solution (Special Order) |
| 53639 | Morphine 10mg modified-release tablets (Sigma Pharmaceuticals Plc) |
| 53918 | Morphine sulfate 10mg/1ml solution for injection ampoules (Hameln Pharmaceuticals Ltd) |
| 54017 | Morphine sulphate Capsule |
| 54406 | Morphine sulfate 30mg suppositories (Martindale Pharmaceuticals Ltd) |
| 54520 | Morphine sulphate Oral solution |
| 55052 | Morcap SR 50mg capsules (Hospira UK Ltd) |
| 55206 | Morphine 20mg modified-release capsules |
| 55365 | Morphine sulfate 50mg/50ml solution for infusion vials (A A H Pharmaceuticals Ltd) |
| 55832 | Morphine sulphate 15mg/ml Injection (Celltech Pharma Europe Ltd) |
| 56202 | Morphine sulphate Injection |
| 56329 | Morphine sulfate 10mg/2ml solution for injection ampoules |
| 56544 | Morphine sulfate 50mg/50ml solution for infusion vials (Martindale Pharmaceuticals Ltd) |
| 56788 | Morphine sulfate 10mg/5ml oral solution (A A H Pharmaceuticals Ltd) |
| 57623 | Morphine sulfate 15mg/1ml solution for injection ampoules (UCB Pharma Ltd) |
| 57750 | Morphine sulfate 15mg/1ml solution for injection ampoules (Wockhardt UK Ltd) |
| 58215 | Kaolin and Morphine mixture (Thornton & Ross Ltd) |
| 58290 | Morphine sulfate 10mg suppositories (Martindale Pharmaceuticals Ltd) |
| 58710 | Morphine sulfate 1mg/1ml solution for injection ampoules |
| 58836 | Morphine sulfate 5mg/1ml solution for injection ampoules |
| 58879 | Morphine hydrochloride 10mg/5ml oral solution |
| 59584 | Morphine sulfate 10mg/5ml oral solution (Alliance Healthcare (Distribution) Ltd) |
| 60082 | Morphine sulfate 5mg/10ml solution for injection ampoules |
| 60507 | Morphine 0.1% in Intrasite gel |
| 60518 | Morphine sulfate 500micrograms/5ml oral solution |
| 60950 | Morphine sulfate 5mg/5ml oral solution |
| 61241 | Morphine sulfate 50mg/50ml solution for infusion vials (Alliance Healthcare (Distribution) Ltd) |
| 61400 | Morphine sulfate 30mg/1ml solution for injection ampoules (A A H Pharmaceuticals Ltd) |
| 61423 | Morphine 30mg modified-release tablets (Sigma Pharmaceuticals Plc) |
| 61506 | Morphine sulfate 10mg/1ml solution for injection ampoules (Wockhardt UK Ltd) |
| 61584 | Morphine sulfate 50mg/50ml solution for infusion vials (Torbay Pharmaceuticals) |
| 61744 | Morphine sulfate 100mg/50ml solution for infusion vials (A A H Pharmaceuticals Ltd) |
| 61918 | Morphine sulfate 20mg/2ml solution for injection ampoules |
| 61942 | Morphine sulfate 2.5mg/5ml solution for injection ampoules |
| 62689 | Morphine hydrochloride 1mg/1ml solution for injection ampoules |
| 63423 | Morphine sulfate 10mg/1ml solution for injection ampoules (DE Pharmaceuticals) |
| 63593 | Morphine sulfate 10mg/5ml oral solution (Actavis UK Ltd) |
| 63817 | Morphine hcl Powder (Celltech Pharma Europe Ltd) |
| 64417 | Morphine sulfate 2mg/5ml oral solution |
| 64780 | Morphine sulfate 50mg/1ml solution for injection ampoules |
| 64781 | Morphine sulfate 50mg/5ml solution for injection ampoules |
| 64860 | Morphine sulfate 10mg/10ml solution for injection ampoules |
| 66336 | Morphine sulphate 10mg/ml Injection (Celltech Pharma Europe Ltd) |
| 66815 | Morphine sulfate 10mg/10ml solution for injection ampoules (Hameln Pharmaceuticals Ltd) |
| 68712 | Morphine hydrochloride 100mg/5ml oral solution |
| 70274 | Morphine sulfate 5mg/5ml solution for injection ampoules (Torbay Pharmaceuticals) |
| 71171 | Morphine sulfate 10mg/10ml solution for injection ampoules (Torbay Pharmaceuticals) |
| 71462 | Morphine sulfate 1mg/1ml solution for injection ampoules (Torbay Pharmaceuticals) |
| 72225 | Morphine sulfate 10mg/5ml oral solution (Wockhardt UK Ltd) |
| 72255 | Morphine 0.2% in Intrasite gel |
| 72457 | Sevredol 20mg Suppository (Napp Pharmaceuticals Ltd) |
| 5585 | Oxycodone 10mg capsules |
| 5599 | OxyContin 10mg modified-release tablets (Napp Pharmaceuticals Ltd) |
| 5843 | Oxycodone 10mg modified-release tablets |
| 6414 | Oxycodone hydrochloride 10mg/ml injection |
| 6557 | OxyNorm 5mg capsules (Napp Pharmaceuticals Ltd) |
| 6608 | Oxycodone 20mg modified-release tablets |
| 6609 | Oxycodone 5mg/5ml oral solution sugar free |
| 6708 | Oxycodone 40mg modified-release tablets |
| 6769 | Oxycodone 5mg modified-release tablets |
| 6790 | Oxycodone 5mg capsules |
| 6948 | Oxycodone 80mg modified-release tablets |
| 7167 | OxyContin 5mg modified-release tablets (Napp Pharmaceuticals Ltd) |
| 7275 | Oxycodone 20mg capsules |
| 7372 | OxyNorm 20mg capsules (Napp Pharmaceuticals Ltd) |
| 7389 | OxyContin 20mg modified-release tablets (Napp Pharmaceuticals Ltd) |
| 7406 | OxyNorm 10mg/ml concentrate oral solution (Napp Pharmaceuticals Ltd) |
| 9874 | OxyNorm liquid 5mg/5ml oral solution (Napp Pharmaceuticals Ltd) |
| 9927 | OxyContin 40mg modified-release tablets (Napp Pharmaceuticals Ltd) |
| 9928 | Oxynorm 10mg/ml Solution for injection (Napp Pharmaceuticals Ltd) |
| 9973 | OxyNorm 10mg capsules (Napp Pharmaceuticals Ltd) |
| 10021 | OxyContin 80mg modified-release tablets (Napp Pharmaceuticals Ltd) |
| 10866 | Oxycodone hydrochloride 30mg suppositories |
| 11405 | Oxycodone 10mg/ml oral solution sugar free |
| 27548 | OXYCODONE |
| 32425 | OxyNorm 10mg/1ml solution for injection ampoules (Napp Pharmaceuticals Ltd) |
| 35038 | OxyNorm 20mg/2ml solution for injection ampoules (Napp Pharmaceuticals Ltd) |
| 35085 | Oxycodone 20mg/2ml solution for injection ampoules |
| 35341 | Oxycodone 10mg/1ml solution for injection ampoules |
| 39475 | Oxycodone 10mg / Naloxone 5mg modified-release tablets |
| 39477 | Targinact 10mg/5mg modified-release tablets (Napp Pharmaceuticals Ltd) |
| 39478 | Targinact 20mg/10mg modified-release tablets (Napp Pharmaceuticals Ltd) |
| 39498 | Oxycodone 20mg / Naloxone 10mg modified-release tablets |
| 40616 | Oxycodone 5mg / Naloxone 2.5mg modified-release tablets |
| 40645 | Targinact 5mg/2.5mg modified-release tablets (Napp Pharmaceuticals Ltd) |
| 40688 | Oxycodone 50mg/1ml solution for injection ampoules |
| 40752 | OxyNorm 50mg/1ml solution for injection ampoules (Napp Pharmaceuticals Ltd) |
| 40785 | Oxycodone 40mg / Naloxone 20mg modified-release tablets |
| 40961 | Targinact 40mg/20mg modified-release tablets (Napp Pharmaceuticals Ltd) |
| 45745 | OxyContin 30mg modified-release tablets (Napp Pharmaceuticals Ltd) |
| 45766 | OxyContin 60mg modified-release tablets (Napp Pharmaceuticals Ltd) |
| 45788 | OxyContin 15mg modified-release tablets (Napp Pharmaceuticals Ltd) |
| 45790 | Oxycodone 15mg modified-release tablets |
| 45827 | Oxycodone 30mg modified-release tablets |
| 45830 | OxyContin 120mg modified-release tablets (Napp Pharmaceuticals Ltd) |
| 45929 | Oxycodone 60mg modified-release tablets |
| 46187 | Oxycodone 120mg modified-release tablets |
| 49742 | OxyContin 5mg modified-release tablets (DE Pharmaceuticals) |
| 49787 | OxyContin 20mg modified-release tablets (Lexon (UK) Ltd) |
| 49791 | OxyNorm 20mg/2ml solution for injection ampoules (Waymade Healthcare Plc) |
| 49940 | OxyNorm 5mg capsules (Lexon (UK) Ltd) |
| 50095 | OxyNorm 5mg capsules (DE Pharmaceuticals) |
| 50726 | OxyNorm 10mg/1ml solution for injection ampoules (Waymade Healthcare Plc) |
| 50733 | OxyContin 10mg modified-release tablets (Mawdsley-Brooks & Company Ltd) |
| 51384 | OxyNorm 20mg capsules (DE Pharmaceuticals) |
| 51611 | OxyNorm 10mg/1ml solution for injection ampoules (Mawdsley-Brooks & Company Ltd) |
| 51789 | OxyNorm 10mg capsules (Waymade Healthcare Plc) |
| 51896 | OxyContin 80mg modified-release tablets (Mawdsley-Brooks & Company Ltd) |
| 52216 | Longtec 5mg modified-release tablets (Qdem Pharmaceuticals Ltd) |
| 52217 | Longtec 10mg modified-release tablets (Qdem Pharmaceuticals Ltd) |
| 52220 | Longtec 40mg modified-release tablets (Qdem Pharmaceuticals Ltd) |
| 52592 | OxyNorm 10mg capsules (DE Pharmaceuticals) |
| 52809 | OxyContin 10mg modified-release tablets (Lexon (UK) Ltd) |
| 53113 | OxyContin 10mg modified-release tablets (DE Pharmaceuticals) |
| 53116 | Longtec 20mg modified-release tablets (Qdem Pharmaceuticals Ltd) |
| 54694 | Longtec 80mg modified-release tablets (Qdem Pharmaceuticals Ltd) |
| 56665 | Oxylan 10mg modified-release tablets (Chanelle Medical UK Ltd) |
| 57033 | Oxylan 10mg modified-release tablets (Actavis UK Ltd) |
| 57052 | Oxylan 20mg modified-release tablets (Actavis UK Ltd) |
| 58039 | Oxycodone 5mg/5ml oral solution |
| 58114 | Lynlor 20mg capsules (Actavis UK Ltd) |
| 58217 | Lynlor 5mg capsules (Actavis UK Ltd) |
| 58493 | Oxylan 20mg modified-release tablets (Chanelle Medical UK Ltd) |
| 58853 | Lynlor 10mg capsules (Actavis UK Ltd) |
| 59865 | Shortec 10mg capsules (Qdem Pharmaceuticals Ltd) |
| 60146 | Shortec 5mg capsules (Qdem Pharmaceuticals Ltd) |
| 60158 | Shortec 20mg capsules (Qdem Pharmaceuticals Ltd) |
| 60196 | Oxylan 40mg modified-release tablets (Chanelle Medical UK Ltd) |
| 61779 | Reltebon 40mg modified-release tablets (Actavis UK Ltd) |
| 61836 | Reltebon 20mg modified-release tablets (Actavis UK Ltd) |
| 61935 | Reltebon 10mg modified-release tablets (Actavis UK Ltd) |
| 61936 | Reltebon 5mg modified-release tablets (Actavis UK Ltd) |
| 62322 | Reltebon 80mg modified-release tablets (Actavis UK Ltd) |
| 63198 | OxyContin 40mg modified-release tablets (DE Pharmaceuticals) |
| 63332 | Reltebon 30mg modified-release tablets (Actavis UK Ltd) |
| 63714 | Reltebon 60mg modified-release tablets (Actavis UK Ltd) |
| 64150 | Oxylan 5mg modified-release tablets (Chanelle Medical UK Ltd) |
| 64164 | Reltebon 15mg modified-release tablets (Actavis UK Ltd) |
| 64333 | Longtec 30mg modified-release tablets (Qdem Pharmaceuticals Ltd) |
| 64426 | Longtec 15mg modified-release tablets (Qdem Pharmaceuticals Ltd) |
| 64552 | Longtec 60mg modified-release tablets (Qdem Pharmaceuticals Ltd) |
| 64807 | Longtec 120mg modified-release tablets (Qdem Pharmaceuticals Ltd) |
| 64965 | Oxycodone 5mg/5ml oral solution sugar free (Wockhardt UK Ltd) |
| 65390 | Abtard 30mg modified-release tablets (Ethypharm UK Ltd) |
| 65392 | Abtard 20mg modified-release tablets (Ethypharm UK Ltd) |
| 65932 | Abtard 40mg modified-release tablets (Ethypharm UK Ltd) |
| 65933 | Abtard 80mg modified-release tablets (Ethypharm UK Ltd) |
| 66298 | Abtard 10mg modified-release tablets (Ethypharm UK Ltd) |
| 66606 | Oxeltra 10mg modified-release tablets (Wockhardt UK Ltd) |
| 66616 | OxyContin 5mg modified-release tablets (Mawdsley-Brooks & Company Ltd) |
| 66619 | OxyContin 20mg modified-release tablets (Waymade Healthcare Plc) |
| 66760 | Abtard 5mg modified-release tablets (Ethypharm UK Ltd) |
| 66837 | Abtard 15mg modified-release tablets (Ethypharm UK Ltd) |
| 67446 | Abtard 60mg modified-release tablets (Ethypharm UK Ltd) |
| 68797 | Oxeltra 20mg modified-release tablets (Wockhardt UK Ltd) |
| 69474 | Oxycodone 5mg/5ml oral solution sugar free (DE Pharmaceuticals) |
| 69559 | Shortec liquid 5mg/5ml oral solution (Qdem Pharmaceuticals Ltd) |
| 69993 | Shortec 10mg/ml concentrate oral solution (Qdem Pharmaceuticals Ltd) |
| 70123 | Shortec 10mg/1ml solution for injection ampoules (Qdem Pharmaceuticals Ltd) |
| 71335 | Oxycodone 20mg modified-release tablets (Teva UK Ltd) |
| 166 | PAPAVERETUM 20 MG INJ |
| 685 | Aspav dispersible tablets (Actavis UK Ltd) |
| 6226 | Aspirin 500mg / Papaveretum 7.71mg dispersible tablets sugar free |
| 11129 | Papaveretum 15.4mg/1ml solution for injection ampoules |
| 15353 | Papaveretum 7.7mg/1ml solution for injection ampoules |
| 18261 | Aspirin 500mg with Papaveretum 7.71mg dispersible tablets |
| 19764 | Papaveretum 10mg tablet |
| 26115 | Omnopon-scopolamine Injection (Roche Products Ltd) |
| 28732 | Papaveretum with hyoscine 7.7mg with 400 micrograms/ml injection |
| 47072 | Omnopon 15.4mg/ml Injection (Roche Products Ltd) |
| 4557 | ASPIRIN & PAPAVERUTUM 10 MG TAB |
| 687 | Tramacet 37.5mg/325mg tablets (Grunenthal Ltd) |
| 6558 | Tramadol 37.5mg / Paracetamol 325mg tablets |
| 7450 | Pentazocine 15mg with paracetamol 500mg tablet |
| 10509 | Fortagesic Tablet (Sanofi-Synthelabo Ltd) |
| 16076 | Paracetamol 325mg with tramadol 37.5 mg tablet |
| 36472 | Paracetamol 500 mg+ pentazocine 15mg tablet |
| 42280 | Tramadol 37.5mg / Paracetamol 325mg effervescent tablets sugar free |
| 42332 | Tramacet 37.5mg/325mg effervescent tablets (Grunenthal Ltd) |
| 62778 | Tramacet 37.5mg/325mg tablets (Waymade Healthcare Plc) |
| 64459 | Tramadol 37.5mg / Paracetamol 325mg tablets (A A H Pharmaceuticals Ltd) |
| 123 | Fortral 30mg/ml Injection (Sterwin Medicines) |
| 328 | Pentazocine 50mg capsules |
| 2367 | Pentazocine 25mg tablets |
| 7450 | Pentazocine 15mg with paracetamol 500mg tablet |
| 8375 | Fortral 25mg tablets (Zentiva) |
| 10509 | Fortagesic Tablet (Sanofi-Synthelabo Ltd) |
| 10583 | Pentazocine 30mg/ml injection |
| 10769 | Fortral 50mg Capsule (Sanofi-Synthelabo Ltd) |
| 17863 | Pentazocine 50mg suppositories |
| 25833 | Fortral 50mg suppositories (Sterwin Medicines) |
| 31582 | Pentazocine 30mg/1ml solution for injection ampoules |
| 31584 | Pentazocine 60mg/2ml solution for injection ampoules |
| 36472 | Paracetamol 500 mg+ pentazocine 15mg tablet |
| 38092 | Pentazocine 30mg/ml Injection (Sterwin Medicines) |
| 42094 | Fortral 30mg/1ml solution for injection ampoules (Zentiva) |
| 44867 | Fortral 60mg/2ml solution for injection ampoules (Zentiva) |
| 71170 | Pentazocine 25mg tablets (Actavis UK Ltd) |
| 234 | Pethidine 25mg tablet |
| 249 | Pethidine 10mg/ml injection |
| 423 | Pethidine 50mg/ml injection |
| 826 | Pethidine 50mg/1ml solution for injection ampoules |
| 2450 | Pethidine 50mg tablets |
| 2966 | Pethidine 50mg/ml injection |
| 17043 | Pethidine 100mg/2ml solution for injection ampoules |
| 17386 | Pethidine 50mg Tablet (Roche Products Ltd) |
| 18491 | Pamergan P100 solution for injection 2ml ampoules (Martindale Pharmaceuticals Ltd) |
| 19116 | Pethidine 100mg/2ml / Promethazine 50mg/2ml solution for injection ampoules |
| 22896 | PETHIDINE CO 50 MG INJ |
| 23442 | Pethidine 50mg/5ml solution for injection ampoules |
| 24867 | Pethidine with levallorphan tartrate injection |
| 29426 | Pethidine 50mg/ml Injection (Martindale Pharmaceuticals Ltd) |
| 30319 | Pethidine powder |
| 31253 | Pethidine 50mg/ml Injection (Auden McKenzie (Pharma Division) Ltd) |
| 31885 | Pethidine 100mg/10ml solution for injection ampoules |
| 31935 | Pethidine 50mg Tablet (Roche Products Ltd) |
| 32831 | Pethidine 100mg/2ml Injection (Roche Products Ltd) |
| 33954 | Promethazine hydrochloride 50mg with pethidine 100mg/2ml injection |
| 37703 | Pethidine 50mg/1ml solution for injection ampoules (AMCo) |
| 38013 | Pethidine 50mg capsules |
| 38103 | Pethidine 25mg Tablet (Roche Products Ltd) |
| 40239 | Pethidine 50mg tablets (Martindale Pharmaceuticals Ltd) |
| 41550 | Pethidine 100mg/2ml Injection (C P Pharmaceuticals Ltd) |
| 42708 | Pethidine 50mg/ml intramuscular injection (Roche Products Ltd) |
| 45325 | Pethidine injection |
| 48128 | Pethidine 100mg/2ml solution for injection ampoules (Martindale Pharmaceuticals Ltd) |
| 48148 | Pethidine 100mg/2ml solution for injection ampoules (Actavis UK Ltd) |
| 52400 | Pethidine 100mg/10ml solution for injection ampoules (Alliance Healthcare (Distribution) Ltd) |
| 53709 | Pethidine oral liquid |
| 53929 | Pethidine 50mg/1ml solution for injection ampoules (A A H Pharmaceuticals Ltd) |
| 54085 | Pethidine capsule |
| 54790 | Pethidine 50mg tablets (Teva UK Ltd) |
| 55839 | Pethidine 50mg/ml Injection (Roche Products Ltd) |
| 55852 | Pethidine 10mg/ml Injection (Martindale Pharmaceuticals Ltd) |
| 56022 | Pethidine 50mg Capsule (Martindale Pharmaceuticals Ltd) |
| 57027 | Pethidine 50mg tablets (A A H Pharmaceuticals Ltd) |
| 58190 | Pethidine 50mg/5ml solution for injection ampoules (A A H Pharmaceuticals Ltd) |
| 58737 | Pethidine 50mg tablets (Alliance Healthcare (Distribution) Ltd) |
| 63182 | Pethidine 50mg/1ml solution for injection ampoules (Alliance Healthcare (Distribution) Ltd) |
| 67599 | Pethidine 100mg/2ml solution for injection ampoules (AMCo) |
| 38183 | Remifentanil 1mg powder for solution for injection vials |
| 60296 | Remifentanil 5mg powder for solution for injection vials |
| 38183 | Remifentanil 1mg powder for solution for injection vials |
| 60296 | Remifentanil 5mg powder for solution for injection vials |
| 45800 | Tapentadol 200mg modified-release tablets |
| 45811 | Tapentadol 50mg tablets |
| 45936 | Palexia 50mg tablets (Grunenthal Ltd) |
| 45982 | Palexia SR 50mg tablets (Grunenthal Ltd) |
| 46018 | Tapentadol 100mg modified-release tablets |
| 46019 | Tapentadol 150mg modified-release tablets |
| 46020 | Palexia SR 100mg tablets (Grunenthal Ltd) |
| 46021 | Tapentadol 50mg modified-release tablets |
| 46022 | Palexia 75mg tablets (Grunenthal Ltd) |
| 46159 | Palexia SR 150mg tablets (Grunenthal Ltd) |
| 46461 | Tapentadol 75mg tablets |
| 46659 | Palexia SR 200mg tablets (Grunenthal Ltd) |
| 47399 | Tapentadol 250mg modified-release tablets |
| 47460 | Palexia SR 250mg tablets (Grunenthal Ltd) |
| 60759 | Tapentadol 20mg/ml oral solution sugar free |
| 61764 | Palexia 20mg/ml oral solution (Grunenthal Ltd) |
| 86 | Tramadol 50mg capsules |
| 187 | Zydol 50mg capsules (Grunenthal Ltd) |
| 687 | Tramacet 37.5mg/325mg tablets (Grunenthal Ltd) |
| 701 | Tramadol 50mg modified-release capsules |
| 3378 | Tramadol 50mg soluble tablets sugar free |
| 3644 | Zydol SR 100mg tablets (Grunenthal Ltd) |
| 4114 | Tramadol 100mg modified-release capsules |
| 4115 | Tramadol 100mg modified-release tablets |
| 4834 | Tramadol 150mg modified-release capsules |
| 4999 | Tramadol 24 Modified-release tablet |
| 5028 | Tramadol 24 Modified-release tablet |
| 5169 | Zydol SR 200mg tablets (Grunenthal Ltd) |
| 5257 | Tramadol 12 Modified-release tablet |
| 6153 | Zydol SR 150mg tablets (Grunenthal Ltd) |
| 6215 | Tramadol 200mg modified-release capsules |
| 6558 | Tramadol 37.5mg / Paracetamol 325mg tablets |
| 8416 | Tramadol 12 Modified-release tablet |
| 9389 | Zamadol SR 50mg capsules (Meda Pharmaceuticals Ltd) |
| 9396 | Zamadol SR 100mg capsules (Meda Pharmaceuticals Ltd) |
| 9739 | Tramadol 100mg effervescent powder sachets sugar free |
| 11101 | Zydol 50mg soluble tablets (Grunenthal Ltd) |
| 11275 | Zydol 100mg/2ml solution for injection ampoules (Grunenthal Ltd) |
| 11471 | Tramadol 100mg/2ml solution for injection ampoules |
| 11549 | Tramadol 75mg modified-release tablets |
| 11559 | Tramadol 50mg effervescent powder sachets sugar free |
| 11734 | Tramadol 50mg orodispersible tablets sugar free |
| 11746 | Tramadol 300mg modified-release tablets |
| 11748 | Tramadol 400mg modified-release tablets |
| 13813 | Zamadol 50mg capsules (Meda Pharmaceuticals Ltd) |
| 14490 | Tramake 50mg capsules (Galen Ltd) |
| 16076 | Paracetamol 325mg with tramadol 37.5 mg tablet |
| 16271 | Zydol XL 300mg tablets (Grunenthal Ltd) |
| 16395 | Zydol XL 200mg tablets (Grunenthal Ltd) |
| 19993 | Dromadol SR 100mg tablets (Teva UK Ltd) |
| 20310 | Zamadol Melt 50mg tablets (Meda Pharmaceuticals Ltd) |
| 21256 | Tramake Insts 50mg sachets (Galen Ltd) |
| 21397 | Zydol XL 400mg tablets (Grunenthal Ltd) |
| 21777 | Dromadol SR 200mg tablets (Teva UK Ltd) |
| 21797 | Zamadol SR 200mg capsules (Meda Pharmaceuticals Ltd) |
| 21947 | Zydol XL 150mg tablets (Grunenthal Ltd) |
| 23625 | Dromadol SR 150mg tablets (Teva UK Ltd) |
| 23981 | Zamadol SR 150mg capsules (Meda Pharmaceuticals Ltd) |
| 24383 | Tramake Insts 100mg sachets (Galen Ltd) |
| 26336 | Dromadol XL 300mg tablets (IVAX Pharmaceuticals UK Ltd) |
| 26986 | Zamadol 24hr 200mg modified-release tablets (Meda Pharmaceuticals Ltd) |
| 27591 | Zamadol 24hr 150mg modified-release tablets (Meda Pharmaceuticals Ltd) |
| 28728 | Zamadol 24hr 300mg modified-release tablets (Meda Pharmaceuticals Ltd) |
| 29324 | Dromadol SR 75mg tablets (IVAX Pharmaceuticals UK Ltd) |
| 29860 | Tramadol 50mg capsules (IVAX Pharmaceuticals UK Ltd) |
| 31105 | Dromadol XL 200mg tablets (IVAX Pharmaceuticals UK Ltd) |
| 31107 | Dromadol XL 150mg tablets (IVAX Pharmaceuticals UK Ltd) |
| 31734 | Dromadol XL 400mg tablets (IVAX Pharmaceuticals UK Ltd) |
| 32165 | Tramadol 50mg Capsule (Generics (UK) Ltd) |
| 32450 | Zamadol 24hr 400mg modified-release tablets (Meda Pharmaceuticals Ltd) |
| 34065 | Tramadol sr 150mg Modified-release tablet (Winthrop Pharmaceuticals Ltd) |
| 34260 | Tramadol sr 100mg Modified-release tablet (Winthrop Pharmaceuticals Ltd) |
| 34281 | Tramadol sr 200mg Modified-release tablet (Winthrop Pharmaceuticals Ltd) |
| 34422 | Tramadol 50mg capsules (Actavis UK Ltd) |
| 34521 | Tramadol 50mg capsules (A A H Pharmaceuticals Ltd) |
| 34570 | Tramadol 50mg capsules (Teva UK Ltd) |
| 34639 | Tramadol 50mg capsules (Genus Pharmaceuticals Ltd) |
| 34808 | Tramadol 50mg capsules (PLIVA Pharma Ltd) |
| 35347 | Tramadol 24 Modified-release tablet |
| 35438 | Tramquel SR 100mg capsules (Beechmere Pharmaceuticals Ltd) |
| 35651 | Tradorec XL 200mg tablets (Endo Ventures Ltd) |
| 35656 | Tradorec XL 100mg tablets (Endo Ventures Ltd) |
| 35806 | Larapam SR 100mg tablets (Sandoz Ltd) |
| 36035 | Tradorec XL 300mg tablets (Endo Ventures Ltd) |
| 36697 | Mabron 200mg modified-release tablets (Morningside Healthcare Ltd) |
| 36732 | Tramadol 50mg modified-release tablets |
| 36873 | Zydol SR 50mg tablets (Grunenthal Ltd) |
| 36949 | Tramquel SR 50mg capsules (Beechmere Pharmaceuticals Ltd) |
| 37020 | Tramadol 150mg modified-release tablets |
| 37021 | Tramadol 200mg modified-release tablets |
| 37831 | Mabron 100mg modified-release tablets (Morningside Healthcare Ltd) |
| 37867 | Tramadol (roi) Tablet |
| 38196 | Larapam SR 200mg tablets (Sandoz Ltd) |
| 38528 | Tramadol 50mg Capsule (Tillomed Laboratories Ltd) |
| 38874 | Zamadol 100mg/2ml solution for injection ampoules (Meda Pharmaceuticals Ltd) |
| 38956 | Tramquel SR 200mg capsules (Beechmere Pharmaceuticals Ltd) |
| 39505 | Marol 100mg modified-release tablets (Morningside Healthcare Ltd) |
| 39709 | Marol 200mg modified-release tablets (Morningside Healthcare Ltd) |
| 39750 | Marol 150mg modified-release tablets (Morningside Healthcare Ltd) |
| 39798 | Nobligan retard 100mg tablets (Grunenthal Ltd) |
| 39811 | Maxitram SR 200mg capsules (Chiesi Ltd) |
| 40058 | Tramulief SR 100mg tablets (AMCo) |
| 40060 | Tramulief SR 200mg tablets (AMCo) |
| 40061 | Tramulief SR 150mg tablets (AMCo) |
| 40166 | Tramadol 50mg capsules (Niche Generics Ltd) |
| 40249 | Maxitram SR 100mg capsules (Chiesi Ltd) |
| 40254 | Maxitram SR 50mg capsules (Chiesi Ltd) |
| 40718 | Tramadol 50mg capsules (Almus Pharmaceuticals Ltd) |
| 40805 | Tramquel SR 150mg capsules (Beechmere Pharmaceuticals Ltd) |
| 40883 | Maxitram SR 150mg capsules (Chiesi Ltd) |
| 40926 | Larapam SR 150mg tablets (Sandoz Ltd) |
| 41976 | Tramadol 100mg modified-release tablets (A A H Pharmaceuticals Ltd) |
| 42280 | Tramadol 37.5mg / Paracetamol 325mg effervescent tablets sugar free |
| 42332 | Tramacet 37.5mg/325mg effervescent tablets (Grunenthal Ltd) |
| 42798 | Tramadol 150mg modified-release tablets (A A H Pharmaceuticals Ltd) |
| 43198 | Tramadol sr 50mg Capsule (Hillcross Pharmaceuticals Ltd) |
| 43513 | Tramadol 50mg capsules (Zentiva) |
| 44371 | Mabron 150mg modified-release tablets (Morningside Healthcare Ltd) |
| 46279 | Tramadol 200mg modified-release capsules (A A H Pharmaceuticals Ltd) |
| 46587 | Tramadol 100mg/ml oral drops |
| 46643 | Zeridame SR 150mg tablets (Actavis UK Ltd) |
| 47854 | Tramadol (roi) Tablet |
| 48090 | Tramadol 200mg modified-release tablets (A A H Pharmaceuticals Ltd) |
| 49323 | Marol 150mg modified-release tablets (Teva UK Ltd) |
| 49324 | Marol 100mg modified-release tablets (Teva UK Ltd) |
| 50862 | Marol 200mg modified-release tablets (Teva UK Ltd) |
| 50947 | Tramadol 100mg modified-release capsules (Alliance Healthcare (Distribution) Ltd) |
| 52495 | Tramadol 50mg capsules (Bristol Laboratories Ltd) |
| 52605 | Tramadol 50mg capsules (Accord Healthcare Ltd) |
| 52977 | Tramadol 100mg modified-release capsules (A A H Pharmaceuticals Ltd) |
| 54023 | Tramadol 50mg modified-release capsules (A A H Pharmaceuticals Ltd) |
| 56491 | Zamadol SR 200mg capsules (Lexon (UK) Ltd) |
| 58129 | Zeridame SR 100mg tablets (Actavis UK Ltd) |
| 58316 | Tramadol 50mg modified-release capsules (DE Pharmaceuticals) |
| 60121 | Tramadol 50mg modified-release capsules (Waymade Healthcare Plc) |
| 60751 | Tilodol SR 200mg tablets (Sandoz Ltd) |
| 61272 | Tramadol 50mg capsules (Phoenix Healthcare Distribution Ltd) |
| 61610 | Tramadol 50mg capsules (Morningside Healthcare Ltd) |
| 61775 | Tramadol 50mg capsules (Sigma Pharmaceuticals Plc) |
| 62778 | Tramacet 37.5mg/325mg tablets (Waymade Healthcare Plc) |
| 63047 | Tramadol 100mg modified-release capsules (Waymade Healthcare Plc) |
| 63898 | Tramadol 50mg modified-release capsules (J M McGill Ltd) |
| 64459 | Tramadol 37.5mg / Paracetamol 325mg tablets (A A H Pharmaceuticals Ltd) |
| 64496 | Tramadol 100mg modified-release capsules (Ennogen Healthcare Ltd) |
| 64731 | Tramadol 100mg modified-release capsules (Icarus Pharmaceuticals Ltd) |
| 64871 | Maneo 100mg modified-release tablets (Mylan) |
| 65266 | Tramadol 50mg capsules (Kent Pharmaceuticals Ltd) |
| 65954 | Tramadol 50mg modified-release capsules (Cubic Pharmaceuticals Ltd) |
| 66299 | Maneo 200mg modified-release tablets (Mylan) |
| 66729 | Tilodol SR 100mg tablets (Sandoz Ltd) |
| 67161 | Tramadol 150mg modified-release capsules (A A H Pharmaceuticals Ltd) |
| 67197 | Tramadol 50mg capsules (DE Pharmaceuticals) |
| 67310 | Zydol SR 200mg tablets (Mawdsley-Brooks & Company Ltd) |
| 67323 | Zydol SR 150mg tablets (Waymade Healthcare Plc) |
| 67744 | Zydol 50mg capsules (Lexon (UK) Ltd) |
| 68210 | Tramadol 100mg modified-release tablets (Elite Pharma (Surrey) Ltd) |
| 68427 | Tramadol 50mg modified-release capsules (CST Pharma Ltd) |
| 68833 | Tramadol 100mg modified-release capsules (DE Pharmaceuticals) |
| 69894 | Zeridame SR 200mg tablets (Actavis UK Ltd) |
| 71355 | Tramadol 150mg modified-release capsules (Waymade Healthcare Plc) |
| 71358 | Tramadol 150mg modified-release tablets (Sigma Pharmaceuticals Plc) |
| 72646 | Zydol SR 100mg tablets (DE Pharmaceuticals) |
| **Weak opioids** | |
| 306 | Aspirin with codeine phosphate and caffeine tablets |
| 2047 | Co-codaprin 8mg with 400mg tablets |
| 2986 | Co-codaprin 8mg/400mg dispersible tablets |
| 7518 | Aspirin 400mg with Codeine 8mg dispersible tablets |
| 7770 | ASPIRIN/CODEINE PHOSPHATE/PARACETAMOL 250 MG TAB |
| 7989 | DIHYDROCODEINE TARTRATE/ASPIRIN 300 MG TAB |
| 9044 | Codis 500 dispersible tablets (Reckitt Benckiser Healthcare (UK) Ltd) |
| 9129 | Paracetamol 250mg with aspirin 250mg & codeine 6.8mg tablet |
| 9432 | Aspirin 500mg / Codeine 8mg soluble tablets |
| 11961 | Veganin Tablet (Pfizer Consumer Healthcare Ltd) |
| 12992 | Aspirin 500mg / Codeine 8mg dispersible tablets sugar free |
| 13598 | ASPIRIN & CODEINE 500 MG TAB |
| 15779 | Codeine 8mg with aspirin 500mg soluble tablets |
| 17926 | Aspirin 400mg with Codeine 8mg tablets |
| 19724 | ASPIRIN & CODEINE paed 75 MG TAB |
| 20127 | Codeine phosphate 8mg with aspirin 400mg with caffeine dispersible tablets |
| 21067 | Doloxene Capsule (Eli Lilly and Company Ltd) |
| 22450 | ASPIRIN & CODEINE 75 MG TAB |
| 24498 | ASPIRIN/CODEINE PHOSPHATE/PARACETAMOL 300 MG TAB |
| 24828 | Hypon Tablet (Wellcome Medical Division) |
| 25959 | ASPIRIN/CAFFEINE/DEXTROPROPOXYPHENE NAPS PUL |
| 28606 | ASPIRIN/CAFFEINE/CODEINE PHOSPHATE 300 MG TAB |
| 28784 | Codeine phosphate 8mg with aspirin 400mg tablets |
| 31498 | ASPIRIN / CAFFEINE CIT./ CODEINE PHOS./ 200 MG TAB |
| 31894 | Safapryn -Co Tablet (Pfizer Ltd) |
| 42218 | Co-codaprin 8mg/400mg dispersible tablets (A A H Pharmaceuticals Ltd) |
| 44311 | DEXTROPROPOXYPHENE NAPSYLATE/ASPIRIN 100 MG TAB |
| 46925 | Co-codaprin 8mg/400mg dispersible tablets (Actavis UK Ltd) |
| 52856 | Co-codaprin 8mg/400mg tablets |
| 63658 | Co-codaprin 400/8 Tablet (Hillcross Pharmaceuticals Ltd) |
| 63683 | Aspirin with codeine Dispersible tablet (Actavis UK Ltd) |
| 19 | Co-codamol 8mg/500mg tablets |
| 57 | Co-codamol 8mg/500mg effervescent tablets |
| 96 | Co-codamol 30mg/500mg tablets |
| 142 | CODEINE SOLUBLE TAB |
| 152 | Codeine 15mg/5ml linctus |
| 158 | Codeine 30mg tablets |
| 203 | CODEINE CO SOLUBLE TAB |
| 213 | Codeine 25mg/5ml oral solution |
| 241 | CODEINE CO TAB |
| 306 | Aspirin with codeine phosphate and caffeine tablets |
| 382 | Codeine 15mg tablets |
| 462 | Codeine 60mg/1ml solution for injection ampoules |
| 539 | Codeine 60mg tablets |
| 625 | Co-codamol 8mg/500mg capsules |
| 635 | Codeine 30mg/1ml solution for injection ampoules |
| 656 | Tylex 30mg/500mg capsules (UCB Pharma Ltd) |
| 767 | Solpadol 30mg/500mg capsules (Sanofi) |
| 800 | Co-codamol 30mg/500mg capsules |
| 810 | Co-codamol 30mg/500mg effervescent tablets |
| 913 | Kaodene oral suspension (Sovereign Medical Ltd) |
| 1261 | Co-codamol 30mg/500mg effervescent powder sachets sugar free |
| 1616 | Migraleve Pink tablets (McNeil Products Ltd) |
| 1617 | Codeine 15mg/5ml linctus sugar free |
| 1640 | Kapake 30mg/500mg tablets (Galen Ltd) |
| 1708 | Codafen Continus tablets (Napp Pharmaceuticals Ltd) |
| 2047 | Co-codaprin 8mg with 400mg tablets |
| 2178 | Phensedyl Oral solution (Rhone-Poulenc Rorer Ltd) |
| 2211 | Solpadol 30mg/500mg effervescent tablets (Sanofi) |
| 2250 | CODEINE & PARACETAMOL TAB |
| 2698 | CODEINE & PARACETAMOL 8 MG TAB |
| 2764 | Codeine 3mg/5ml linctus paediatric sugar free |
| 2794 | Co-codamol 30mg/500mg tablets (Wockhardt UK Ltd) |
| 2846 | Paracetamol 500mg with codeine phosphate 30mg effervescent tablet |
| 2917 | Paracetamol 500mg with codeine phosphate 30 mg tablet |
| 2986 | Co-codaprin 8mg/400mg dispersible tablets |
| 2988 | CODEINE PHOSPHATE 15 MG ELI |
| 3034 | Diarrest Liquid (Galen Ltd) |
| 3156 | Solpadol 30mg/500mg caplets (Sanofi) |
| 3185 | Paracetamol 500mg with codeine phosphate 30mg capsule |
| 3435 | Tylex 30mg/500mg effervescent tablets (UCB Pharma Ltd) |
| 3713 | Medocodene Tablet (Manufacturer unknown) |
| 3724 | Migraleve Yellow tablets (McNeil Products Ltd) |
| 4349 | PARACETAMOL & CODEINE TAB |
| 4369 | Galcodine 3mg/5ml Oral solution (Thornton & Ross Ltd) |
| 4487 | CAFFEINE CITRATE/CODEINE PHOSPHATE/PARAC 15 MG TAB |
| 4671 | Codeine phosphate 30mg with Paracetamol 500mg capsules |
| 4718 | Migraleve - 1 Tablet (Pfizer Consumer Healthcare Ltd) |
| 4805 | Codeine phosphate 15mg/5ml diabetic oral solution |
| 5498 | Propain caplet (Sankyo Pharma UK Ltd) |
| 5572 | Galcodine 15mg/5ml linctus (Thornton & Ross Ltd) |
| 5685 | Syndol easy to swallow Tablet (SSL International Plc) |
| 6665 | Paracetamol with codeine & buclizine tablet |
| 6886 | Codeine phosphate 30mg with paracetamol 500mg tablets |
| 7072 | Co-codamol 15mg/500mg tablets |
| 7104 | Kapake 30mg/500mg effervescent tablets (Galen Ltd) |
| 7499 | Calpol extra Tablet (Warner Lambert UK Ltd) |
| 7518 | Aspirin 400mg with Codeine 8mg dispersible tablets |
| 7534 | Benylin with Codeine oral solution (Pfizer Consumer Healthcare Ltd) |
| 7542 | Codeine phosphate 8mg with paracetamol 500mg tablets |
| 7696 | PARACETAMOL 500MG/CODEINE 10MG MG TAB |
| 7770 | ASPIRIN/CODEINE PHOSPHATE/PARACETAMOL 250 MG TAB |
| 7976 | PARACETAMOL 450MG/CODEINE 8.1MG TAB |
| 8053 | BROMPHENIRAMINE /CODEINE PHOSPHATE / 2 MG ELI |
| 8233 | Kaolin light 1.5g/5ml / Codeine 5mg/5ml oral suspension sugar free |
| 8246 | Codeine phosphate 8mg with paracetamol 500mg effervescent tablets |
| 8329 | Solpadeine Tablet (GlaxoSmithKline Consumer Healthcare) |
| 8335 | Paracodol 8mg/500mg capsules (Bayer Plc) |
| 8500 | CODEINE PHOSPHATE/PARACETAMOL/SODIUM CIT 8 MG CAP |
| 8732 | BUCLIZINE HYD/CODEINE PHOS/DOCUSATE SOD/ 6.25 MG TAB |
| 8835 | CODEINE PHOS /DIPHENHYDRAMINE HYD. /MENT 5.7 MG ELI |
| 8879 | Terpin 32.5mg/5ml / Codeine 16mg/5ml linctus |
| 9044 | Codis 500 dispersible tablets (Reckitt Benckiser Healthcare (UK) Ltd) |
| 9129 | Paracetamol 250mg with aspirin 250mg & codeine 6.8mg tablet |
| 9202 | CODEINE PHOS/EPHEDRINE HYD/PROMETHAZINE LIN |
| 9432 | Aspirin 500mg / Codeine 8mg soluble tablets |
| 9457 | Paracetamol 500mg with codeine phosphate 8mg tablet |
| 9460 | Paracetamol 500mg with codeine phosphate 8mg capsule |
| 9462 | Paracetamol 500mg with codeine phosphate 8mg effervescent tablet |
| 9516 | Kapake 30mg/500mg capsules (Galen Ltd) |
| 9742 | Solpadeine Soluble tablet (GlaxoSmithKline Consumer Healthcare) |
| 9917 | Kapake Insts 30mg/500mg effervescent powder sachets (Galen Ltd) |
| 10099 | Paracetamol 500mg with codeine phosphate 8mg & buclizine 6.25mg |
| 10176 | Codipar 15mg/500mg tablets (AMCo) |
| 10178 | Solpadeine Plus capsules (Omega Pharma Ltd) |
| 10226 | Solpadeine Plus tablets (Omega Pharma Ltd) |
| 10519 | CODEINE PHOS/IBUPROFEN SR (20MG/300MG) TAB |
| 10582 | Formulix Oral solution (Cilag Pharmaceuticals Ltd) |
| 10602 | Paracodol 8mg/500mg effervescent tablets (Bayer Plc) |
| 10701 | Dimotane Co oral solution paediatric sugar free (Wyeth Consumer Healthcare) |
| 11009 | Paracetamol 500mg with codeine phosphate 8mg & caffeine 30mg tablet |
| 11250 | Migraleve - 2 8mg+500mg Tablet (Pfizer Consumer Healthcare Ltd) |
| 11325 | Paracetamol 500mg with codeine phosphate 30 mg tablet |
| 11461 | Ibuprofen 300mg modified-release / Codeine 20mg tablets |
| 11554 | Ibuprofen 200mg / Codeine 12.8mg tablets |
| 11665 | Zapain 30mg/500mg tablets (AMCo) |
| 11807 | Paracetamol 500mg with codeine 12.8mg tablet |
| 11945 | Syndol Tablet (SSL International Plc) |
| 11961 | Veganin Tablet (Pfizer Consumer Healthcare Ltd) |
| 12171 | Dimotane Co oral solution sugar free (Wyeth Consumer Healthcare) |
| 12709 | Ibuprofen and codeine 200mg + 12.5mg Tablet |
| 12992 | Aspirin 500mg / Codeine 8mg dispersible tablets sugar free |
| 13598 | ASPIRIN & CODEINE 500 MG TAB |
| 13893 | Nurofen Plus tablets (Reckitt Benckiser Healthcare (UK) Ltd) |
| 13992 | Pseudoephedrine with brompheniramine & codeine phosphate oral solution |
| 14378 | Feminax tablets (Bayer Plc) |
| 14602 | Co-codamol 60mg/1000mg effervescent powder sachets sugar free |
| 14676 | Paracetamol 500mg with codeine phosphate 8mg & caffeine 30mg effervescent tablet |
| 14785 | Paracetamol500mg with codeine phosphate 15mg tablet |
| 14912 | Codeine phosphate 8mg with Paracetamol 500mg capsules |
| 14964 | Solpadeine Plus soluble tablets (Omega Pharma Ltd) |
| 15360 | Codeine phosphate powder |
| 15779 | Codeine 8mg with aspirin 500mg soluble tablets |
| 15831 | Codeine phosphate 30mg with paracetamol 500mg effervescent powder sugar free |
| 15871 | Kapake Insts 60mg/1000mg effervescent powder sachets (Galen Ltd) |
| 15937 | Diphenhydramine with codeine phosphate, sodium citrate and menthol 14mg with 5.7mg with 57mg with 1.1mg/5ml oral solution |
| 16039 | Solpadeine Capsule (GlaxoSmithKline Consumer Healthcare) |
| 16096 | Codeine 6.75mg/5ml oral solution |
| 16467 | Codeine phosphate 30mg with paracetamol 500mg effervescent tablets |
| 16592 | Parahypon Tablet (Wellcome Consumer) |
| 16818 | Paracetamol 500mg with codeine phosphate 30mg effervescent powder sugar free |
| 17158 | Panadeine forte Tablet (Sanofi-Synthelabo Ltd) |
| 17563 | Solpadeine Max 12.8mg/500mg tablets (Omega Pharma Ltd) |
| 17707 | CODEINE PHOS/GUAIPHENESIN/PSEUDOEPHEDRIN 7.5 MG ELI |
| 17926 | Aspirin 400mg with Codeine 8mg tablets |
| 17998 | Medocodene 30mg/500mg capsules (UCB Pharma Ltd) |
| 18221 | Codeine phosphate 15mg with paracetamol 500mg tablets |
| 19622 | Pseudoephedrine with brompheniramine & codeine phosphate paediatric oral solution |
| 19724 | ASPIRIN & CODEINE paed 75 MG TAB |
| 19854 | Propain plus Tablet (Ceuta Healthcare Ltd) |
| 20077 | Paracetamol 400mg with codeine phosphate 10mg with diphenhydramine 5mg & caffeine 50mg tablet |
| 20127 | Codeine phosphate 8mg with aspirin 400mg with caffeine dispersible tablets |
| 20256 | Paracetamol 450mg with codeine phosphate 10mg with doxylamine 5mg & caffeine 30mg tablet |
| 20565 | Paracetamol 500mg with codeine phosphate 8mg & caffeine 30mg capsule |
| 20853 | Propain Tablet (Sankyo Pharma UK Ltd) |
| 21104 | PARACETAMOL/CODEINE PHOSPHATE 500 MG TAB |
| 21251 | Ultramol Soluble tablets (Zentiva) |
| 21673 | Paracetamol 500mg with codeine phosphate 8mg & caffeine 30mg effervescent tablet |
| 21693 | Codeine phosphate with dicycloverine with salts liquid |
| 21703 | Paracetamol 1000mg with codeine phosphate 60mg effervescent powder sugar free |
| 21746 | Pharmidone Tablet (Pharmacia Ltd) |
| 21880 | Zapain 30mg/500mg capsules (AMCo) |
| 22129 | Terpoin Oral solution (Hough Hoseason & Co Ltd) |
| 22450 | ASPIRIN & CODEINE 75 MG TAB |
| 22627 | CODEINE PHOSPHATE 15/PARACETAMOL 500MG TAB |
| 22764 | Panadeine Tablet (GlaxoSmithKline UK Ltd) |
| 23420 | Codeine phosphate 60mg with paracetamol 1000mg effervescent powder sugar free |
| 23580 | Codeine phosphate with diphenhydramine with paracetamol with phenylephrine with caffeine with vitamin c tablets |
| 23952 | Panadeine Effervescent tablet (Sanofi-Synthelabo Ltd) |
| 23977 | Codeine 15mg/5ml linctus (Thornton & Ross Ltd) |
| 24124 | Codeine 15mg/5ml linctus (A A H Pharmaceuticals Ltd) |
| 24125 | Codeine 15mg/5ml Oral solution (Approved Prescription Services Ltd) |
| 24187 | Paracetamol with diphenhydramine with codeine with phenylephrine with caffeine & vitamin c tablet |
| 24304 | CODEINE PHOS/GUAIPHENESIN/PSEUDOEPHEDRIN 3 MG ELI |
| 24498 | ASPIRIN/CODEINE PHOSPHATE/PARACETAMOL 300 MG TAB |
| 24517 | CODEINE PHOSPHATE / MAGNESIUM CHLORIDE MG MIX |
| 24828 | Hypon Tablet (Wellcome Medical Division) |
| 24859 | CODEINE 8MG & PARACETAMOL 500MG SUP |
| 24996 | Codeine phosphate 8mg with paracetamol 500mg with buclizine 6.25mg tablets |
| 25109 | Veganin tablets (Omega Pharma Ltd) |
| 25330 | Solpaflex tablets (GlaxoSmithKline Consumer Healthcare) |
| 25485 | Codeine phosphate with other ingredient pastilles |
| 25514 | Paracetamol 500mg with codeine phosphate 10mg tablet |
| 25529 | Paracetamol 500mg with codeine phosphate 8mg & caffeine 30mg tablet |
| 26291 | Phenylephrine with diphenhydramine, paracetamol, codeine, caffeine & vitamin c tablet |
| 27353 | CAFFEINE /CODEINE PHOS./DOXYLAMINE SUCCI 30 MG TAB |
| 27598 | CAFFEINE/CODEINE PHOS./DIPHENHYDRAMINE H 50 MG TAB |
| 27784 | Co-codamol 8mg/500mg tablets (Actavis UK Ltd) |
| 27785 | Co-codamol 30mg/500mg tablets (Zentiva) |
| 28606 | ASPIRIN/CAFFEINE/CODEINE PHOSPHATE 300 MG TAB |
| 28756 | Robitussin night-time Oral solution (Wyeth Consumer Healthcare) |
| 28784 | Codeine phosphate 8mg with aspirin 400mg tablets |
| 29342 | Co-codamol 8mg+500mg Tablet (M & A Pharmachem Ltd) |
| 29373 | Pardale 400mg+9mg+10mg Tablet (Martindale Pharmaceuticals Ltd) |
| 29488 | Co-codamol 8mg/500mg effervescent tablets (Zentiva) |
| 29828 | ACETYLSALICYLIC ACID / CODEINE PHOSPHATE MG TAB |
| 30021 | Paracetamol 500mg with codeine phosphate 13.5mg tablet |
| 30123 | Panadol Ultra 12.8mg/500mg tablets (GlaxoSmithKline Consumer Healthcare) |
| 30556 | Co-codamol 8mg/500mg effervescent tablets (Fannin UK Ltd) |
| 31155 | Parake Tablet (Galen Ltd) |
| 31452 | Codeine 30mg tablets (Wockhardt UK Ltd) |
| 31498 | ASPIRIN / CAFFEINE CIT./ CODEINE PHOS./ 200 MG TAB |
| 31577 | Co-codamol 30mg/500mg capsules (A A H Pharmaceuticals Ltd) |
| 31700 | Codeine 15mg tablets (Actavis UK Ltd) |
| 31871 | Paracetamol 450mg with codeine phosphate 8.1mg tablet |
| 31894 | Safapryn -Co Tablet (Pfizer Ltd) |
| 31943 | Codeine 30mg tablets (IVAX Pharmaceuticals UK Ltd) |
| 32436 | Codeine 3mg/5ml linctus paediatric (Thornton & Ross Ltd) |
| 32510 | CODEINE LINCTUS |
| 32519 | Painex caplets (Sandoz Ltd) |
| 32692 | Co-codamol 8mg/500mg effervescent tablets (A A H Pharmaceuticals Ltd) |
| 32891 | Syndol caplets (Sanofi) |
| 33260 | Paracetamol with codeine with hyoscine & caffeine tablet |
| 33495 | BUCLIZINE HYD/CODEINE PHOS/PARACET / COD 6.25 MG TAB |
| 33528 | Codeine 15mg/5ml linctus sugar free (A A H Pharmaceuticals Ltd) |
| 33643 | Co-codamol 8mg+500mg Tablet (Family Health) |
| 33653 | Co-codamol 8mg/500mg tablets (IVAX Pharmaceuticals UK Ltd) |
| 33679 | Co-codamol 8mg/500mg tablets (Zentiva) |
| 33688 | Co-codamol 30mg/500mg effervescent tablets (Fannin UK Ltd) |
| 33961 | Co-codamol 30mg/500mg capsules (IVAX Pharmaceuticals UK Ltd) |
| 34090 | Codeine Oral solution (Berk Pharmaceuticals Ltd) |
| 34099 | Codeine 15mg/5ml linctus sugar free (Pinewood Healthcare) |
| 34152 | Codeine 15mg/5ml Oral solution (Celltech Pharma Europe Ltd) |
| 34168 | Codeine 15mg/5ml linctus sugar free (Thornton & Ross Ltd) |
| 34172 | Codeine 15mg/5ml Oral solution (William Ransom) |
| 34176 | Codeine 15mg/5ml Oral solution (Rusco Ltd) |
| 34229 | Co-codamol 8mg+500mg Dispersible tablet (Rhone-Poulenc Rorer Ltd) |
| 34257 | Co-codamol 8mg/500mg effervescent tablets (Mylan) |
| 34264 | Co-codamol 30mg/500mg tablets (IVAX Pharmaceuticals UK Ltd) |
| 34348 | Codeine 15mg tablets (A A H Pharmaceuticals Ltd) |
| 34373 | Codeine Oral solution (Co-operative) |
| 34383 | Codeine 30mg tablets (A A H Pharmaceuticals Ltd) |
| 34437 | Codeine 15mg/5ml Oral solution (Nucare Plc) |
| 34444 | Codeine 15mg tablets (Wockhardt UK Ltd) |
| 34495 | Co-codamol 8mg/500mg tablets (Mylan) |
| 34497 | Co-codamol 8mg+500mg Tablet (Berk Pharmaceuticals Ltd) |
| 34518 | Co-codamol 8mg/500mg tablets (A A H Pharmaceuticals Ltd) |
| 34552 | Codeine 30mg tablets (Actavis UK Ltd) |
| 34667 | Co-codamol 30mg/500mg tablets (A A H Pharmaceuticals Ltd) |
| 34784 | Co-codamol 8mg/500mg effervescent tablets (Sandoz Ltd) |
| 34789 | Codeine 30mg tablets (Kent Pharmaceuticals Ltd) |
| 34815 | Co-codamol 8mg/500mg tablets (Kent Pharmaceuticals Ltd) |
| 34840 | Co-codamol 30mg/500mg tablets (Almus Pharmaceuticals Ltd) |
| 34845 | Co-codamol 30mg/500mg effervescent tablets (Zentiva) |
| 34865 | Co-codamol 8mg+500mg Tablet (C P Pharmaceuticals Ltd) |
| 34968 | Co-codamol 8mg/500mg tablets (Teva UK Ltd) |
| 35792 | Codeine 25mg/5ml oral solution (Thornton & Ross Ltd) |
| 35965 | Medocodene 30mg/500mg effervescent tablets (Mylan) |
| 36013 | Pulmo Bailly oral solution (Dendron Ltd) |
| 36152 | Propain caplets (Ceuta Healthcare Ltd) |
| 36488 | Co-codamol 30mg/500mg capsules (Mylan) |
| 36608 | Co-codamol Effervescent tablet (A A H Pharmaceuticals Ltd) |
| 36846 | Guaiacol 75mg/5ml / Codeine 7mg/5ml oral solution |
| 36993 | Co-codamol 30mg/500mg capsules (Teva UK Ltd) |
| 37298 | PARACETAMOL,CAFFEINE,CODEINE & NICOTINAM TAB |
| 37348 | Codeine phosphate with paracetamol, doxylamine and caffeine 10mg with 450mg with 5mg with 30mg tablets |
| 37816 | Cuprofen PLUS tablets (SSL International Plc) |
| 37904 | Co-codamol 12.8mg/500mg tablets |
| 38085 | Paracetamol 500mg with codeine phosphate 10mg capsule |
| 38088 | Paracetamol 500mg with codeine phosphate 30 mg tablet |
| 38363 | Codeine phosphate 12.8mg with paracetamol 500mg tablets |
| 38987 | Galcodine 3mg/5ml linctus paediatric (Thornton & Ross Ltd) |
| 39340 | Co-codamol 8mg/500mg caplets (Vantage) |
| 39461 | Solpadeine Migraine Ibuprofen & Codeine tablets (Omega Pharma Ltd) |
| 40385 | Co-codamol 8mg/500mg effervescent tablets (Almus Pharmaceuticals Ltd) |
| 40662 | Co-codamol 8mg/500mg effervescent tablets (Actavis UK Ltd) |
| 40663 | Co-codamol 30mg/500mg effervescent tablets (Actavis UK Ltd) |
| 41214 | Codeine 15mg/5ml Oral solution (Thornton & Ross Ltd) |
| 41259 | Co-codamol 30mg/500mg effervescent tablets (Teva UK Ltd) |
| 41275 | Co-codamol 8mg+500mg Tablet (Nucare Plc) |
| 41276 | Co-codamol 8mg/500mg tablets (Almus Pharmaceuticals Ltd) |
| 41416 | Codeine 60mg tablets (Wockhardt UK Ltd) |
| 41523 | Codeine Oral solution (Family Health) |
| 41535 | Codeine 30mg tablets (Teva UK Ltd) |
| 41599 | Codeine 15mg tablets (Teva UK Ltd) |
| 41682 | Co-codamol 30mg+500mg Effervescent tablet (Roche Consumer Health) |
| 42213 | Co-codamol 8mg/500mg capsules (Actavis UK Ltd) |
| 42218 | Co-codaprin 8mg/400mg dispersible tablets (A A H Pharmaceuticals Ltd) |
| 42706 | Feminax Period Pain capsules (Bayer Plc) |
| 42791 | Co-codamol 30mg/500mg tablets (Sandoz Ltd) |
| 42792 | Codeine 15mg/5ml Oral solution (Actavis UK Ltd) |
| 43238 | Co-codamol 30mg/500mg effervescent tablets (Mylan) |
| 43244 | Co-codamol 30mg+500mg Effervescent tablet (Hillcross Pharmaceuticals Ltd) |
| 43414 | Co-codamol 8mg/500mg effervescent tablets (Bayer Plc) |
| 43504 | Codeine phosphate Oral solution (William Ransom) |
| 43550 | Codeine 15mg tablets (Ranbaxy (UK) Ltd) |
| 44159 | Solpadeine Max soluble tablets (Omega Pharma Ltd) |
| 44210 | Kapake 15mg/500mg tablets (Galen Ltd) |
| 44924 | Co-codamol 30mg/500mg tablets (Teva UK Ltd) |
| 46511 | Co-codamol 15mg/500mg effervescent tablets sugar free |
| 46633 | Co-codamol 8mg/500mg capsules (A A H Pharmaceuticals Ltd) |
| 46729 | Co-codamol 15mg/500mg capsules |
| 46898 | Codipar 15mg/500mg capsules (AMCo) |
| 46906 | Co-codamol 8mg+500mg Effervescent tablet (Numark Management Ltd) |
| 46925 | Co-codaprin 8mg/400mg dispersible tablets (Actavis UK Ltd) |
| 46987 | Co-codamol 15mg+500mg Tablet (Hillcross Pharmaceuticals Ltd) |
| 47003 | Codeine 60mg tablets (Ranbaxy (UK) Ltd) |
| 47081 | Paracetamol 500mg with codeine phosphate 12.8mg & caffeine 30mg effervescent tablet |
| 47200 | Propain Plus caplets (Ceuta Healthcare Ltd) |
| 47508 | Codipar 15mg/500mg effervescent tablets (AMCo) |
| 47847 | Co-codamol 30mg/500mg capsules (Zentiva) |
| 47919 | Codeine phosphate 15mg Tablet (Wockhardt UK Ltd) |
| 47952 | Codeine phosphate 30mg Tablet (Wockhardt UK Ltd) |
| 48004 | Codeine 30mg tablets (Ranbaxy (UK) Ltd) |
| 48066 | Codeine phosphate oral solution |
| 48136 | Codeine 15mg tablets (Almus Pharmaceuticals Ltd) |
| 48153 | Codeine 60mg tablets (Actavis UK Ltd) |
| 48311 | Co-codamol 30mg/500mg caplets (Kent Pharmaceuticals Ltd) |
| 48775 | Co-codamol 30mg/500mg caplets (AMCo) |
| 48964 | Codeine 3mg/5ml linctus paediatric |
| 50421 | Codeine phosphate 15mg Tablet (Celltech Pharma Europe Ltd) |
| 50468 | Codeine 15mg tablets (Alliance Healthcare (Distribution) Ltd) |
| 50659 | Codeine 60mg tablets (A A H Pharmaceuticals Ltd) |
| 51084 | Co-codamol 30mg/500mg capsules (AMCo) |
| 51327 | Codeine 30mg tablets (Alliance Healthcare (Distribution) Ltd) |
| 51381 | Generic Solpadeine Plus capsules |
| 51644 | Codeine 30mg tablets (Bristol Laboratories Ltd) |
| 51819 | Co-codamol 8mg/500mg capsules (Phoenix Healthcare Distribution Ltd) |
| 51937 | Codeine 15mg tablets (Bristol Laboratories Ltd) |
| 52085 | Co-codamol 30mg/500mg effervescent tablets (Zanza Laboratories Ltd) |
| 52856 | Co-codaprin 8mg/400mg tablets |
| 52888 | Codeine 15mg tablets (Kent Pharmaceuticals Ltd) |
| 52889 | Codeine 25mg/5ml oral solution (A A H Pharmaceuticals Ltd) |
| 52929 | Codeine 15mg tablets (Sigma Pharmaceuticals Plc) |
| 52966 | Generic Solpadeine Plus tablets |
| 53287 | Co-codamol 30mg/500mg effervescent tablets (Alliance Healthcare (Distribution) Ltd) |
| 53600 | Codeine 60mg tablets (Alliance Healthcare (Distribution) Ltd) |
| 53617 | Ibuprofen and codeine 200mg+12.8mg Tablet (Almus Pharmaceuticals Ltd) |
| 53679 | Co-codamol 30mg/500mg effervescent tablets (A A H Pharmaceuticals Ltd) |
| 53702 | Co-codamol 30mg/500mg tablets (Actavis UK Ltd) |
| 53999 | Codeine phosphate 60mg Tablet (Wockhardt UK Ltd) |
| 55044 | Co-codamol 30mg/500mg caplets (Waymade Healthcare Plc) |
| 55309 | Codeine 15mg tablets (Phoenix Healthcare Distribution Ltd) |
| 55465 | Co-codamol 30mg/500mg capsules (Sigma Pharmaceuticals Plc) |
| 56006 | Co-codamol 8mg/500mg tablets (Bristol Laboratories Ltd) |
| 56171 | Co-codamol 30mg/500mg capsules (Kent Pharmaceuticals Ltd) |
| 56178 | Codeine 60mg/1ml solution for injection ampoules (A A H Pharmaceuticals Ltd) |
| 56205 | Boots Paracetamol and Codeine Extra capsules (The Boots Company Plc) |
| 56266 | Co-codamol 30mg/500mg tablets (Alliance Healthcare (Distribution) Ltd) |
| 56340 | Co-codamol 30mg/500mg caplets (J M McGill Ltd) |
| 56461 | Co-codamol 30mg/500mg tablets (Kent Pharmaceuticals Ltd) |
| 56549 | Co-codamol 8mg/500mg effervescent tablets (Waymade Healthcare Plc) |
| 56559 | Codeine 30mg tablets (Phoenix Healthcare Distribution Ltd) |
| 56565 | Co-codamol 15mg/500mg tablets (A A H Pharmaceuticals Ltd) |
| 56817 | Codeine 15mg tablets (Almus Pharmaceuticals Ltd) |
| 57097 | Co-codamol 30mg/500mg capsules (Alliance Healthcare (Distribution) Ltd) |
| 57353 | Co-codamol 30mg/500mg caplets (DE Pharmaceuticals) |
| 57381 | Codeine 60mg tablets (Teva UK Ltd) |
| 57465 | Co-codamol 8mg/500mg caplets (DE Pharmaceuticals) |
| 57487 | Codeine 30mg tablets (Waymade Healthcare Plc) |
| 57752 | Codeine 10mg/5ml oral solution |
| 57839 | Co-codamol 15mg/500mg tablets (Waymade Healthcare Plc) |
| 57865 | Co-codamol 30mg/500mg caplets (Actavis UK Ltd) |
| 57900 | Co-codamol 30mg/500mg caplets (A A H Pharmaceuticals Ltd) |
| 57929 | Co-codamol 8mg/500mg capsules (Waymade Healthcare Plc) |
| 58131 | Codeine 15mg/5ml linctus sugar free (DE Pharmaceuticals) |
| 58288 | Co-codamol 30mg/500mg capsules (DE Pharmaceuticals) |
| 58501 | Co-codamol 30mg/500mg caplets (AM Distributions (Yorkshire) Ltd) |
| 58636 | Co-codamol 8mg/500mg effervescent tablets (Teva UK Ltd) |
| 58855 | Co-codamol 30mg/500mg capsules (Waymade Healthcare Plc) |
| 58909 | Codeine 15mg/5ml linctus sugar free (Kent Pharmaceuticals Ltd) |
| 59131 | Co-codamol 8mg/500mg capsules (Bayer Plc) |
| 59442 | Co-codamol 30mg/500mg caplets (Phoenix Healthcare Distribution Ltd) |
| 59479 | Co-codamol 30mg/500mg capsules (Actavis UK Ltd) |
| 59705 | Co-codamol 8mg/500mg capsules (DE Pharmaceuticals) |
| 59986 | Co-codamol 30mg/500mg tablets (M & A Pharmachem Ltd) |
| 60040 | Generic Migraleve Pink tablets |
| 60489 | Codeine 30mg suppositories |
| 60517 | Co-codamol 30mg/500mg effervescent tablets (Almus Pharmaceuticals Ltd) |
| 60640 | Codeine 15mg tablets (Waymade Healthcare Plc) |
| 60958 | Codeine 15mg tablets (DE Pharmaceuticals) |
| 61049 | Codeine 15mg/5ml linctus (Waymade Healthcare Plc) |
| 61091 | Codeine 60mg tablets (Waymade Healthcare Plc) |
| 61647 | Co-codamol 15mg/500mg tablets (Alliance Healthcare (Distribution) Ltd) |
| 62169 | Co-codamol 8mg/500mg tablets (Vantage) |
| 62228 | Codeine 30mg tablets (DE Pharmaceuticals) |
| 63547 | Codeine 1mg suppositories |
| 63551 | Co-codamol 8mg/500mg effervescent tablets (Vantage) |
| 63600 | Codeine phosphate powder (Martindale Pharmaceuticals Ltd) |
| 63658 | Co-codaprin 400/8 Tablet (Hillcross Pharmaceuticals Ltd) |
| 63683 | Aspirin with codeine Dispersible tablet (Actavis UK Ltd) |
| 63900 | Co-codamol 8mg/500mg tablets (DE Pharmaceuticals) |
| 64108 | Codeine 15mg tablets (Crescent Pharma Ltd) |
| 64387 | Co-codamol 30mg/500mg tablets (DE Pharmaceuticals) |
| 64545 | Co-codamol 8mg/500mg tablets (Aspar Pharmaceuticals Ltd) |
| 64726 | Co-codamol 15mg/500mg tablets (Galen Ltd) |
| 64751 | Codeine 60mg/1ml solution for injection ampoules (Alliance Healthcare (Distribution) Ltd) |
| 64752 | Codeine 30mg tablets (Sigma Pharmaceuticals Plc) |
| 65092 | Co-codamol 30mg/500mg caplets (Mawdsley-Brooks & Company Ltd) |
| 65118 | Codeine 30mg tablets (Crescent Pharma Ltd) |
| 65245 | Codeine 30mg tablets (Mawdsley-Brooks & Company Ltd) |
| 65269 | Codeine 60mg tablets (DE Pharmaceuticals) |
| 65314 | Co-codamol 8mg/500mg caplets (Actavis UK Ltd) |
| 65440 | Co-codamol 30mg/500mg tablets (Mylan) |
| 65806 | Co-codamol 8mg/500mg tablets (Alliance Healthcare (Distribution) Ltd) |
| 65904 | Co-codamol 8mg/500mg capsules (Sigma Pharmaceuticals Plc) |
| 66115 | Codeine 60mg tablets (Kent Pharmaceuticals Ltd) |
| 66352 | Co-codamol 8mg/500mg caplets (Kent Pharmaceuticals Ltd) |
| 66538 | Co-codamol 8mg/500mg capsules (Alliance Healthcare (Distribution) Ltd) |
| 66553 | Co-codamol 30mg/500mg effervescent tablets (AMCo) |
| 66602 | Generic Solpadeine Max soluble tablets sugar free |
| 66807 | Paracetamol with codeine phosphate peadiatric elixir |
| 66893 | Codeine 15mg tablets (Mawdsley-Brooks & Company Ltd) |
| 66904 | Co-codamol 8mg/500mg caplets (Wockhardt UK Ltd) |
| 67106 | Co-codamol 30mg/500mg caplets (Wockhardt UK Ltd) |
| 67751 | Codeine 30mg tablets (Almus Pharmaceuticals Ltd) |
| 67753 | Co-codamol 8mg/500mg tablets (Wockhardt UK Ltd) |
| 68252 | Co-codamol 30mg/500mg tablets (Bristol Laboratories Ltd) |
| 68509 | Codeine phosphate 30mg Tablet (Celltech Pharma Europe Ltd) |
| 68538 | Codeine 30mg/5ml oral solution |
| 68562 | Generic Syndol caplets |
| 68861 | Codeine 25mg/5ml oral solution (DE Pharmaceuticals) |
| 69066 | Codeine 5mg/5ml oral suspension |
| 69285 | Boots Ibuprofen and Codeine 200mg/12.8mg tablets (The Boots Company Plc) |
| 69304 | Co-codamol 15mg/500mg tablets (Actavis UK Ltd) |
| 69576 | Codeine 10mg/5ml oral suspension |
| 70497 | Co-codamol 15mg/500mg capsules (A A H Pharmaceuticals Ltd) |
| 70518 | Co-codamol 60mg/1000mg tablets |
| 70552 | Codeine 60mg/1ml solution for injection ampoules (Torbay Pharmaceuticals) |
| 71227 | Syndol Headache Relief tablets (Sanofi) |
| 71492 | Codeine 25mg/5ml oral solution (Alliance Healthcare (Distribution) Ltd) |
| 71614 | Co-codamol 15mg/500mg tablets (Zentiva) |
| 71987 | Codeine 15mg/5ml linctus (Bell,Sons & Co (Druggists) Ltd) |
| 72389 | Co-codamol 15mg/500mg capsules (Alliance Healthcare (Distribution) Ltd) |
| 72438 | Codeine 15mg tablets (Sun Pharmaceuticals UK Ltd) |
| 4 | Co-proxamol 32.5mg/325mg tablets |
| 124 | Distalgesic 32.5mg/325mg tablets (Meda Pharmaceuticals Ltd) |
| 483 | Co-proxamol (dextropropoxyphene and paracetamol) 32.5mg with 325mg/5ml oral suspension sugar free |
| 1762 | Dextropropoxyphene HCl with paracetamol 32.5mg with 325mg tablets |
| 2462 | Cosalgesic Tablet (Actavis UK Ltd) |
| 3714 | DEXTROPROPOXYPHENE NAPSYLATE/PARACETAMOL MG TAB |
| 4607 | Co-proxamol 32.5/325 Tablet (Dista Products Ltd) |
| 12076 | Dextropropoxyphene 60mg capsules |
| 18482 | Paracetamol 325mg with dextropropoxyphene 32.5mg tablet |
| 19069 | Dextropropoxyphene 60mg capsules |
| 21067 | Doloxene Capsule (Eli Lilly and Company Ltd) |
| 24733 | DEXTROPROPOXYPHENE HCl S/R 150 MG CAP |
| 25959 | ASPIRIN/CAFFEINE/DEXTROPROPOXYPHENE NAPS PUL |
| 25979 | Doloxene 60mg Capsule (Eli Lilly and Company Ltd) |
| 28253 | Co-proxamol 32.5/325 Oral suspension (Rosemont Pharmaceuticals Ltd) |
| 30954 | Co-proxamol 32.5mg/325mg tablets (Actavis UK Ltd) |
| 30966 | Co-proxamol 32.5/325 Tablet (Dista Products Ltd) |
| 33647 | Co-proxamol 32.5mg/325mg tablets (A A H Pharmaceuticals Ltd) |
| 33995 | Co-proxamol 32.5/325 Tablet (Berk Pharmaceuticals Ltd) |
| 34022 | Co-proxamol 32.5mg/325mg tablets (Teva UK Ltd) |
| 34319 | Co-proxamol 32.5mg/325mg tablets (Zentiva) |
| 34349 | Co-proxamol 32.5mg/325mg tablets (IVAX Pharmaceuticals UK Ltd) |
| 34397 | Co-proxamol 32.5mg+325mg Tablet (M & A Pharmachem Ltd) |
| 34468 | Co-proxamol 32.5mg/325mg tablets (Sandoz Ltd) |
| 34546 | Co-proxamol 32.5mg+325mg Tablet (Neo Laboratories Ltd) |
| 34554 | Co-proxamol 32.5/325 Tablet (C P Pharmaceuticals Ltd) |
| 34597 | Co-proxamol 32.5mg/325mg tablets (Kent Pharmaceuticals Ltd) |
| 41407 | Co-proxamol 32.5mg/325mg tablets (Ranbaxy (UK) Ltd) |
| 43536 | Co-proxamol 32.5/325 Tablet (Numark Management Ltd) |
| 43891 | Co-proxamol 32.5mg/325mg/5ml oral suspension |
| 44311 | DEXTROPROPOXYPHENE NAPSYLATE/ASPIRIN 100 MG TAB |
| 45231 | Co-proxamol 32.5mg+325mg Tablet (Regent Laboratories Ltd) |
| 45276 | Co-proxamol 32.5mg+325mg Tablet (Sigma Pharmaceuticals Plc) |
| 53208 | Co-proxamol 32.5mg/325mg tablets (Lexon (UK) Ltd) |
| 55245 | Co-proxamol 32.5mg/325mg tablets (Phoenix Healthcare Distribution Ltd) |
| 67755 | Co-proxamol 32.5mg/325mg tablets (Almus Pharmaceuticals Ltd) |
| 69043 | Co-proxamol 32.5mg/325mg tablets (DE Pharmaceuticals) |
| 69534 | Co-proxamol 32.5mg/325mg tablets (Alliance Healthcare (Distribution) Ltd) |
| 11 | Co-dydramol 10mg/500mg tablets |
| 53 | Dihydrocodeine 30mg tablets |
| 191 | Dihydrocodeine 10mg/5ml oral solution |
| 2040 | Remedeine tablets (Crescent Pharma Ltd) |
| 2041 | Dihydrocodeine 60mg modified-release tablets |
| 2555 | Dihydrocodeine with paracetamol 10mg+500mg tablets |
| 3653 | Dihydrocodeine 50mg/1ml solution for injection ampoules |
| 3698 | Df118 40mg Tablet (Martindale Pharmaceuticals Ltd) |
| 4556 | Paramol tablets (SSL International Plc) |
| 4823 | Dihydrocodeine 40mg tablets |
| 4950 | Dihydrocodeine with paracetamol 30mg+500mg tablets |
| 5955 | Paracetamol 500mg / Dihydrocodeine 30mg tablets |
| 6234 | Dihydrocodeine 120mg modified-release tablets |
| 7063 | Co-dydramol 10mg/500mg/5ml oral suspension |
| 7469 | Df118 10mg/5ml Oral solution (Martindale Pharmaceuticals Ltd) |
| 7989 | DIHYDROCODEINE TARTRATE/ASPIRIN 300 MG TAB |
| 8456 | DHC Continus 60mg tablets (Napp Pharmaceuticals Ltd) |
| 9163 | Remedeine 20mg+500mg Effervescent tablet (Napp Pharmaceuticals Ltd) |
| 9209 | DHC Continus 90mg tablets (Napp Pharmaceuticals Ltd) |
| 9275 | DHC Continus 120mg tablets (Napp Pharmaceuticals Ltd) |
| 9313 | Dihydrocodeine 90mg modified-release tablets |
| 9562 | Remedeine 30mg+500mg Effervescent tablet (Napp Pharmaceuticals Ltd) |
| 9785 | Remedeine 30mg+500mg Tablet (Napp Pharmaceuticals Ltd) |
| 9855 | Paracetamol 500mg / Dihydrocodeine 20mg tablets |
| 10023 | Dihydrocodeine with paracetamol 20mg+500mg tablets |
| 10122 | Dihydrocodeine 10mg with paracetamol 500mg/5ml oral suspension sugar free |
| 10309 | Df118 50mg/ml Injection (Martindale Pharmaceuticals Ltd) |
| 14688 | Paracetamol 500mg with dihydrocodeine 10mg tablet |
| 15198 | Co-dydramol 10mg+500mg Tablet (C P Pharmaceuticals Ltd) |
| 16112 | Paramol soluble tablets (SSL International Plc) |
| 17917 | Dihydrocodeine with paracetamol 20mg with 500mg effervescent tablets |
| 19206 | Paracetamol 500mg / Dihydrocodeine 7.46mg tablets |
| 21113 | Dihydrocodeine with paracetamol forte 30mg with 500mg effervescent tablets |
| 21229 | Paracetamol 500mg / Dihydrocodeine 7.46mg effervescent tablets sugar free |
| 26653 | DIHYDROCODEINE 10mg/PARACETAMOL 500mg |
| 28598 | Paracetamol with dihydrocodeine 500mg +10mg/5ml suspension sugar free |
| 28780 | Co-dydramol 10mg/500mg tablets (A A H Pharmaceuticals Ltd) |
| 30165 | Co-dydramol (dihydrocodeine and paracetamol) 7.46mg with 500mg tablets |
| 30295 | Dihydrocodeine with paracetamol 7.46mg+500mg tablets |
| 30444 | Co-dydramol 10mg/500mg tablets (Mylan) |
| 32926 | Co-dydramol 10mg/500mg tablets (Actavis UK Ltd) |
| 33340 | Co-dydramol 10mg+500mg/5ml Liquid (Rosemont Pharmaceuticals Ltd) |
| 33654 | Dihydrocodeine 30mg tablets (Wockhardt UK Ltd) |
| 33743 | Dihydrocodeine with paracetamol 7.46mg with 500mg effervescent tablets |
| 34008 | Dihydrocodeine 30mg tablets (IVAX Pharmaceuticals UK Ltd) |
| 34440 | Dihydrocodeine 30mg tablets (A A H Pharmaceuticals Ltd) |
| 34579 | Dihydrocodeine 30mg tablets (Actavis UK Ltd) |
| 34662 | Dihydrocodeine 30mg tablets (Mylan) |
| 34730 | Dihydrocodeine 30mg Tablet (Berk Pharmaceuticals Ltd) |
| 34737 | Co-dydramol 10mg/500mg tablets (Teva UK Ltd) |
| 34920 | Co-dydramol 10mg+500mg Tablet (Berk Pharmaceuticals Ltd) |
| 34939 | Co-dydramol 10mg+500mg Tablet (Duncan Flockhart Ltd) |
| 36019 | Co-dydramol 10mg+500mg Tablet (M & A Pharmachem Ltd) |
| 37291 | Co-dydramol 10mg/500mg tablets (Ranbaxy (UK) Ltd) |
| 38430 | Co-dydramol 10mg+500mg Tablet (Merck Generics (UK) Ltd) |
| 38521 | Dihydrocodeine 30mg tablets (Teva UK Ltd) |
| 38950 | Remedeine Forte tablets (Crescent Pharma Ltd) |
| 38970 | DF 118 Forte 40mg tablets (Martindale Pharmaceuticals Ltd) |
| 39558 | Dihydrocodeine 30mg tablets (Zentiva) |
| 40159 | Dihydrocodeine 10mg/5ml oral solution (Martindale Pharmaceuticals Ltd) |
| 40422 | Co-dydramol 10mg/500mg tablets (Zentiva) |
| 41278 | Co-dydramol 10mg/500mg tablets (IVAX Pharmaceuticals UK Ltd) |
| 42208 | Df118 30mg Tablet (Martindale Pharmaceuticals Ltd) |
| 43441 | Co-dydramol 10mg+500mg Tablet (Celltech Pharma Europe Ltd) |
| 47071 | Co-dydramol (dihydrocodeine and paracetamol) 10mg with 500mg/5ml oral suspension |
| 48133 | Dihydrocodeine 30mg tablets (Almus Pharmaceuticals Ltd) |
| 50532 | Dihydrocodeine 30mg tablets (Bristol Laboratories Ltd) |
| 53079 | Co-dydramol 10mg/500mg tablets (Sigma Pharmaceuticals Plc) |
| 54354 | Dihydrocodeine 30mg tablets (Kent Pharmaceuticals Ltd) |
| 54713 | Dypracet 20mg/500mg tablets (Auden McKenzie (Pharma Division) Ltd) |
| 55425 | Dihydrocodeine 10mg tablets |
| 55530 | Co-dydramol 10mg/500mg tablets (Phoenix Healthcare Distribution Ltd) |
| 57197 | Co-dydramol 10mg/500mg tablets (Waymade Healthcare Plc) |
| 58848 | Dypracet 30mg/500mg tablets (Auden McKenzie (Pharma Division) Ltd) |
| 59978 | Dihydrocodeine 30mg tablets (Waymade Healthcare Plc) |
| 59989 | Dihydrocodeine 30mg tablets (Ranbaxy (UK) Ltd) |
| 61372 | Co-dydramol 10mg/500mg tablets (Kent Pharmaceuticals Ltd) |
| 61698 | Co-dydramol 10mg/500mg/5ml oral solution |
| 62635 | Co-dydramol 10mg/500mg tablets (Alliance Healthcare (Distribution) Ltd) |
| 64074 | Co-dydramol 10mg/500mg tablets (DE Pharmaceuticals) |
| 64079 | Dihydrocodeine 10mg/5ml oral solution (Waymade Healthcare Plc) |
| 64368 | Paracetamol 500mg / Dihydrocodeine 30mg tablets (Icarus Pharmaceuticals Ltd) |
| 65035 | Co-dydramol 10mg/500mg tablets (Almus Pharmaceuticals Ltd) |
| 65689 | Dihydrocodeine 30mg/5ml oral solution |
| 66121 | Dihydrocodeine 10mg/5ml oral suspension |
| 67779 | Co-dydramol 10mg/500mg tablets (Wockhardt UK Ltd) |
| 69006 | Co-dydramol 10mg/500mg/5ml oral suspension sugar free |
| 71006 | Co-dydramol 10mg/500mg oral powder sachets (Special Order) |
| 71523 | Co-dydramol 20mg/500mg tablets (M & A Pharmachem Ltd) |
| 71566 | Co-dydramol 30mg/500mg tablets (M & A Pharmachem Ltd) |
| 72110 | Co-dydramol 30mg/500mg tablets (A A H Pharmaceuticals Ltd) |
| 72111 | Co-dydramol 20mg/500mg tablets (A A H Pharmaceuticals Ltd) |
| 72209 | Dihydrocodeine 30mg tablets (DE Pharmaceuticals) |
| 72210 | Dihydrocodeine 30mg tablets (Alliance Healthcare (Distribution) Ltd) |
| 72258 | Co-dydramol 30mg/500mg tablets (Alliance Healthcare (Distribution) Ltd) |
| 72265 | Dihydrocodeine 15mg/5ml oral solution |
| 72282 | Co-dydramol 20mg/500mg tablets (Alliance Healthcare (Distribution) Ltd) |
| 1708 | Codafen Continus tablets (Napp Pharmaceuticals Ltd) |
| 10519 | CODEINE PHOS/IBUPROFEN SR (20MG/300MG) TAB |
| 11461 | Ibuprofen 300mg modified-release / Codeine 20mg tablets |
| 11554 | Ibuprofen 200mg / Codeine 12.8mg tablets |
| 12709 | Ibuprofen and codeine 200mg + 12.5mg Tablet |
| 13893 | Nurofen Plus tablets (Reckitt Benckiser Healthcare (UK) Ltd) |
| 25330 | Solpaflex tablets (GlaxoSmithKline Consumer Healthcare) |
| 37816 | Cuprofen PLUS tablets (SSL International Plc) |
| 39461 | Solpadeine Migraine Ibuprofen & Codeine tablets (Omega Pharma Ltd) |
| 53617 | Ibuprofen and codeine 200mg+12.8mg Tablet (Almus Pharmaceuticals Ltd) |
| 69285 | Boots Ibuprofen and Codeine 200mg/12.8mg tablets (The Boots Company Plc) |
| 3239 | Meptazinol 200mg tablets |
| 8447 | Meptid 200mg Tablet (Shire Pharmaceuticals Ltd) |
| 10925 | Meptid 100mg/1ml solution for injection ampoules (Almirall Ltd) |
| 11801 | Meptazinol 100mg/1ml solution for injection ampoules |
| 39842 | Meptid 200mg tablets (Almirall Ltd) |
| 4 | Co-proxamol 32.5mg/325mg tablets |
| 11 | Co-dydramol 10mg/500mg tablets |
| 19 | Co-codamol 8mg/500mg tablets |
| 57 | Co-codamol 8mg/500mg effervescent tablets |
| 96 | Co-codamol 30mg/500mg tablets |
| 124 | Distalgesic 32.5mg/325mg tablets (Meda Pharmaceuticals Ltd) |
| 483 | Co-proxamol (dextropropoxyphene and paracetamol) 32.5mg with 325mg/5ml oral suspension sugar free |
| 625 | Co-codamol 8mg/500mg capsules |
| 656 | Tylex 30mg/500mg capsules (UCB Pharma Ltd) |
| 767 | Solpadol 30mg/500mg capsules (Sanofi) |
| 800 | Co-codamol 30mg/500mg capsules |
| 810 | Co-codamol 30mg/500mg effervescent tablets |
| 1261 | Co-codamol 30mg/500mg effervescent powder sachets sugar free |
| 1616 | Migraleve Pink tablets (McNeil Products Ltd) |
| 1640 | Kapake 30mg/500mg tablets (Galen Ltd) |
| 1762 | Dextropropoxyphene HCl with paracetamol 32.5mg with 325mg tablets |
| 2040 | Remedeine tablets (Crescent Pharma Ltd) |
| 2211 | Solpadol 30mg/500mg effervescent tablets (Sanofi) |
| 2250 | CODEINE & PARACETAMOL TAB |
| 2462 | Cosalgesic Tablet (Actavis UK Ltd) |
| 2555 | Dihydrocodeine with paracetamol 10mg+500mg tablets |
| 2698 | CODEINE & PARACETAMOL 8 MG TAB |
| 2794 | Co-codamol 30mg/500mg tablets (Wockhardt UK Ltd) |
| 2846 | Paracetamol 500mg with codeine phosphate 30mg effervescent tablet |
| 2917 | Paracetamol 500mg with codeine phosphate 30 mg tablet |
| 3156 | Solpadol 30mg/500mg caplets (Sanofi) |
| 3185 | Paracetamol 500mg with codeine phosphate 30mg capsule |
| 3435 | Tylex 30mg/500mg effervescent tablets (UCB Pharma Ltd) |
| 3713 | Medocodene Tablet (Manufacturer unknown) |
| 3714 | DEXTROPROPOXYPHENE NAPSYLATE/PARACETAMOL MG TAB |
| 3724 | Migraleve Yellow tablets (McNeil Products Ltd) |
| 4349 | PARACETAMOL & CODEINE TAB |
| 4556 | Paramol tablets (SSL International Plc) |
| 4607 | Co-proxamol 32.5/325 Tablet (Dista Products Ltd) |
| 4671 | Codeine phosphate 30mg with Paracetamol 500mg capsules |
| 4718 | Migraleve - 1 Tablet (Pfizer Consumer Healthcare Ltd) |
| 4950 | Dihydrocodeine with paracetamol 30mg+500mg tablets |
| 5498 | Propain caplet (Sankyo Pharma UK Ltd) |
| 5685 | Syndol easy to swallow Tablet (SSL International Plc) |
| 5955 | Paracetamol 500mg / Dihydrocodeine 30mg tablets |
| 6665 | Paracetamol with codeine & buclizine tablet |
| 6886 | Codeine phosphate 30mg with paracetamol 500mg tablets |
| 7063 | Co-dydramol 10mg/500mg/5ml oral suspension |
| 7072 | Co-codamol 15mg/500mg tablets |
| 7104 | Kapake 30mg/500mg effervescent tablets (Galen Ltd) |
| 7499 | Calpol extra Tablet (Warner Lambert UK Ltd) |
| 7542 | Codeine phosphate 8mg with paracetamol 500mg tablets |
| 7696 | PARACETAMOL 500MG/CODEINE 10MG MG TAB |
| 7770 | ASPIRIN/CODEINE PHOSPHATE/PARACETAMOL 250 MG TAB |
| 7976 | PARACETAMOL 450MG/CODEINE 8.1MG TAB |
| 8246 | Codeine phosphate 8mg with paracetamol 500mg effervescent tablets |
| 8329 | Solpadeine Tablet (GlaxoSmithKline Consumer Healthcare) |
| 8335 | Paracodol 8mg/500mg capsules (Bayer Plc) |
| 8500 | CODEINE PHOSPHATE/PARACETAMOL/SODIUM CIT 8 MG CAP |
| 9129 | Paracetamol 250mg with aspirin 250mg & codeine 6.8mg tablet |
| 9163 | Remedeine 20mg+500mg Effervescent tablet (Napp Pharmaceuticals Ltd) |
| 9457 | Paracetamol 500mg with codeine phosphate 8mg tablet |
| 9460 | Paracetamol 500mg with codeine phosphate 8mg capsule |
| 9462 | Paracetamol 500mg with codeine phosphate 8mg effervescent tablet |
| 9516 | Kapake 30mg/500mg capsules (Galen Ltd) |
| 9562 | Remedeine 30mg+500mg Effervescent tablet (Napp Pharmaceuticals Ltd) |
| 9742 | Solpadeine Soluble tablet (GlaxoSmithKline Consumer Healthcare) |
| 9785 | Remedeine 30mg+500mg Tablet (Napp Pharmaceuticals Ltd) |
| 9855 | Paracetamol 500mg / Dihydrocodeine 20mg tablets |
| 9917 | Kapake Insts 30mg/500mg effervescent powder sachets (Galen Ltd) |
| 10023 | Dihydrocodeine with paracetamol 20mg+500mg tablets |
| 10099 | Paracetamol 500mg with codeine phosphate 8mg & buclizine 6.25mg |
| 10122 | Dihydrocodeine 10mg with paracetamol 500mg/5ml oral suspension sugar free |
| 10176 | Codipar 15mg/500mg tablets (AMCo) |
| 10178 | Solpadeine Plus capsules (Omega Pharma Ltd) |
| 10226 | Solpadeine Plus tablets (Omega Pharma Ltd) |
| 10582 | Formulix Oral solution (Cilag Pharmaceuticals Ltd) |
| 10602 | Paracodol 8mg/500mg effervescent tablets (Bayer Plc) |
| 11009 | Paracetamol 500mg with codeine phosphate 8mg & caffeine 30mg tablet |
| 11250 | Migraleve - 2 8mg+500mg Tablet (Pfizer Consumer Healthcare Ltd) |
| 11325 | Paracetamol 500mg with codeine phosphate 30 mg tablet |
| 11665 | Zapain 30mg/500mg tablets (AMCo) |
| 11807 | Paracetamol 500mg with codeine 12.8mg tablet |
| 11945 | Syndol Tablet (SSL International Plc) |
| 11961 | Veganin Tablet (Pfizer Consumer Healthcare Ltd) |
| 14378 | Feminax tablets (Bayer Plc) |
| 14602 | Co-codamol 60mg/1000mg effervescent powder sachets sugar free |
| 14676 | Paracetamol 500mg with codeine phosphate 8mg & caffeine 30mg effervescent tablet |
| 14688 | Paracetamol 500mg with dihydrocodeine 10mg tablet |
| 14785 | Paracetamol500mg with codeine phosphate 15mg tablet |
| 14912 | Codeine phosphate 8mg with Paracetamol 500mg capsules |
| 14964 | Solpadeine Plus soluble tablets (Omega Pharma Ltd) |
| 15198 | Co-dydramol 10mg+500mg Tablet (C P Pharmaceuticals Ltd) |
| 15831 | Codeine phosphate 30mg with paracetamol 500mg effervescent powder sugar free |
| 15871 | Kapake Insts 60mg/1000mg effervescent powder sachets (Galen Ltd) |
| 16039 | Solpadeine Capsule (GlaxoSmithKline Consumer Healthcare) |
| 16112 | Paramol soluble tablets (SSL International Plc) |
| 16467 | Codeine phosphate 30mg with paracetamol 500mg effervescent tablets |
| 16592 | Parahypon Tablet (Wellcome Consumer) |
| 16818 | Paracetamol 500mg with codeine phosphate 30mg effervescent powder sugar free |
| 17158 | Panadeine forte Tablet (Sanofi-Synthelabo Ltd) |
| 17563 | Solpadeine Max 12.8mg/500mg tablets (Omega Pharma Ltd) |
| 17917 | Dihydrocodeine with paracetamol 20mg with 500mg effervescent tablets |
| 17998 | Medocodene 30mg/500mg capsules (UCB Pharma Ltd) |
| 18221 | Codeine phosphate 15mg with paracetamol 500mg tablets |
| 18482 | Paracetamol 325mg with dextropropoxyphene 32.5mg tablet |
| 19206 | Paracetamol 500mg / Dihydrocodeine 7.46mg tablets |
| 19854 | Propain plus Tablet (Ceuta Healthcare Ltd) |
| 20077 | Paracetamol 400mg with codeine phosphate 10mg with diphenhydramine 5mg & caffeine 50mg tablet |
| 20256 | Paracetamol 450mg with codeine phosphate 10mg with doxylamine 5mg & caffeine 30mg tablet |
| 20565 | Paracetamol 500mg with codeine phosphate 8mg & caffeine 30mg capsule |
| 20853 | Propain Tablet (Sankyo Pharma UK Ltd) |
| 21104 | PARACETAMOL/CODEINE PHOSPHATE 500 MG TAB |
| 21113 | Dihydrocodeine with paracetamol forte 30mg with 500mg effervescent tablets |
| 21229 | Paracetamol 500mg / Dihydrocodeine 7.46mg effervescent tablets sugar free |
| 21251 | Ultramol Soluble tablets (Zentiva) |
| 21673 | Paracetamol 500mg with codeine phosphate 8mg & caffeine 30mg effervescent tablet |
| 21703 | Paracetamol 1000mg with codeine phosphate 60mg effervescent powder sugar free |
| 21746 | Pharmidone Tablet (Pharmacia Ltd) |
| 21880 | Zapain 30mg/500mg capsules (AMCo) |
| 22627 | CODEINE PHOSPHATE 15/PARACETAMOL 500MG TAB |
| 22764 | Panadeine Tablet (GlaxoSmithKline UK Ltd) |
| 23420 | Codeine phosphate 60mg with paracetamol 1000mg effervescent powder sugar free |
| 23580 | Codeine phosphate with diphenhydramine with paracetamol with phenylephrine with caffeine with vitamin c tablets |
| 23952 | Panadeine Effervescent tablet (Sanofi-Synthelabo Ltd) |
| 24187 | Paracetamol with diphenhydramine with codeine with phenylephrine with caffeine & vitamin c tablet |
| 24498 | ASPIRIN/CODEINE PHOSPHATE/PARACETAMOL 300 MG TAB |
| 24859 | CODEINE 8MG & PARACETAMOL 500MG SUP |
| 24996 | Codeine phosphate 8mg with paracetamol 500mg with buclizine 6.25mg tablets |
| 25109 | Veganin tablets (Omega Pharma Ltd) |
| 25514 | Paracetamol 500mg with codeine phosphate 10mg tablet |
| 25529 | Paracetamol 500mg with codeine phosphate 8mg & caffeine 30mg tablet |
| 26291 | Phenylephrine with diphenhydramine, paracetamol, codeine, caffeine & vitamin c tablet |
| 26653 | DIHYDROCODEINE 10mg/PARACETAMOL 500mg |
| 27784 | Co-codamol 8mg/500mg tablets (Actavis UK Ltd) |
| 27785 | Co-codamol 30mg/500mg tablets (Zentiva) |
| 28253 | Co-proxamol 32.5/325 Oral suspension (Rosemont Pharmaceuticals Ltd) |
| 28598 | Paracetamol with dihydrocodeine 500mg +10mg/5ml suspension sugar free |
| 28780 | Co-dydramol 10mg/500mg tablets (A A H Pharmaceuticals Ltd) |
| 29342 | Co-codamol 8mg+500mg Tablet (M & A Pharmachem Ltd) |
| 29373 | Pardale 400mg+9mg+10mg Tablet (Martindale Pharmaceuticals Ltd) |
| 29488 | Co-codamol 8mg/500mg effervescent tablets (Zentiva) |
| 30021 | Paracetamol 500mg with codeine phosphate 13.5mg tablet |
| 30123 | Panadol Ultra 12.8mg/500mg tablets (GlaxoSmithKline Consumer Healthcare) |
| 30165 | Co-dydramol (dihydrocodeine and paracetamol) 7.46mg with 500mg tablets |
| 30295 | Dihydrocodeine with paracetamol 7.46mg+500mg tablets |
| 30444 | Co-dydramol 10mg/500mg tablets (Mylan) |
| 30556 | Co-codamol 8mg/500mg effervescent tablets (Fannin UK Ltd) |
| 30954 | Co-proxamol 32.5mg/325mg tablets (Actavis UK Ltd) |
| 30966 | Co-proxamol 32.5/325 Tablet (Dista Products Ltd) |
| 31155 | Parake Tablet (Galen Ltd) |
| 31577 | Co-codamol 30mg/500mg capsules (A A H Pharmaceuticals Ltd) |
| 31871 | Paracetamol 450mg with codeine phosphate 8.1mg tablet |
| 31894 | Safapryn -Co Tablet (Pfizer Ltd) |
| 32519 | Painex caplets (Sandoz Ltd) |
| 32692 | Co-codamol 8mg/500mg effervescent tablets (A A H Pharmaceuticals Ltd) |
| 32891 | Syndol caplets (Sanofi) |
| 32926 | Co-dydramol 10mg/500mg tablets (Actavis UK Ltd) |
| 33260 | Paracetamol with codeine with hyoscine & caffeine tablet |
| 33340 | Co-dydramol 10mg+500mg/5ml Liquid (Rosemont Pharmaceuticals Ltd) |
| 33643 | Co-codamol 8mg+500mg Tablet (Family Health) |
| 33647 | Co-proxamol 32.5mg/325mg tablets (A A H Pharmaceuticals Ltd) |
| 33653 | Co-codamol 8mg/500mg tablets (IVAX Pharmaceuticals UK Ltd) |
| 33679 | Co-codamol 8mg/500mg tablets (Zentiva) |
| 33688 | Co-codamol 30mg/500mg effervescent tablets (Fannin UK Ltd) |
| 33743 | Dihydrocodeine with paracetamol 7.46mg with 500mg effervescent tablets |
| 33961 | Co-codamol 30mg/500mg capsules (IVAX Pharmaceuticals UK Ltd) |
| 33995 | Co-proxamol 32.5/325 Tablet (Berk Pharmaceuticals Ltd) |
| 34022 | Co-proxamol 32.5mg/325mg tablets (Teva UK Ltd) |
| 34229 | Co-codamol 8mg+500mg Dispersible tablet (Rhone-Poulenc Rorer Ltd) |
| 34257 | Co-codamol 8mg/500mg effervescent tablets (Mylan) |
| 34264 | Co-codamol 30mg/500mg tablets (IVAX Pharmaceuticals UK Ltd) |
| 34319 | Co-proxamol 32.5mg/325mg tablets (Zentiva) |
| 34349 | Co-proxamol 32.5mg/325mg tablets (IVAX Pharmaceuticals UK Ltd) |
| 34397 | Co-proxamol 32.5mg+325mg Tablet (M & A Pharmachem Ltd) |
| 34468 | Co-proxamol 32.5mg/325mg tablets (Sandoz Ltd) |
| 34495 | Co-codamol 8mg/500mg tablets (Mylan) |
| 34497 | Co-codamol 8mg+500mg Tablet (Berk Pharmaceuticals Ltd) |
| 34518 | Co-codamol 8mg/500mg tablets (A A H Pharmaceuticals Ltd) |
| 34546 | Co-proxamol 32.5mg+325mg Tablet (Neo Laboratories Ltd) |
| 34554 | Co-proxamol 32.5/325 Tablet (C P Pharmaceuticals Ltd) |
| 34597 | Co-proxamol 32.5mg/325mg tablets (Kent Pharmaceuticals Ltd) |
| 34667 | Co-codamol 30mg/500mg tablets (A A H Pharmaceuticals Ltd) |
| 34737 | Co-dydramol 10mg/500mg tablets (Teva UK Ltd) |
| 34784 | Co-codamol 8mg/500mg effervescent tablets (Sandoz Ltd) |
| 34815 | Co-codamol 8mg/500mg tablets (Kent Pharmaceuticals Ltd) |
| 34840 | Co-codamol 30mg/500mg tablets (Almus Pharmaceuticals Ltd) |
| 34845 | Co-codamol 30mg/500mg effervescent tablets (Zentiva) |
| 34865 | Co-codamol 8mg+500mg Tablet (C P Pharmaceuticals Ltd) |
| 34920 | Co-dydramol 10mg+500mg Tablet (Berk Pharmaceuticals Ltd) |
| 34939 | Co-dydramol 10mg+500mg Tablet (Duncan Flockhart Ltd) |
| 34968 | Co-codamol 8mg/500mg tablets (Teva UK Ltd) |
| 35965 | Medocodene 30mg/500mg effervescent tablets (Mylan) |
| 36019 | Co-dydramol 10mg+500mg Tablet (M & A Pharmachem Ltd) |
| 36152 | Propain caplets (Ceuta Healthcare Ltd) |
| 36488 | Co-codamol 30mg/500mg capsules (Mylan) |
| 36608 | Co-codamol Effervescent tablet (A A H Pharmaceuticals Ltd) |
| 36993 | Co-codamol 30mg/500mg capsules (Teva UK Ltd) |
| 37291 | Co-dydramol 10mg/500mg tablets (Ranbaxy (UK) Ltd) |
| 37298 | PARACETAMOL,CAFFEINE,CODEINE & NICOTINAM TAB |
| 37348 | Codeine phosphate with paracetamol, doxylamine and caffeine 10mg with 450mg with 5mg with 30mg tablets |
| 37904 | Co-codamol 12.8mg/500mg tablets |
| 38085 | Paracetamol 500mg with codeine phosphate 10mg capsule |
| 38088 | Paracetamol 500mg with codeine phosphate 30 mg tablet |
| 38363 | Codeine phosphate 12.8mg with paracetamol 500mg tablets |
| 38430 | Co-dydramol 10mg+500mg Tablet (Merck Generics (UK) Ltd) |
| 38950 | Remedeine Forte tablets (Crescent Pharma Ltd) |
| 39340 | Co-codamol 8mg/500mg caplets (Vantage) |
| 40385 | Co-codamol 8mg/500mg effervescent tablets (Almus Pharmaceuticals Ltd) |
| 40422 | Co-dydramol 10mg/500mg tablets (Zentiva) |
| 40662 | Co-codamol 8mg/500mg effervescent tablets (Actavis UK Ltd) |
| 40663 | Co-codamol 30mg/500mg effervescent tablets (Actavis UK Ltd) |
| 41259 | Co-codamol 30mg/500mg effervescent tablets (Teva UK Ltd) |
| 41275 | Co-codamol 8mg+500mg Tablet (Nucare Plc) |
| 41276 | Co-codamol 8mg/500mg tablets (Almus Pharmaceuticals Ltd) |
| 41278 | Co-dydramol 10mg/500mg tablets (IVAX Pharmaceuticals UK Ltd) |
| 41407 | Co-proxamol 32.5mg/325mg tablets (Ranbaxy (UK) Ltd) |
| 41682 | Co-codamol 30mg+500mg Effervescent tablet (Roche Consumer Health) |
| 42213 | Co-codamol 8mg/500mg capsules (Actavis UK Ltd) |
| 42706 | Feminax Period Pain capsules (Bayer Plc) |
| 42791 | Co-codamol 30mg/500mg tablets (Sandoz Ltd) |
| 43238 | Co-codamol 30mg/500mg effervescent tablets (Mylan) |
| 43244 | Co-codamol 30mg+500mg Effervescent tablet (Hillcross Pharmaceuticals Ltd) |
| 43414 | Co-codamol 8mg/500mg effervescent tablets (Bayer Plc) |
| 43441 | Co-dydramol 10mg+500mg Tablet (Celltech Pharma Europe Ltd) |
| 43536 | Co-proxamol 32.5/325 Tablet (Numark Management Ltd) |
| 43891 | Co-proxamol 32.5mg/325mg/5ml oral suspension |
| 44159 | Solpadeine Max soluble tablets (Omega Pharma Ltd) |
| 44210 | Kapake 15mg/500mg tablets (Galen Ltd) |
| 44924 | Co-codamol 30mg/500mg tablets (Teva UK Ltd) |
| 45231 | Co-proxamol 32.5mg+325mg Tablet (Regent Laboratories Ltd) |
| 45276 | Co-proxamol 32.5mg+325mg Tablet (Sigma Pharmaceuticals Plc) |
| 46511 | Co-codamol 15mg/500mg effervescent tablets sugar free |
| 46633 | Co-codamol 8mg/500mg capsules (A A H Pharmaceuticals Ltd) |
| 46729 | Co-codamol 15mg/500mg capsules |
| 46898 | Codipar 15mg/500mg capsules (AMCo) |
| 46906 | Co-codamol 8mg+500mg Effervescent tablet (Numark Management Ltd) |
| 46987 | Co-codamol 15mg+500mg Tablet (Hillcross Pharmaceuticals Ltd) |
| 47071 | Co-dydramol (dihydrocodeine and paracetamol) 10mg with 500mg/5ml oral suspension |
| 47081 | Paracetamol 500mg with codeine phosphate 12.8mg & caffeine 30mg effervescent tablet |
| 47200 | Propain Plus caplets (Ceuta Healthcare Ltd) |
| 47508 | Codipar 15mg/500mg effervescent tablets (AMCo) |
| 47847 | Co-codamol 30mg/500mg capsules (Zentiva) |
| 48311 | Co-codamol 30mg/500mg caplets (Kent Pharmaceuticals Ltd) |
| 48775 | Co-codamol 30mg/500mg caplets (AMCo) |
| 51084 | Co-codamol 30mg/500mg capsules (AMCo) |
| 51381 | Generic Solpadeine Plus capsules |
| 51819 | Co-codamol 8mg/500mg capsules (Phoenix Healthcare Distribution Ltd) |
| 52085 | Co-codamol 30mg/500mg effervescent tablets (Zanza Laboratories Ltd) |
| 52966 | Generic Solpadeine Plus tablets |
| 53079 | Co-dydramol 10mg/500mg tablets (Sigma Pharmaceuticals Plc) |
| 53208 | Co-proxamol 32.5mg/325mg tablets (Lexon (UK) Ltd) |
| 53287 | Co-codamol 30mg/500mg effervescent tablets (Alliance Healthcare (Distribution) Ltd) |
| 53679 | Co-codamol 30mg/500mg effervescent tablets (A A H Pharmaceuticals Ltd) |
| 53702 | Co-codamol 30mg/500mg tablets (Actavis UK Ltd) |
| 54713 | Dypracet 20mg/500mg tablets (Auden McKenzie (Pharma Division) Ltd) |
| 55044 | Co-codamol 30mg/500mg caplets (Waymade Healthcare Plc) |
| 55245 | Co-proxamol 32.5mg/325mg tablets (Phoenix Healthcare Distribution Ltd) |
| 55465 | Co-codamol 30mg/500mg capsules (Sigma Pharmaceuticals Plc) |
| 55530 | Co-dydramol 10mg/500mg tablets (Phoenix Healthcare Distribution Ltd) |
| 56006 | Co-codamol 8mg/500mg tablets (Bristol Laboratories Ltd) |
| 56171 | Co-codamol 30mg/500mg capsules (Kent Pharmaceuticals Ltd) |
| 56205 | Boots Paracetamol and Codeine Extra capsules (The Boots Company Plc) |
| 56266 | Co-codamol 30mg/500mg tablets (Alliance Healthcare (Distribution) Ltd) |
| 56340 | Co-codamol 30mg/500mg caplets (J M McGill Ltd) |
| 56461 | Co-codamol 30mg/500mg tablets (Kent Pharmaceuticals Ltd) |
| 56549 | Co-codamol 8mg/500mg effervescent tablets (Waymade Healthcare Plc) |
| 56565 | Co-codamol 15mg/500mg tablets (A A H Pharmaceuticals Ltd) |
| 57097 | Co-codamol 30mg/500mg capsules (Alliance Healthcare (Distribution) Ltd) |
| 57197 | Co-dydramol 10mg/500mg tablets (Waymade Healthcare Plc) |
| 57353 | Co-codamol 30mg/500mg caplets (DE Pharmaceuticals) |
| 57465 | Co-codamol 8mg/500mg caplets (DE Pharmaceuticals) |
| 57839 | Co-codamol 15mg/500mg tablets (Waymade Healthcare Plc) |
| 57865 | Co-codamol 30mg/500mg caplets (Actavis UK Ltd) |
| 57900 | Co-codamol 30mg/500mg caplets (A A H Pharmaceuticals Ltd) |
| 57929 | Co-codamol 8mg/500mg capsules (Waymade Healthcare Plc) |
| 58288 | Co-codamol 30mg/500mg capsules (DE Pharmaceuticals) |
| 58501 | Co-codamol 30mg/500mg caplets (AM Distributions (Yorkshire) Ltd) |
| 58636 | Co-codamol 8mg/500mg effervescent tablets (Teva UK Ltd) |
| 58848 | Dypracet 30mg/500mg tablets (Auden McKenzie (Pharma Division) Ltd) |
| 58855 | Co-codamol 30mg/500mg capsules (Waymade Healthcare Plc) |
| 59131 | Co-codamol 8mg/500mg capsules (Bayer Plc) |
| 59442 | Co-codamol 30mg/500mg caplets (Phoenix Healthcare Distribution Ltd) |
| 59479 | Co-codamol 30mg/500mg capsules (Actavis UK Ltd) |
| 59705 | Co-codamol 8mg/500mg capsules (DE Pharmaceuticals) |
| 59986 | Co-codamol 30mg/500mg tablets (M & A Pharmachem Ltd) |
| 60040 | Generic Migraleve Pink tablets |
| 60517 | Co-codamol 30mg/500mg effervescent tablets (Almus Pharmaceuticals Ltd) |
| 61372 | Co-dydramol 10mg/500mg tablets (Kent Pharmaceuticals Ltd) |
| 61647 | Co-codamol 15mg/500mg tablets (Alliance Healthcare (Distribution) Ltd) |
| 61698 | Co-dydramol 10mg/500mg/5ml oral solution |
| 62169 | Co-codamol 8mg/500mg tablets (Vantage) |
| 62635 | Co-dydramol 10mg/500mg tablets (Alliance Healthcare (Distribution) Ltd) |
| 63551 | Co-codamol 8mg/500mg effervescent tablets (Vantage) |
| 63900 | Co-codamol 8mg/500mg tablets (DE Pharmaceuticals) |
| 64074 | Co-dydramol 10mg/500mg tablets (DE Pharmaceuticals) |
| 64368 | Paracetamol 500mg / Dihydrocodeine 30mg tablets (Icarus Pharmaceuticals Ltd) |
| 64387 | Co-codamol 30mg/500mg tablets (DE Pharmaceuticals) |
| 64545 | Co-codamol 8mg/500mg tablets (Aspar Pharmaceuticals Ltd) |
| 64726 | Co-codamol 15mg/500mg tablets (Galen Ltd) |
| 65035 | Co-dydramol 10mg/500mg tablets (Almus Pharmaceuticals Ltd) |
| 65092 | Co-codamol 30mg/500mg caplets (Mawdsley-Brooks & Company Ltd) |
| 65314 | Co-codamol 8mg/500mg caplets (Actavis UK Ltd) |
| 65440 | Co-codamol 30mg/500mg tablets (Mylan) |
| 65806 | Co-codamol 8mg/500mg tablets (Alliance Healthcare (Distribution) Ltd) |
| 65904 | Co-codamol 8mg/500mg capsules (Sigma Pharmaceuticals Plc) |
| 66352 | Co-codamol 8mg/500mg caplets (Kent Pharmaceuticals Ltd) |
| 66538 | Co-codamol 8mg/500mg capsules (Alliance Healthcare (Distribution) Ltd) |
| 66553 | Co-codamol 30mg/500mg effervescent tablets (AMCo) |
| 66602 | Generic Solpadeine Max soluble tablets sugar free |
| 66807 | Paracetamol with codeine phosphate peadiatric elixir |
| 66904 | Co-codamol 8mg/500mg caplets (Wockhardt UK Ltd) |
| 67106 | Co-codamol 30mg/500mg caplets (Wockhardt UK Ltd) |
| 67753 | Co-codamol 8mg/500mg tablets (Wockhardt UK Ltd) |
| 67755 | Co-proxamol 32.5mg/325mg tablets (Almus Pharmaceuticals Ltd) |
| 67779 | Co-dydramol 10mg/500mg tablets (Wockhardt UK Ltd) |
| 68252 | Co-codamol 30mg/500mg tablets (Bristol Laboratories Ltd) |
| 68562 | Generic Syndol caplets |
| 69006 | Co-dydramol 10mg/500mg/5ml oral suspension sugar free |
| 69043 | Co-proxamol 32.5mg/325mg tablets (DE Pharmaceuticals) |
| 69304 | Co-codamol 15mg/500mg tablets (Actavis UK Ltd) |
| 69534 | Co-proxamol 32.5mg/325mg tablets (Alliance Healthcare (Distribution) Ltd) |
| 70497 | Co-codamol 15mg/500mg capsules (A A H Pharmaceuticals Ltd) |
| 70518 | Co-codamol 60mg/1000mg tablets |
| 71006 | Co-dydramol 10mg/500mg oral powder sachets (Special Order) |
| 71227 | Syndol Headache Relief tablets (Sanofi) |
| 71523 | Co-dydramol 20mg/500mg tablets (M & A Pharmachem Ltd) |
| 71566 | Co-dydramol 30mg/500mg tablets (M & A Pharmachem Ltd) |
| 71614 | Co-codamol 15mg/500mg tablets (Zentiva) |
| 72110 | Co-dydramol 30mg/500mg tablets (A A H Pharmaceuticals Ltd) |
| 72111 | Co-dydramol 20mg/500mg tablets (A A H Pharmaceuticals Ltd) |
| 72258 | Co-dydramol 30mg/500mg tablets (Alliance Healthcare (Distribution) Ltd) |
| 72282 | Co-dydramol 20mg/500mg tablets (Alliance Healthcare (Distribution) Ltd) |
| 72389 | Co-codamol 15mg/500mg capsules (Alliance Healthcare (Distribution) Ltd) |
| **Non-opioids analgesics** | |
| 49 | Amitriptyline 25mg tablets |
| 83 | Amitriptyline 10mg tablets |
| 182 | Tryptizol 10mg/ml Injection (Merck Sharp & Dohme Ltd) |
| 487 | Amitriptyline 25mg modified-release capsules |
| 595 | Amitriptyline 25mg / Perphenazine 2mg tablets |
| 873 | AMITRIPTYLINE 100 MG TAB |
| 1208 | Triptafen tablets (AMCo) |
| 1453 | Triptafen m 2mg+10mg Tablet (Goldshield Pharmaceuticals Ltd) |
| 1888 | Amitriptyline 50mg tablets |
| 2486 | Lentizol 25mg modified-release capsules (Pfizer Ltd) |
| 2525 | Amitriptyline 75mg modified-release capsules |
| 2985 | Lentizol 50mg modified-release capsules (Pfizer Ltd) |
| 3490 | Amitriptyline 10mg / Perphenazine 2mg tablets |
| 3771 | AMITRIPTYLINE 75 MG TAB |
| 3777 | Amitriptyline 10mg/5ml sugar free oral solution |
| 4682 | Amitriptyline 50mg modified-release capsules |
| 4690 | Amitriptyline 50mg/5ml oral solution sugar free |
| 6312 | Amitriptyline 25mg/5ml oral solution sugar free |
| 6894 | Perphenazine 2mg with Amitriptyline 25mg tablet |
| 7751 | Tryptizol 25mg Tablet (Merck Sharp & Dohme Ltd) |
| 8250 | AMITRIPTYLINE S/F 25 MG/5ML SYR |
| 8332 | Tryptizol 50mg Tablet (Merck Sharp & Dohme Ltd) |
| 8726 | Tryptizol 10mg Tablet (Merck Sharp & Dohme Ltd) |
| 8831 | Tryptizol mr 75mg Modified-release capsule (Merck Sharp & Dohme Ltd) |
| 8878 | Tryptizol 10mg/5ml sugar free Oral solution (Merck Sharp and Dohme Ltd) |
| 11963 | Limbitrol 10 Capsule (Roche Products Ltd) |
| 13496 | AMITRIPTYLINE 200 MG TAB |
| 14534 | Limbitrol 5 Capsule (Roche Products Ltd) |
| 16323 | Perphenazine 2mg with Amitriptyline 10mg tablet |
| 18342 | Amitriptyline 25mg / Chlordiazepoxide 10mg capsules |
| 19779 | Amitriptyline 10mg/ml injection |
| 20026 | Domical 25mg Tablet (Berk Pharmaceuticals Ltd) |
| 20712 | AMITRIPTYLINE S/R |
| 21081 | Amitriptyline 12.5mg / Chlordiazepoxide 5mg capsules |
| 22070 | Amitriptyline 10mg/5ml Oral solution (Rosemont Pharmaceuticals Ltd) |
| 23497 | AMITRIPTYLINE 300 MG TAB |
| 24134 | Amitriptyline 25mg tablets (Kent Pharmaceuticals Ltd) |
| 24141 | Amitriptyline 10mg tablets (Actavis UK Ltd) |
| 24145 | Amitriptyline 25mg tablets (Actavis UK Ltd) |
| 24147 | Amitriptyline 25mg tablets (Teva UK Ltd) |
| 24152 | Amitriptyline 10mg tablets (Teva UK Ltd) |
| 24680 | Elavil 10mg Tablet (DDSA Pharmaceuticals Ltd) |
| 26213 | Domical 10mg Tablet (Berk Pharmaceuticals Ltd) |
| 27008 | Domical 50mg Tablet (Berk Pharmaceuticals Ltd) |
| 27876 | AMITRIPTYLINE |
| 30738 | AMITRIPTYLINE S/F |
| 32439 | Amitriptyline 25mg Tablet (Sussex Pharmaceutical Ltd) |
| 33090 | Amitriptyline 10mg tablets (A A H Pharmaceuticals Ltd) |
| 33624 | Amitriptyline 50mg tablets (Teva UK Ltd) |
| 34107 | Amitriptyline 50mg tablets (Wockhardt UK Ltd) |
| 34129 | Amitriptyline 25mg tablets (Wockhardt UK Ltd) |
| 34182 | Amitriptyline 50mg tablets (Kent Pharmaceuticals Ltd) |
| 34197 | Amitriptyline 25mg Tablet (Berk Pharmaceuticals Ltd) |
| 34224 | Amitriptyline 25mg/5ml oral solution sugar free (Rosemont Pharmaceuticals Ltd) |
| 34251 | Amitriptyline 50mg/5ml oral solution sugar free (Rosemont Pharmaceuticals Ltd) |
| 34274 | Amitriptyline 50mg tablets (A A H Pharmaceuticals Ltd) |
| 34401 | Amitriptyline 10mg tablets (Wockhardt UK Ltd) |
| 34474 | Amitriptyline 25mg Tablet (Regent Laboratories Ltd) |
| 34503 | Amitriptyline 25mg tablets (IVAX Pharmaceuticals UK Ltd) |
| 34634 | Amitriptyline 50mg tablets (Actavis UK Ltd) |
| 34731 | Amitriptyline 10mg tablets (Kent Pharmaceuticals Ltd) |
| 34782 | Amitriptyline 25mg tablets (A A H Pharmaceuticals Ltd) |
| 34916 | Amitriptyline 10mg Tablet (Berk Pharmaceuticals Ltd) |
| 38827 | Triptafen-M tablets (Mercury Pharma Group Ltd) |
| 40396 | Amitriptyline 50mg Tablet (Berk Pharmaceuticals Ltd) |
| 41729 | Amitriptyline 25mg Tablet (Celltech Pharma Europe Ltd) |
| 42078 | Amitriptyline 25mg tablets (Almus Pharmaceuticals Ltd) |
| 42394 | Amitriptyline 25mg Tablet (Crosspharma Ltd) |
| 45233 | Amitriptyline 10mg tablets (IVAX Pharmaceuticals UK Ltd) |
| 45242 | Amitriptyline 10mg Tablet (Sussex Pharmaceutical Ltd) |
| 46801 | Amitriptyline 10mg/5ml oral solution |
| 46818 | Amitriptyline 10mg/5ml oral suspension |
| 46970 | Amitriptyline 50mg tablets (IVAX Pharmaceuticals UK Ltd) |
| 48065 | Amitriptyline oral solution |
| 52867 | Amitriptyline 10mg tablets (Accord Healthcare Ltd) |
| 54877 | Amitriptyline 25mg tablets (Accord Healthcare Ltd) |
| 55139 | Amitriptyline 25mg tablets (Alliance Healthcare (Distribution) Ltd) |
| 55491 | Amitriptyline 10mg tablets (Almus Pharmaceuticals Ltd) |
| 57107 | Amitriptyline 10mg tablets (Phoenix Healthcare Distribution Ltd) |
| 57972 | Amitriptyline 10mg tablets (Alliance Healthcare (Distribution) Ltd) |
| 59161 | Amitriptyline 10mg tablets (Waymade Healthcare Plc) |
| 59820 | Amitriptyline 50mg/5ml oral solution sugar free (Wockhardt UK Ltd) |
| 60355 | Amitriptyline 25mg tablets (Phoenix Healthcare Distribution Ltd) |
| 60410 | Amitriptyline 25mg/5ml oral solution sugar free (Wockhardt UK Ltd) |
| 61835 | Amitriptyline 10mg tablets (DE Pharmaceuticals) |
| 64000 | Amitriptyline 10mg/5ml oral solution sugar free |
| 64141 | Amitriptyline 5mg/5ml oral solution |
| 64330 | Amitriptyline 50mg tablets (Almus Pharmaceuticals Ltd) |
| 64647 | Amitriptyline 25mg tablets (DE Pharmaceuticals) |
| 65439 | Amitriptyline 25mg tablets (Sandoz Ltd) |
| 65879 | Amitriptyline 10mg tablets (Sigma Pharmaceuticals Plc) |
| 65987 | Amitriptyline 25mg tablets (Crescent Pharma Ltd) |
| 66572 | Amitriptyline 25mg tablets (Sigma Pharmaceuticals Plc) |
| 66578 | Amitriptyline 10mg tablets (Mawdsley-Brooks & Company Ltd) |
| 66579 | Amitriptyline 25mg tablets (Mawdsley-Brooks & Company Ltd) |
| 67127 | Amitriptyline 25mg/5ml oral solution sugar free (DE Pharmaceuticals) |
| 69712 | Amitriptyline 50mg tablets (Sigma Pharmaceuticals Plc) |
| 70300 | Amitriptyline 10mg/5ml oral solution sugar free (Alliance Healthcare (Distribution) Ltd) |
| 70991 | Amitriptyline 10mg tablets (Arrow Generics Ltd) |
| 71042 | Amitriptyline 25mg tablets (Arrow Generics Ltd) |
| 3 | Aspirin 75mg dispersible tablets |
| 16 | Aspirin 75mg tablets |
| 34 | Aspirin 75mg gastro-resistant tablets |
| 111 | ASPIRIN 40 MG CAP |
| 216 | ASPIRIN 70 MG TAB |
| 254 | Aspirin 300mg tablets |
| 377 | Aspirin 300mg dispersible tablets |
| 381 | Anadin Tablet (Wyeth Consumer Healthcare) |
| 383 | ASPIRIN 60 MG TAB |
| 393 | Disprin 300mg dispersible tablets (Reckitt Benckiser Healthcare (UK) Ltd) |
| 395 | Aspirin mixture |
| 430 | Aspirin 500mg / Caffeine 32mg capsules |
| 434 | Aspirin 300mg gastro-resistant tablets |
| 645 | Aspirin 300mg suppositories |
| 657 | Aspirin 500mg granules sachets sugar free |
| 1049 | Nu-seals aspirin 600mg Tablet (Eli Lilly and Company Ltd) |
| 1137 | Nu-seals aspirin ec 300mg Gastro-resistant tablet (Eli Lilly and Company Ltd) |
| 1486 | ASPIRIN 75 MG SUP |
| 1902 | Aspirn 600mg gastro-resistant tablets |
| 2105 | Solprin 300mg Tablet (Reckitt Benckiser Healthcare (UK) Ltd) |
| 2607 | Paynocil Tablet (Beecham Research Laboratories) |
| 2628 | Nu-seals aspirin ec 75mg Gastro-resistant tablet (Eli Lilly and Company Ltd) |
| 2754 | ASPIRIN SOLUBLE 150 MG TAB |
| 2924 | ASPIRIN 150 MG TAB |
| 3155 | Migravess 5mg+325mg Effervescent tablet (Bayer Plc) |
| 3309 | Aspirin 325mg / Caffeine 15mg tablets |
| 3386 | Migravess forte 5mg+450mg Effervescent tablet (Bayer Plc) |
| 3726 | Metoclopramide with aspirin 5mg + 325mg Effervescent tablet |
| 4271 | ASPIRIN SOLUBLE 200 MG TAB |
| 4523 | ASPIRIN 50 MG CAP |
| 4679 | Asasantin Retard capsules (Boehringer Ingelheim Ltd) |
| 5288 | MigraMax oral powder sachets (Zentiva) |
| 6006 | Nu-Seals 75 gastro-resistant tablets (Alliance Pharmaceuticals Ltd) |
| 6007 | Nu-Seals 300 gastro-resistant tablets (Alliance Pharmaceuticals Ltd) |
| 6666 | Dipyridamole 200mg modified-release / Aspirin 25mg capsules |
| 6696 | Micropirin 75mg gastro-resistant tablets (Dexcel-Pharma Ltd) |
| 7417 | ASPIRIN 40 MG TAB |
| 7462 | ASPIRIN 325 MG CAP |
| 7486 | ASPIRIN 37.5 MG TAB |
| 7516 | Aspirin 300mg effervescent tablets sugar free |
| 7520 | Anadin Extra soluble tablets (Pfizer Consumer Healthcare Ltd) |
| 7539 | Beechams Powders oral powder sachets (GlaxoSmithKline Consumer Healthcare) |
| 7665 | ASPIRIN SR 300 MG TAB |
| 7769 | ASPIRIN/PARACETAMOL TAB |
| 7915 | ASPIRIN SR 100 MG TAB |
| 7944 | ASPIRIN SOLUBLE 40 MG CAP |
| 8185 | Disprin CV 300mg modified-release tablets (Reckitt Benckiser Healthcare (UK) Ltd) |
| 8186 | Aspirin 300mg modified-release tablets |
| 8381 | ASPIRIN /CAMPHOR /MENTHOL /METHYL SALICY 1.2 % LIN |
| 8424 | ASPIRIN PAED 81 MG TAB |
| 8645 | Aspirin 300mg effervescent tablets |
| 8733 | JUNIOR ASPIRIN 37.5 MG TAB |
| 8734 | ASPIRIN disp 37.5 MG TAB |
| 8843 | ASPIRIN 325 MG TAB |
| 8920 | ASPIRIN SOLUBLE 500 MG TAB |
| 9027 | ASPIRIN disp 150 MG TAB |
| 9144 | Caprin 75mg gastro-resistant tablets (Wockhardt UK Ltd) |
| 9301 | Aspirin 100mg modified-release tablets |
| 9939 | Aspirin 500mg effervescent tablets sugar free |
| 10031 | Aspirin 25mg with Dipyridamole 200mg modified-release capsules |
| 10305 | Aspirin 162.5mg capsules |
| 10310 | Aspirin powder |
| 11941 | ASPIRIN SACHETS 30 MG |
| 11951 | Original Phensic Aspirin tablets (Merck Consumer Health Products) |
| 11977 | Aspro clear maximum strength tablets |
| 12047 | Aspellin Ointment (Rpr / Fisons) |
| 12102 | ASPIRIN SOLUBLE 100 MG TAB |
| 12605 | ASPIRIN & CAFFEINE disp 300 MG TAB |
| 12964 | Aspirin 600mg / Caffeine 50mg oral powder sachets sugar free |
| 12976 | Aspirin 900mg / Metoclopramide 10mg oral powder sachets sugar free |
| 13882 | Imazin XL tablets (Napp Pharmaceuticals Ltd) |
| 14517 | Robaxisal forte Tablet (Shire Pharmaceuticals Ltd) |
| 15044 | ASPIRIN disp 500 MG TAB |
| 15352 | ASPIRIN & PARACETAMOL TAB |
| 15364 | Aspirin 150mg suppositories |
| 15367 | Anadin Extra tablets (Pfizer Consumer Healthcare Ltd) |
| 15397 | ASPIRIN SOLUBLE 50 MG TAB |
| 15447 | ASPIRIN SOLUBLE 600 MG TAB |
| 15517 | ASPIRIN 100 MG SUP |
| 16184 | Alka-seltzer Tablet (Bayer Plc) |
| 16611 | Anadin Tablet (Wyeth Consumer Healthcare) |
| 17180 | Metoclopramide with aspirin 5mg + 450mg Effervescent tablet |
| 17456 | Metoclopramide with lysine acetylsalicylate 10mg + 900mg Oral solution |
| 17704 | Platet 100mg Effervescent tablet (Roche Products Ltd) |
| 17920 | Disprin cv 100mg Modified-release tablet (Reckitt Benckiser Healthcare (UK) Ltd) |
| 18030 | Imazin XL forte tablets (Napp Pharmaceuticals Ltd) |
| 18217 | Aspirin 300mg orodispersible tablets sugar free |
| 18329 | Enprin 75mg gastro-resistant tablets (Galpharm International Ltd) |
| 19189 | Micropirin 75mg Gastro-resistant tablet (Ratiopharm UK Ltd) |
| 19255 | Paracetamol 200mg with aspirin 300mg & caffeine 45mg tablet |
| 19577 | NU-SEALS ASPIRIN |
| 19674 | ASPIRIN DISPERSIBLE |
| 19797 | NU-SEALS ASPIRIN |
| 19813 | ASPIRIN SOLUBLE |
| 20206 | ASPIRIN 50 MG SUP |
| 20650 | Aspirin 300mg / Paracetamol 200mg dispersible tablets sugar free |
| 20840 | Acetylsalicylic acid mix |
| 21380 | Aspirin 75mg / Isosorbide mononitrate 60mg modified-release tablets |
| 21382 | Aspirin 150mg / Isosorbide mononitrate 60mg modified-release tablets |
| 21770 | Paracetamol 200mg with aspirin 300mg dispersible tablet |
| 21921 | Postmi ec 300mg Gastro-resistant tablet (Ashbourne Pharmaceuticals Ltd) |
| 22107 | ASPIRIN disp 200 MG TAB |
| 22138 | Aspirin 324mg modified-release tablets |
| 22232 | Disprin Direct 300mg orodispersible tablets (Reckitt Benckiser Healthcare (UK) Ltd) |
| 22253 | ASPIRIN paed 150 MG SUP |
| 22305 | Disprin Extra dispersible tablets (Reckitt Benckiser Healthcare (UK) Ltd) |
| 22618 | Solprin 75mg Tablet (Reckitt Benckiser Healthcare (UK) Ltd) |
| 22776 | Aspirin 500mg with Cyclizine 25mg effervescent tablets |
| 22824 | ASPIRIN disp 600 MG TAB |
| 22863 | ASPIRIN S/R 500 MG TAB |
| 22864 | ASPIRIN PAED MIX |
| 23488 | Claradin 300mg Tablet (Nicholas Laboratories Ltd) |
| 23491 | ASPIRIN 500 MG SUP |
| 23495 | ASPIRIN |
| 23593 | PostMI 75 dispersible tablets (Ashbourne Pharmaceuticals Ltd) |
| 23841 | Safapryn Tablet (Pfizer Ltd) |
| 23878 | Nu-seals cardio ec 75mg Gastro-resistant tablet (Genus Pharmaceuticals Ltd) |
| 23932 | Aspro Clear 300mg effervescent tablets (Bayer Plc) |
| 24025 | Caprin 300mg gastro-resistant tablets (Pinewood Healthcare) |
| 24309 | Paracetamol 120mg with aspirin 165.3mg & caffeine 60mg powder |
| 24427 | ANHYDROUS CITRIC ACID /ASPIRIN /CALCIUM 8 MG TAB |
| 24622 | Aspirin 325mg / Caffeine 22mg tablets |
| 24857 | ASPIRIN 250 MG SUP |
| 24960 | Aspirin 300mg tablets (Vantage) |
| 25211 | Anadin Original tablets (Pfizer Consumer Healthcare Ltd) |
| 25335 | PostMI 75 EC tablets (Ashbourne Pharmaceuticals Ltd) |
| 25433 | Radian B Muscle lotion (Thornton & Ross Ltd) |
| 25718 | Angettes 75 tablets (Bristol-Myers Squibb Pharmaceuticals Ltd) |
| 26099 | ASPIRIN 175 MG SUP |
| 26424 | ASPIRIN 200 MG SUP |
| 26582 | ASPIRIN PAED 100 MG SUP |
| 26792 | ASPIRIN 125 MG SUP |
| 26967 | Alka-Seltzer XS effervescent tablets (Bayer Plc) |
| 27435 | Paracetamol 200mg with aspirin 300mg & caffeine 45mg soluble tablet |
| 27467 | ASPIRIN SOLUBLE 400 MG TAB |
| 28238 | ASPIRIN 300MG/LYSINE 245MG 300 MG TAB |
| 28707 | ASPIRIN M/F 324 MG TAB |
| 28810 | Aspirin 300mg with Glycine 133mg soluble tablets |
| 29054 | Methocarbamol with aspirin Tablet |
| 29759 | Aspro Tablet (Roche Consumer Health) |
| 29848 | Aspirin 300mg with Glycine 150mg chewable tablets |
| 30022 | Fynnon calcium aspirin 500mg Soluble tablet (SSL International Plc) |
| 30432 | ASPIRIN & DOVER'S pwdr TAB |
| 30695 | ASPIRIN 120 MG SUP |
| 30920 | Aspirin 300mg Dispersible tablet (M & A Pharmachem Ltd) |
| 31001 | Cullens headache powders Sachets (Cullen and Davidson) |
| 31210 | Aspirin 300mg Tablet (Co-operative) |
| 31211 | Aspirin 75mg Dispersible tablet (A A H Pharmaceuticals Ltd) |
| 31499 | Paracetamol with aspirin tablet |
| 31858 | Caspac xl 162.5mg Capsule (Pharmacia Ltd) |
| 31870 | Aspirin 320mg tablets |
| 31938 | Aspirin 75mg gastro-resistant tablets (Sandoz Ltd) |
| 31953 | Aspirin 75mg dispersible tablets (IVAX Pharmaceuticals UK Ltd) |
| 31954 | Aspirin 75mg dispersible tablets (Teva UK Ltd) |
| 31956 | Aspirin 75mg gastro-resistant tablets (Kent Pharmaceuticals Ltd) |
| 32036 | Aspirin 75mg dispersible tablets (Actavis UK Ltd) |
| 32154 | Anadin Soluble tablet (Wyeth Consumer Healthcare) |
| 32178 | Paracetamol 133mg with aspirin 267mg & caffeine 40mg effervescent tablet |
| 32210 | Aspirin 300mg dispersible tablets (Actavis UK Ltd) |
| 32314 | Aspirin with aloxiprin and caffeine powder |
| 32728 | Askit oral powder sachets (Bayer Plc) |
| 32992 | Aspirin 75mg gastro-resistant tablets (Mylan) |
| 33075 | ASPIRIN 600MG/GLYCINE 300MG 600 MG TAB |
| 33139 | Anadin maximum strength Capsule (Wyeth Consumer Healthcare) |
| 33293 | Aspirin 75mg gastro-resistant tablets (Sterwin Medicines) |
| 33317 | Aspirin with Sodium bicarbonate with Citric acid effervescent tablets |
| 33320 | Aspirin 75mg Dispersible tablet (Sovereign Medical Ltd) |
| 33656 | Aspirin 75mg dispersible tablets (A A H Pharmaceuticals Ltd) |
| 33662 | Aspirin 300mg Dispersible tablet (A A H Pharmaceuticals Ltd) |
| 33668 | Aspirin 300mg Dispersible tablet (Rusco Ltd) |
| 33676 | Aspirin 75mg dispersible tablets (Kent Pharmaceuticals Ltd) |
| 34233 | Aspirin with aloxiprin and caffeine capsules |
| 34309 | Aspirin 300mg dispersible tablets (A A H Pharmaceuticals Ltd) |
| 34385 | Aspirin 75mg Soluble tablet (Co-operative) |
| 34386 | Aspirin 300mg tablets (Actavis UK Ltd) |
| 34434 | Aspirin 75mg dispersible tablets (Thornton & Ross Ltd) |
| 34485 | Aspirin 75mg gastro-resistant tablets (IVAX Pharmaceuticals UK Ltd) |
| 34611 | Aspirin 75mg gastro-resistant tablets (C P Pharmaceuticals Ltd) |
| 34666 | Aspirin ec 300mg Gastro-resistant tablet (A A H Pharmaceuticals Ltd) |
| 34762 | Aspirin 300mg Gastro-resistant tablet (Galen Ltd) |
| 34796 | Aspirin 75mg Gastro-resistant tablet (Galen Ltd) |
| 34797 | Aspirin 75mg gastro-resistant tablets (Actavis UK Ltd) |
| 34942 | Aspirin 75mg Dispersible tablet (Nucare Plc) |
| 35967 | Paramed Extra Power Pain Control tablets (Galpharm International Ltd) |
| 36521 | Aspirin 500mg modified-release tablets |
| 36543 | Aspirin 100mg effervescent tablets |
| 37541 | Aspirin 227mg medicated chewing-gum |
| 39738 | Aspirin 162.5mg modified-release capsules |
| 40144 | Aspirin 300mg Dispersible tablet (Thornton & Ross Ltd) |
| 40191 | ASPIRIN /CAFFEINE /QUININE SULPHATE 325 MG TAB |
| 40381 | Aspirin 75mg Soluble tablet (C P Pharmaceuticals Ltd) |
| 41512 | Aspirin 75mg gastro-resistant tablets (Teva UK Ltd) |
| 41569 | Aspirin 300mg tablets (A A H Pharmaceuticals Ltd) |
| 41594 | Aspirin 300mg Dispersible tablet (Teva UK Ltd) |
| 41766 | Maximum Strength Aspro Clear 500mg effervescent tablets (Bayer Plc) |
| 42061 | ASPIRIN 65 MG SUP |
| 43060 | Aspirin 300mg Soluble tablet (Celltech Pharma Europe Ltd) |
| 43434 | Aspirin 300mg gastro-resistant tablets (A A H Pharmaceuticals Ltd) |
| 43679 | Flamasacard 162.5mg Modified-release capsule (Abbey Pharmaceuticals Ltd) |
| 43709 | Aspirin 75mg gastro-resistant tablets (Almus Pharmaceuticals Ltd) |
| 43806 | Aspirin 300mg gastro-resistant tablets (Sandoz Ltd) |
| 44639 | Aspirin 300mg Dispersible tablet (Nucare Plc) |
| 45643 | Aspirin 75mg Soluble tablet (Celltech Pharma Europe Ltd) |
| 45840 | Aspirin 300mg Dispersible tablet (Numark Management Ltd) |
| 45851 | Aspirin 300mg Soluble tablet (Ranbaxy (UK) Ltd) |
| 47937 | Aspirin 75mg dispersible tablets (Wockhardt UK Ltd) |
| 47992 | Aspirin 75mg gastro-resistant tablets (A A H Pharmaceuticals Ltd) |
| 48000 | Aspirin 300mg tablets (Sigma Pharmaceuticals Plc) |
| 48021 | Aspirin 75mg Tablet (Hillcross Pharmaceuticals Ltd) |
| 48165 | Aspirin 300mg tablets (Aspar Pharmaceuticals Ltd) |
| 48974 | Aspirin 75mg tablets (Phoenix Healthcare Distribution Ltd) |
| 49060 | Aspirin 75mg dispersible tablets (Alliance Healthcare (Distribution) Ltd) |
| 49220 | Aspirin 300mg tablets (Kent Pharmaceuticals Ltd) |
| 49685 | Aspirin 75mg dispersible tablets (Sigma Pharmaceuticals Plc) |
| 49799 | Aspirin 150mg suppositories (A A H Pharmaceuticals Ltd) |
| 50166 | Generic Anadin Extra tablets |
| 50555 | Aspirin 300mg dispersible tablets (DE Pharmaceuticals) |
| 50926 | Aspirin 75mg dispersible tablets (The Boots Company Plc) |
| 50949 | Aspirin 75mg tablets (A A H Pharmaceuticals Ltd) |
| 51474 | Aspirin 150mg suppositories (Martindale Pharmaceuticals Ltd) |
| 51561 | Aspirin 75mg gastro-resistant tablets (Zanza Laboratories Ltd) |
| 52044 | Aspirin 300mg caplets (The Boots Company Plc) |
| 52280 | Aspirin 300mg Tablet (Wockhardt UK Ltd) |
| 52618 | Aspirin 75mg dispersible tablets (Bristol Laboratories Ltd) |
| 52905 | Aspirin 300mg tablets (Lloyds Pharmacy Ltd) |
| 53178 | Aspirin 75mg gastro-resistant tablets (Wockhardt UK Ltd) |
| 53325 | Salicylates with menthol and camphor liniment |
| 53622 | Aspirin 300mg Tablet (M & A Pharmachem Ltd) |
| 53711 | Aspirin 300mg Tablet (Nucare Plc) |
| 53791 | Aspirin 150mg suppositories (Alliance Healthcare (Distribution) Ltd) |
| 53804 | Aspirin 300mg gastro-resistant tablets (Alliance Healthcare (Distribution) Ltd) |
| 53816 | Aspirin 300mg dispersible tablets (Alliance Healthcare (Distribution) Ltd) |
| 54284 | Aspirin 75mg dispersible tablets (Almus Pharmaceuticals Ltd) |
| 54353 | Generic Anadin Extra soluble tablets sugar free |
| 54430 | Aspirin 75mg tablets (Alliance Healthcare (Distribution) Ltd) |
| 54526 | Aspirin 300mg tablets (Alliance Healthcare (Distribution) Ltd) |
| 54565 | Aspirin 75mg dispersible tablets (Lloyds Pharmacy Ltd) |
| 54734 | Aspirin 300mg tablets (Wockhardt UK Ltd) |
| 54997 | Aspirin 75mg dispersible tablets (Dowelhurst Ltd) |
| 55230 | Aspirin 300mg dispersible tablets (Kent Pharmaceuticals Ltd) |
| 55579 | Aspirin 300mg tablets (Almus Pharmaceuticals Ltd) |
| 56007 | Aspirin 300mg dispersible tablets (Sigma Pharmaceuticals Plc) |
| 56503 | Asasantin Retard capsules (Mawdsley-Brooks & Company Ltd) |
| 56736 | Aspirin 300mg tablets (Waymade Healthcare Plc) |
| 56883 | Aspirin 75mg tablets (Waymade Healthcare Plc) |
| 56995 | Aspirin 75mg dispersible tablets (Phoenix Healthcare Distribution Ltd) |
| 56996 | Aspirin 75mg dispersible tablets (Waymade Healthcare Plc) |
| 57057 | Aspirin 75mg dispersible tablets (Wockhardt UK Ltd) |
| 57585 | Asasantin Retard capsules (Dowelhurst Ltd) |
| 58112 | Alka-Seltzer effervescent tablets original (Bayer Plc) |
| 58331 | Aspirin 300mg gastro-resistant tablets (Mylan) |
| 59021 | Aspirin 75mg gastro-resistant tablets (Bristol Laboratories Ltd) |
| 59244 | Aspirin 100mg capsules |
| 59253 | Aspirin 75mg gastro-resistant tablets (Waymade Healthcare Plc) |
| 59728 | Aspirin 75mg tablets (Alissa Healthcare Research Ltd) |
| 59791 | Aspirin 75mg dispersible tablets (Aspar Pharmaceuticals Ltd) |
| 60127 | Aspirin 75mg tablets (DE Pharmaceuticals) |
| 60278 | Aspirin 300mg tablets (DE Pharmaceuticals) |
| 60693 | Aspirin 15mg/5ml oral solution |
| 60694 | Aspirin 25mg/5ml oral solution |
| 60777 | Aspirin 75mg gastro-resistant tablets (DE Pharmaceuticals) |
| 62334 | Aspirin 300mg caplets (Wockhardt UK Ltd) |
| 62430 | Aspirin 300mg suppositories (A A H Pharmaceuticals Ltd) |
| 63603 | Laboprin Tablet (Laboratories For Applied Biology Ltd) |
| 64071 | Aspirin powder (J M Loveridge Ltd) |
| 65027 | Bisoprolol 5mg / Aspirin 100mg capsules |
| 66171 | Aspirin 150mg Suppository (Distriphar (UK)) |
| 66345 | Aspirin 75mg dispersible tablets (DE Pharmaceuticals) |
| 66546 | Aspirin 75mg dispersible tablets (Numark Ltd) |
| 66563 | Aspirin 75mg gastro-resistant tablets (Phoenix Healthcare Distribution Ltd) |
| 66861 | Aspirin 75mg effervescent tablets |
| 67124 | Bisoprolol 10mg / Aspirin 75mg capsules |
| 67160 | Aspirin 300mg dispersible tablets (Lloyds Pharmacy Ltd) |
| 67362 | Aspirin 300mg suppositories (Alliance Healthcare (Distribution) Ltd) |
| 67521 | Aspirin 15mg/5ml oral suspension |
| 67754 | Aspirin 300mg dispersible tablets (Almus Pharmaceuticals Ltd) |
| 67858 | Aspirin 25mg capsules |
| 68051 | Aspirin 150mg suppositories (Colorama Pharmaceuticals Ltd) |
| 68752 | Aspirin 75mg tablets (Sigma Pharmaceuticals Plc) |
| 68764 | Molita 200mg/25mg modified-release capsules (Dr Reddy's Laboratories (UK) Ltd) |
| 69216 | Dipyridamole 200mg modified-release / Aspirin 25mg capsules (A A H Pharmaceuticals Ltd) |
| 70549 | Danamep 75mg dispersible tablets (Ecogen Europe Ltd) |
| 70841 | Aspirin 300mg Dispersible tablet (Family Health) |
| 71078 | Aspirin 300mg dispersible tablets (Mawdsley-Brooks & Company Ltd) |
| 71192 | Aspirin 75mg tablets (Kent Pharmaceuticals Ltd) |
| 71663 | Aspirin 75mg tablets (Actavis UK Ltd) |
| 71676 | Aspirin 75mg dispersible tablets (Mawdsley-Brooks & Company Ltd) |
| 71821 | Aspirin 300mg Tablet (Numark Management Ltd) |
| 432 | Carbamazepine 100mg tablets |
| 596 | Carbamazepine 100mg/5ml oral suspension sugar free |
| 652 | Carbamazepine 400mg tablets |
| 1158 | Carbamazepine 200mg tablets |
| 2085 | Tegretol 200mg tablets (Novartis Pharmaceuticals UK Ltd) |
| 2388 | Carbamazepine 200mg modified-release tablets |
| 2823 | Tegretol retard 200mg Modified-release tablet (Novartis Pharmaceuticals UK Ltd) |
| 2824 | Tegretol retard 400mg Modified-release tablet (Novartis Pharmaceuticals UK Ltd) |
| 3569 | Carbamazepine 400mg modified-release tablets |
| 4066 | Tegretol 400mg tablets (Novartis Pharmaceuticals UK Ltd) |
| 4913 | Tegretol 100mg/5ml liquid (Novartis Pharmaceuticals UK Ltd) |
| 4982 | Carbamazepine 100mg chewable tablets sugar free |
| 5977 | Tegretol 100mg tablets (Novartis Pharmaceuticals UK Ltd) |
| 11696 | Carbamazepine 200mg chewable tablets sugar free |
| 12880 | Tegretol 200mg Chewtabs (Novartis Pharmaceuticals UK Ltd) |
| 12931 | Epimaz Retard 200mg tablets (IVAX Pharmaceuticals UK Ltd) |
| 12932 | Epimaz 100mg tablets (IVAX Pharmaceuticals UK Ltd) |
| 13112 | Carbagen SR 200mg tablets (Mylan) |
| 13524 | Tegretol 100mg Chewtabs (Novartis Pharmaceuticals UK Ltd) |
| 15082 | Tegretol 250mg suppositories (Essential Pharma Ltd) |
| 17428 | Carbamazepine 250mg suppositories |
| 17429 | Carbamazepine 125mg suppositories |
| 17978 | Teril Retard 200mg tablets (Taro Pharmaceuticals (UK) Ltd) |
| 18011 | Teril Retard 400mg tablets (Taro Pharmaceuticals (UK) Ltd) |
| 18762 | Epimaz 200mg tablets (Teva UK Ltd) |
| 18763 | Epimaz 400mg tablets (Teva UK Ltd) |
| 21441 | Tegretol 125mg suppositories (Essential Pharma Ltd) |
| 26012 | Carbagen SR 400mg tablets (Mylan) |
| 28345 | Carbamazepine 100mg tablets (Teva UK Ltd) |
| 30509 | Timonil retard 400mg Modified-release tablet (C P Pharmaceuticals Ltd) |
| 30763 | Carbamazepine 100mg tablets (A A H Pharmaceuticals Ltd) |
| 32900 | Carbamazepine sr 200mg Tablet (IVAX Pharmaceuticals UK Ltd) |
| 32931 | Carbamazepine 400mg tablets (Teva UK Ltd) |
| 34370 | Carbamazepine 200mg modified-release tablets (A A H Pharmaceuticals Ltd) |
| 34904 | Carbamazepine 200mg tablets (A A H Pharmaceuticals Ltd) |
| 34958 | Carbamazepine 100mg Tablet (Berk Pharmaceuticals Ltd) |
| 37058 | Carbamazepine 500mg/5ml oral suspension |
| 37800 | Carbamazepine 200mg Modified-release tablet (Lagap) |
| 40403 | Carbamazepine 400mg Modified-release tablet (Lagap) |
| 40404 | Carbamazepine 400mg modified-release tablets (A A H Pharmaceuticals Ltd) |
| 41726 | Carbamazepine 100mg Tablet (IVAX Pharmaceuticals UK Ltd) |
| 43417 | Carbamazepine 400mg tablets (A A H Pharmaceuticals Ltd) |
| 43451 | Carbamazepine 400mg Modified-release tablet (Generics (UK) Ltd) |
| 45903 | Tegretol Prolonged Release 400mg tablets (Novartis Pharmaceuticals UK Ltd) |
| 45941 | Tegretol Prolonged Release 200mg tablets (Novartis Pharmaceuticals UK Ltd) |
| 46888 | Carbamazepine 200mg Modified-release tablet (Generics (UK) Ltd) |
| 46972 | Carbamazepine 200mg Tablet (IVAX Pharmaceuticals UK Ltd) |
| 47294 | Carbamazepine sr 400mg Tablet (IVAX Pharmaceuticals UK Ltd) |
| 48397 | Tegretol Prolonged Release 200mg tablets (DE Pharmaceuticals) |
| 49833 | Tegretol 200mg tablets (Lexon (UK) Ltd) |
| 51760 | Tegretol 400mg tablets (Lexon (UK) Ltd) |
| 52840 | Tegretol Prolonged Release 400mg tablets (Necessity Supplies Ltd) |
| 53188 | Carbamazepine 500mg/5ml Oral suspension (Martindale Pharmaceuticals Ltd) |
| 53492 | Tegretol Prolonged Release 200mg tablets (Sigma Pharmaceuticals Plc) |
| 53893 | Tegretol Prolonged Release 400mg tablets (Stephar (U.K.) Ltd) |
| 54445 | Carbamazepine 400mg tablets (Sigma Pharmaceuticals Plc) |
| 55904 | Carbamazepine 100mg tablets (Mylan) |
| 56734 | Carbamazepine 100mg tablets (Phoenix Healthcare Distribution Ltd) |
| 57726 | Tegretol Prolonged Release 200mg tablets (Mawdsley-Brooks & Company Ltd) |
| 57898 | Carbamazepine 100mg/5ml oral suspension |
| 58515 | Tegretol 100mg/5ml liquid (Lexon (UK) Ltd) |
| 59038 | Carbamazepine 100mg/5ml oral suspension sugar free (A A H Pharmaceuticals Ltd) |
| 59211 | Carbamazepine 200mg tablets (Alliance Healthcare (Distribution) Ltd) |
| 59428 | Carbamazepine 100mg tablets (Almus Pharmaceuticals Ltd) |
| 59429 | Carbamazepine 200mg tablets (Almus Pharmaceuticals Ltd) |
| 59430 | Carbamazepine 400mg tablets (Almus Pharmaceuticals Ltd) |
| 59696 | Carbamazepine 400mg tablets (Kent Pharmaceuticals Ltd) |
| 59697 | Carbamazepine 10mg/5ml oral suspension |
| 59745 | Carbamazepine 100mg/5ml oral suspension sugar free (DE Pharmaceuticals) |
| 59746 | Carbamazepine 100mg tablets (DE Pharmaceuticals) |
| 59887 | Carbamazepine 200mg tablets (Mylan) |
| 60133 | Tegretol Prolonged Release 400mg tablets (Sigma Pharmaceuticals Plc) |
| 60181 | Carbagen 100mg tablets (Mylan) |
| 60187 | Carbagen 200mg tablets (Mylan) |
| 60405 | Carbagen 400mg tablets (Mylan) |
| 61143 | Carbamazepine 100mg tablets (Actavis UK Ltd) |
| 61377 | Carbamazepine 100mg tablets (Kent Pharmaceuticals Ltd) |
| 62457 | Carbamazepine 100mg tablets (Alliance Healthcare (Distribution) Ltd) |
| 62538 | Carbamazepine 200mg tablets (Actavis UK Ltd) |
| 63213 | Tegretol 200mg tablets (Necessity Supplies Ltd) |
| 63341 | Tegretol Prolonged Release 200mg tablets (Lexon (UK) Ltd) |
| 63735 | Carbamazepine 400mg tablets (Alliance Healthcare (Distribution) Ltd) |
| 63930 | Tegretol 100mg/5ml liquid (DE Pharmaceuticals) |
| 66058 | Carbamazepine 200mg tablets (DE Pharmaceuticals) |
| 66073 | Carbamazepine 400mg tablets (DE Pharmaceuticals) |
| 66375 | Carbamazepine 400mg modified-release tablets (Phoenix Healthcare Distribution Ltd) |
| 69155 | Carbamazepine 40mg/5ml oral suspension |
| 70773 | Carbamazepine 200mg modified-release tablets (DE Pharmaceuticals) |
| 70901 | Carbamazepine 100mg tablets (Waymade Healthcare Plc) |
| 70902 | Carbamazepine 200mg modified-release tablets (Phoenix Healthcare Distribution Ltd) |
| 71149 | Tegretol Prolonged Release 400mg tablets (Waymade Healthcare Plc) |
| 71350 | Tegretol Prolonged Release 200mg tablets (Necessity Supplies Ltd) |
| 71365 | Tegretol Prolonged Release 400mg tablets (DE Pharmaceuticals) |
| 71432 | Tegretol Prolonged Release 200mg tablets (Waymade Healthcare Plc) |
| 72018 | Carbamazepine 100mg/5ml oral suspension sugar free (Actavis UK Ltd) |
| 474 | Celecoxib 100mg capsules |
| 5080 | Celebrex 200mg capsules (Pfizer Ltd) |
| 5175 | Celebrex 100mg capsules (Pfizer Ltd) |
| 5254 | Celecoxib 200mg capsules |
| 43616 | Celecoxib 400mg capsules |
| 50059 | Celebrex 100mg capsules (Necessity Supplies Ltd) |
| 52420 | Celebrex 100mg capsules (Mawdsley-Brooks & Company Ltd) |
| 55582 | Celebrex 200mg capsules (Lexon (UK) Ltd) |
| 62840 | Celebrex 100mg capsules (DE Pharmaceuticals) |
| 64245 | Celecoxib 200mg capsules (A A H Pharmaceuticals Ltd) |
| 64935 | Celecoxib 200mg capsules (Sigma Pharmaceuticals Plc) |
| 65016 | Celecoxib 200mg capsules (Alliance Healthcare (Distribution) Ltd) |
| 66571 | Celecoxib 200mg capsules (Actavis UK Ltd) |
| 66757 | Celebrex 200mg capsules (Waymade Healthcare Plc) |
| 72293 | Celecoxib 200mg capsules (Mylan) |
| 10325 | Dexibuprofen 300mg tablets |
| 11907 | Dexibuprofen 400mg tablets |
| 21419 | Seractil 300mg tablets (Thornton & Ross Ltd) |
| 21421 | Seractil 400mg tablets (Thornton & Ross Ltd) |
| 40 | Diclofenac sodium 50mg gastro-resistant tablets |
| 156 | Diclofenac 1.16% gel |
| 157 | Voltarol 100mg Suppository (Novartis Pharmaceuticals UK Ltd) |
| 162 | Arthrotec 50 gastro-resistant tablets (Pfizer Ltd) |
| 417 | Diclofenac 50mg dispersible tablets sugar free |
| 447 | Diclofenac sodium 75mg modified-release capsules |
| 497 | Voltarol 25mg gastro-resistant tablets (Novartis Pharmaceuticals UK Ltd) |
| 499 | Diclofenac 50mg suppositories |
| 580 | Diclofenac sodium 75mg modified-release tablets |
| 589 | Voltarol 50mg dispersible tablets (Novartis Pharmaceuticals UK Ltd) |
| 597 | Diclofenac potassium 50mg tablets |
| 612 | Dicloflex 25mg gastro-resistant tablets (Dexcel-Pharma Ltd) |
| 628 | Diclofenac potassium 25mg tablets |
| 649 | Diclofenac sodium 25mg gastro-resistant tablets |
| 676 | Diclofenac 75mg/3ml solution for injection ampoules |
| 827 | Voltarol 1.16% Emulgel (GlaxoSmithKline Consumer Healthcare) |
| 917 | Diclofenac sodium 50mg tablets |
| 928 | Diclofenac sodium 25mg tablets |
| 1075 | Diclofenac sodium 50mg gastro-resistant tablets |
| 1096 | Diclofenac sodium 25mg gastro-resistant tablets |
| 1115 | Diclofenac sodium 100mg modified-release capsules |
| 1116 | Diclofenac 100mg suppositories |
| 1139 | Voltarol 25mg Tablet (Novartis Pharmaceuticals UK Ltd) |
| 1233 | Diclofenac sodium 75mg modified-release tablets |
| 1446 | Voltarol 50mg Tablet (Novartis Pharmaceuticals UK Ltd) |
| 1692 | Diclofenac sodium 50mg gastro-resistant / Misoprostol 200microgram tablets |
| 1766 | Voltarol sr 75mg Modified-release tablet (Novartis Pharmaceuticals UK Ltd) |
| 1984 | Diclofenac sodium 100mg modified-release tablets |
| 2293 | Voltarol 25mg/ml Injection (Novartis Pharmaceuticals UK Ltd) |
| 2386 | Voltarol Retard 100mg tablets (Novartis Pharmaceuticals UK Ltd) |
| 2387 | Arthrotec 75 gastro-resistant tablets (Pfizer Ltd) |
| 2904 | Diclofenac sodium 75mg gastro-resistant modified-release capsules |
| 3416 | Diclofenac sodium 100mg modified-release tablets |
| 3421 | Diclomax sr 75mg Modified-release capsule (Provalis Healthcare Ltd) |
| 3852 | Diclomax 100mg Modified-release capsule (Provalis Healthcare Ltd) |
| 3958 | Diclofenac 25mg suppositories |
| 4095 | Voltarol 12.5mg Suppository (Novartis Pharmaceuticals UK Ltd) |
| 4506 | Volsaid Retard 75 tablets (Chiesi Ltd) |
| 4625 | Voltarol 75mg SR tablets (Novartis Pharmaceuticals UK Ltd) |
| 4631 | Voltarol 50mg gastro-resistant tablets (Novartis Pharmaceuticals UK Ltd) |
| 4692 | Dicloflex 50mg gastro-resistant tablets (Dexcel-Pharma Ltd) |
| 4713 | Voltarol 75mg/3ml solution for injection ampoules (Novartis Pharmaceuticals UK Ltd) |
| 4806 | Voltarol 100mg suppositories (Novartis Pharmaceuticals UK Ltd) |
| 4880 | Diclofenac sodium 75mg gastro-resistant / Misoprostol 200microgram tablets |
| 5085 | Voltarol Rapid 50mg tablets (Novartis Pharmaceuticals UK Ltd) |
| 5200 | Voltarol 50mg suppositories (Novartis Pharmaceuticals UK Ltd) |
| 5401 | Voltarol Rapid 25mg tablets (Novartis Pharmaceuticals UK Ltd) |
| 6115 | Diclofenac sodium 3% gel |
| 6208 | Voltarol 1.16% Emulgel P (GlaxoSmithKline Consumer Healthcare) |
| 6435 | Pennsaid 16mg/ml cutaneous solution (Movianto UK Ltd) |
| 6881 | Solaraze 3% gel (Almirall Ltd) |
| 7458 | DICLOFENAC SODIUM (3ML) 25 MG/ML INJ |
| 7667 | Diclofenac 12.5mg suppositories |
| 7774 | DICLOFENAC 75 MG INJ |
| 8062 | Motifene 75mg modified-release capsules (Daiichi Sankyo UK Ltd) |
| 8789 | Dicloflex retard tabs 100 100mg Modified-release tablet (Dexcel-Pharma Ltd) |
| 9222 | Dicloflex 75mg SR tablets (Dexcel-Pharma Ltd) |
| 9465 | Diclotard 100 100mg Modified-release tablet (Galen Ltd) |
| 9500 | Diclotard 75mg modified-release tablets (Galen Ltd) |
| 9688 | Diclovol 75mg SR tablets (Mylan) |
| 9886 | Dicloflex 50mg Gastro-resistant tablet (Ratiopharm UK Ltd) |
| 10792 | Voltarol 50mg Suppository (Novartis Pharmaceuticals UK Ltd) |
| 10898 | Voltarol Ophtha 0.1% eye drops 0.3ml unit dose (Thea Pharmaceuticals Ltd) |
| 10913 | Diclofenac 0.1% eye drops |
| 10917 | Flamrase SR 100mg tablets (Teva UK Ltd) |
| 10978 | Voltarol 25mg Suppository (Novartis Pharmaceuticals UK Ltd) |
| 11168 | Volsaid Retard 100 tablets (Chiesi Ltd) |
| 11215 | Voltarol 25mg suppositories (Novartis Pharmaceuticals UK Ltd) |
| 11322 | Flamrase sr 75mg Modified-release tablet (APS Berk) |
| 11522 | Pennsaid 1.50% Cutaneous solution (Provalis Healthcare Ltd) |
| 11540 | Diclofenac 16mg/ml topical solution |
| 14084 | Diclovol 75mg SR tablets (Arun Pharmaceuticals Ltd) |
| 14085 | Diclovol Retard 100mg tablets (Arun Pharmaceuticals Ltd) |
| 14672 | Defanac 75mg SR tablets (Ranbaxy (UK) Ltd) |
| 14678 | Defanac sr 100mg Modified-release tablet (Ranbaxy (UK) Ltd) |
| 14707 | Defanac Retard 100mg tablets (Ranbaxy (UK) Ltd) |
| 14884 | Voltarol Gel Patch 1% medicated plasters (Novartis Consumer Health UK Ltd) |
| 14901 | Diclofenac 1% transdermal patches |
| 15201 | Volraman 50mg gastro-resistant tablets (LPC Medical (UK) Ltd) |
| 15732 | Diclovol 50mg gastro-resistant tablets (Arun Pharmaceuticals Ltd) |
| 16221 | Diclozip 25mg gastro-resistant tablets (Ashbourne Pharmaceuticals Ltd) |
| 16222 | Diclozip 50mg gastro-resistant tablets (Ashbourne Pharmaceuticals Ltd) |
| 16225 | Dexomon retard 100mg Modified-release tablet (Hillcross Pharmaceuticals Ltd) |
| 16272 | Lofensaid Retard 100 tablets (Opus Pharmaceuticals Ltd) |
| 16286 | Lofensaid Retard 75 tablets (Opus Pharmaceuticals Ltd) |
| 17029 | Rhumalgan CR 75 tablets (Sandoz Ltd) |
| 17030 | Rhumalgan SR 75mg capsules (Sandoz Ltd) |
| 17124 | Dicloflex sr 100mg Tablet (IVAX Pharmaceuticals UK Ltd) |
| 17126 | Fenactol SR 75mg tablets (Discovery Pharmaceuticals) |
| 17128 | Fenactol 50mg gastro-resistant tablets (Discovery Pharmaceuticals) |
| 17491 | Dicloflex sr 75mg Tablet (Ratiopharm UK Ltd) |
| 17525 | Fenactol Retard 100mg tablets (Discovery Pharmaceuticals) |
| 17532 | Dicloflex Retard 100mg tablets (Kent Pharmaceuticals Ltd) |
| 18151 | Voltarol Pain-eze 1.16% Emulgel (GlaxoSmithKline Consumer Healthcare) |
| 18371 | Digenac xl 100mg Modified-release tablet (Genus Pharmaceuticals Ltd) |
| 18448 | Voltarol 12.5mg suppositories (Novartis Pharmaceuticals UK Ltd) |
| 18798 | Lofensaid 50mg gastro-resistant tablets (Opus Pharmaceuticals Ltd) |
| 18921 | Fenactol 25mg gastro-resistant tablets (Discovery Pharmaceuticals) |
| 19382 | Slofenac 75mg SR tablets (Sterwin Medicines) |
| 20105 | Dicloflex 25mg Gastro-resistant tablet (Ratiopharm UK Ltd) |
| 20384 | Flamatak MR 100mg tablets (Actavis UK Ltd) |
| 20395 | Flamatak MR 75mg tablets (Actavis UK Ltd) |
| 20466 | Voltarol Ophtha Multidose 0.1% eye drops (Thea Pharmaceuticals Ltd) |
| 20621 | Dicloflex 75mg SR tablets (Kent Pharmaceuticals Ltd) |
| 20653 | DICLOFENAC SODIUM S/R |
| 20805 | Dicloflex 75mg SR tablets (Teva UK Ltd) |
| 21387 | Diclofenac sodium 50mg gastro-resistant tablets (Mylan) |
| 21444 | Volraman 25mg gastro-resistant tablets (LPC Medical (UK) Ltd) |
| 21610 | Rhumalgan CR 100 tablets (Sandoz Ltd) |
| 21807 | Flamrase 25 EC tablets (Teva UK Ltd) |
| 21824 | Flamrase 50 EC tablets (Teva UK Ltd) |
| 24121 | Diclofenac sodium 25mg gastro-resistant tablets (Actavis UK Ltd) |
| 24122 | Diclofenac sodium 50mg gastro-resistant tablets (Actavis UK Ltd) |
| 24128 | Diclofenac sodium 25mg gastro-resistant tablets (A A H Pharmaceuticals Ltd) |
| 24236 | Slofenac 100mg Modified-release tablet (Sterwin Medicines) |
| 25283 | Valenac ec 50mg Gastro-resistant tablet (Shire Pharmaceuticals Ltd) |
| 25329 | Lofensaid 25mg gastro-resistant tablets (Opus Pharmaceuticals Ltd) |
| 25358 | Defanac 50mg gastro-resistant tablets (Ranbaxy (UK) Ltd) |
| 25361 | Diclovol 25mg gastro-resistant tablets (Arun Pharmaceuticals Ltd) |
| 25362 | Defanac 25mg gastro-resistant tablets (Ranbaxy (UK) Ltd) |
| 25790 | Rhumalgan 25mg Tablet (Lagap) |
| 26165 | Diclofenac sodium 50mg gastro-resistant tablets (A A H Pharmaceuticals Ltd) |
| 26351 | Rheumatac Retard 75 tablets (AMCo) |
| 26631 | Rhumalgan XL 100mg capsules (Sandoz Ltd) |
| 26888 | Difenor xl 100mg Modified-release tablet (IVAX Pharmaceuticals UK Ltd) |
| 27055 | Diclofenac sodium 50mg gastro-resistant tablets (Kent Pharmaceuticals Ltd) |
| 27200 | Diclovol Retard 100mg tablets (Mylan) |
| 27362 | Diclofenac 100mg Modified-release tablet (Actavis UK Ltd) |
| 27677 | Diclofenac 75mg/3ml Injection (Antigen Pharmaceuticals) |
| 28256 | Diclofenac 50mg Tablet (Berk Pharmaceuticals Ltd) |
| 28390 | Valenac ec 25mg Gastro-resistant tablet (Shire Pharmaceuticals Ltd) |
| 28553 | Diclofenac sodium 50mg gastro-resistant tablets (Teva UK Ltd) |
| 28764 | Closteril 100mg Modified-release tablet (Pharmalife Healthcare Services Ltd) |
| 29037 | Valdic 100 Retard tablets (Fannin UK Ltd) |
| 29181 | Dicloflex 75mg SR tablets (Almus Pharmaceuticals Ltd) |
| 29330 | Diclofenac sodium 50mg gastro-resistant tablets (Sandoz Ltd) |
| 29455 | Flexotard MR 100mg tablets (Pfizer Ltd) |
| 29523 | DICLOFENAC SODIUM (3ML) |
| 30282 | Diclofenac 75mg Modified-release tablet (Galen Ltd) |
| 30297 | Diclofenac 50mg Gastro-resistant tablet (Pharmacia Ltd) |
| 30790 | Dicloflex sr 75mg Tablet (Genus Pharmaceuticals Ltd) |
| 30806 | Rhumalgan 50mg Tablet (Lagap) |
| 30849 | Valdic 75 Retard tablets (Fannin UK Ltd) |
| 30923 | Diclofenac 100mg suppositories (A A H Pharmaceuticals Ltd) |
| 30942 | Diclofenac 50mg Tablet (Regent Laboratories Ltd) |
| 31383 | Dexomon 75mg SR tablets (Hillcross Pharmaceuticals Ltd) |
| 31589 | Diclofenac sodium 75mg modified-release tablets (A A H Pharmaceuticals Ltd) |
| 31787 | Econac SR 75mg tablets (AMCo) |
| 31944 | Diclofenac sodium 25mg gastro-resistant tablets (Mylan) |
| 31950 | Diclofenac sodium 50mg gastro-resistant tablets (Sterwin Medicines) |
| 32108 | Diclofenac sodium 25mg gastro-resistant tablets (Teva UK Ltd) |
| 32536 | Diclofenac 25mg Tablet (Berk Pharmaceuticals Ltd) |
| 32601 | Econac 100mg suppositories (AMCo) |
| 32854 | Diclofenac sodium 75mg modified-release capsules (A A H Pharmaceuticals Ltd) |
| 32916 | Diclofenac 75mg Modified-release capsule (Sandoz Ltd) |
| 33457 | Isclofen 50mg Gastro-resistant tablet (Isis Products Ltd) |
| 33559 | Diclofenac 50mg Tablet (C P Pharmaceuticals Ltd) |
| 33645 | Diclofenac 75mg Modified-release tablet (IVAX Pharmaceuticals UK Ltd) |
| 33669 | Diclofenac 50mg Gastro-resistant tablet (Genus Pharmaceuticals Ltd) |
| 33994 | Diclofenac sodium 25mg gastro-resistant tablets (IVAX Pharmaceuticals UK Ltd) |
| 34091 | Diclofenac sodium 25mg gastro-resistant tablets (Sandoz Ltd) |
| 34212 | Diclofenac 75mg Modified-release tablet (Genus Pharmaceuticals Ltd) |
| 34218 | Diclofenac 25mg Gastro-resistant tablet (Pharmacia Ltd) |
| 34271 | Diclofenac sodium 100mg modified-release tablets (A A H Pharmaceuticals Ltd) |
| 34362 | Diclofenac 25mg Gastro-resistant tablet (Genus Pharmaceuticals Ltd) |
| 34487 | Diclofenac sodium 50mg gastro-resistant tablets (IVAX Pharmaceuticals UK Ltd) |
| 34744 | Diclofenac 100mg Modified-release capsule (Sandoz Ltd) |
| 35711 | Dicloflex 25mg gastro-resistant tablets (Teva UK Ltd) |
| 35882 | Diclofenac 0.1% eye drops 0.3ml unit dose preservative free |
| 35893 | Dicloflex Retard 100mg tablets (Almus Pharmaceuticals Ltd) |
| 36486 | Econac XL 100mg tablets (AMCo) |
| 37688 | Diclofenac sodium 1% gel |
| 37763 | Diclofenac 75mg/2ml solution for injection vials |
| 38817 | Diclofenac potassium 12.5mg tablets |
| 38881 | Diclomax SR 75mg capsules (Galen Ltd) |
| 38948 | Diclomax Retard 100mg capsules (Galen Ltd) |
| 38992 | Flamrase 75mg SR tablets (Teva UK Ltd) |
| 39264 | Dicloflex Retard 100mg tablets (Dexcel-Pharma Ltd) |
| 39708 | Diclofenac 4% cutaneous spray |
| 39722 | Voltarol Pain-eze 12.5mg tablets (Novartis Consumer Health UK Ltd) |
| 39823 | Dicloflex 50mg gastro-resistant tablets (Almus Pharmaceuticals Ltd) |
| 39876 | Mobigel 4% spray (Mercury Pharma Group Ltd) |
| 40086 | Acoflam 50mg gastro-resistant tablets (Mercury Pharma Group Ltd) |
| 40756 | Dicloflex 25mg gastro-resistant tablets (Almus Pharmaceuticals Ltd) |
| 42406 | Diclofenac 50mg Gastro-resistant tablet (Almus Pharmaceuticals Ltd) |
| 42455 | Dicloflex Retard 100mg tablets (Teva UK Ltd) |
| 42793 | Diclofenac 100mg Modified-release tablet (IVAX Pharmaceuticals UK Ltd) |
| 42905 | Diclofenac 75mg Modified-release tablet (Actavis UK Ltd) |
| 43045 | Diclofenac potassium 50mg tablets (Accord Healthcare Ltd) |
| 44112 | Voltarol Joint Pain 12.5mg tablets (Novartis Consumer Health UK Ltd) |
| 45213 | Diclofenac 10mg dispersible tablets |
| 45814 | First Resort Double Action Pain Relief 12.5mg tablets (Actavis UK Ltd) |
| 46844 | Dicloflex 75mg SR tablets (Actavis UK Ltd) |
| 47350 | Voltarol Active 4% spray (Novartis Consumer Health UK Ltd) |
| 47501 | Rhumalgan SR 75mg capsules (Almus Pharmaceuticals Ltd) |
| 47820 | Voltarol Pain-eze Extra Strength 25mg tablets (Novartis Consumer Health UK Ltd) |
| 48059 | Diclofenac potassium 50mg tablets (A A H Pharmaceuticals Ltd) |
| 48218 | Dicloflex sr 100mg Tablet (Teva UK Ltd) |
| 48871 | Diclofenac potassium 25mg tablets (Accord Healthcare Ltd) |
| 49059 | Voltarol 50mg dispersible tablets (Lexon (UK) Ltd) |
| 49132 | Voltarol 1.16% Emulgel (Necessity Supplies Ltd) |
| 49788 | Voltarol 1.16% Emulgel (DE Pharmaceuticals) |
| 49862 | Voltarol 1.16% Emulgel (Lexon (UK) Ltd) |
| 50058 | Voltarol 50mg dispersible tablets (DE Pharmaceuticals) |
| 50269 | Arthrotec 75 gastro-resistant tablets (Mawdsley-Brooks & Company Ltd) |
| 50317 | Voltarol 75mg SR tablets (Lexon (UK) Ltd) |
| 50602 | Diclofenac potassium 50mg tablets (Alliance Healthcare (Distribution) Ltd) |
| 50785 | Diclofenac sodium 50mg gastro-resistant tablets (Genesis Pharmaceuticals Ltd) |
| 51099 | Voltarol Rapid 50mg tablets (Mawdsley-Brooks & Company Ltd) |
| 51237 | Voltarol 1.16% Emulgel (Waymade Healthcare Plc) |
| 51293 | Diclofenac potassium 50mg tablets (Phoenix Healthcare Distribution Ltd) |
| 51343 | Voltarol Rapid 25mg tablets (DE Pharmaceuticals) |
| 51808 | Diclofenac 12.5mg/5ml oral solution |
| 52229 | Voltarol 1.16% Emulgel (Sigma Pharmaceuticals Plc) |
| 52338 | Diclofenac potassium 50mg tablets (Focus Pharmaceuticals Ltd) |
| 52389 | Voltarol 50mg suppositories (Sigma Pharmaceuticals Plc) |
| 52956 | Voltarol 1.16% Emulgel (Stephar (U.K.) Ltd) |
| 53164 | Diclofenac sodium 25mg gastro-resistant tablets (Kent Pharmaceuticals Ltd) |
| 53345 | Voltarol Rapid 50mg tablets (Lexon (UK) Ltd) |
| 53384 | Voltarol 50mg dispersible tablets (Mawdsley-Brooks & Company Ltd) |
| 54021 | Voltarol Retard 100mg tablets (Sigma Pharmaceuticals Plc) |
| 54075 | Voltarol 50mg dispersible tablets (Stephar (U.K.) Ltd) |
| 54463 | Diclofenac 50mg Tablet (Approved Prescription Services Ltd) |
| 54518 | Diclofenac sodium 50mg gastro-resistant tablets (Phoenix Healthcare Distribution Ltd) |
| 54660 | Diclofenac sodium 50mg capsules |
| 54906 | Diclofenac 50mg/5ml oral suspension |
| 55099 | Acoflam 100mg Retard tablets (Mercury Pharma Group Ltd) |
| 55913 | Voltarol 50mg suppositories (Lexon (UK) Ltd) |
| 56071 | Voltarol Active 4% spray (Novartis Consumer Health UK Ltd) |
| 56078 | Rhumalgan XL 100mg capsules (Almus Pharmaceuticals Ltd) |
| 56282 | Diclofenac 2.32% gel |
| 56558 | Voltarol 12 Hour Emulgel P 2.32% gel (GlaxoSmithKline Consumer Healthcare) |
| 56898 | Rhumalgan SR 75mg capsules (Actavis UK Ltd) |
| 57006 | Diclofenac sodium 25mg gastro-resistant tablets (Phoenix Healthcare Distribution Ltd) |
| 57045 | Voltarol 50mg dispersible tablets (Waymade Healthcare Plc) |
| 57162 | Diclofenac 50mg dispersible tablets sugar free (DE Pharmaceuticals) |
| 57545 | Voltarol 1.16% Emulgel (Dowelhurst Ltd) |
| 58048 | Diclofenac sodium 50mg gastro-resistant tablets (Waymade Healthcare Plc) |
| 58071 | Voltarol Rapid 50mg tablets (Waymade Healthcare Plc) |
| 58415 | Diclofenac sodium 50mg gastro-resistant / Misoprostol 200microgram tablets (A A H Pharmaceuticals Ltd) |
| 58572 | Diclofenac potassium 25mg tablets (A A H Pharmaceuticals Ltd) |
| 58842 | Misofen 75mg/200microgram gastro-resistant tablets (Morningside Healthcare Ltd) |
| 59289 | Diclofenac sodium 100mg modified-release tablets (AM Distributions (Yorkshire) Ltd) |
| 59595 | Diclofenac 50mg dispersible tablets sugar free (Sigma Pharmaceuticals Plc) |
| 59880 | Diclofenac sodium 75mg modified-release capsules (Waymade Healthcare Plc) |
| 60368 | Diclofenac 10mg/5ml oral solution |
| 60443 | Diclofenac sodium 75mg modified-release capsules (DE Pharmaceuticals) |
| 60666 | Diclofenac sodium 75mg modified-release capsules (Actavis UK Ltd) |
| 60786 | Voltarol 50mg dispersible tablets (Sigma Pharmaceuticals Plc) |
| 61596 | Diclofenac sodium 75mg modified-release capsules (Phoenix Healthcare Distribution Ltd) |
| 61762 | Diclofenac 10mg/5ml oral suspension |
| 62636 | Diclofenac sodium 25mg gastro-resistant tablets (Sterwin Medicines) |
| 62851 | Voltarol 1.16% Emulgel (Mawdsley-Brooks & Company Ltd) |
| 64303 | Diclofenac sodium 25mg gastro-resistant tablets (DE Pharmaceuticals) |
| 64595 | Misofen 50mg/200microgram gastro-resistant tablets (Morningside Healthcare Ltd) |
| 64759 | Diclofenac 50mg/5ml oral solution |
| 65007 | Voltarol 140mg medicated plasters (GlaxoSmithKline Consumer Healthcare) |
| 65179 | Diclofenac 140mg medicated plasters |
| 65528 | Arthrotec 75 gastro-resistant tablets (Lexon (UK) Ltd) |
| 65783 | Diclofenac potassium 50mg tablets (DE Pharmaceuticals) |
| 65877 | Diclofenac sodium 75mg modified-release tablets (Mawdsley-Brooks & Company Ltd) |
| 66123 | Diclofenac sodium 100mg modified-release tablets (Ethigen Ltd) |
| 66577 | Diclofenac sodium 75mg modified-release capsules (Sigma Pharmaceuticals Plc) |
| 67220 | Diclofenac sodium 100mg modified-release tablets (Phoenix Healthcare Distribution Ltd) |
| 68354 | Diclofenac sodium 100mg modified-release capsules (Actavis UK Ltd) |
| 68849 | Diclofenac 12.5mg/5ml oral suspension |
| 69046 | Diclofenac 0.074% mouthwash sugar free |
| 69477 | Voltarol Rapid 50mg tablets (DE Pharmaceuticals) |
| 69582 | Voltarol 100mg suppositories (Lexon (UK) Ltd) |
| 69584 | Diclofenac sodium 100mg modified-release tablets (Sigma Pharmaceuticals Plc) |
| 69683 | Diclofenac 2.32% gel (Colorama Pharmaceuticals Ltd) |
| 70145 | Voltarol Rapid 50mg tablets (Stephar (U.K.) Ltd) |
| 70438 | Arthrotec 50 gastro-resistant tablets (Lexon (UK) Ltd) |
| 70468 | Voltarol Rapid 50mg tablets (Sigma Pharmaceuticals Plc) |
| 71064 | Diclofenac sodium 75mg modified-release capsules (Waymade Healthcare Plc) |
| 71088 | Arthrotec 50 gastro-resistant tablets (Mawdsley-Brooks & Company Ltd) |
| 71100 | Diclofenac sodium 50mg gastro-resistant tablets (Sigma Pharmaceuticals Plc) |
| 71117 | Diclomax SR 75mg capsules (DE Pharmaceuticals) |
| 71307 | Diclofenac sodium 100mg modified-release capsules (DE Pharmaceuticals) |
| 71362 | Diclomax Retard 100mg capsules (Waymade Healthcare Plc) |
| 71364 | Voltarol 1.16% Emulgel P (Sigma Pharmaceuticals Plc) |
| 72113 | Diclofenac 0.74mg/ml mouthwash sugar free (Morningside Healthcare Ltd) |
| 72396 | Diclofenac sodium 50mg gastro-resistant tablets (Medreich Plc) |
| 72546 | Diclofenac sodium 100mg modified-release capsules (A A H Pharmaceuticals Ltd) |
| 6895 | Duloxetine 60mg gastro-resistant capsules |
| 7122 | Duloxetine 30mg gastro-resistant capsules |
| 7147 | Duloxetine 40mg gastro-resistant capsules |
| 7153 | Duloxetine 20mg gastro-resistant capsules |
| 13151 | Cymbalta 30mg gastro-resistant capsules (Eli Lilly and Company Ltd) |
| 14803 | Yentreve 40mg gastro-resistant capsules (Eli Lilly and Company Ltd) |
| 14849 | Cymbalta 60mg gastro-resistant capsules (Eli Lilly and Company Ltd) |
| 16969 | Yentreve 20mg gastro-resistant capsules (Eli Lilly and Company Ltd) |
| 51383 | Duloxetine 60mg gastro-resistant capsules (Sigma Pharmaceuticals Plc) |
| 62688 | Duloxetine 30mg gastro-resistant capsules (Sigma Pharmaceuticals Plc) |
| 63216 | Cymbalta 60mg gastro-resistant capsules (Mawdsley-Brooks & Company Ltd) |
| 63370 | Duloxetine 30mg gastro-resistant capsules (Mawdsley-Brooks & Company Ltd) |
| 63763 | Duloxetine 60mg gastro-resistant capsules (A A H Pharmaceuticals Ltd) |
| 64442 | Duloxetine 60mg gastro-resistant capsules (Teva UK Ltd) |
| 65165 | Duloxetine 20mg gastro-resistant capsules (DE Pharmaceuticals) |
| 65618 | Duloxetine 30mg gastro-resistant capsules (A A H Pharmaceuticals Ltd) |
| 65809 | Duloxetine 30mg gastro-resistant capsules (Actavis UK Ltd) |
| 65888 | Duloxetine 60mg gastro-resistant capsules (Actavis UK Ltd) |
| 65892 | Duloxetine 60mg gastro-resistant capsules (Mawdsley-Brooks & Company Ltd) |
| 66405 | Duloxetine 60mg gastro-resistant capsules (DE Pharmaceuticals) |
| 66412 | Duloxetine 30mg gastro-resistant capsules (DE Pharmaceuticals) |
| 67564 | Duloxetine 20mg gastro-resistant capsules (Actavis UK Ltd) |
| 68096 | Duloxetine 60mg gastro-resistant capsules (Zentiva) |
| 69428 | Duloxetine 60mg gastro-resistant capsules (Alliance Healthcare (Distribution) Ltd) |
| 69752 | Duloxetine 60mg gastro-resistant capsules (Creo Pharma Ltd) |
| 69965 | Duloxetine 60mg gastro-resistant capsules (Consilient Health Ltd) |
| 70063 | Duloxetine 20mg gastro-resistant capsules (A A H Pharmaceuticals Ltd) |
| 70405 | Duloxetine 30mg gastro-resistant capsules (Teva UK Ltd) |
| 70728 | Duloxetine 30mg gastro-resistant capsules (Alliance Healthcare (Distribution) Ltd) |
| 71669 | Duloxetine 20mg gastro-resistant capsules (Zentiva) |
| 72211 | Duloxetine 60mg gastro-resistant capsules (Dr Reddy's Laboratories (UK) Ltd) |
| 650 | Etoricoxib 60mg tablets |
| 5812 | Etoricoxib 90mg tablets |
| 5938 | Etoricoxib 120mg tablets |
| 6464 | Arcoxia 60mg tablets (Grunenthal Ltd) |
| 6498 | Arcoxia 90mg tablets (Grunenthal Ltd) |
| 9822 | Arcoxia 120mg tablets (Grunenthal Ltd) |
| 37562 | Arcoxia 30mg tablets (Grunenthal Ltd) |
| 37587 | Etoricoxib 30mg tablets |
| 51284 | Arcoxia 60mg tablets (Sigma Pharmaceuticals Plc) |
| 51874 | Arcoxia 30mg tablets (Lexon (UK) Ltd) |
| 53576 | Arcoxia 120mg tablets (DE Pharmaceuticals) |
| 56584 | Arcoxia 60mg tablets (Lexon (UK) Ltd) |
| 62251 | Arcoxia 90mg tablets (Sigma Pharmaceuticals Plc) |
| 62658 | Arcoxia 120mg tablets (Waymade Healthcare Plc) |
| 62843 | Arcoxia 90mg tablets (Lexon (UK) Ltd) |
| 64521 | Arcoxia 90mg tablets (Mawdsley-Brooks & Company Ltd) |
| 66486 | Arcoxia 90mg tablets (DE Pharmaceuticals) |
| 660 | Gabapentin 100mg capsules |
| 1584 | Gabapentin 300mg capsules |
| 4781 | Gabapentin 400mg capsules |
| 5221 | Gabapentin 600mg tablets |
| 6304 | Neurontin 300mg capsules (Pfizer Ltd) |
| 7538 | Gabapentin 800mg tablets |
| 9979 | Gabapentin 600mg tablets and Gabapentin 300mg capsules |
| 10007 | Neurontin 100mg capsules (Pfizer Ltd) |
| 16215 | Neurontin 400mg capsules (Pfizer Ltd) |
| 17564 | Neurontin 600mg tablets (Pfizer Ltd) |
| 18211 | Gabapentin 250mg/5ml oral solution |
| 25815 | Gabapentin 400mg/5ml oral suspension |
| 27454 | Gabapentin 300mg capsules (Teva UK Ltd) |
| 28713 | Neurontin 800mg tablets (Pfizer Ltd) |
| 34506 | Gabapentin 100mg capsules (Teva UK Ltd) |
| 34606 | Gabapentin 100mg capsules (A A H Pharmaceuticals Ltd) |
| 34716 | Gabapentin 300mg capsules (A A H Pharmaceuticals Ltd) |
| 34946 | Gabapentin 600mg tablets (Teva UK Ltd) |
| 44022 | Gabapentin 250mg/5ml oral suspension |
| 44187 | Gabapentin 600mg tablets (A A H Pharmaceuticals Ltd) |
| 44261 | Gabapentin 250mg/5ml Oral solution (Boots Pharmaceuticals Ltd) |
| 47579 | Gabapentin 100mg capsules (Sandoz Ltd) |
| 48035 | Gabapentin 300mg capsules (Mylan) |
| 48060 | Gabapentin 300mg capsules (Sandoz Ltd) |
| 51118 | Gabapentin 300mg capsules (Ranbaxy (UK) Ltd) |
| 53296 | Gabapentin 300mg capsules (Actavis UK Ltd) |
| 53784 | Gabapentin 300mg/5ml oral solution |
| 54609 | Gabapentin 50mg/ml oral solution sugar free |
| 55008 | Gabapentin 6% gel |
| 55535 | Gabapentin 300mg capsules (Almus Pharmaceuticals Ltd) |
| 55624 | Gabapentin 300mg capsules (Bristol Laboratories Ltd) |
| 57120 | Gabapentin 100mg capsules (Mylan) |
| 57527 | Neurontin 300mg capsules (Dowelhurst Ltd) |
| 57649 | Gabapentin 300mg capsules (Arrow Generics Ltd) |
| 58162 | Gabapentin 600mg/5ml oral solution |
| 58382 | Gabapentin 100mg capsules (Actavis UK Ltd) |
| 58383 | Gabapentin 400mg capsules (Almus Pharmaceuticals Ltd) |
| 58472 | Gabapentin 300mg/5ml oral suspension |
| 58960 | Gabapentin 100mg capsules (Zentiva) |
| 59147 | Gabapentin 600mg tablets (Zentiva) |
| 59196 | Gabapentin 400mg/5ml oral solution |
| 60389 | Gabapentin 300mg capsules (Creo Pharma Ltd) |
| 61266 | Gabapentin 300mg capsules (Waymade Healthcare Plc) |
| 63375 | Gabapentin 200mg/5ml oral solution |
| 63432 | Gabapentin 300mg capsules (Zentiva) |
| 64213 | Gabapentin 600mg tablets (DE Pharmaceuticals) |
| 64302 | Gabapentin 300mg capsules (Alliance Healthcare (Distribution) Ltd) |
| 64306 | Gabapentin 50mg/ml oral solution sugar free (Alliance Healthcare (Distribution) Ltd) |
| 64981 | Gabapentin 100mg/5ml oral solution |
| 66617 | Gabapentin 300mg capsules (Medreich Plc) |
| 67091 | Gabapentin 50mg/5ml oral solution |
| 67969 | Gabapentin 300mg capsules (DE Pharmaceuticals) |
| 68047 | Gabapentin 300mg capsules (Phoenix Healthcare Distribution Ltd) |
| 68049 | Gabapentin 100mg capsules (Almus Pharmaceuticals Ltd) |
| 69914 | Gabapentin 300mg capsules (Almus Pharmaceuticals Ltd) |
| 70247 | Gabapentin 600mg tablets (Aurobindo Pharma Ltd) |
| 70459 | Gabapentin 400mg capsules (Waymade Healthcare Plc) |
| 70506 | Gabapentin 500mg/5ml oral solution |
| 70738 | Gabapentin 600mg tablets (Actavis UK Ltd) |
| 70954 | Gabapentin 300mg capsules (Brown & Burk UK Ltd) |
| 71013 | Gabapentin 100mg capsules (Bristol Laboratories Ltd) |
| 15 | Ibuprofen 400mg tablets |
| 112 | Ibuprofen 5% cream |
| 215 | IBUPROFEN 200 MG CAP |
| 332 | Ibuprofen 5% gel |
| 345 | IBUPROFEN S/R 300 MG CAP |
| 360 | Brufen 100mg/5ml syrup (Mylan) |
| 392 | Ibuprofen 200mg modified-release capsules |
| 402 | Nurofen 200mg Tablet (Crookes Healthcare Ltd) |
| 407 | Brufen 600mg effervescent granules sachets (Mylan) |
| 416 | Ibuprofen 200mg tablets |
| 586 | Ibuprofen 200mg Capsule |
| 647 | Ibuprofen 100mg/5ml oral suspension |
| 784 | Ibuprofen 300mg modified-release capsules |
| 849 | Ibumed 400mg Tablet (Medipharma Ltd) |
| 1030 | Junifen 100mg/5ml Oral suspension (Crookes Healthcare Ltd) |
| 1086 | Ibuprofen 600mg tablets |
| 1156 | Ibugel 5% gel (Dermal Laboratories Ltd) |
| 1270 | Ibuleve 5% gel (Dendron Ltd) |
| 1392 | Ibuprofen 800mg modified-release tablets |
| 1468 | Ibuprofen 200mg Soluble tablet |
| 1621 | Brufen 200mg tablets (Abbott Laboratories Ltd) |
| 1739 | Brufen 400mg tablets (Mylan) |
| 2129 | Brufen retard tabs 800mg Modified-release tablet (Abbott Laboratories Ltd) |
| 2622 | Ibuprofen 800mg tablets |
| 2693 | Proflex 5% cream (Novartis Consumer Health UK Ltd) |
| 2938 | Ibuprofen 100mg/5ml Oral suspension |
| 3597 | Nurofen 200mg Soluble tablet (Crookes Healthcare Ltd) |
| 3599 | Ibuprofen 600mg effervescent granules sachets |
| 4216 | Brufen 600mg tablets (Mylan) |
| 4298 | Nurofen 200mg Tablet (Crookes Healthcare Ltd) |
| 4309 | Ibuprofen lysine 200mg tablets |
| 4648 | Ibuspray 5% spray (Dermal Laboratories Ltd) |
| 4731 | Nurofen for children 100mg/5ml Oral suspension (Reckitt Benckiser Healthcare (UK) Ltd) |
| 4762 | Ibuleve Sports 5% gel (Dendron Ltd) |
| 4911 | Ibuprofen 400mg Granules |
| 5648 | Ibuprofen 200mg orodispersible tablets sugar free |
| 5767 | Ibuprofen 10% gel |
| 5896 | Ibuleve Maximum Strength 10% gel (Dendron Ltd) |
| 6853 | Ibutop Ralgex Ibuprofen 5% gel (SSL International Plc) |
| 7058 | Calprofen 100mg/5ml Oral suspension (McNeil Products Ltd) |
| 7141 | Ibugel Forte 10% gel (Dermal Laboratories Ltd) |
| 7261 | Cuprofen 5% gel (SSL International Plc) |
| 7535 | Nurofen 200mg Capsule (Crookes Healthcare Ltd) |
| 8401 | Motrin 400mg tablets (Pfizer Ltd) |
| 8510 | Ibuprofen 5% spray |
| 9201 | Ibuleve 5% spray (Dendron Ltd) |
| 10149 | Ibuprofen 200mg capsules |
| 10209 | Ibufem 200mg tablets (Galpharm International Ltd) |
| 10265 | Fenbid 5% gel (AMCo) |
| 10785 | Fenbid 300mg Spansules (Mercury Pharma Group Ltd) |
| 11550 | Nurofen Meltlets 200mg tablets (Reckitt Benckiser Healthcare (UK) Ltd) |
| 11980 | Cuprofen 400mg Tablet (SSL International Plc) |
| 12776 | Ibumousse 5% (Dermal Laboratories Ltd) |
| 13083 | Deep Relief gel (The Mentholatum Company Ltd) |
| 13807 | Deep relief ibuprofen 5% Gel (Mentholatum Company) |
| 14333 | Ibuprofen 400mg capsules |
| 14385 | Cuprofen 200mg Tablet (SSL International Plc) |
| 14570 | Ibuleve 5% Mousse (Dendron Ltd) |
| 15068 | Arthrofen 400 tablets (Ashbourne Pharmaceuticals Ltd) |
| 15363 | Nurofen Cold and Flu tablets (Reckitt Benckiser Healthcare (UK) Ltd) |
| 15767 | Ibuprofen 5% foam |
| 15930 | Ibuprofen 5% / Levomenthol 3% gel |
| 16001 | Ibuprofen 200mg tablets (A A H Pharmaceuticals Ltd) |
| 16192 | Motrin 200mg Tablet (Pharmacia Ltd) |
| 16193 | Motrin 800mg tablets (Pfizer Ltd) |
| 17068 | Nurofen Maximum Strength 10% gel (Reckitt Benckiser Healthcare (UK) Ltd) |
| 17201 | Motrin 600mg tablets (Pfizer Ltd) |
| 18196 | Orbifen for children 100mg/5ml Oral suspension (Orbis Consumer Products Ltd) |
| 18364 | Ibular 400mg Tablet (Lagap) |
| 18527 | Mandafen 400mg tablets (M & A Pharmachem Ltd) |
| 18812 | Nurofen meltlets lemon 200mg Orodispersible tablet (Reckitt Benckiser Healthcare (UK) Ltd) |
| 18820 | Fenpaed 100mg/5ml Oral suspension (Pinewood Healthcare) |
| 19036 | Arthrofen 200 tablets (Ashbourne Pharmaceuticals Ltd) |
| 19046 | Ibuprofen 400mg tablets (A A H Pharmaceuticals Ltd) |
| 19398 | Radian B Ibuprofen 5% gel (Thornton & Ross Ltd) |
| 19575 | Proflex 200mg Tablet (Novartis Consumer Health UK Ltd) |
| 20442 | Nurofen 5% gel (Reckitt Benckiser Healthcare (UK) Ltd) |
| 20907 | Sudafed Sinus Pressure & Pain tablets (McNeil Products Ltd) |
| 20967 | Phorpain 5% gel (AMCo) |
| 20978 | Anadin Ultra liquid capsules (Wyeth Consumer Healthcare) |
| 21045 | Ibumetin 400mg Tablet (Alfred Benzon (UK) Ltd) |
| 21811 | Lidifen 200mg Tablet (Berk Pharmaceuticals Ltd) |
| 21813 | Lidifen 400mg Tablet (Berk Pharmaceuticals Ltd) |
| 21815 | Arthrofen 600 tablets (Ashbourne Pharmaceuticals Ltd) |
| 21821 | Lidifen f 600mg Tablet (Berk Pharmaceuticals Ltd) |
| 22206 | Nurofen Long Lasting 300mg capsules (Crookes Healthcare Ltd) |
| 22283 | Lemsip flu 12 hr Modified-release capsule (Reckitt Benckiser Healthcare (UK) Ltd) |
| 23425 | Nurofen Migraine Pain 342mg tablets (Reckitt Benckiser Healthcare (UK) Ltd) |
| 24086 | Ibuprofen 5% gel (Thornton & Ross Ltd) |
| 24305 | Ibufac 400mg Tablet (DDSA Pharmaceuticals Ltd) |
| 24469 | Cuprofen for Children 100mg/5ml oral suspension (SSL International Plc) |
| 24887 | Nurofen Advance 200mg tablets (Crookes Healthcare Ltd) |
| 25205 | Ibuprofen 100mg/5ml oral suspension 5ml sachets sugar free (Thornton & Ross Ltd) |
| 25257 | Advil 200mg tablets (Wyeth Consumer Healthcare) |
| 25619 | Nurofen 400mg Tablet (Crookes Healthcare Ltd) |
| 25794 | Isisfen 400mg Tablet (Isis Products Ltd) |
| 25800 | Feverfen 100mg/5ml oral suspension (Wise Pharmaceuticals Ltd) |
| 26095 | Ibuprofen lysine 400mg tablets |
| 26159 | Fenbid Forte 10% gel (AMCo) |
| 26970 | Ibuprofen 100mg/5ml oral suspension sugar free (Teva UK Ltd) |
| 27438 | Pseudoephedrine 45mg with ibuprofen 300mg modified-release capsule |
| 27782 | Ibuprofen 400mg tablets (Teva UK Ltd) |
| 27783 | Ibuprofen 400mg tablets sugar coated (Actavis UK Ltd) |
| 27968 | Apsifen 400mg Tablet (Approved Prescription Services Ltd) |
| 28168 | Nurofen Recovery 200mg orodispersible tablets (Reckitt Benckiser Healthcare (UK) Ltd) |
| 28172 | Ibuprofen 300mg / Pseudoephedrine 45mg modified-release capsules |
| 28348 | Ibuprofen 200mg tablets (Teva UK Ltd) |
| 28479 | Nurofen Back Pain SR 300mg capsules (Reckitt Benckiser Healthcare (UK) Ltd) |
| 28519 | Pseudoephedrine 30mg with ibuprofen 200mg tablet |
| 28522 | Ibuprofen 200mg / Pseudoephedrine hydrochloride 30mg tablets |
| 28822 | Ibuprofen with pseudoephedrine hc 400mg + 60mg Liquid |
| 28888 | Galprofen Long Lasting 200mg capsules (Galpharm International Ltd) |
| 29068 | Nurofen Extra Strength 400mg capsules (Reckitt Benckiser Healthcare (UK) Ltd) |
| 29232 | Care ibuprofen 5% Gel (Thornton & Ross Ltd) |
| 29316 | Care ibuprofen 400mg Tablet (Thornton & Ross Ltd) |
| 29332 | Ibuprofen 100mg/5ml oral suspension sugar free (Sandoz Ltd) |
| 29345 | Ibuprofen 100mg/5ml Oral suspension (Hillcross Pharmaceuticals Ltd) |
| 29352 | Ibuprofen 100mg/5ml oral suspension sugar free (Vantage) |
| 29524 | Ibumetin 600mg Tablet (Alfred Benzon (UK) Ltd) |
| 29587 | Ebufac 400mg Tablet (DDSA Pharmaceuticals Ltd) |
| 29704 | Paxofen 200mg Tablet (M A Steinhard Ltd) |
| 29749 | Ibuprofen 200mg tablets (Ranbaxy (UK) Ltd) |
| 30164 | Lemsip Cold and Flu Sinus 12 Hr Ibuprofen + Pseudoephedrine modified-release capsules (Reckitt Benckiser Healthcare (UK) Ltd) |
| 30243 | Ibuprofen 200mg effervescent tablets |
| 30382 | Ibuprofen 200mg Tablet (C P Pharmaceuticals Ltd) |
| 30724 | Galprofen 100mg/5ml oral suspension (Galpharm International Ltd) |
| 30811 | Proflex 300mg Modified-release capsule (Novartis Consumer Health UK Ltd) |
| 30892 | Mandafen for Children 100mg/5ml oral suspension sugar free (M & A Pharmachem Ltd) |
| 31054 | Phorpain Maximum Strength 10% gel (AMCo) |
| 31469 | Apsifen -f 600mg Tablet (Approved Prescription Services Ltd) |
| 31482 | Apsifen 200mg Tablet (Approved Prescription Services Ltd) |
| 32100 | Ibuprofen 600mg tablets (A A H Pharmaceuticals Ltd) |
| 32136 | Ibular 200mg Tablet (Lagap) |
| 32242 | Ibuprofen 400mg tablets (Sterwin Medicines) |
| 32365 | Relcofen 400mg tablets (Actavis UK Ltd) |
| 32366 | Relcofen 200mg Tablet (Actavis UK Ltd) |
| 32509 | Anadin Ibuprofen 200mg tablets (Pfizer Consumer Healthcare Ltd) |
| 32704 | Advil cold and sinus 200mg+30mg Tablet (Wyeth Consumer Healthcare) |
| 32862 | Ibuprofen 100mg/5ml oral suspension sugar free (Thornton & Ross Ltd) |
| 32875 | Ibuprofen 400mg tablets (Sandoz Ltd) |
| 33357 | Pacifene 200mg tablets (Sussex Pharmaceutical Ltd) |
| 33589 | Ibuprofen 400mg tablets (Thornton & Ross Ltd) |
| 33704 | Ibuprofen 100mg/5ml oral suspension sugar free (A A H Pharmaceuticals Ltd) |
| 33785 | Galprofen 200mg tablets (Galpharm International Ltd) |
| 33935 | Nurofen Maximum Strength Migraine Pain 684mg caplets (Reckitt Benckiser Healthcare (UK) Ltd) |
| 34354 | Ibuprofen 200mg tablets (Vantage) |
| 34359 | Ibuprofen 400mg tablets (Vantage) |
| 34425 | Ibuprofen 400mg Tablet (Family Health) |
| 34447 | Ibuprofen 200mg tablets (Thornton & Ross Ltd) |
| 34527 | Ibuprofen 200mg tablets (Zentiva) |
| 34536 | Ibuprofen 400mg tablets (IVAX Pharmaceuticals UK Ltd) |
| 34550 | Ibuprofen 400mg tablets film coated (Actavis UK Ltd) |
| 34621 | Ibuprofen 200mg Tablet (Nucare Plc) |
| 34663 | Ibuprofen 100mg/5ml Oral suspension (Neo Laboratories Ltd) |
| 34729 | Ibuprofen 400mg tablets (OBG Pharmaceuticals Ltd) |
| 34757 | Ibuprofen 400mg Tablet (Unichem) |
| 34850 | Ibuprofen 600mg tablets (Teva UK Ltd) |
| 34889 | Ibuprofen 400mg Tablet (Celltech Pharma Europe Ltd) |
| 34911 | Ibuprofen 200mg Tablet (Celltech Pharma Europe Ltd) |
| 34931 | Ibuprofen 200mg Tablet (Regent Laboratories Ltd) |
| 34961 | Ibuprofen 600mg tablets (Sandoz Ltd) |
| 34980 | Ibuprofen 200mg tablets sugar coated (Actavis UK Ltd) |
| 35129 | Biatain-Ibu Non-Adhesive dressing 15cm x 15cm square (Coloplast Ltd) |
| 35265 | Nurofen for children 3 months to 9 years 100mg/5ml Oral suspension (Reckitt Benckiser Healthcare (UK) Ltd) |
| 35292 | Nurofen 200mg liquid capsules (Reckitt Benckiser Healthcare (UK) Ltd) |
| 35749 | Radian B Ibuprofen Massage stick (Thornton & Ross Ltd) |
| 35890 | Nurofen 200mg caplets (Reckitt Benckiser Healthcare (UK) Ltd) |
| 36030 | Biatain-Ibu Soft-Hold dressing 15cm x 15cm square (Coloplast Ltd) |
| 36329 | Ibuprofen 10% gel (Thornton & Ross Ltd) |
| 36597 | Hedex Ibuprofen 200mg tablets (Omega Pharma Ltd) |
| 36606 | Manorfen 400mg tablets (The Manor Drug Company (Nottingham) Ltd) |
| 36650 | Nurofen 200mg tablets (Reckitt Benckiser Healthcare (UK) Ltd) |
| 36787 | Nurofen Express 684mg caplets (Reckitt Benckiser Healthcare (UK) Ltd) |
| 37002 | Nurofen Express 200mg liquid capsules (Reckitt Benckiser Healthcare (UK) Ltd) |
| 37053 | Migrafen 200mg tablets (Chatfield Laboratories) |
| 37094 | Cuprofen 200mg tablets (SSL International Plc) |
| 37235 | Ibuprofen 100mg/5ml / Pseudoephedrine 15mg/5ml oral suspension sugar free |
| 37253 | Anadin ultra double strength 400mg Capsule (Wyeth Consumer Healthcare) |
| 37502 | Ibuprofen 10mg/2ml solution for infusion ampoules |
| 37553 | Ibucalm 400mg tablets (Aspar Pharmaceuticals Ltd) |
| 37648 | Nurofen Express 400mg liquid capsules (Reckitt Benckiser Healthcare (UK) Ltd) |
| 37731 | Nurofen Express 342mg caplets (Reckitt Benckiser Healthcare (UK) Ltd) |
| 37805 | Polyurethane foam Film dressing 15cmx15cm |
| 37850 | Ibucalm Ibuprofen Pain Relief 5% gel (Aspar Pharmaceuticals Ltd) |
| 37972 | Ibuleve Speed Relief 5% gel (Dendron Ltd) |
| 38182 | Orbifen Cold & Flu oral suspension (Orbis Consumer Products Ltd) |
| 38332 | Ibucalm 200mg tablets (Aspar Pharmaceuticals Ltd) |
| 38493 | Anadin Joint Pain 200mg tablets (Pfizer Consumer Healthcare Ltd) |
| 38527 | Proflex Pain Relief 5% cream (Novartis Consumer Health UK Ltd) |
| 39019 | Brufen Retard 800mg tablets (Mylan) |
| 39354 | Galpharm ibuprofen for children 100mg/5ml Oral suspension (Galpharm International Ltd) |
| 39502 | Ibuprofen sodium dihydrate 200mg tablets |
| 39758 | Nurofen Express 256mg caplets (Reckitt Benckiser Healthcare (UK) Ltd) |
| 39873 | Cuprofen Maximum Strength 400mg tablets (SSL International Plc) |
| 40083 | Ibuprofen 200mg caplets (Galpharm International Ltd) |
| 40253 | Ibuprofen 600mg Tablet (Sovereign Medical Ltd) |
| 40394 | Advil 400mg Tablet (Wyeth Consumer Healthcare) |
| 40516 | Anadin Ultra 200mg capsules (Pfizer Consumer Healthcare Ltd) |
| 41513 | Ibuprofen 200mg tablets (IVAX Pharmaceuticals UK Ltd) |
| 41701 | Ibuprofen 600mg tablets (Actavis UK Ltd) |
| 42108 | Ibuprofen 200mg tablets (OBG Pharmaceuticals Ltd) |
| 42397 | Nurofen Express 256mg tablets (Reckitt Benckiser Healthcare (UK) Ltd) |
| 43032 | Inoven 200mg Tablet (Janssen-Cilag Ltd) |
| 43096 | Ibuleve Speed Relief 5% spray (Dendron Ltd) |
| 43426 | Ibuprofen 5% gel (A A H Pharmaceuticals Ltd) |
| 43456 | Anadin LiquiFast 400mg capsules (Pfizer Consumer Healthcare Ltd) |
| 43904 | Feminax Express 342mg tablets (Bayer Plc) |
| 43911 | Ibuprofen 600mg Tablet (C P Pharmaceuticals Ltd) |
| 44233 | Nurofen for children baby 100mg/5ml Oral suspension (Reckitt Benckiser Healthcare (UK) Ltd) |
| 44483 | Nurofen Express 512mg tablets (Reckitt Benckiser Healthcare (UK) Ltd) |
| 44730 | Mentholatum Ibuprofen 5% gel (The Mentholatum Company Ltd) |
| 44892 | Sudafed sinus pressure & pain Tablet (McNeil Products Ltd) |
| 45145 | Ibuleve Speed Relief Max Strength 10% gel (Dendron Ltd) |
| 45216 | Ibuprofen 400mg Tablet (C P Pharmaceuticals Ltd) |
| 45320 | Ibuprofen 200mg tablets (Sandoz Ltd) |
| 45331 | Ibuprofen 200mg Tablet (Co-Pharma Ltd) |
| 45842 | Ibuprofen 600mg Tablet (Celltech Pharma Europe Ltd) |
| 45988 | Ibuprofen 200mg / Phenylephrine 5mg tablets |
| 46141 | Nurofen Tension Headache 342mg caplets (Reckitt Benckiser Healthcare (UK) Ltd) |
| 46342 | Medifen 3with months 100mg/5ml Oral suspension (SSL International Plc) |
| 46638 | Paracetamol 500mg / Ibuprofen 200mg tablets |
| 46860 | Anadin LiquiFast 200mg effervescent tablets (Pfizer Consumer Healthcare Ltd) |
| 46904 | Nuromol 200mg/500mg tablets (Reckitt Benckiser Healthcare (UK) Ltd) |
| 46921 | Ibuprofen 400mg tablets (Ranbaxy (UK) Ltd) |
| 46942 | Ibuprofen 600mg tablets (IVAX Pharmaceuticals UK Ltd) |
| 47401 | Ibuprofen 5% gel (Galpharm International Ltd) |
| 48062 | Ibuprofen 200mg Tablet (Wockhardt UK Ltd) |
| 48084 | Ibuprofen 200mg/5ml oral suspension |
| 48138 | Ibuprofen 200mg tablets (Aspar Pharmaceuticals Ltd) |
| 48326 | Ibuprofen 100mg/5ml oral suspension sugar free |
| 48546 | Ibuprofen 400mg caplets (Bristol Laboratories Ltd) |
| 48562 | Ibuprofen 100mg/5ml oral suspension 5ml sachets sugar free |
| 48568 | Boots Rapid Ibuprofen lysine 342mg tablets (The Boots Company Plc) |
| 48644 | Ibuprofen 400mg caplets (Lloyds Pharmacy Ltd) |
| 48675 | Ibuprofen Pain Relief Maximum Strength 10% gel (Numark Ltd) |
| 48738 | Nurofen for Children 100mg/5ml oral suspension orange (Reckitt Benckiser Healthcare (UK) Ltd) |
| 49133 | Nurofen for Children 100mg/5ml oral suspension strawberry (Reckitt Benckiser Healthcare (UK) Ltd) |
| 49266 | Ibuprofen for Children 100mg/5ml oral suspension (Galpharm International Ltd) |
| 49277 | Ibuprofen 200mg caplets (Bristol Laboratories Ltd) |
| 49432 | Calprofen 100mg/5ml oral suspension (McNeil Products Ltd) |
| 50117 | Brufen 100mg/5ml syrup (Lexon (UK) Ltd) |
| 50266 | Ibuprofen 200mg caplets (The Boots Company Plc) |
| 50314 | Brufen 600mg effervescent granules sachets (DE Pharmaceuticals) |
| 50363 | Nurofen for Children Singles 100mg/5ml oral suspension 5ml sachets strawberry (Reckitt Benckiser Healthcare (UK) Ltd) |
| 50628 | Ibuprofen 400mg caplets (The Boots Company Plc) |
| 50652 | Junior Ibuprofen 100mg/5ml oral suspension (Numark Ltd) |
| 50813 | Ibuderm 5% gel (Dermal Laboratories Ltd) |
| 51614 | Ibuprofen 200mg caplets (Lloyds Pharmacy Ltd) |
| 51769 | Nurofen for Children Singles 100mg/5ml oral suspension 5ml sachets orange (Reckitt Benckiser Healthcare (UK) Ltd) |
| 51828 | Ibuprofen 100mg/5ml oral suspension sugar free (Kent Pharmaceuticals Ltd) |
| 51923 | Ibuprofen 10% gel (A A H Pharmaceuticals Ltd) |
| 51943 | Orbifen For Children 100mg/5ml oral suspension (Orbis Consumer Products Ltd) |
| 52009 | Ibuprofen 200mg capsules (Galpharm International Ltd) |
| 52154 | Ibuprofen 200mg tablets (Galpharm International Ltd) |
| 52617 | Ibuprofen 100mg/5ml oral suspension sugar free (Sigma Pharmaceuticals Plc) |
| 53331 | Ibuprofen 100mg/5ml oral suspension sugar free (Alliance Healthcare (Distribution) Ltd) |
| 53397 | Brufen 100mg/5ml syrup (Mawdsley-Brooks & Company Ltd) |
| 53604 | Ibuprofen 200mg capsules (Numark Ltd) |
| 53803 | Ibuprofen 200mg capsules (Kent Pharmaceuticals Ltd) |
| 54137 | Ibuprofen 400mg tablets (Aspar Pharmaceuticals Ltd) |
| 54514 | Ibuprofen lysine 400mg oral powder sachets |
| 55009 | Brufen 600mg effervescent granules sachets (Necessity Supplies Ltd) |
| 55153 | Nurofen Express Soluble 400mg oral powder sachets (Reckitt Benckiser Healthcare (UK) Ltd) |
| 55233 | Ibuprofen 400mg Tablet (Nucare Plc) |
| 55313 | Ibuprofen 400mg tablets (Boston Healthcare Ltd) |
| 55434 | Ibuprofen 400mg tablets (Bristol Laboratories Ltd) |
| 56039 | Ibuprofen 600mg tablets (Waymade Healthcare Plc) |
| 56213 | Ibuprofen 400mg tablets sugar coated (Kent Pharmaceuticals Ltd) |
| 56441 | Calprofen 100mg/5ml oral suspension 5ml sachets (McNeil Products Ltd) |
| 57112 | Ibuprofen 400mg tablets (Alliance Healthcare (Distribution) Ltd) |
| 58652 | Ibuprofen 600mg tablets (Sigma Pharmaceuticals Plc) |
| 58888 | Ibuprofen 10% gel (Vantage) |
| 59067 | Ibuprofen 200mg capsules (AM Distributions (Yorkshire) Ltd) |
| 59203 | Brufen 100mg/5ml syrup (Sigma Pharmaceuticals Plc) |
| 59502 | Nurofen for Children Cold, Pain and Fever Strawberry Flavour 100mg/5ml oral suspension (Reckitt Benckiser Healthcare (UK) Ltd) |
| 59553 | Ibuprofen 200mg tablets (Alliance Healthcare (Distribution) Ltd) |
| 59562 | Ibuprofen 600mg tablets (Boston Healthcare Ltd) |
| 60035 | Ibuprofen 200mg tablets film coated (Actavis UK Ltd) |
| 60510 | Nurofen for Children Cold, Pain and Fever Orange Flavour 100mg/5ml oral suspension (Reckitt Benckiser Healthcare (UK) Ltd) |
| 61260 | Ibuprofen 10% gel (AMCo) |
| 61878 | Nurofen Express Period Pain 200mg capsules (Reckitt Benckiser Healthcare (UK) Ltd) |
| 61953 | Ibuprofen 200mg caplets (Wockhardt UK Ltd) |
| 62238 | Ibuprofen 200mg capsules (Colorama Pharmaceuticals Ltd) |
| 62892 | Ibuprofen 200mg tablets (Wockhardt UK Ltd) |
| 63036 | Ibuprofen 400mg capsules (AM Distributions (Yorkshire) Ltd) |
| 63079 | Ibuprofen 400mg tablets (Waymade Healthcare Plc) |
| 63132 | Ibuprofen 5% gel (Alliance Healthcare (Distribution) Ltd) |
| 65025 | Ibuprofen 400mg tablets (Crescent Pharma Ltd) |
| 65121 | Fenpaed 100mg/5ml oral suspension (Pinewood Healthcare) |
| 65471 | Ibuprofen 200mg tablets (Almus Pharmaceuticals Ltd) |
| 65514 | Ibuprofen 200mg tablets (Boston Healthcare Ltd) |
| 65591 | Ibuprofen 200mg tablets (Crescent Pharma Ltd) |
| 66194 | Care Ibuprofen for Children 100mg/5ml oral suspension (Thornton & Ross Ltd) |
| 66247 | Ibuprofen 100mg chewable capsules |
| 66353 | Nurofen Day & Night Cold & Flu 200mg/5mg tablets (Reckitt Benckiser Healthcare (UK) Ltd) |
| 66461 | Boots Ibuprofen Max Strength 10% gel (The Boots Company Plc) |
| 66544 | Ibuprofen 400mg tablets (DE Pharmaceuticals) |
| 66567 | Ibuprofen sodium dihydrate 400mg tablets |
| 66648 | Ibuprofen 400mg tablets (Almus Pharmaceuticals Ltd) |
| 67594 | Ibuprofen 400mg tablets (Kent Pharmaceuticals Ltd) |
| 67740 | Ibuprofen 600mg tablets (Fannin UK Ltd) |
| 68018 | Ibuprofen 200mg tablets (Mawdsley-Brooks & Company Ltd) |
| 68220 | Ibuprofen 200mg capsules (Ennogen Healthcare Ltd) |
| 68582 | Ibuprofen 200mg tablets (DE Pharmaceuticals) |
| 69018 | Nurofen Joint & Back Pain Relief 256mg tablets (Reckitt Benckiser Healthcare (UK) Ltd) |
| 69271 | Boots Ibuprofen 5% gel (The Boots Company Plc) |
| 69935 | Ibuprofen 5% / Levomenthol 3% gel (Colorama Pharmaceuticals Ltd) |
| 70299 | Ibuprofen 200mg capsules (Bell,Sons & Co (Druggists) Ltd) |
| 70821 | Nurofen Joint & Back Pain Relief 5% gel (Reckitt Benckiser Healthcare (UK) Ltd) |
| 70878 | Nurofen Joint & Back Pain Relief 200mg capsules (Reckitt Benckiser Healthcare (UK) Ltd) |
| 71041 | Ibuprofen 600mg tablets (Bristol Laboratories Ltd) |
| 71072 | Ibuprofen 100mg/5ml oral suspension sugar free (Phoenix Healthcare Distribution Ltd) |
| 71314 | Ibuprofen 200mg tablets (Kent Pharmaceuticals Ltd) |
| 71374 | Galpharm Ibuprofen For Children 100mg/5ml oral suspension 5ml sachets (Galpharm International Ltd) |
| 71584 | Boots Ibuprofen 3 Months Plus 100mg/5ml oral suspension strawberry (The Boots Company Plc) |
| 71779 | Boots Ibuprofen Long Lasting 200mg capsules (The Boots Company Plc) |
| 71949 | Flarin 200mg capsules (infirst Healthcare Ltd) |
| 71968 | Ibuprofen 400mg capsules (Ennogen Healthcare Ltd) |
| 72156 | Nurofen Cold & Flu Relief 200mg/5mg tablets (Reckitt Benckiser Healthcare (UK) Ltd) |
| 129 | Naprosyn 500mg suppositories (Roche Products Ltd) |
| 140 | Naproxen 500mg suppositories |
| 661 | Naproxen 250mg tablets |
| 807 | Naproxen 500mg tablets |
| 1043 | Naproxen sodium 275mg tablets |
| 1866 | Naprosyn 500mg tablets (Atnahs Pharma UK Ltd) |
| 2197 | Naproxen 375mg Tablet |
| 2288 | Naprosyn 250mg tablets (Atnahs Pharma UK Ltd) |
| 2391 | NAPROXEN 250 MG CAP |
| 3053 | Naproxen 500mg gastro-resistant tablets |
| 3431 | Naproxen 250mg gastro-resistant tablets |
| 3432 | Naproxen 375mg gastro-resistant tablets |
| 3496 | Nycopren 250mg gastro-resistant tablets (Ardern Healthcare Ltd) |
| 3817 | Synflex 275mg tablets (Roche Products Ltd) |
| 3901 | Naprosyn EC 500mg tablets (Atnahs Pharma UK Ltd) |
| 3972 | Naprosyn EC 250mg tablets (Atnahs Pharma UK Ltd) |
| 4045 | Naprosyn EC 375mg tablets (Atnahs Pharma UK Ltd) |
| 4320 | Naprosyn 125mg/5ml oral suspension (Roche Products Ltd) |
| 4984 | Naproxen 500mg tablets and Misoprostol 200microgram tablets |
| 5268 | Naproxen 500mg modified-release tablets |
| 5407 | Naproxen 125mg/5ml oral suspension |
| 8663 | Naprosyn S/R 500mg tablets (Roche Products Ltd) |
| 15023 | Naproxen 375mg Modified-release tablet |
| 15104 | Naproxen 500mg Granules |
| 15180 | Naproxen and misoprostol 500mgwith200microgram combined Tablet |
| 17165 | Nycopren 500mg gastro-resistant tablets (Ardern Healthcare Ltd) |
| 17733 | Condrotec 500mg+200microgram Tablet (Pharmacia Ltd) |
| 19007 | Naprosyn 500mg Granules (Roche Products Ltd) |
| 20385 | Arthrosin 500 tablets (Ashbourne Pharmaceuticals Ltd) |
| 20704 | NAPROXEN SODIUM |
| 21816 | Pranoxen continus 500mg Tablet (Napp Pharmaceuticals Ltd) |
| 21840 | Arthrosin 250 tablets (Ashbourne Pharmaceuticals Ltd) |
| 21843 | Pranoxen continus 375mg Tablet (Napp Pharmaceuticals Ltd) |
| 23121 | Arthroxen 500mg Tablet (C P Pharmaceuticals Ltd) |
| 23323 | Prosaid 500mg Tablet (BHR Pharmaceuticals Ltd) |
| 24007 | Valrox 500mg Tablet (Shire Pharmaceuticals Ltd) |
| 24020 | Valrox 250mg Tablet (Shire Pharmaceuticals Ltd) |
| 25092 | NAPROXEN |
| 25341 | Arthrosin EC 250 tablets (Ashbourne Pharmaceuticals Ltd) |
| 25342 | Arthrosin EC 500 tablets (Ashbourne Pharmaceuticals Ltd) |
| 25750 | Rheuflex 250mg Tablet (Goldshield Pharmaceuticals Ltd) |
| 26216 | Timpron 500mg Tablet (Berk Pharmaceuticals Ltd) |
| 26231 | Timpron 500mg Gastro-resistant tablet (Berk Pharmaceuticals Ltd) |
| 26242 | Timpron 250mg Tablet (Berk Pharmaceuticals Ltd) |
| 27366 | Naproxen 500mg gastro-resistant tablets (Teva UK Ltd) |
| 28255 | Naproxen 250mg tablets (Wockhardt UK Ltd) |
| 28313 | NAPROXEN |
| 28816 | Rheuflex 500mg Tablet (Goldshield Pharmaceuticals Ltd) |
| 30168 | Arthroxen 250mg Tablet (C P Pharmaceuticals Ltd) |
| 30982 | Naproxen 500mg gastro-resistant tablets (Actavis UK Ltd) |
| 31429 | Timpron 250mg Gastro-resistant tablet (Berk Pharmaceuticals Ltd) |
| 31945 | Naproxen 500mg Gastro-resistant tablet (Sterwin Medicines) |
| 33111 | Prosaid 250mg Tablet (BHR Pharmaceuticals Ltd) |
| 34143 | Naprosyn 375 Tablet (Roche Products Ltd) |
| 34289 | Naproxen 250mg gastro-resistant tablets (Mylan) |
| 34290 | Naproxen 250mg gastro-resistant tablets (Teva UK Ltd) |
| 34610 | Naproxen 500mg gastro-resistant tablets (Mylan) |
| 34670 | Naproxen 250mg Gastro-resistant tablet (Galen Ltd) |
| 34738 | Naproxen 250mg gastro-resistant tablets (A A H Pharmaceuticals Ltd) |
| 34743 | Naproxen 500mg gastro-resistant tablets (A A H Pharmaceuticals Ltd) |
| 34769 | Naproxen 500mg tablets (A A H Pharmaceuticals Ltd) |
| 34922 | Naproxen 500mg Tablet (Berk Pharmaceuticals Ltd) |
| 34923 | Naproxen 250mg Tablet (Berk Pharmaceuticals Ltd) |
| 34977 | Naproxen 500mg Gastro-resistant tablet (Galen Ltd) |
| 38511 | Feminax Ultra 250mg gastro-resistant tablets (Bayer Plc) |
| 39085 | Naproxen 250mg tablets (A A H Pharmaceuticals Ltd) |
| 39317 | Naproxen 500mg tablets (Wockhardt UK Ltd) |
| 39693 | Naproxen 200mg/5ml oral suspension |
| 40401 | Naproxen 250mg gastro-resistant tablets (IVAX Pharmaceuticals UK Ltd) |
| 44800 | Naproxen 500mg / Esomeprazole 20mg modified-release tablets |
| 44986 | Vimovo 500mg/20mg modified-release tablets (AstraZeneca UK Ltd) |
| 45262 | Naproxen Oral solution |
| 46440 | Naproxen 500mg Tablet (M & A Pharmachem Ltd) |
| 46848 | Naproxen 500mg Gastro-resistant tablet (Almus Pharmaceuticals Ltd) |
| 47994 | Naproxen 250mg Gastro-resistant tablet (Almus Pharmaceuticals Ltd) |
| 48161 | Naproxen 500mg Tablet (Almus Pharmaceuticals Ltd) |
| 51242 | Naproxen 500mg tablets (Pfizer Ltd) |
| 51360 | Naproxen 250mg tablets (Accord Healthcare Ltd) |
| 51829 | Naproxen 250mg tablets (Kent Pharmaceuticals Ltd) |
| 52931 | Naproxen 500mg gastro-resistant tablets (Kent Pharmaceuticals Ltd) |
| 53626 | Naproxen 500mg gastro-resistant tablets (Alliance Healthcare (Distribution) Ltd) |
| 53700 | Naproxen 250mg gastro-resistant tablets (Alliance Healthcare (Distribution) Ltd) |
| 53980 | Naproxen 250mg tablets (Phoenix Healthcare Distribution Ltd) |
| 54304 | Naproxen 500mg tablets (Actavis UK Ltd) |
| 54476 | Naproxen 500mg gastro-resistant tablets (Genesis Pharmaceuticals Ltd) |
| 54783 | Naproxen 250mg tablets (Teva UK Ltd) |
| 55454 | Naproxen 500mg tablets (Kent Pharmaceuticals Ltd) |
| 55486 | Naproxen 500mg tablets (Teva UK Ltd) |
| 55505 | Naproxen 250mg gastro-resistant tablets (Kent Pharmaceuticals Ltd) |
| 55894 | Naproxen 500mg gastro-resistant tablets (Phoenix Healthcare Distribution Ltd) |
| 56106 | Naproxen 500mg/5ml oral suspension |
| 56554 | Naproxen 250mg/5ml oral suspension |
| 56762 | Naproxen 100mg/5ml oral suspension |
| 56925 | Naproxen 250mg tablets (Actavis UK Ltd) |
| 58213 | Naproxen 500mg tablets (Milpharm Ltd) |
| 58221 | Naproxen 250mg gastro-resistant tablets (Actavis UK Ltd) |
| 58708 | Naproxen 500mg gastro-resistant tablets (Ranbaxy (UK) Ltd) |
| 59246 | Naproxen 500mg tablets (Accord Healthcare Ltd) |
| 59878 | Naproxen 250mg gastro-resistant tablets (Almus Pharmaceuticals Ltd) |
| 60115 | Naproxen 250mg tablets (Alliance Healthcare (Distribution) Ltd) |
| 60408 | Naproxen 500mg gastro-resistant tablets (Waymade Healthcare Plc) |
| 61695 | Naproxen 500mg tablets (Alliance Healthcare (Distribution) Ltd) |
| 63357 | Naproxen 250mg tablets (Almus Pharmaceuticals Ltd) |
| 63843 | Naproxen 250mg tablets (Milpharm Ltd) |
| 65348 | Naproxen 250mg gastro-resistant tablets (Genesis Pharmaceuticals Ltd) |
| 65862 | Naproxen 250mg effervescent tablets sugar free |
| 65952 | Naproxen 500mg gastro-resistant tablets (Almus Pharmaceuticals Ltd) |
| 66993 | Naproxen 125mg/5ml oral suspension sugar free |
| 67117 | Stirlescent 250mg effervescent tablets (Stirling Anglian Pharmaceuticals Ltd) |
| 67363 | Naproxen 250mg gastro-resistant tablets (Sigma Pharmaceuticals Plc) |
| 67768 | Naproxen 250mg tablets (Mylan) |
| 68097 | Naproxen 250mg tablets (Crescent Pharma Ltd) |
| 68470 | Naproxen 25mg/ml oral suspension sugar free (Orion Pharma (UK) Ltd) |
| 68685 | Naproxen 250mg tablets (Waymade Healthcare Plc) |
| 69645 | Naproxen 50mg/ml oral suspension (Thornton & Ross Ltd) |
| 69828 | Naproxen 50mg/ml oral suspension (Alliance Healthcare (Distribution) Ltd) |
| 70393 | Naproxen 250mg tablets (DE Pharmaceuticals) |
| 70698 | Naproxen 250mg gastro-resistant tablets (Ranbaxy (UK) Ltd) |
| 70828 | Naproxen 500mg tablets (DE Pharmaceuticals) |
| 71709 | Naproxen 50mg/ml oral suspension (A A H Pharmaceuticals Ltd) |
| 72030 | Naproxen 500mg tablets (Sigma Pharmaceuticals Plc) |
| 72047 | Naproxen 250mg gastro-resistant tablets (Phoenix Healthcare Distribution Ltd) |
| 3794 | Acupan 30mg Tablet (3M Health Care Ltd) |
| 4016 | Nefopam 30mg tablets |
| 7488 | Acupan 20mg/1ml solution for injection ampoules (3M Health Care Ltd) |
| 11942 | Nefopam 20mg/1ml solution for injection ampoules |
| 37979 | Acupan 30mg tablets (Meda Pharmaceuticals Ltd) |
| 64313 | Nefopam 30mg/5ml oral suspension |
| 65308 | Acupan 30mg tablets (DE Pharmaceuticals) |
| 66814 | Nefopam 30mg tablets (Alliance Healthcare (Distribution) Ltd) |
| 68271 | Nefopam 30mg tablets (Meda Pharmaceuticals Ltd) |
| 70428 | Acupan 30mg tablets (Lexon (UK) Ltd) |
| 2936 | Motival 10mg/500microgram tablets (Sanofi) |
| 3183 | Nortriptyline 10mg tablets |
| 3903 | Nortriptyline 25mg tablets |
| 4118 | Nortriptyline 10mg Capsule |
| 7677 | Allegron 10mg tablets (King Pharmaceuticals Ltd) |
| 7678 | Nortriptyline 25mg Capsule |
| 7780 | Nortriptyline 10mg / Fluphenazine 500microgram tablets |
| 8493 | Motipress tablets (Sanofi-Synthelabo Ltd) |
| 8640 | Allegron 25mg tablets (King Pharmaceuticals Ltd) |
| 12194 | NORTRIPTYLINE 10 MG ELI |
| 12353 | Aventyl 25mg Capsule (Eli Lilly and Company Ltd) |
| 12549 | Aventyl 10mg/5ml Liquid (Eli Lilly and Company Ltd) |
| 14578 | Nortriptyline 30mg / Fluphenazine 1.5mg tablets |
| 17183 | Aventyl 10mg Capsule (Eli Lilly and Company Ltd) |
| 20571 | Fluphenazine with nortriptyline 500microgramswith10mg Tablet |
| 39145 | Nortriptyline 10mg/5ml Liquid |
| 48216 | Nortriptyline 25mg tablets (A A H Pharmaceuticals Ltd) |
| 55970 | Nortriptyline 10mg tablets (King Pharmaceuticals Ltd) |
| 63276 | Nortriptyline 25mg tablets (Alliance Healthcare (Distribution) Ltd) |
| 65237 | Nortriptyline 10mg tablets (A A H Pharmaceuticals Ltd) |
| 66201 | Nortriptyline 25mg tablets (Sigma Pharmaceuticals Plc) |
| 68228 | Nortriptyline 10mg/5ml oral suspension |
| 69317 | Nortriptyline 50mg tablets |
| 72626 | Nortriptyline 10mg/5ml oral solution |
| 7 | Paracetamol 500mg tablets |
| 139 | Paracetamol 500mg capsules |
| 175 | PARACETAMOL 1 GM SUP |
| 258 | Panadol 500mg tablets (GlaxoSmithKline Consumer Healthcare) |
| 262 | Paracetamol 250mg/5ml oral suspension |
| 759 | Calpol infant 120mg/5ml Liquid (McNeil Products Ltd) |
| 855 | Disprol 120mg/5ml Oral suspension (Reckitt Benckiser Healthcare (UK) Ltd) |
| 890 | Paramax sachets (Zentiva) |
| 892 | Paramax tablets (Zentiva) |
| 899 | Calpol Six Plus 250mg/5ml oral suspension (McNeil Products Ltd) |
| 901 | Calpol 120mg/5ml Liquid (Pfizer Consumer Healthcare Ltd) |
| 1068 | Calpol Paediatric 120mg/5ml oral suspension sugar free (McNeil Products Ltd) |
| 1147 | Paracetamol 120mg/5ml oral suspension |
| 1404 | Paracetamol 120mg/5ml oral suspension paediatric (Rosemont Pharmaceuticals Ltd) |
| 1609 | Paracetamol 500mg soluble tablets |
| 1689 | Paracetamol 120mg/5ml oral suspension sugar free |
| 1862 | Paracetamol 500mg/5ml oral suspension sugar free |
| 1865 | PARACETAMOL 1 GM TAB |
| 1946 | PARACETAMOL 500 MG SUP |
| 1999 | Paracetamol 125mg suppositories |
| 2034 | Alvedon 125mg suppositories (Intrapharm Laboratories Ltd) |
| 2055 | Paracetamol 120mg/5ml oral solution paediatric |
| 2306 | Paracetamol 500mg / Metoclopramide 5mg tablets |
| 2344 | Paracetamol 120mg suppositories |
| 2546 | PARACETAMOL SOLUBLE TAB |
| 2586 | Disprol 120mg/5ml Oral suspension (Reckitt Benckiser Healthcare (UK) Ltd) |
| 2800 | Panadol 500mg Soluble tablet (GlaxoSmithKline Consumer Healthcare) |
| 3073 | Paracetamol 500mg / Pseudoephedrine hydrochloride 60mg tablets |
| 3074 | Paracetamol 120mg soluble tablets sugar free |
| 3313 | Calpol six plus 250mg/5ml Oral suspension sugar free (McNeil Products Ltd) |
| 3316 | Disprol Paracetamol 120mg soluble tablets (Reckitt Benckiser Healthcare (UK) Ltd) |
| 3514 | Paracetamol 500mg / Metoclopramide 5mg effervescent powder sachets sugar free |
| 4186 | Paracetamol 250mg/5ml oral suspension sugar free |
| 4196 | Paracetamol 240mg suppositories |
| 4203 | Medised Liquid (SSL International Plc) |
| 4600 | Chlormezanone with paracetamol tablets |
| 4633 | Paracetamol 120mg/5ml oral solution paediatric sugar free |
| 5239 | Paracetamol 1g oral powder sachets |
| 5243 | Paracetamol 500mg suppositories |
| 5323 | Paracetamol 250mg suppositories |
| 6571 | Paracetamol 240mg oral powder sachets sugar free |
| 6699 | Paracetamol 60mg suppositories |
| 7205 | Panadol 500mg capsules (GlaxoSmithKline Consumer Healthcare) |
| 7489 | Panadol baby & infant 120mg/5ml Oral suspension (GlaxoSmithKline Consumer Healthcare) |
| 7520 | Anadin Extra soluble tablets (Pfizer Consumer Healthcare Ltd) |
| 7621 | PARACETAMOL 300 MG TAB |
| 7650 | PARACETAMOL 125 MG ELI |
| 7668 | PARACETAMOL 250 MG TAB |
| 7769 | ASPIRIN/PARACETAMOL TAB |
| 8020 | PARACETAMOL 125 MG MIX |
| 8091 | PARACETAMOL CO TAB |
| 8093 | PARACETAMOL SOLUBLE TAB |
| 8139 | Midrid 325mg/65mg capsules (DHP Healthcare Ltd) |
| 8259 | PARACETAMOL 500 MG ELI |
| 8292 | PARACETAMOL 240 MG ELI |
| 8700 | PARACETAMOL 125 MG TAB |
| 8930 | Sudafed Non-Drowsy Congestion Cold and Flu tablets (McNeil Products Ltd) |
| 9193 | Paracetamol 500mg with sodium bicarbonate 1342 mg effervescent tablet |
| 9231 | Calpol infant 120mg/5ml Oral suspension (McNeil Products Ltd) |
| 9239 | Paracetamol 120 mg capsule |
| 9271 | Paracetamol 125mg/5ml syrup |
| 9329 | Paracetamol 100mg/ml oral solution sugar free |
| 9476 | Domperamol tablets (Servier Laboratories Ltd) |
| 9581 | Isometheptene mucate with paracetamol 65mg+325mg Capsule |
| 9712 | Paracetamol 250mg orodispersible tablets sugar free |
| 9914 | Calpol Six Plus Fastmelts 250mg tablets (McNeil Products Ltd) |
| 10123 | Paracetamol 120mg/5ml / Diphenhydramine 12.5mg/5ml oral solution |
| 10196 | Solpadeine Headache tablets (GlaxoSmithKline Consumer Healthcare) |
| 10293 | Sinutab Tablet (Pfizer Consumer Healthcare Ltd) |
| 10399 | PARACETAMOL/ORPHENADRINE CITRATE TAB |
| 10748 | Tramil 500mg Capsule (Wyeth Consumer Healthcare) |
| 10901 | Paracetamol with promethazine hydrochloride 120mg+1.5mg/5ml suspension |
| 10993 | Medinol 120mg/5ml Oral suspension (SSL International Plc) |
| 11614 | Paracetamol 500mg / Domperidone 10mg tablets |
| 11925 | Medised For Children oral solution (SSL International Plc) |
| 11986 | Medinol 120mg/5ml Oral suspension (SSL International Plc) |
| 12332 | PARACETAMOL 240 MG SUS |
| 12394 | Lobak Tablet (Sanofi-Synthelabo Ltd) |
| 12447 | Norgesic Tablet (3M Health Care Ltd) |
| 12778 | Metoclopramide with paracetamol 5mg + 500mg Tablet |
| 12869 | Metoclopramide with paracetamol 5mg + 500mg Sachets |
| 13193 | Lemsip Cold & Flu Lemon oral powder sachets (Reckitt Benckiser Healthcare (UK) Ltd) |
| 14151 | Medised infant 120mg + 12.5mg / 5ml Oral solution (Seton Scholl Healthcare Plc) |
| 14384 | Flu strength all in one Oral solution (A A H Pharmaceuticals Ltd) |
| 14551 | Infadrops 100mg/ml liquid (Mercury Pharma Group Ltd) |
| 14560 | PARACETAMOL 60 MG SUP |
| 14627 | Paracetamol 325mg / Isometheptene 65mg capsules |
| 14655 | Paracetamol 500mg with phenylpropanolamine 12.5 mg tablet |
| 14829 | Paracetamol 500mg / Caffeine 65mg tablets |
| 14899 | Alvedon 250mg suppositories (Intrapharm Laboratories Ltd) |
| 15238 | Paracetamol 500mg tablets (Zentiva) |
| 15241 | Sudafed non-drowsy dual relief Capsule (Pfizer Consumer Healthcare Ltd) |
| 15352 | ASPIRIN & PARACETAMOL TAB |
| 15367 | Anadin Extra tablets (Pfizer Consumer Healthcare Ltd) |
| 15439 | PARACETAMOL 150 MG SUP |
| 15461 | PARACETAMOL 100 MG SUP |
| 15845 | Panadol NightPain tablets (GlaxoSmithKline Consumer Healthcare) |
| 16058 | Paracetamol 500mg / Pseudoephedrine hydrochloride 30mg tablets |
| 16106 | Paracetamol 500mg / Caffeine 65mg effervescent tablets sugar free |
| 17412 | Paracetamol 500mg / Diphenhydramine 25mg tablets |
| 17651 | Beechams All-In-One oral solution (GlaxoSmithKline Consumer Healthcare) |
| 17773 | Paracetamol 500mg with phenylpropanolamine 25mg tablet |
| 17845 | Paracetamol 500mg with methionine 100 mg tablet |
| 18441 | Alvedon 60mg suppositories (Intrapharm Laboratories Ltd) |
| 18583 | Paracetamol 500mg with pseudoephedrine 30mg & pholcodine 5mg capsule |
| 18675 | Paracetamol with caffeine capsule |
| 18799 | Flu strength hot lemon 1g Powder (A A H Pharmaceuticals Ltd) |
| 18919 | Lemsip cold and flu sachets (Reckitt Benckiser Healthcare (UK) Ltd) |
| 18922 | Solpadeine Headache soluble tablets (Omega Pharma Ltd) |
| 19139 | Paracetamol 500mg Suppository (Distriphar (UK)) |
| 19255 | Paracetamol 200mg with aspirin 300mg & caffeine 45mg tablet |
| 19626 | Triogesic Tablet (Novartis Consumer Health UK Ltd) |
| 19917 | Pseudoephedrine 60mg with paracetamol 500mg tablet |
| 20068 | Paracetamol 250mg/5ml oral suspension sugar free |
| 20116 | Paradote 100mg/500mg tablets (Sinclair IS Pharma Plc) |
| 20194 | PARACETAMOL 75 MG SUS |
| 20403 | Paracetamol powder |
| 20495 | Lemsip Cold & Flu Breathe Easy oral powder sachets (Reckitt Benckiser Healthcare (UK) Ltd) |
| 20627 | Sinutab Non-Drowsy tablets (McNeil Products Ltd) |
| 20646 | Triogesic Oral solution (Novartis Consumer Health UK Ltd) |
| 20650 | Aspirin 300mg / Paracetamol 200mg dispersible tablets sugar free |
| 21370 | PARACETAMOL/PHENYLPROPANOLAMINE/PHOLCODI 150 MG SYR |
| 21491 | PARACETAMOL 30 MG SUP |
| 21754 | PARACETAMOL 20 MG SUP |
| 21770 | Paracetamol 200mg with aspirin 300mg dispersible tablet |
| 22014 | Disprol paracetamol 120mg/5ml Oral suspension (Reckitt Benckiser Healthcare (UK) Ltd) |
| 22062 | Lemsip Max Cold & Flu Lemon powder sachets (Reckitt Benckiser Healthcare (UK) Ltd) |
| 22073 | Paracetamol 350mg & Ascorbic acid 50mg powder |
| 22085 | Lemsip Max Flu Lemon oral powder sachets (Reckitt Benckiser Healthcare (UK) Ltd) |
| 22125 | Lemsip cold and flu combined relief 300mg+5mg+25mg Capsule (Reckitt Benckiser Healthcare (UK) Ltd) |
| 22236 | Paracetamol with promethazine hydrochloride 120mg+1.5mg/5ml suspension sugar free colour free |
| 22288 | Medinol 250mg/5ml Oral suspension (SSL International Plc) |
| 22305 | Disprin Extra dispersible tablets (Reckitt Benckiser Healthcare (UK) Ltd) |
| 22380 | Paracetamol 1g/100ml solution for infusion vials |
| 22988 | Day nurse with phenylpropanolamine and dextromethorphan Liquid (GlaxoSmithKline Consumer Healthcare) |
| 23052 | Promethazine hydrochloride 1.5mg with paracetamol 120mg/5ml oral solution colour free and sugar free |
| 23075 | Beechams All-In-One tablets (GlaxoSmithKline Consumer Healthcare) |
| 23077 | Paracetamol 120mg/5ml Oral suspension sugar free (Pinewood Healthcare) |
| 23107 | Night Nurse oral solution (GlaxoSmithKline Consumer Healthcare) |
| 23108 | Day nurse with phenylpropanolamine and dextromethorphan Capsule (GlaxoSmithKline Consumer Healthcare) |
| 23114 | Panaleve plus 120mg/5ml Oral suspension sugar free (Pinewood Healthcare) |
| 23274 | PARACETAMOL |
| 23318 | Sinutab nightime Tablet (Warner Lambert UK Ltd) |
| 23617 | Paracetamol 50mg oral powder sachets sugar free |
| 23716 | Paracetamol 500mg caplets (IVAX Pharmaceuticals UK Ltd) |
| 23840 | Panasorb 500mg Tablet (Sanofi-Synthelabo Ltd) |
| 23841 | Safapryn Tablet (Pfizer Ltd) |
| 23909 | Paracetamol 650mg / Ascorbic acid 50mg oral powder sachets |
| 24000 | Mandanol 500mg tablets (M & A Pharmachem Ltd) |
| 24075 | Mandanol 500mg caplets (M & A Pharmachem Ltd) |
| 24133 | Panadol Extra soluble tablets (GlaxoSmithKline Consumer Healthcare) |
| 24283 | Dozol liquid (M & A Pharmachem Ltd) |
| 24309 | Paracetamol 120mg with aspirin 165.3mg & caffeine 60mg powder |
| 24400 | PARACETAMOL |
| 24445 | Paracetamol with promethazine hydrochloride tablet |
| 24480 | Night Nurse capsules (GlaxoSmithKline Consumer Healthcare) |
| 24534 | Anadin Paracetamol 500mg tablets (Pfizer Consumer Healthcare Ltd) |
| 24803 | SALICYLAMIDE/PARACETAMOL MG TAB |
| 24947 | Co-methiamol 100mg/500mg tablets |
| 25562 | Lemsip Max Sinus capsules (Reckitt Benckiser Healthcare (UK) Ltd) |
| 25607 | Paracetamol 500mg with methionine 250 mg tablet |
| 25895 | Hedex 500mg tablets (Omega Pharma Ltd) |
| 25941 | PARACETAMOL/PHENYLPROPANOLAMINE HYD/PHEN 300 MG TAB |
| 26133 | Panadol Extra tablets (GlaxoSmithKline Consumer Healthcare) |
| 26294 | Benylin day and night Cold treatment (Pfizer Consumer Healthcare Ltd) |
| 26295 | Day Nurse oral solution (GlaxoSmithKline Consumer Healthcare) |
| 26572 | Beechams Flu-Plus caplets (GlaxoSmithKline Consumer Healthcare) |
| 26746 | Hedex Extra tablets (Omega Pharma Ltd) |
| 26967 | Alka-Seltzer XS effervescent tablets (Bayer Plc) |
| 26988 | Paracets 500mg Tablet (Sussex Pharmaceutical Ltd) |
| 27064 | Paldesic paracetamol 120mg/5ml oral suspension (Rosemont Pharmaceuticals Ltd) |
| 27128 | Paracets plus Capsule (Sussex Pharmaceutical Ltd) |
| 27145 | Day Nurse capsules (GlaxoSmithKline Consumer Healthcare) |
| 27197 | Femerital Tablet (Boehringer Mannheim UK Ltd) |
| 27337 | Benylin four flu Liquid (Pfizer Consumer Healthcare Ltd) |
| 27435 | Paracetamol 200mg with aspirin 300mg & caffeine 45mg soluble tablet |
| 27446 | Paracetamol 120mg/5ml oral solution paediatric sugar free (Pinewood Healthcare) |
| 27452 | Paracetamol 500mg soluble tablets (Zentiva) |
| 27459 | Paracetamol 500mg caplets (Zentiva) |
| 27492 | Phenylpropanolamine with paracetamol oral solution |
| 27553 | PARACETAMOL |
| 27804 | Benylin four flu Tablet (Pfizer Consumer Healthcare Ltd) |
| 27837 | Beechams Decongestant Plus with Paracetamol capsules (GlaxoSmithKline Consumer Healthcare) |
| 27856 | Midrid (rpr) Capsule (Rhone-Poulenc Rorer Ltd) |
| 27979 | Lemsip max cold and flu Capsule (Reckitt Benckiser Healthcare (UK) Ltd) |
| 28211 | Paracetamol 250mg/5ml oral suspension sugar free |
| 28236 | Beechams Cold and Flu Hot Lemon oral powder 6g sachets (GlaxoSmithKline Consumer Healthcare) |
| 28237 | Lemsip Max Cold & Flu capsules (Reckitt Benckiser Healthcare (UK) Ltd) |
| 28344 | Paracetamol 500mg caplets (Wockhardt UK Ltd) |
| 28346 | Paracetamol 500mg Tablet (M & A Pharmachem Ltd) |
| 28636 | Paracetamol 120mg/5ml Oral solution (Rosemont Pharmaceuticals Ltd) |
| 28712 | Galpamol 120mg/5ml Oral suspension (Galpharm International Ltd) |
| 28792 | Medised colour and sf 120mg+1.5mg/5ml Liquid (SSL International Plc) |
| 28878 | Paracetamol 120mg/5ml oral solution paediatric (A A H Pharmaceuticals Ltd) |
| 28937 | Benylin four flu 1000mg+25mg+12mg Powder (Pfizer Consumer Healthcare Ltd) |
| 28946 | Lemsip Max Cold & Flu Direct Lemon oral powder sachets (Reckitt Benckiser Healthcare (UK) Ltd) |
| 28955 | Panadol ActiFast 500mg tablets (GlaxoSmithKline Consumer Healthcare) |
| 29074 | PARACETAMOL & CAFFEINE TAB |
| 29375 | Pameton Tablet (GlaxoSmithKline UK Ltd) |
| 29401 | Otrivine Mu-Cron tablets (Novartis Consumer Health UK Ltd) |
| 29558 | Maximum flu strength Tablet (Wockhardt UK Ltd) |
| 29670 | Phenylpropanolamine 12.5mg with paracetamol 500mg tablet |
| 29951 | Andrews Plus effervescent powder sachets (GlaxoSmithKline Consumer Healthcare) |
| 30070 | Paraclear extra Tablet (Roche Consumer Health) |
| 30116 | Beechams Cold and Flu Hot Lemon and Honey oral powder 6g sachets (GlaxoSmithKline Consumer Healthcare) |
| 30590 | Medised Tablet (SSL International Plc) |
| 30958 | Resolve effervescent granules 8.09g sachets (G.R. Lane Health Products Ltd) |
| 30984 | Paracetamol 250mg/5ml oral suspension (A A H Pharmaceuticals Ltd) |
| 30994 | De Witt's Analgesic Pills (E.C. DeWitt and Company Ltd) |
| 31151 | Cafadol Tablet (Typharm Ltd) |
| 31196 | Paracetamol 500mg tablets (IVAX Pharmaceuticals UK Ltd) |
| 31257 | Paracetamol 500mg caplets (Galpharm International Ltd) |
| 31499 | Paracetamol with aspirin tablet |
| 31717 | Lemsip max cold and flu lemon Sachets (Reckitt Benckiser Healthcare (UK) Ltd) |
| 31998 | Lemsip Cold & Flu Blackcurrant oral powder sachets (Reckitt Benckiser Healthcare (UK) Ltd) |
| 32035 | Paracetamol 240mg with phenylephrine 2.5mg sachet |
| 32093 | Paracetamol 250mg/5ml oral suspension (Rosemont Pharmaceuticals Ltd) |
| 32163 | Paracetamol 500mg tablets (Vantage) |
| 32178 | Paracetamol 133mg with aspirin 267mg & caffeine 40mg effervescent tablet |
| 32184 | Beechams Cold & Flu capsules (GlaxoSmithKline Consumer Healthcare) |
| 32554 | Placidex 120mg/5ml Oral solution (E C De Witt) |
| 32609 | Real lemon cold 750mg+10mg+60mg Powder (Sterwin Medicines) |
| 32626 | Paracetamol 150mg / Sodium salicylate 100mg tablets |
| 32672 | DISPROL PARACETAMOL SF |
| 32681 | Covonia Cold & Flu Formula oral solution (Thornton & Ross Ltd) |
| 32766 | Paracetamol 500mg/50ml solution for infusion vials |
| 32839 | Paracetamol 500mg tablets (A A H Pharmaceuticals Ltd) |
| 32942 | Paracetamol 1000mg with diphenhydramine 25mg & phenylephrine 12mg powder |
| 32963 | Lemsip lemcap Capsule (Reckitt Benckiser Healthcare (UK) Ltd) |
| 32970 | Paracets 500mg capsules (Sussex Pharmaceutical Ltd) |
| 32993 | Children's Lemsip Cold & Flu Blackcurrant oral powder sachets (Reckitt Benckiser Healthcare (UK) Ltd) |
| 33042 | Lemsip children's six plus cold and flu relief 240mg+2.5mg Sachets (Reckitt Benckiser Healthcare (UK) Ltd) |
| 33060 | Paracetamol Plus tablets (Zentiva) |
| 33104 | Paraclear 500mg Soluble tablet (Roche Consumer Health) |
| 33230 | Promethazine hydrochloride 1.5mg with paracetamol 120mg/5ml oral solution |
| 33386 | Paracetamol 120mg/5ml oral suspension paediatric (A A H Pharmaceuticals Ltd) |
| 33614 | Obimol 500mg Tablet (Ayrton Saunders Ltd) |
| 33642 | Vicks Medinite syrup (Procter & Gamble (Health & Beauty Care) Ltd) |
| 33666 | Paracetamol 500mg tablets (Actavis UK Ltd) |
| 33687 | Paracetamol 250mg/5ml oral suspension sugar free (A A H Pharmaceuticals Ltd) |
| 33710 | Paracetamol 500mg caplets (A A H Pharmaceuticals Ltd) |
| 33826 | Paracetamol 500mg Tablet (Teva UK Ltd) |
| 33838 | Doans extra strength Tablet (Novartis Consumer Health UK Ltd) |
| 34164 | Paracetamol 500mg soluble tablets (Fannin UK Ltd) |
| 34209 | Paracetamol 500mg/5ml Oral suspension sugar free (Rosemont Pharmaceuticals Ltd) |
| 34235 | Paracetamol 120mg Suppository (Penn Pharmaceuticals Ltd) |
| 34266 | Tixymol 120mg/5ml Oral suspension (Novartis Consumer Health UK Ltd) |
| 34305 | Paracetamol 500mg Tablet (M & A Pharmachem Ltd) |
| 34350 | Paracetamol 500mg Tablet (Celltech Pharma Europe Ltd) |
| 34396 | Paracetamol 500mg Tablet (Family Health) |
| 34409 | Paracetamol 500mg Tablet (Co-operative) |
| 34500 | Paracetamol 500mg tablets (Kent Pharmaceuticals Ltd) |
| 34669 | Paracetamol 500mg capsules (Zentiva) |
| 34678 | Paracetamol 120mg/5ml oral solution paediatric (Thornton & Ross Ltd) |
| 34700 | Paracetamol 250mg/5ml oral suspension sugar free (Pinewood Healthcare) |
| 34718 | Paracetamol 120mg/5ml oral suspension sugar free |
| 34761 | Paracetamol 120mg/5ml Oral solution (Teva UK Ltd) |
| 34858 | Paracetamol Capsule (Co-operative) |
| 34861 | Paracetamol 120mg suppositories (Martindale Pharmaceuticals Ltd) |
| 34954 | Paracetamol 500mg tablets (Aspar Pharmaceuticals Ltd) |
| 35679 | Paracetamol 500mg Tablet (Nucare Plc) |
| 35800 | Mandanol Infant paracetamol 120mg/5ml oral suspension (M & A Pharmachem Ltd) |
| 35885 | Lemsip Max Cold & Flu Blackcurrant powder sachets (Reckitt Benckiser Healthcare (UK) Ltd) |
| 35888 | Lemsip Max Cold & Flu Direct Blackcurrant oral powder sachets (Reckitt Benckiser Healthcare (UK) Ltd) |
| 35889 | Lemsip Max Cold & Flu Breathe Easy oral powder sachets (Reckitt Benckiser Healthcare (UK) Ltd) |
| 35967 | Paramed Extra Power Pain Control tablets (Galpharm International Ltd) |
| 36598 | Beechams Cold and Flu Hot Blackcurrant oral powder 6g sachets (GlaxoSmithKline Consumer Healthcare) |
| 36614 | Paracetamol 1g / Phenylephrine 12.2mg oral powder sachets |
| 36720 | Resolve Extra effervescent granules sachets (G.R. Lane Health Products Ltd) |
| 36754 | Paracetamol 500mg soluble tablets (A A H Pharmaceuticals Ltd) |
| 36929 | Calpol Night oral solution (McNeil Products Ltd) |
| 36945 | Mandanol 6+ paracetamol 250mg/5ml oral suspension (M & A Pharmachem Ltd) |
| 37411 | Paldesic paracetamol 250mg/5ml oral suspension (Rosemont Pharmaceuticals Ltd) |
| 37446 | Benylin Cold & Flu Max Strength capsules (McNeil Products Ltd) |
| 37571 | Beechams Active Cold Relief caplets (GlaxoSmithKline Consumer Healthcare) |
| 37607 | Lemsip Max All in One Lemon oral powder sachets (Reckitt Benckiser Healthcare (UK) Ltd) |
| 38032 | Paracetamol 500mg capsules (Teva UK Ltd) |
| 38122 | Paracetamol 330mg with caffeine 30mg tablet |
| 38299 | CalCold Six Plus oral solution (McNeil Products Ltd) |
| 38323 | Paracetamol 120mg/5ml Oral solution sugar free (A A H Pharmaceuticals Ltd) |
| 38943 | Medinol Paediatric paracetamol 120mg/5ml oral suspension (SSL International Plc) |
| 38984 | Paracetamol 500mg capsules (Actavis UK Ltd) |
| 39291 | Benylin Cold and Flu Max Strength Non-Drowsy oral powder sachets (McNeil Products Ltd) |
| 39333 | Paracetamol 500mg Tablet (Aspar Pharmaceuticals Ltd) |
| 39481 | Panadol Advance 500mg tablets (GlaxoSmithKline Consumer Healthcare) |
| 39629 | Sudafed Non-Drowsy Dual Relief capsules (McNeil Products Ltd) |
| 39647 | Medinol Over 6 paracetamol 250mg/5ml oral suspension (SSL International Plc) |
| 39656 | Medinol Under 6 paracetamol 120mg/5ml oral suspension (SSL International Plc) |
| 39739 | Sudafed non-drowsy congestion & headache Capsule (McNeil Products Ltd) |
| 39893 | Lemsip Max Sinus oral powder sachets (Reckitt Benckiser Healthcare (UK) Ltd) |
| 39934 | Paracetamol 500mg caplets (Almus Pharmaceuticals Ltd) |
| 39940 | Panadol ActiFast Soluble tablets (GlaxoSmithKline Consumer Healthcare) |
| 40107 | Paracetamol 500mg soluble tablets (Kent Pharmaceuticals Ltd) |
| 40158 | Paracetamol 500mg Capsule (A A H Pharmaceuticals Ltd) |
| 40624 | Benylin Four Flu tablets (McNeil Products Ltd) |
| 40880 | Vicks sinex decongestant Capsule (Procter & Gamble (Health & Beauty Care) Ltd) |
| 40948 | Paracetamol with phenylephrine & paracetamol with phenylephrine & caffeine capsule |
| 41142 | Sudafed non-drowsy sinus & pain Capsule (McNeil Products Ltd) |
| 41409 | Paracetamol 500mg suppositories (Martindale Pharmaceuticals Ltd) |
| 41414 | Paracetamol 120mg/5ml Oral suspension (Co-Pharma Ltd) |
| 41435 | Lemsip Max All in One Breathe Easy oral powder sachets (Reckitt Benckiser Healthcare (UK) Ltd) |
| 41680 | Paracetamol 120mg/5ml Oral suspension sugar free (IVAX Pharmaceuticals UK Ltd) |
| 42101 | Perfalgan 1g/100ml solution for infusion vials (Bristol-Myers Squibb Pharmaceuticals Ltd) |
| 42125 | Paracetamol 120mg Suppository (Distriphar (UK)) |
| 42201 | Paracetamol 500mg capsules (A A H Pharmaceuticals Ltd) |
| 42345 | Paracets Powder (Sussex Pharmaceutical Ltd) |
| 42371 | Paracetamol 1g tablets |
| 42514 | Paracetamol oral liquid |
| 42834 | Panadol OA 1000mg tablets (GlaxoSmithKline Consumer Healthcare) |
| 42915 | Paracetamol 120mg/5ml Oral solution (William Ransom) |
| 43028 | Paracetamol 650mg oral powder sachets |
| 43199 | Paracetamol 250mg/5ml oral suspension sugar free (Vantage) |
| 43204 | Paracetamol 500mg with phenylephrine 6.1mg & caffeine 25mg tablet |
| 43233 | Paracetamol 500mg capsules (Kent Pharmaceuticals Ltd) |
| 43252 | Paracetamol 500mg capsules (Aspar Pharmaceuticals Ltd) |
| 43447 | Paracetamol 120mg/5ml oral suspension paediatric sugar free (Thornton & Ross Ltd) |
| 43479 | Paracetamol 500mg caplets (Actavis UK Ltd) |
| 43554 | Paracetamol 120mg/5ml Oral solution (Celltech Pharma Europe Ltd) |
| 44258 | Dichloralphenazone with paracetamol oral solution |
| 44369 | Beechams Flu-Plus Hot Berry Fruits oral powder sachets (GlaxoSmithKline Consumer Healthcare) |
| 44537 | Sudafed Blocked Nose & Headache capsules (McNeil Products Ltd) |
| 44622 | Beechams Flu-Plus Hot Lemon oral powder sachets (GlaxoSmithKline Consumer Healthcare) |
| 44632 | Sudafed Sinus Max Strength capsules (McNeil Products Ltd) |
| 44977 | Paracetamol with pseudoephedrine & pholcodine 500mg with 30mg with 5mg/15ml oral liquid |
| 44984 | Sudafed decongestant & pain Tablet (McNeil Products Ltd) |
| 45259 | Paracetamol 500mg Tablet (OBG Pharmaceuticals Ltd) |
| 45298 | Paracetamol 500mg tablets (Galpharm International Ltd) |
| 45348 | Paracetamol 120mg/5ml Oral suspension (Nucare Plc) |
| 45349 | Paracetamol 240mg suppositories (Martindale Pharmaceuticals Ltd) |
| 45469 | Benylin Four Flu liquid (McNeil Products Ltd) |
| 45497 | Lemsip Max All In One Liquid (Reckitt Benckiser Healthcare (UK) Ltd) |
| 45645 | Medinol for children 120mg/5ml Oral suspension (SSL International Plc) |
| 45649 | Calpol infant 120mg/5ml Oral suspension (McNeil Products Ltd) |
| 46498 | Paracetamol 1000mg with phenylephrine 12mg & ascorbic acid 100mg sachet |
| 46544 | Paracetamol 500mg capsules (Wockhardt UK Ltd) |
| 46638 | Paracetamol 500mg / Ibuprofen 200mg tablets |
| 46763 | Paracetamol 500mg/5ml oral suspension |
| 46830 | Beechams Max Strength All In One capsules (GlaxoSmithKline Consumer Healthcare) |
| 46846 | Paracetamol 500mg/5ml oral solution |
| 46876 | Contac Non Drowsy Dual Relief tablets (Omega Pharma Ltd) |
| 46895 | Sudafed Mucus Relief Triple Action Cold & Flu tablets (McNeil Products Ltd) |
| 46904 | Nuromol 200mg/500mg tablets (Reckitt Benckiser Healthcare (UK) Ltd) |
| 47026 | Lemsip Max All in One Cold & Flu capsules (Reckitt Benckiser Healthcare (UK) Ltd) |
| 47116 | Paracetamol 500mg soluble tablets (Almus Pharmaceuticals Ltd) |
| 47211 | Paracetamol 500mg capsules (Focus Pharmaceuticals Ltd) |
| 47834 | Paracetamol 500mg soluble tablets (Actavis UK Ltd) |
| 47920 | Flu Relief with Decongestant capsules (Galpharm International Ltd) |
| 48129 | Paracetamol 120mg/5ml oral suspension paediatric sugar free (Vantage) |
| 48157 | Paracetamol 120mg/5ml oral suspension paediatric sugar free (A A H Pharmaceuticals Ltd) |
| 48166 | Paracetamol 500mg / Phenylephrine 12.18mg capsules |
| 48178 | Panadol Extra Advance 500mg/65mg tablets (GlaxoSmithKline Consumer Healthcare) |
| 48295 | Paracetamol 250mg/5ml oral suspension sugar free |
| 48301 | Paracetamol 120mg/5ml oral suspension paediatric |
| 48330 | Paracetamol 250mg/5ml oral suspension 5ml sachets sugar free |
| 48354 | Paracetamol 120mg/5ml oral suspension paediatric sugar free |
| 48444 | Calpol Infant 120mg/5ml oral suspension sugar free (McNeil Products Ltd) |
| 48526 | Paracetamol 1g suppositories |
| 48535 | Paracetamol 500mg caplets (Rusco Ltd) |
| 48561 | Calpol Six Plus 250mg/5ml oral suspension sugar free (McNeil Products Ltd) |
| 48597 | Paracetamol 500mg caplets (Alliance Healthcare (Distribution) Ltd) |
| 48622 | Calpol Infant 120mg/5ml oral suspension 5ml sachets sugar free (McNeil Products Ltd) |
| 48816 | Disprol Paracetamol 120mg/5ml oral suspension (Reckitt Benckiser Healthcare (UK) Ltd) |
| 49096 | Paracetamol 500mg caplets (Lloyds Pharmacy Ltd) |
| 49105 | Paracetamol 500mg caplets (Teva UK Ltd) |
| 49219 | Calpol Infant 120mg/5ml oral suspension (McNeil Products Ltd) |
| 49314 | Paracetamol 120mg/5ml oral suspension 5ml sachets sugar free |
| 49417 | Paracetamol 500mg caplets (Kent Pharmaceuticals Ltd) |
| 49524 | Paracetamol 250mg/5ml oral suspension sugar free (Alliance Healthcare (Distribution) Ltd) |
| 49575 | Paracetamol 500mg caplets (Vantage) |
| 49849 | Paracetamol 500mg caplets (Phoenix Healthcare Distribution Ltd) |
| 49954 | Generic Cold and Flu Relief tablets |
| 50009 | Paracetamol 120mg/5ml oral suspension 5ml sachets |
| 50166 | Generic Anadin Extra tablets |
| 50232 | Calpol Six Plus 250mg/5ml oral suspension 5ml sachets sugar free (McNeil Products Ltd) |
| 50482 | Paracetamol 500mg tablets (DE Pharmaceuticals) |
| 50504 | Numark Paracetamol 500mg capsules (Numark Ltd) |
| 50881 | Paracetamol Extra Strength Pain Relief tablets (Galpharm International Ltd) |
| 50984 | Paracetamol 250mg/5ml oral solution |
| 51595 | Calpol Infant 120mg/5ml oral suspension 5ml sachets (McNeil Products Ltd) |
| 52455 | Medinol For Children 120mg/5ml oral suspension (SSL International Plc) |
| 52456 | Cold and Flu Relief tablets (The Boots Company Plc) |
| 52948 | Paracetamol 1g/5ml oral suspension |
| 53952 | Paracetamol 500mg capsules (Lloyds Pharmacy Ltd) |
| 53961 | Perfalgan 500mg/50ml solution for infusion vials (Bristol-Myers Squibb Pharmaceuticals Ltd) |
| 53984 | Paracetamol 500mg/5ml oral suspension sugar free |
| 54179 | Paracetamol 500mg tablets (The Boots Company Plc) |
| 54353 | Generic Anadin Extra soluble tablets sugar free |
| 54737 | Generic Covonia Cold & Flu Formula oral solution sugar free |
| 54853 | Paracetamol 120mg/5ml oral solution paediatric sugar free (A A H Pharmaceuticals Ltd) |
| 55129 | Paracetamol 500mg caplets (Waymade Healthcare Plc) |
| 55290 | Paracetamol 300mg with phenylephrine 5mg & caffeine 25mg capsule |
| 55418 | Paracetamol 500mg Tablet (Almus Pharmaceuticals Ltd) |
| 55817 | Lloydspharmacy Paracetamol 120mg/5ml oral suspension sugar free (Lloyds Pharmacy Ltd) |
| 55951 | Generic Beechams All-In-One tablets |
| 56046 | Paracetamol 500mg capsules (Waymade Healthcare Plc) |
| 56566 | Paracetamol 500mg caplets (J M McGill Ltd) |
| 56877 | Coldrex Tablet (GlaxoSmithKline UK Ltd) |
| 56945 | Paracetamol 500mg soluble tablets (Waymade Healthcare Plc) |
| 57092 | Night Nurse Hot Lemon Menthol oral powder sachets (GlaxoSmithKline Consumer Healthcare) |
| 57425 | Generic Beechams Flu-Plus Hot Lemon oral powder sachets |
| 57433 | Boots Paracetamol Extra tablets (The Boots Company Plc) |
| 57650 | Paracetamol 500mg capsules (Sigma Pharmaceuticals Plc) |
| 58214 | Lemsip Cough Max for Mucus Cough & Cold 500mg/100mg/6.1mg capsules (Reckitt Benckiser Healthcare (UK) Ltd) |
| 58526 | Paracetamol 500mg caplets (AM Distributions (Yorkshire) Ltd) |
| 58582 | Paracetamol 500mg tablets (Teva UK Ltd) |
| 58631 | Paracetamol 650mg / Phenylephrine 10mg oral powder sachets |
| 58743 | Paracetamol 500mg tablets (Accord Healthcare Ltd) |
| 58857 | Lemsip Max Blackcurrant Flavour tablets (Reckitt Benckiser Healthcare (UK) Ltd) |
| 59599 | Paracetamol 500mg tablets (Wockhardt UK Ltd) |
| 59773 | Paracetamol 500mg/5ml oral solution sugar free |
| 59801 | Generic Night Nurse oral solution |
| 59823 | Paracetamol 1g / Pseudoephedrine 60mg oral powder sachets |
| 60378 | Flu Strength Hot Lemon Powders 1g oral powder sachets (Bell,Sons & Co (Druggists) Ltd) |
| 60442 | Paracetamol 500mg tablets (Alliance Healthcare (Distribution) Ltd) |
| 60812 | Paracetamol 500mg caplets (Ethigen Ltd) |
| 61124 | Paracetamol 500mg caplets (Bristol Laboratories Ltd) |
| 61406 | Paracetamol 80mg suppositories |
| 61485 | Paracetamol 120mg/5ml oral solution paediatric sugar free (Alliance Healthcare (Distribution) Ltd) |
| 61522 | Paracetamol 500mg caplets (Numark Ltd) |
| 61851 | Boots Max Strength sinus relief capsules (The Boots Company Plc) |
| 61963 | Paracetamol 500mg caplets (Ennogen Healthcare Ltd) |
| 62088 | Paracetamol 500mg caplets (Icarus Pharmaceuticals Ltd) |
| 62257 | Sudafed Congestion & Headache Relief Max Strength capsules (McNeil Products Ltd) |
| 62305 | Lemsip Cough Max for Mucus Cough & Cold 1000mg/200mg/12.2mg oral powder sachets (Reckitt Benckiser Healthcare (UK) Ltd) |
| 62323 | Calpol Infant Sugar Free Colour Free 120mg/5ml oral suspension (McNeil Products Ltd) |
| 62372 | Benylin Chesty Cough & Cold tablets (McNeil Products Ltd) |
| 62491 | Generic Lemsip Max Cold & Flu capsules |
| 62743 | Boots Paracetamol 500mg capsules (The Boots Company Plc) |
| 63543 | Paracetamol 500mg tablets (Sigma Pharmaceuticals Plc) |
| 63908 | Generic Beechams Flu-Plus caplets |
| 63943 | Paracetamol 500mg caplets (Crescent Pharma Ltd) |
| 63962 | Paracetamol 500mg soluble tablets (Teva UK Ltd) |
| 64073 | Paracetamol 120mg/5ml oral suspension paediatric sugar free (Alliance Healthcare (Distribution) Ltd) |
| 64125 | Paramed Max Strength Cold and Flu Relief oral powder sachets (Galpharm International Ltd) |
| 64154 | Paracetamol 120mg/5ml oral solution paediatric sugar free (Waymade Healthcare Plc) |
| 64932 | Generic Night Nurse capsules |
| 65080 | Paracetamol 250mg/5ml oral suspension sugar free (DE Pharmaceuticals) |
| 65107 | Paracetamol 500mg capsules (Bristol Laboratories Ltd) |
| 65239 | Paracetamol 500mg caplets (Mawdsley-Brooks & Company Ltd) |
| 65299 | Paracetamol 500mg caplets (Sigma Pharmaceuticals Plc) |
| 65330 | Paracetamol 500mg capsules (DE Pharmaceuticals) |
| 65650 | Dolvan tablets (Norma Chemicals Ltd) |
| 65683 | Paracetamol 120mg/5ml oral suspension paediatric sugar free (Pinewood Healthcare) |
| 66097 | Paracetamol 500mg capsules (Almus Pharmaceuticals Ltd) |
| 66481 | Boots Paracetamol Extra soluble tablets (The Boots Company Plc) |
| 66664 | Medised plain 120mg/5ml Oral suspension (SSL International Plc) |
| 67086 | Paracetamol 500mg capsules (Galpharm International Ltd) |
| 67370 | Beechams flu plus 1000mg+10mg+40mg Powder (GlaxoSmithKline Consumer Healthcare) |
| 67784 | Paracetamol 500mg capsules (Almus Pharmaceuticals Ltd) |
| 67887 | Paracetamol 120mg soluble tablets sugar free (Ennogen Healthcare Ltd) |
| 68070 | Paracetamol 500mg tablets (Zanza Laboratories Ltd) |
| 68434 | Paracetamol 250mg/5ml Liquid (Co-Pharma Ltd) |
| 68714 | Generic Benylin Four Flu tablets |
| 68738 | Junior Parapaed 120mg/5ml oral suspension sugar free colour free (Pinewood Healthcare) |
| 68857 | Generic Day Nurse capsules |
| 70018 | Boots Paracetamol 500mg caplets (The Boots Company Plc) |
| 70326 | Paravict 500mg tablets (Ecogen Europe Ltd) |
| 70355 | Paracetamol 1g/100ml solution for infusion bottles |
| 71178 | Paracetamol 1g / Phenylephrine 12.2mg oral powder sachets sugar free |
| 71270 | Paracetamol 500mg effervescent tablets sugar free |
| 71545 | Lemsip Cough Max for Chesty Cough & Cold oral powder sachets (Reckitt Benckiser Healthcare (UK) Ltd) |
| 72131 | Paracetamol 500mg capsules (Mawdsley-Brooks & Company Ltd) |
| 72288 | Paracetamol 500mg caplets (Accord Healthcare Ltd) |
| 570 | Dynastat 40mg Powder for solution for injection (Pharmacia Ltd) |
| 19975 | Parecoxib 40mg powder for injection |
| 50080 | Dynastat 40mg powder and solvent for solution for injection vials (Pfizer Ltd) |
| 51306 | Parecoxib 40mg powder for solution for injection vials |
| 54760 | Parecoxib 40mg powder and solvent for solution for injection vials |
| 58644 | Dynastat 40mg powder for solution for injection vials (Pfizer Ltd) |
| 790 | Pregabalin 25mg capsules |
| 819 | Pregabalin 75mg capsules |
| 6584 | Lyrica 75mg capsules (Pfizer Ltd) |
| 6631 | Pregabalin 150mg capsules |
| 6936 | Pregabalin 50mg capsules |
| 6949 | Pregabalin 200mg capsules |
| 6999 | Pregabalin 100mg capsules |
| 7005 | Pregabalin 300mg capsules |
| 7208 | Lyrica 100mg capsules (Pfizer Ltd) |
| 7209 | Lyrica 50mg capsules (Pfizer Ltd) |
| 7394 | Lyrica 200mg capsules (Pfizer Ltd) |
| 10189 | Lyrica 300mg capsules (Pfizer Ltd) |
| 16509 | Lyrica 150mg capsules (Pfizer Ltd) |
| 16542 | Lyrica 25mg capsules (Pfizer Ltd) |
| 37801 | Pregabalin 225mg capsules |
| 38293 | Lyrica 225mg capsules (Pfizer Ltd) |
| 48253 | Lyrica 150mg capsules (Lexon (UK) Ltd) |
| 51227 | Pregabalin 20mg/ml oral solution sugar free |
| 51924 | Lyrica 20mg/ml oral solution (Pfizer Ltd) |
| 52547 | Lyrica 50mg capsules (Waymade Healthcare Plc) |
| 55972 | Pregabalin 150mg/5ml oral solution |
| 60543 | Pregabalin 75mg/5ml oral solution |
| 63069 | Rewisca 75mg capsules (Consilient Health Ltd) |
| 63088 | Rewisca 50mg capsules (Consilient Health Ltd) |
| 63089 | Rewisca 300mg capsules (Consilient Health Ltd) |
| 63090 | Rewisca 150mg capsules (Consilient Health Ltd) |
| 63091 | Rewisca 100mg capsules (Consilient Health Ltd) |
| 63174 | Rewisca 200mg capsules (Consilient Health Ltd) |
| 63300 | Rewisca 25mg capsules (Consilient Health Ltd) |
| 63317 | Rewisca 225mg capsules (Consilient Health Ltd) |
| 63877 | Pregabalin 50mg capsules (A A H Pharmaceuticals Ltd) |
| 63964 | Alzain 25mg capsules (Dr Reddy's Laboratories (UK) Ltd) |
| 63965 | Alzain 100mg capsules (Dr Reddy's Laboratories (UK) Ltd) |
| 64005 | Pregabalin 100mg capsules (A A H Pharmaceuticals Ltd) |
| 64037 | Alzain 300mg capsules (Dr Reddy's Laboratories (UK) Ltd) |
| 64038 | Alzain 225mg capsules (Dr Reddy's Laboratories (UK) Ltd) |
| 64039 | Alzain 150mg capsules (Dr Reddy's Laboratories (UK) Ltd) |
| 64040 | Alzain 75mg capsules (Dr Reddy's Laboratories (UK) Ltd) |
| 64041 | Alzain 50mg capsules (Dr Reddy's Laboratories (UK) Ltd) |
| 64042 | Alzain 200mg capsules (Dr Reddy's Laboratories (UK) Ltd) |
| 64285 | Pregabalin 150mg/5ml oral suspension |
| 64497 | Lyrica 75mg capsules (Waymade Healthcare Plc) |
| 64568 | Lyrica 150mg capsules (Sigma Pharmaceuticals Plc) |
| 65069 | Pregabalin 75mg capsules (Teva UK Ltd) |
| 65073 | Pregabalin 25mg capsules (Teva UK Ltd) |
| 65218 | Lecaent 50mg capsules (Actavis UK Ltd) |
| 65606 | Pregabalin 50mg capsules (Accord Healthcare Ltd) |
| 65787 | Pregabalin 50mg capsules (Sandoz Ltd) |
| 65863 | Lyrica 75mg capsules (Stephar (U.K.) Ltd) |
| 66509 | Pregabalin 75mg capsules (Zentiva) |
| 66941 | Lecaent 75mg capsules (Actavis UK Ltd) |
| 67053 | Pregabalin 75mg capsules (DE Pharmaceuticals) |
| 67184 | Lyrica 50mg capsules (Mawdsley-Brooks & Company Ltd) |
| 67384 | Pregabalin 75mg/5ml oral suspension |
| 67440 | Pregabalin 300mg capsules (A A H Pharmaceuticals Ltd) |
| 68014 | Lyrica 75mg capsules (Lexon (UK) Ltd) |
| 68441 | Pregabalin 25mg/5ml oral solution |
| 69034 | Pregabalin 300mg capsules (Mylan) |
| 69125 | Axalid 100mg capsules (Kent Pharmaceuticals Ltd) |
| 69296 | Axalid 25mg capsules (Kent Pharmaceuticals Ltd) |
| 69418 | Axalid 300mg capsules (Kent Pharmaceuticals Ltd) |
| 69497 | Axalid 225mg capsules (Kent Pharmaceuticals Ltd) |
| 69498 | Axalid 75mg capsules (Kent Pharmaceuticals Ltd) |
| 69499 | Axalid 150mg capsules (Kent Pharmaceuticals Ltd) |
| 69501 | Lyrica 75mg capsules (DE Pharmaceuticals) |
| 69554 | Axalid 50mg capsules (Kent Pharmaceuticals Ltd) |
| 69781 | Lyrica 25mg capsules (Lexon (UK) Ltd) |
| 69799 | Axalid 200mg capsules (Kent Pharmaceuticals Ltd) |
| 69877 | Lecaent 300mg capsules (Actavis UK Ltd) |
| 69987 | Pregabalin 75mg capsules (A A H Pharmaceuticals Ltd) |
| 70064 | Pregabalin 25mg capsules (Accord Healthcare Ltd) |
| 70229 | Pregabalin 75mg capsules (Mylan) |
| 70478 | Pregabalin 150mg capsules (Teva UK Ltd) |
| 70544 | Pregabalin 150mg capsules (Alliance Healthcare (Distribution) Ltd) |
| 70545 | Pregabalin 25mg capsules (Alliance Healthcare (Distribution) Ltd) |
| 70546 | Pregabalin 50mg capsules (Alliance Healthcare (Distribution) Ltd) |
| 70648 | Pregabalin 300mg capsules (Teva UK Ltd) |
| 70729 | Pregabalin 200mg capsules (Alliance Healthcare (Distribution) Ltd) |
| 70730 | Pregabalin 100mg capsules (Alliance Healthcare (Distribution) Ltd) |
| 70731 | Pregabalin 75mg capsules (Alliance Healthcare (Distribution) Ltd) |
| 70735 | Pregabalin 300mg capsules (Alliance Healthcare (Distribution) Ltd) |
| 71221 | Lecaent 200mg capsules (Actavis UK Ltd) |
| 71313 | Pregabalin 50mg capsules (Zentiva) |
| 71461 | Pregabalin 50mg/5ml oral suspension |
| 71533 | Pregabalin 50mg capsules (Mylan) |
| 71659 | Lecaent 25mg capsules (Actavis UK Ltd) |
| 72068 | Lecaent 150mg capsules (Actavis UK Ltd) |
| 518 | Rofecoxib 12.5mg tablets |
| 538 | Vioxx 12.5mg tablets (Merck Sharp & Dohme Ltd) |
| 613 | Vioxx 12.5mg/5ml oral suspension (Merck Sharp & Dohme Ltd) |
| 637 | Rofecoxib 25mg/5ml oral suspension sugar free |
| 640 | Rofecoxib 12.5mg/5ml oral suspension sugar free |
| 666 | Vioxx 25mg tablets (Merck Sharp & Dohme Ltd) |
| 706 | Rofecoxib 25mg tablets |
| 5695 | VioxxAcute 50mg tablets (Merck Sharp & Dohme Ltd) |
| 5739 | Vioxx 25mg/5ml oral suspension (Merck Sharp & Dohme Ltd) |
| 5841 | Rofecoxib 50mg tablets |
| 6460 | VioxxAcute 25mg tablets (Merck Sharp & Dohme Ltd) |
| 30090 | ROFECOXIB |
| 32362 | ROFECOXIB |
| 36669 | ROFECOXIB |
| 53145 | Vioxx 12.5mg tablets (Dowelhurst Ltd) |
| 67786 | Vioxx 25mg tablets (Dowelhurst Ltd) |
| 723 | Valdecoxib 10mg tablets |
| 6663 | Valdecoxib 20mg tablets |
| 9899 | Bextra 10mg tablets (Pfizer Ltd) |
| 9912 | Bextra 20mg tablets (Pfizer Ltd) |
| 9978 | Bextra 40mg tablets (Pfizer Ltd) |
| 18066 | Valdecoxib 40mg tablets |

ANNEX 8. Algorithms of CRAB investigation and confirmation (laboratory records)

| **ENTYPE** | **Definition** |
| --- | --- |
| **Anaemia – investigation** | |
| 173 – Description Haemoglobin | Any records of ENTYPE = 173 |
| **Hypercalcemia - investigation** | |
| 159 – Description Calcium | Any records of ENTYPE = 159 |
| **Renal impairment - investigation** | |
| 165 – Description Serum Creatine | Any records of ENTYPE = 165 |
| **Anaemia – confirmation** | |
| 173 – Description Haemoglobin | Any records of ENTYPE = 173  Operator = “=”  Units = ‘g/dL’ or ‘g/L’  Test result < 110g/L (11g/dL) for males, < 100g/L (10g/dL) for females |
| **Hypercalcemia - confirmation** | |
| 159 – Description Calcium | Any records of ENTYPE = 159  Operator = “=”  Units = ‘mmol/L’ or ‘ ’  Test result > 2.75 mmol/L (11 mg/dL)  Maximum acceptable test result for valid test 5.0 mmol/L |
| **Renal impairment - confirmation** | |
| 165 – Description Serum Creatine | Any records of ENTYPE = 165  Operator = “=”  Units = ‘umol/L’or ‘ ’  Test result > 177 umol/L (2 mg/dL)  Maximum acceptable test result for valid test 600 umol/L |

ANNEX 9. Definition of CRAB investigation and confirmation (clinical records)

| **READ code** | **Code term** |
| --- | --- |
| **Anaemia - investigation** | |
| ZV78100 | [V]Screening for other or unspecified deficiency anaemia |
| 688Z.00 | Anaemia/blood screen NOS |
| 688..00 | Anaemia/blood screening |
| 428..11 | Mean cell haemoglobin |
| 428..00 | Mean corpusc. haemoglobin(MCH) |
| 423Z.00 | Haemoglobin estimation NOS |
| 423A.00 | Haemoglobin very high |
| 4239.00 | Haemoglobin high |
| 4238.00 | Haemoglobin borderline high |
| 4237.00 | Haemoglobin normal |
| 4233.00 | Haemoglobin - sample sent |
| 4232.00 | Haemoglobin requested |
| 423..00 | Haemoglobin estimation |
| 6884.00 | Anaemia screen |
| 688..11 | Anaemia screen |
| 423..11 | Hb estimation |
| 429Z.00 | MCHC - NOS |
| 4295.00 | MCHC - borderline raised |
| 4294.00 | MCHC - raised |
| 4293.00 | MCHC - raised |
| 4291.00 | MCHC - normal |
| 429..00 | Mean corpusc. Hb. conc. (MCHC) |
| 428Z.00 | MCH - NOS |
| 4281.00 | MCH - normal |
| **Hypercalcaemia - investigation** | |
| 44Z2.00 | Bone profile |
| 44ZR.00 | Calcium profile |
| 7L17200 | Blood withdrawal for testing |
| 44h..00 | Blood electrolyte levels |
| 44i..00 | Plasma electrolyte levels |
| 4Q44.00 | Electrolytes level |
| 44I..00 | Serum electrolytes |
| 44IZ.00 | Serum electrolytes NOS |
| C36zz00 | Electrolyte imbalance NOS |
| C36..11 | Electrolyte disorders |
| 44x..00 | Biochemistry battery tests |
| 4Q...00 | Generic biochemistry levels |
| 4QC0.00 | Other generic biochemistry level |
| ZLEA211 | Discharge from clinical biochemistry service |
| 44h4.00 | Blood calcium level |
| 44h7.00 | Plasma calcium level |
| 44h9.00 | Plasma corrected calcium level |
| 44hD.00 | Blood ionised calcium level |
| 44I8.00 | Serum calcium |
| 44I8000 | Normal serum calcium level |
| 44IC.00 | Corrected serum calcium level |
| 44IC.11 | Adjusted serum calcium level |
| 44ID.00 | Serum ionised calcium level |
| 44IE.00 | Serum ionized calcium (pH 7.4) level |
| 46P4.00 | Urine calcium |
| 4Q72.00 | Calcium level |
| 4Q72100 | Calcium adjusted level |
| C354900 | Calcium deficiency |
| C354z00 | Disorder of calcium metabolism NOS |
| **Renal impairment - investigation** | |
| 4Q40.00 | Creatinine level |
| 4Q2Q.00 | 11 oxo-pregnanetriol:creatinine ratio |
| 4Q2N.00 | 11 betahydroxy androsterone:creatinine ratio |
| 4Q2M.00 | 11 betahydroxy aetiocholanolone:creatinine ratio |
| 4Q2H.00 | Cortisol metabolite:creatinine ratio |
| 4I37.11 | Creatinine in sample |
| 4I37.00 | Fluid sample creatinine |
| 46TD.00 | Urine microalbumin:creatinine ratio |
| 46TC.00 | Urine albumin:creatinine ratio |
| 46PD.00 | Urine nickel:creatinine ratio |
| 46NB.00 | Retinol binding protein creatinine ratio |
| 46N7.00 | Urine protein/creatinine index |
| 46MD.00 | 24 hour urine creatinine output |
| 46M7.00 | Urine creatinine |
| 46M7.00 | Urine creatinine |
| 46LN.00 | Urine normetadrenaline/creatinine ratio |
| 46LM.00 | Urine metadrenaline/creatinine ratio |
| 46LL.00 | Urine cortisol/creatinine ratio |
| 46LK.00 | Urine homovanillic acid/creatinine ratio |
| 46g4.00 | Urine calcium/creatinine ratio |
| 46g3.00 | Urine noradrenaline/creatinine ratio |
| 46g2.00 | Urine oxalate/creatinine ratio |
| 46g0.00 | Urine 3-methoxytyramine/creatinine ratio |
| 46d..00 | Urine deoxypyridinoline/creatinine ratio |
| 451A.00 | Estimated creatinine clearance |
| 4517.00 | Creatinine clearance test |
| 4513000 | Creatinine clearance-glom filt normal |
| 4513.11 | Creatinine clearance |
| 4513.00 | Creatinine clearance-glom filt |
| 44lzL00 | Methyl malonic acid/creatinine ratio |
| 44lzH00 | Proline/creatinine ratio |
| 44lz400 | Cystine/creatinine ratio |
| 44lz300 | Catecholamine/creatinine ratio |
| 44lz100 | Homocysteine/creatinine ratio |
| 44lZ.00 | 5HIAA/creatinine ratio |
| 44lY.00 | Urine uroporphyrin/creatinine ratio |
| 44lX.00 | Urine coproporphyrin/creatinine ratio |
| 44lx.00 | Glutamate/creatinine ratio |
| 44lt.00 | Urea/creatinine ratio |
| 44ls.00 | Lead/creatinine ratio |
| 44lq.00 | Aspartate/creatinine ratio |
| 44lo.00 | Alanine/creatinine ratio |
| 44lN.00 | Deoxypyridinoline/creatinine ratio |
| 44ln.00 | Phosphate/creatinine ratio |
| 44lk.00 | N-telopeptide cross link/creatinine ratio |
| 44li.00 | Chromium/creatinine ratio |
| 44lh.00 | Cobalt/creatinine ratio |
| 44lg.00 | Citrate/creatinine ratio |
| 44lf.00 | Mercury/creatinine ratio |
| 44lE.00 | Urine thiosulphate/creatinine ratio |
| 44lD.00 | Urine protein/creatinine ratio |
| 44lC.00 | Urine porphyrin/creatinine ratio |
| 44lc.00 | Urine urate/creatinine ratio |
| 44lB.00 | Urine arsenic/creatinine ratio |
| 44lb.00 | Magnesium/creatinine ratio |
| 44lA.00 | Urine vanillylmandelic acid/creatinine ratio |
| 44la.00 | ALA/creatinine ratio |
| 44l9.00 | Porphobilinogen/creatinine ratio |
| 44l7.00 | Noradrenaline/creatinine ratio |
| 44l6.00 | Mandelic acid/creatinine ratio |
| 44l3.00 | Dopamine/creatinine ratio |
| 44l1.00 | Calcium/creatinine ratio |
| 44l0.00 | Adrenaline/creatinine ratio |
| 44JF.00 | Plasma creatinine level |
| 44JD.00 | Corrected serum creatinine level |
| 44JC.00 | Corrected plasma creatinine level |
| 44J7.00 | Albumin / creatinine ratio |
| 44J3z00 | Serum creatinine NOS |
| 44J3200 | Serum creatinine normal |
| 44J3100 | Serum creatinine low |
| 44J3.00 | Serum creatinine |
| 44HJ.00 | Plasma creatinine phosphokinase MB isoenzyme level |
| 44H8.00 | Serum creatinine phosphokinase MB isoenzyme level |
| 447K.00 | Dehydroepiandrostenedione:creatinine ratio |
| 43y1.00 | Bence Jones protein/creatinine ratio measurement |
| **Anaemia - confirmed** | |
| U604100 | [X]Vit B12/folic/oth ant-megalobl-anaem caus adv ef ther use |
| U604000 | [X]Fe prep/oth anti-hypochr-anaem prep caus adv eff ther use |
| Dyu2400 | [X]Other specified anaemias |
| Dyu2200 | [X]Anaemia in other chronic diseases classified elsewhere |
| Dyu1.00 | [X]Haemolytic anaemias |
| Dyu0600 | [X]Vitamin B12 deficiency anaemia, unspecified |
| Dyu0000 | [X]Other iron deficiency anaemias |
| D2z..00 | Other anaemias NOS |
| D2y..00 | Other specified anaemias |
| D21z.13 | Macrocytic anaemia of unspecified cause |
| D21z.12 | Normocytic anaemia due to unspecified cause |
| D21z.11 | Secondary anaemia NOS |
| D21z.00 | Anaemia unspecified |
| D21yz00 | Other specified anaemia NOS |
| D21yy00 | Other specified other anaemia |
| D21y200 | Leukoerythroblastic anaemia |
| D21y.00 | Other specified anaemias |
| D215000 | Anaemia secondary to chronic renal failure |
| D215.00 | Anaemia secondary to renal failure |
| D214.00 | Chronic anaemia |
| D213.00 | Refractory Anaemia |
| D212.00 | Anaemia in neoplastic disease |
| D210z00 | Sideroblastic anaemia NOS |
| D210400 | Secondary sideroblastic anaemia due to drugs and toxins |
| D210300 | Secondary sideroblastic anaemia due to disease |
| D210200 | Pyridoxine-responsive sideroblastic anaemia |
| D210100 | Acquired sideroblastic anaemia |
| D210.00 | Sideroblastic anaemia |
| D21..00 | Other and unspecified anaemias |
| D20z.00 | Aplastic anaemia NOS |
| D204.00 | Idiopathic aplastic anaemia |
| D201z00 | Acquired aplastic anaemia NOS |
| D201700 | Transient hypoplastic anaemia |
| D201311 | Radiation aplastic anaemia |
| D201000 | Aplastic anaemia due to chronic disease |
| D201.11 | Normocytic anaemia due to aplasia |
| D201.00 | Acquired aplastic anaemia |
| D200y00 | Other specified constitutional aplastic anaemia |
| D200200 | Constitutional aplastic anaemia with malformation |
| D200111 | Fanconi's hypoplastic anaemia |
| D200011 | Constitutional aplastic anaemia without malformation |
| D200.00 | Constitutional aplastic anaemia |
| D20..00 | Aplastic anaemia |
| D2...00 | Aplastic and other anaemias |
| D0z..00 | Deficiency anaemias NOS |
| D0y..00 | Other specified deficiency anaemias |
| D01z000 | [X]Megaloblastic anaemia NOS |
| D01z.11 | Megaloblastic anaemia NOS |
| D01z.00 | Other deficiency anaemias NOS |
| D01y100 | Vitamin E deficiency anaemia |
| D01y000 | Vitamin C deficiency anaemia |
| D014000 | Amino-acid deficiency anaemia |
| D013z00 | Other specified megaloblastic anaemia NEC NOS |
| D013000 | Combined B12 and folate deficiency anaemia |
| D013.00 | Other specified megaloblastic anaemia NEC |
| D012z00 | Folate-deficiency anaemia NOS |
| D012500 | Macrocytic anaemia unspecified cause |
| D012400 | Folate-deficiency anaemia due to liver disorders |
| D012300 | Folate-deficiency anaemia due to malabsorption |
| D012.11 | Folic acid deficiency anaemia |
| D012.00 | Folate-deficiency anaemia |
| D011z00 | Other vitamin B12 deficiency anaemia NOS |
| D011X00 | Vitamin B12 deficiency anaemia, unspecified |
| D011100 | Vit B12 defic anaemia due to malabsorption with proteinuria |
| D011.11 | Vitamin B12 deficiency anaemia |
| D011.00 | Other vitamin B12 deficiency anaemias |
| D010.12 | Biermer's congenital pernicious anaemia |
| D010.11 | Addison's anaemia |
| D010.00 | Pernicious anaemia |
| D01..11 | Megaloblastic anaemia |
| D01..00 | Other deficiency anaemias |
| D00zz00 | Iron deficiency anaemia NOS |
| D00z200 | Idiopathic hypochromic anaemia |
| D00z100 | Chlorotic anaemia |
| D00z000 | Achlorhydric anaemia |
| D00z.00 | Unspecified iron deficiency anaemia |
| D00yz00 | Other specified iron deficiency anaemia NOS |
| D00y100 | Microcytic hypochromic anaemia |
| D00y.00 | Other specified iron deficiency anaemia |
| D00..12 | Microcytic - hypochromic anaemia |
| D00..11 | Hypochromic - microcytic anaemia |
| D00..00 | Iron deficiency anaemias |
| D0...12 | Sideropenic anaemia |
| D0...11 | Asiderotic anaemia |
| D0...00 | Deficiency anaemias |
| B937X00 | Refractory anaemia, unspecified |
| 2C2Z.00 | O/E - anaemia NOS |
| 2C24.00 | O/E - profoundly anaemic |
| 2C23.00 | O/E - clinically anaemic |
| 2C22.00 | O/E - equivocally anaemic |
| 2C2..11 | O/E - anaemic |
| 2C2..00 | O/E - anaemia |
| 1458.00 | History of sickle cell anaemia |
| 1454.00 | H/O: anaemia NOS |
| 1453.00 | H/O: haemolytic anaemia |
| 1452.00 | H/O: Anaemia vit.B12 deficient |
| 1451.00 | H/O: anaemia - iron deficient |
| 145..11 | H/O: anaemia |
| 423B.00 | Haemoglobin abnormal |
| 4236.00 | Haemoglobin borderline low |
| 4235.00 | Haemoglobin low |
| 4234.00 | Haemoglobin very low |
| 4292.00 | MCHC - borderline low |
| 4286.00 | MCH - abnormal |
| 4285.00 | MCH - borderline raised |
| 4284.00 | MCH - raised |
| 4283.00 | MCH - low |
| 4282.00 | MCH - borderline low |
| **Hypercalcaemia - confirmed** | |
| C354100 | Hypercalcaemia NEC |
| C354111 | Secondary hypercalcaemia |
| 44I8100 | Raised serum calcium level |
| 44I3.00 | Electrolytes abnormal |
| 46P4100 | Urine calcium abnormal |
| **Renal impairment - confirmed** | |
| 7L1A.00 | Compensation for renal failure |
| 7L1A.11 | Dialysis for renal failure |
| 7L1Ay00 | Other specified compensation for renal failure |
| 7L1Az00 | Compensation for renal failure NOS |
| 7L1B.00 | Placement ambulatory apparatus compensation renal failure |
| 7L1By00 | Placement ambulatory apparatus- compensate renal failure OS |
| 7L1C.00 | Placement other apparatus for compensation for renal failure |
| 7L1Cy00 | Placement other apparatus- compensate for renal failure OS |
| 7L1Cz00 | Placement other apparatus- compensate for renal failure NOS |
| C353600 | Renal failure-associated hyperphosphataemia |
| G222.00 | Hypertensive renal disease with renal failure |
| G233.00 | Hypertensive heart and renal disease with renal failure |
| K04..00 | Acute renal failure |
| K04..11 | ARF - Acute renal failure |
| K043.00 | Acute drug-induced renal failure |
| K043000 | Acute renal failure due to ACE inhibitor |
| K043100 | Acute renal failure induced by aminoglycoside |
| K043300 | Acute renal failure induced by cyclosporin A |
| K043400 | Acute renal failure induced by non-steroid anti-inflamm drug |
| K044.00 | Acute renal failure due to urinary obstruction |
| K045.00 | Acute renal failure due to non-traumatic rhabdomyolysis |
| K04B.00 | Acute renal failure due to traumatic rhabdomyolysis |
| K04y.00 | Other acute renal failure |
| K04z.00 | Acute renal failure NOS |
| K05..00 | Chronic renal failure |
| K05..12 | End stage renal failure |
| K050.00 | End stage renal failure |
| K06..00 | Renal failure unspecified |
| K08y300 | Renal function impairment with growth failure |
| K0E..00 | Acute-on-chronic renal failure |
| Kyu2.00 | [X]Renal failure |
| Kyu2000 | [X]Other acute renal failure |
| Kyu2100 | [X]Other chronic renal failure |
| SK05.00 | Renal failure following crush syndrome |
| SK05.11 | Renal failure after crushing |
| SK08.00 | Acute renal failure due to rhabdomyolysis |
| SP15400 | Renal failure as a complication of care |
| SP15412 | Post operative renal failure |
| K06..12 | Kidney failure unspecified |
| SP08300 | Kidney transplant failure and rejection |
| SP15411 | Kidney failure as a complication of care |
| 4513100 | Creatinine clearance-glom filt abnormal |
| 44J3300 | Serum creatinine raised |
| 44J3000 | Serum creatinine abnormal |
